# Evolutionary landscape of plant chalcone isomerase-fold gene families

**DOI:** 10.3389/fpls.2025.1559547

**Published:** 2025-03-28

**Authors:** Kai-yong Luo, Shi-ping Wang, Ling Yang, Sen-lin Luo, Jia Cheng, Yang Dong, Ya Ning, Wei-bin Wang

**Affiliations:** ^1^ College of Food Science and Technology, Yunnan Agricultural University, Kunming, Yunnan, China; ^2^ Yunnan Provincial Key Laboratory of Biological Big Data, Yunnan Agricultural University, Kunming, Yunnan, China; ^3^ Institute of Agro-Products of Processing and Design, Hainan Academy of Agricultural Sciences, Haikou, China; ^4^ Department of Pain Management, The Second Affiliated Hospital of Kunming Medical University, Kunming, Yunnan, China; ^5^ College of Science, Yunnan Agricultural University, Kunming, Yunnan, China

**Keywords:** chalcone isomerase, evolution, flavonoids, diversity, structural cluster analysis

## Abstract

Flavonoids are crucial for plant survival and adaptive evolution, and chalcone isomerase (CHI) genes serve as key rate-limiting gene in the flavonoid biosynthesis pathway. It is important for plant adaptive evolution to comprehensively study the evolution and diversity of the CHI gene families. However, the CHI gene families in many plant lineages remain elusive. This study systematically identified CHI genes from 259 species including algae, bryophytes, ferns, gymnosperms, and angiosperms. A total of 1,738 CHI gene family members were discovered. We analyzed the diversity, distribution trajectory, and the driving forces of gene duplication during the evolution of the plant lineages. The present study is the first to identify potential type II and type IV CHI genes in the extant liverwort model species *Marchantia polymorpha*. The distribution pattern of CHI genes across the plant kingdom reveals that the origin of type II CHI can be traced back to the last common ancestor of bryophytes and vascular plants, and type III CHI may represent the ancestral form of the CHI gene family. The identification of conserved motifs showed significant differences in motif distribution among different CHI gene types. It was found that the drivers of gene duplication varied across plant lineages: dispersed duplications (DSD) were predominant in algae and bryophytes, whole-genome duplication (WGD) was the main driver in basal angiosperms and monocots, while tandem duplications (TD) predominating in eudicots. Structural clustering analysis demonstrated the 3-layer sandwich structure in the CHI-fold proteins remained conserved in the central region, while repeated loss of N-terminal sequences contributed to structural diversity. This study provides a deeper understanding of the evolution and diversity of the CHI-fold proteins and lays a theoretical foundation for further studies of their function and the identification of new functional CHI genes.

## Introduction

During the colonization and evolution of terrestrial plants, a vast array of specialized metabolites has been developed to facilitate adaptation and response to a diverse range of biotic and abiotic stresses (Winkel-Shirley, 2002; Agati et al., 2012; Petrussa et al., 2013; Dias et al., 2021). Flavonoids are one of the largest groups of (poly)phenolic compounds, which are extensively distributed in the plant kingdom, and played a pivotal role in terrestrial colonization and adaptation in plant evolution (Mouradov and Spangenberg, 2014; Yonekura-Sakakibara et al., 2019). Flavonoids in plants are critical for a variety of biological processes, including protection against ultraviolet-B (UV-B) radiation, pollinators attraction, phytoalexins, signaling molecules, and auxin transport and fertility regulation (Agati et al., 2012). It is hypothesized that in early flavonoid-producing plants, their primary functions were to provide defense against UV-B exposure and the regulation of plant hormone activity (Rausher, 2006). Over the course of plant evolution, these functions have diversified considerably. The biosynthesis of plant flavonoids primarily originates from phenylalanine metabolism and branches off from the general phenylpropanoid pathway through the catalytic actions of chalcone synthase (CHS) and chalcone isomerase (CHI) (Liu et al., 2021). As the pivotal enzyme in the flavonoid biosynthetic pathway, CHI catalyzes the intramolecular and stereospecific cyclization of chalcone through the Michael addition reaction, thereby facilitating the formation of the fundamental scaffold for subsequent flavonoid synthesis. Subsequently, a diverse array of flavonoids is produced through the modification of superfamilies such as cytochrome P450, oxidoreductase, and UDP glycosyltransferase (Yin et al., 2019).

Chalcone isomerase (CHI-fold proteins, CHI, EC: 5.5.1.6) was first identified and isolated in the study of the mechanism of synthesis of Phenylpropanoid-like natural products in *Phaseolus vulgaris* under environmental stress, and the key role of chalcone isomerase in the synthesis of flavonoids was discovered (Mehdy and Lamb, 1987). After that, CHI has been cloned and characterized in many plants, including *Arabidopsis thaliana* (Shirley et al., 1992), rice (Druka et al., 2003), *Petunia hybrida* (Van Tunen et al., 1988), soybean (Ralston et al., 2005), and alfalfa (McKhann and Hirsch, 1994). Based on sequence similarity and function, CHI-fold proteins were classified into four subfamilies: type I, type II, type III, and type IV (Zamora et al., 2013; Zu et al., 2019; Lin et al., 2021; Wang et al., 2022b). Studies on CHI-fold proteins in *Arabidopsis* further categorized the type III CHI subfamily into three subclasses, AtFAP1, AtFAP2, and AtFAP3 (Ngaki et al., 2012). Recent studies have also categorized type II CHI into two subgroups, CHIA and CHIB, based on their differentiation in the function of symbiotic nitrogen fixation in rhizomes (Liu et al., 2024).

Together, type I and type II CHIs are referred to as *bona fide* CHIs or active CHIs, catalyzes the cyclization of 4,2’,4’,6’-tetrahydroxychalcone (also known as chalcone) to form naringenin. Although this process is capable of spontaneous reaction, the reaction catalyzed by CHI is 10^7^-fold more efficient and produces specific 2S-stereoisomers (important precursors for downstream hydroxylation), which lead to the synthesis of various flavonoids and isoflavonoids (Jez et al., 2000). In addition, type II CHIs have acquired a new function over type I, due to a substitution of key catalytic residues, enabling the additional cyclization of isoliquiritigenin to 5-deoxy flavanone, i.e., (2S)-liquiritigenin (Shimada et al., 2003; Dastmalchi and Dhaubhadel, 2015). Previous research has shown that the presence of multiple catalytic residues within the CHI protein, along with the hydrogen bonding interactions between the protein and its substrate, is crucial for its catalytic activity (Jez et al., 2000, 2002). In contrast, types III and IV are not catalytically active because they lack one or more key active sites (Dastmalchi and Dhaubhadel, 2015). Although type IV CHI (also known as CHI-like) does not have chalcone isomerase activity, unlike type III CHI, it still plays a crucial role in the flavonoid biosynthetic pathway (Sugimoto et al., 2024). For example, in *Humulus lupulus*, type IV CHI increased the production of 2’,4,4’,6’-tetrahydroxychalcone and reduced the formation of by-products by binding to CHS (Ban et al., 2018). This function has since been shown to be widespread across plants (Waki et al., 2020).

The essential functions of CHI-fold proteins and flavonoid compounds have led to their retention during plant adaptive evolution. Type I CHI is present in almost all vascular plants. Type IV CHI is predominantly identified in terrestrial plants. In contrast, ype III CHI exhibits a broad distribution across the plant kingdom and is also present in homologous structures within certain fungi and bacteria (Gensheimer and Mushegian, 2004). However, the origin and distribution of type II CHI have been subjects of considerable debate. Initial investigations posited that type II CHI was confined to leguminous plants (Shimada et al., 2003); subsequent research, however, has demonstrated its occurrence in ferns and even in bryophyte lineages (Cheng et al., 2018; Ni et al., 2020). This complex distribution pattern, along with the origin and evolution of early CHI-fold proteins, remains an unresolved puzzle. Subsequent studies, including the analysis of the higher structure of type III CHI members in *Arabidopsis*, combined with phylogenetic analyses, have revealed that CHI-fold proteins likely originated from fatty acid-binding proteins (FAPs) (Ngaki et al., 2012). Since then, the evolution of CHI-fold proteins has been widely studied. Research on the type CHI I and II genes in 52 fern species suggests that the emergence of type I CHI may coincide with the divergence of ferns (Ni et al., 2020). Additionally, studies on CHI-fold proteins in 15 species also indicate that type III CHI is the common ancestor of the other CHI types, which evolved from the common ancestor FAP3 (Wang et al., 2022b). This evolutionary path, in green plants, has undergone continual structural evolution and gene duplication under natural selection pressures.

Despite substantial research on the evolution and diversity of CHI-fold proteins in certain model plants and specific plant groups, a comprehensive exploration of their evolutionary history and diversity across the entire plant kingdom remains incomplete. This study aims to elucidate the origin and evolutionary trajectory of the CHI-fold proteins in plants by conducting an extensive investigation into the distribution of CHI-fold protein members across various lineages. It also examines the mechanisms driving gene duplication and the evolutionary relationships, utilizing a wide array of plant and algal genomic data. Furthermore, AlphaFold was utilized to predict the tertiary structures of CHI-fold protein members across diverse lineages, further enhancing our understanding of the structural variations. This research enhances our comprehension of the evolution and diversity of the CHI-fold proteins and establishes a theoretical foundation for further investigation into the functions of the CHI-fold proteins, as well as the identification of novel functional CHI genes.

## Results

### Identification and phylogenetic analysis of CHI-fold protein families in plants

**Figure 1:**
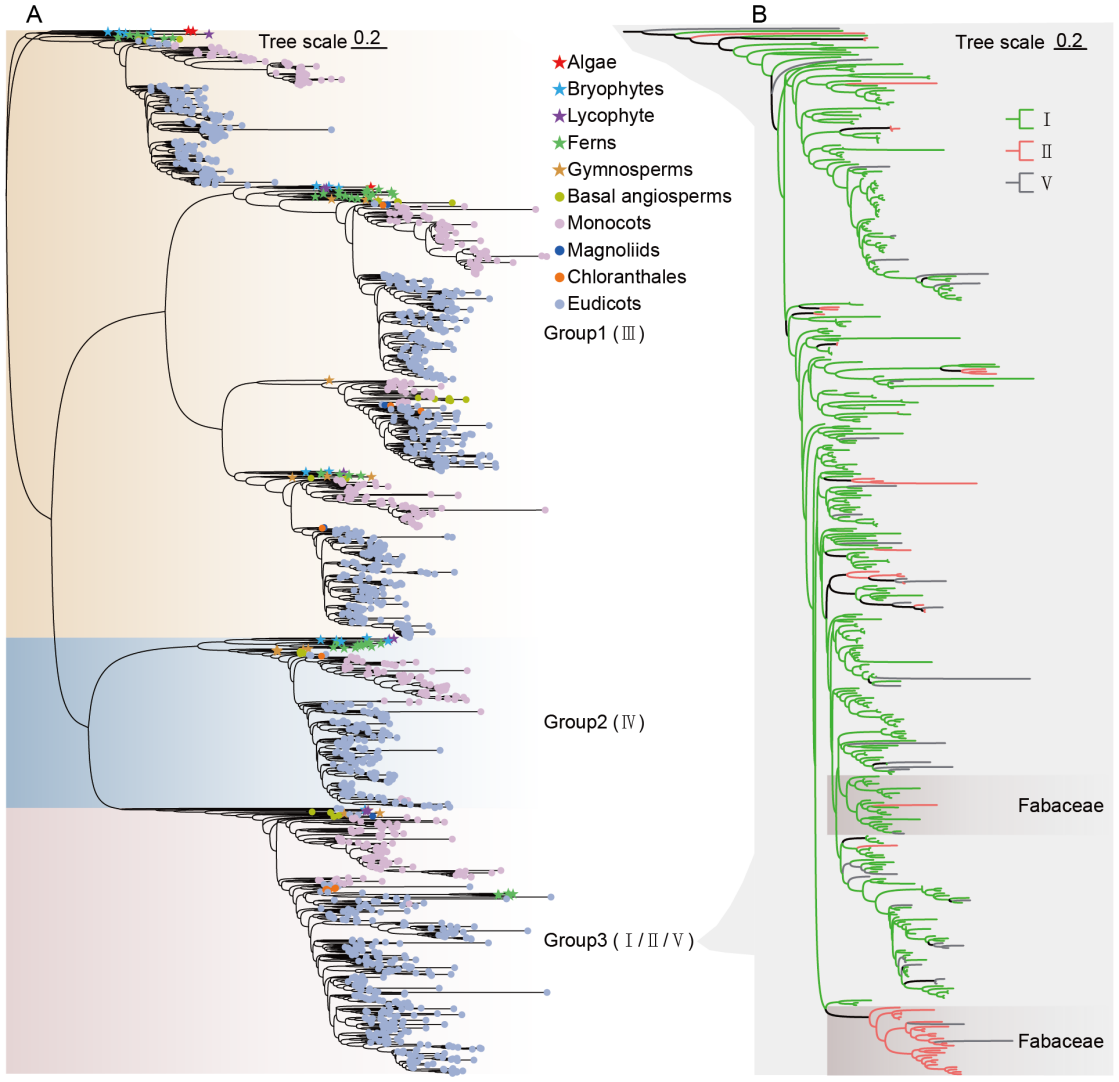
Phylogenetic tree of CHI-fold proteins. **(A)** Maximum likelihood (ML) phylogenetic tree of CHI-fold proteins of 259 species. **(B)** Detailed maximum likelihood phylogenetic tree of types I/II/V CHI in Group 3. Different branch colors represent different types of genes, and the background of the branch where legume CHI genes cluster is highlighted.

To systematically investigate the CHI gene families in plants, Hidden Markov Model (HMM) methodologies were employed to identify CHI protein sequences from 259 species genomes that cover different plant lineages (**Supplementary Table S1**). In total, 1,738 CHI sequences were identified (**Supplementary Table S2**). CHIs were present in all species investigated, ranging from algae to angiosperms. To investigate the evolutionary relationships, a maximum likelihood (ML) phylogenetic tree was constructed using IQ-TREE, which classified 1,738 CHI sequences into three groups (**Figure 1A**; **Supplementary Figure S2**). Group 1 consists predominantly of type III CHIs (1016), Group 2 comprises type IV (282), and Group 3 includes a heterogeneous mix of CHI types. Notably, type I and type II CHIs did not fully resolve into separate branches in Group 3. Previous studies have indicated that the types I and II CHIs can be effectively distinguished by analyzing the amino acids at the active site, particularly at position 190 (Dastmalchi and Dhaubhadel, 2015; Ni et al., 2020). Specifically, a threonine (T) residue at position 190 is characteristic of type II CHI (56), while a serine (S) residue indicates type I CHI (347). In the sequences of Group 3, we observed other amino acid residues besides threonine and serine at position 190, which will be classified as type V CHI (37) (**Figure 1B**; **Supplementary Figures S3**–**S5**).

Group 1, encompassing type III CHIs, is widely distributed across a wide range of plant lineages including algae, bryophytes, lycophytes and ferns, and spermatophytes. This extensive phylogenetic distribution implies that type III CHI genes may represent the earliest evolutionary form within the CHI family, having been extensively conserved throughout subsequent plant evolutionary processes. Group 2 is the type IV CHIs, which have been primarily retained in land plants, and have undergone radiation and expansion in the angiosperm lineage, highlighting their potential role in early terrestrial adaptation of plant lineages. The distinction between types I, II, and V CHIs within Group 3 showed extensive divergence, especially for type II CHIs, which are notably enriched in legumes (**Figure 1B**).

Additionally, gene characteristics such as length, isoelectric point, molecular weight, and subcellular localization were analyzed for each CHI type (**Supplementary Tables S2**, **S3**). The mean value of the isoelectric point of gene members was 7.04 (neutral), of molecular weight was 30,160 Da, and of gene length was 275 amino acids (aa). Notably, type III CHIs are significantly longer, averaging 309 amino acids, while the other types exhibit minimal length variation. Subcellular localization analysis revealed that types I, II, and IV CHIs are predominantly cytoplasmic (cyto), while types III and V are mostly chloroplastic (chlo).

### Distribution and evolution of CHI-fold protein families in plants

**Figure 2:**
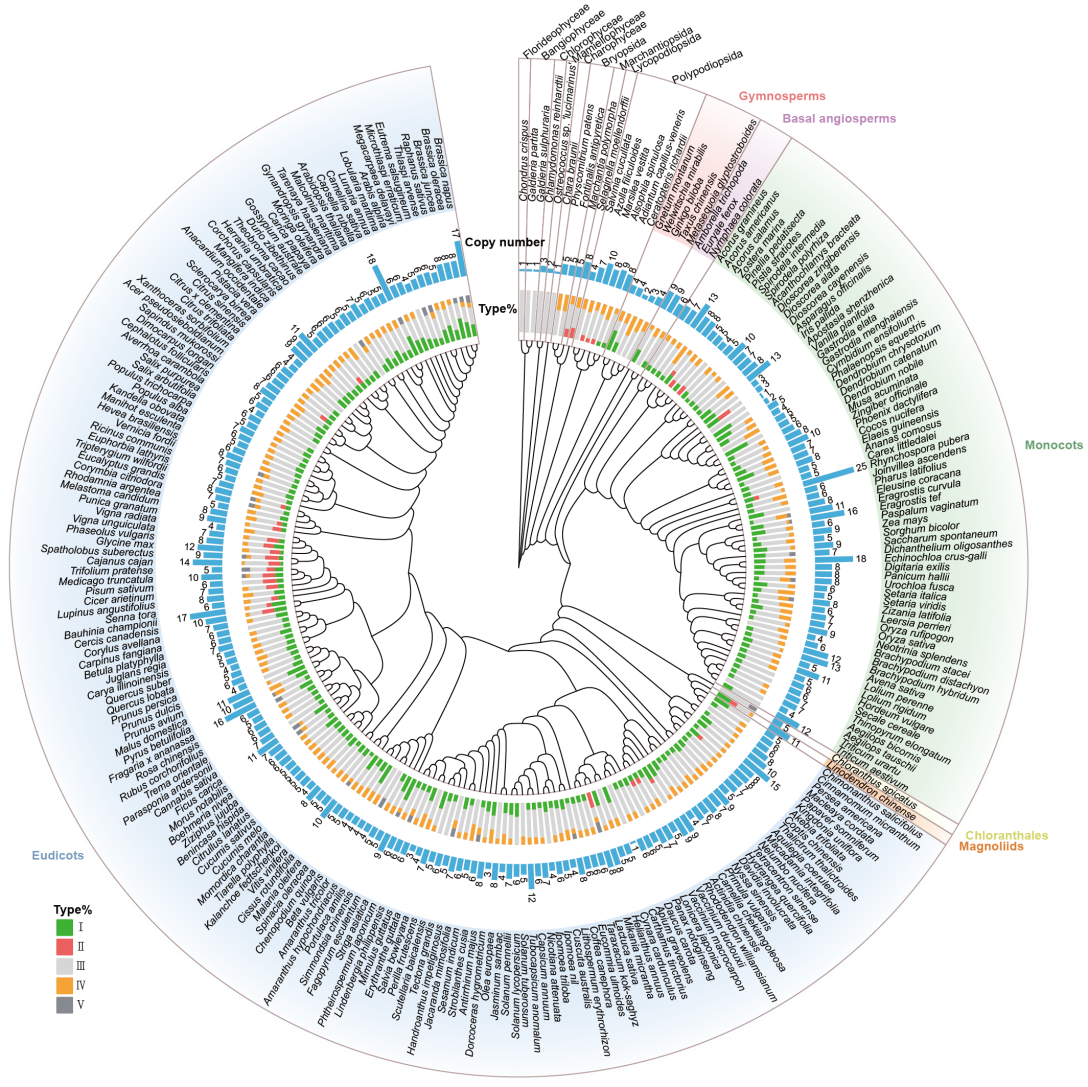
Distribution and evolutionary history of CHI-fold protein families in plants. Phylogenetic tree of 259 species with the copy number of each species and the proportion of each type in each plant. Specifically, algae include charophyceae, chlorophyceae, florideophyceae, and mamiellophyceae; bryophytes include bryopsida and marchantiopsida; and lycophytes and ferns include lycopodiopsida and polypodiopsida, respectively.

To explore the origin, distribution, and evolutionary history of the CHI-fold proteins in plants, we constructed a phylogenetic tree for 259 species (**Supplementary Figure S1**). Using the BUSCO dataset, we identified 196 single-copy homologous genes with more than 80% coverage. ASTRAL merging results indicated that quartet trees from 85% of the gene trees appeared in the final species tree. The phylogenetic tree reconstructed in this study is highly similar to the recently published tree of life (Zuntini et al., 2024). Subsequently, we assessed the number of CHI gene copies (Contains types I/II/III/IV/V) in each species and calculated the proportion of each CHI type (**Figure 2**). Algae (Charophyceae, Florideophyceae, Bangiophyceae, and Mamiellophyceae) contain 1-3 copies of CHI genes, while bryophytes, including Bryopsida and Marchantiopsida, have an average of 5 copies, suggesting CHI gene expansion may be related to terrestrial adaptation (such as increased UV exposure, desiccation, and pathogen resistance). Among lycophytes (Lycopodiopsida) and ferns (Polypodiopsida), the average number of CHI gene copies further increases to 7.71, and *Marsilea vestita* possesses up to 10 copies. Moreover, most species possess at least one copy of the type I or type II CHI gene. Considering that the synthesis of naringenin from 4,2’,4’,6’-tetrahydroxychalcone occurs much less efficiently when the process occurs spontaneously than under the action of types I/II CHI. Lycophytes and ferns may have benefitted from elevated CHI gene copy numbers and the evolution of active CHI to support more robust flavonoid biosynthesis, essential for vascular development and stress adaptation. Gymnosperms display relatively lower CHI copy numbers (2-4), whereas angiosperms exhibit considerable variation, ranging from 1 to 25. This substantial variability likely reflects their ecological diversity and adaptive requirements for specialized flavonoid functions.

We classified members of the CHI-fold proteins into five types, I to V, based on phylogenetic analyses and amino acid residue analyses. These types have emerged progressively across diverse plant lineages, illustrating their functional diversification and emphasizing the crucial role of flavonoid metabolism in plant adaptation to terrestrial habitats (**Figure 2**; **Supplementary Figure S6**). In algae, only the non-catalytic type III CHI gene is present, implying that type III may represent the ancestral form of the CHI-fold proteins in plants. During the process of plant terrestrialization, other types of CHIs gradually evolved. Types II and IV CHI genes were first identified in basal bryophyte liverwort (*M. polymorpha*), where their presence likely represents metabolic adaptations necessary for coping with environmental stresses associated with terrestrial habitats, such as ultraviolet radiation and desiccation. Type IV CHI is nearly ubiquitous across land plants, indicating its essential role in plant evolution. However, type II CHI displays a more sporadic distribution, being present only in ferns, bryophytes, legumes, and a limited number of core angiosperms, while absent in gymnosperms and basal angiosperms, suggesting it has lineage-specific retention. Type I CHI, the primary enzyme catalyzing chalcone isomerization in plants, was identified in ferns, and then widely conserved across vascular plants, further underscoring the importance of flavonoid pathways in plant adaptive evolution. Type V CHIs, which are evolutionarily recent, appear sporadically in only a few core angiosperms. In summary, the CHI-fold proteins have experienced numerous expansions and diversifications throughout the evolutionary transition from aquatic to terrestrial habitats. Notably, bryophytes already exhibit a CHI-fold protein repertoire that closely resembles that of angiosperms.

### Gene structural characteristics of different CHI types in representative species

**Figure 3:**
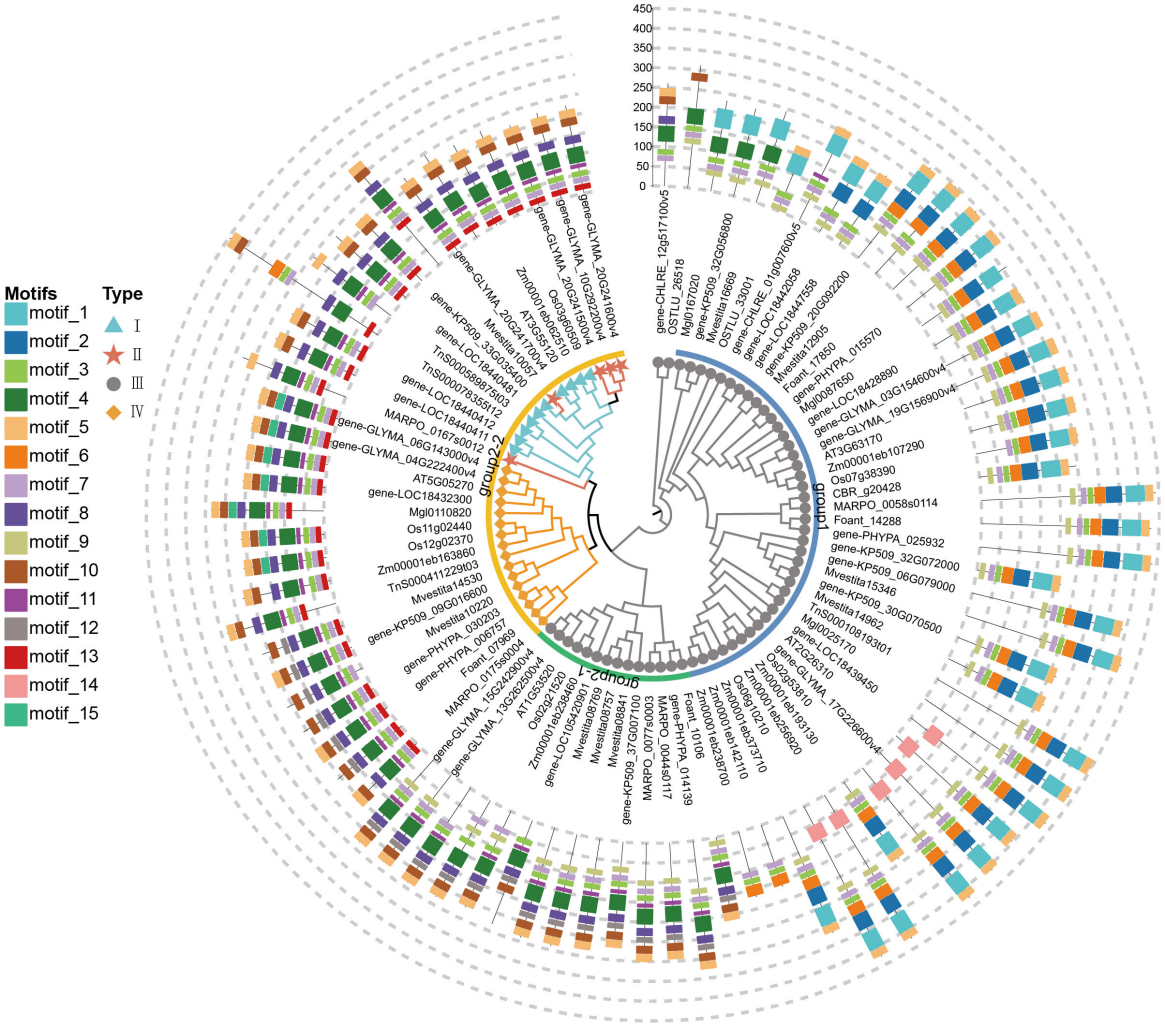
Conserved motif identification and distribution of representative species studied. The phylogenetic tree was obtained using the maximum likelihood method. Each gene's corresponding taxa are distinguished based on the prefix of its label name: two algal species (OSTLU and gene-CHILRE), three bryophyte species (MARPO, Foant, and gene-PHYPA), two fern species (Mvestita and gene-KP509), two gymnosperm species (Mgl and TnS), one basal angiosperm species (gene-LOC), two monocot species (Os and Zm), and two eudicot species (gene-GLYMA and AT).

The conserved motifs of CHI-fold proteins were analyzed for 15 representative species using the MEME software, with the maximum number of conserved motifs set to 15 (**Supplementary Table S4**; **Supplementary Figure S8**). There were large variations in the motifs among the different types of CHI genes (**Figure 3**). Overall, there are three shared motifs (motif 3, motif 5, and motif 7) in all CHI genes, which originated in early algae and remain highly conserved across all plant lineages. The type III CHI subfamily exhibits two distinctly diverged clades, showing significant variability in conserved motifs. Group 2-1 may be an important intermediate transitional CHI type containing specific motifs (motif 4, motif 8, motif 10, motif 11), which are conserved in type IV, type II, and type I CHIs. These motifs were already present in algae. In contrast, another branch of type III (Group 1) is primarily characterized by motif 1 and motif 2. Motif 9 was exclusive to type III and absent in types I, II, and IV. The motif distribution of type I and II CHIs were identical, probably due to their high similarity in conserved domain sequences. Types I, II, and IV shared a conserved motif 13, which originated from bryophytes. Type IV CHIs have an additional motif 12 or motif 15 upon the active CHI (types I and II). The motif 15 appeared initially in gymnosperms, whereas the loosed motifs at the corresponding position in the active CHI has not been replaced by other motifs. Motif 4, motif 8, and motif 10 appeared in some sequences of early algae and remain conserved in Group 2-1 of type III CHI, while completely absent in other branches. Interestingly, despite their distant evolutionary relationship, type II and type IV CHI in bryophytes exhibit highly similar motif structures to those found in vascular plants, including type I, type II, and type IV. This suggests that these structures may have originated from the last common ancestor of the two lineages.

### Analysis of duplication types of CHI families

**Figure 4:**
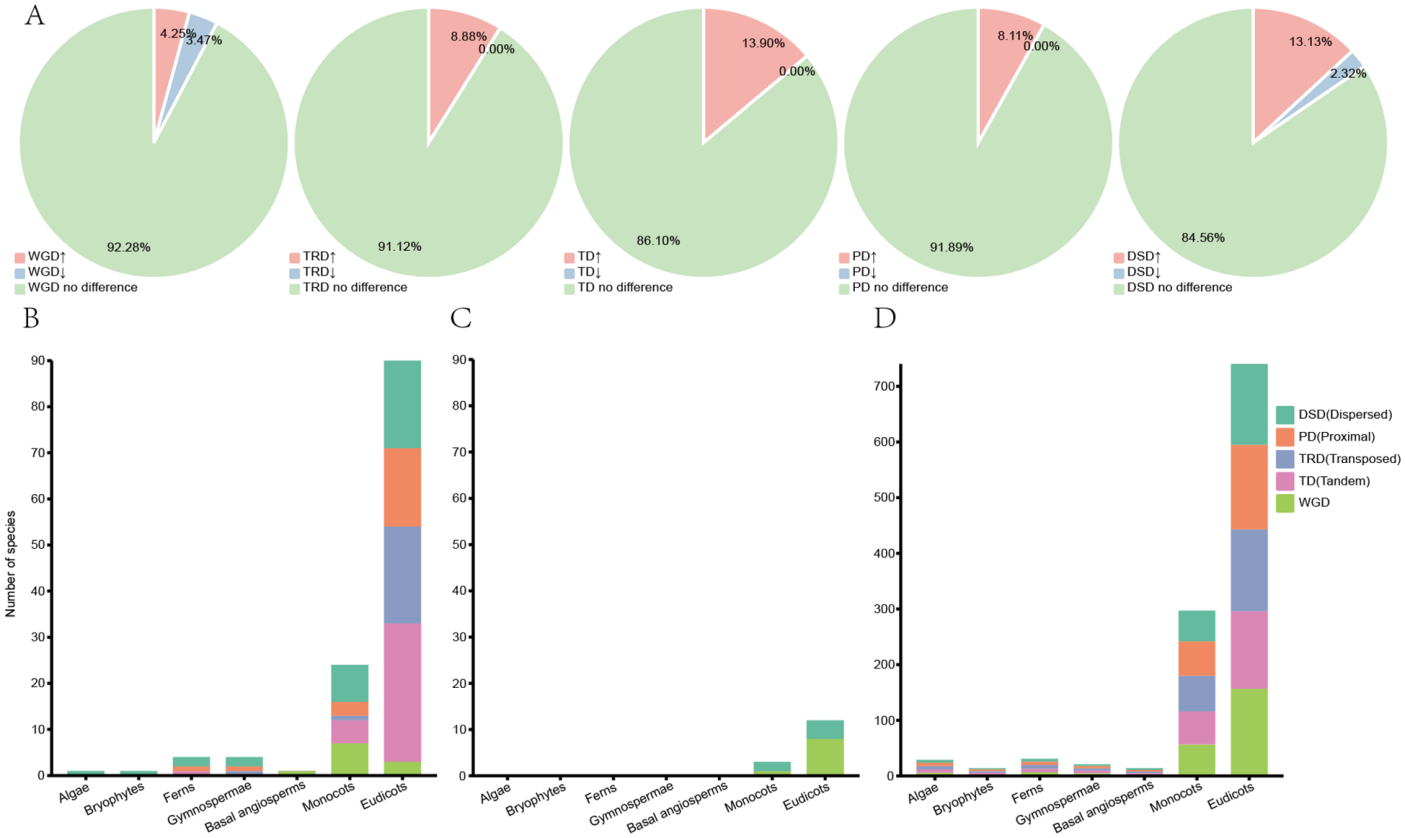
Duplication types and significance analysis for 259 species. **(A)** The proportion of species with significantly enriched or significantly reduced CHI family genes for each duplication type to the total number of species. **(B)** The number of duplicate types that are significantly enriched in the CHI gene family for each taxon. **(C)** The number of duplicate types that are significantly reduced in the CHI gene family for each taxon. **(D)** The number of duplicate types that are not significantly different in the CHI gene family for each taxon.

Gene duplication serves as the main driver of metabolic pathway diversification and gene neofunctionalization, and exploring gene duplication types could further elucidate the evolution of the CHI-fold proteins (Long et al., 2003). Duplication event types were identified at the whole-genome and gene family levels in 259 species using Dupgene_finder (Qiao et al., 2019). Five types of gene duplication events were identified, including whole-genome duplications (WGD), tandem duplications (TD), transposed duplications (TRD), proximal duplications (PD), and dispersed duplications (DSD) (**Supplementary Tables S5**–**S9**; **Supplementary Figure S9**). The significance analysis was conducted on all identified CHI genes to explore differences in duplication types at the genome-wide level and their specificity across various plant lineages (**Figure 4**).

The analysis revealed that, among the five major duplication types, tandem duplication genes were significantly enriched in the CHI-fold proteins, exhibiting the highest percentage of enrichment (13.9%) across 36 species, and no significant reduction was observed (**Figure 4A**; **Supplementary Table S6**). No significant difference was detected in the remaining 223 species. The DSD was the second richest, significantly enriched in 34 species, 6 species significantly reduced, and 219 species without significant difference. The WGD, PD, and TRD were significantly enriched in 11, 21, and 24 species, respectively, significantly reduced in 9, 0, and 0 species, and not significantly different in 239, 238, and 235 species (**Supplementary Tables S5**, **S7**, **S8**). Overall, species with no significant differences accounted for a high proportion of all examined types.

Statistical analyses showed that DSD was the main type in algae and bryophytes. Only whole genome duplication was detected in basal angiosperms. In ferns and gymnosperms PD and DSD dominated, with similar proportions. Monocots showed significant enrichment in all five duplication types, with WGD being the most frequent. In eudicot, the highest proportion of TD, followed by DSD, PD and TRD, while WGD had the lowest proportion (**Figure 4B**). In terms of significant reduction duplications were not found in algae, bryophytes, ferns, gymnosperms, and basal angiosperms. However, monocots and eudicots showed significant reductions in DSD and WGD (**Figure 4C**). In summary, DSD was the main duplication type of CHI gene expansion in algae and bryophytes. In ferns and gymnosperms, PD and DSD were the main types. In basal angiosperms and monocots WGD was the main force driving the expansion of the CHI-fold proteins, whereas in eudicots TD was the main driver.

### Identification of duplicated gene types and Ka/Ks calculations

**Figure 5:**
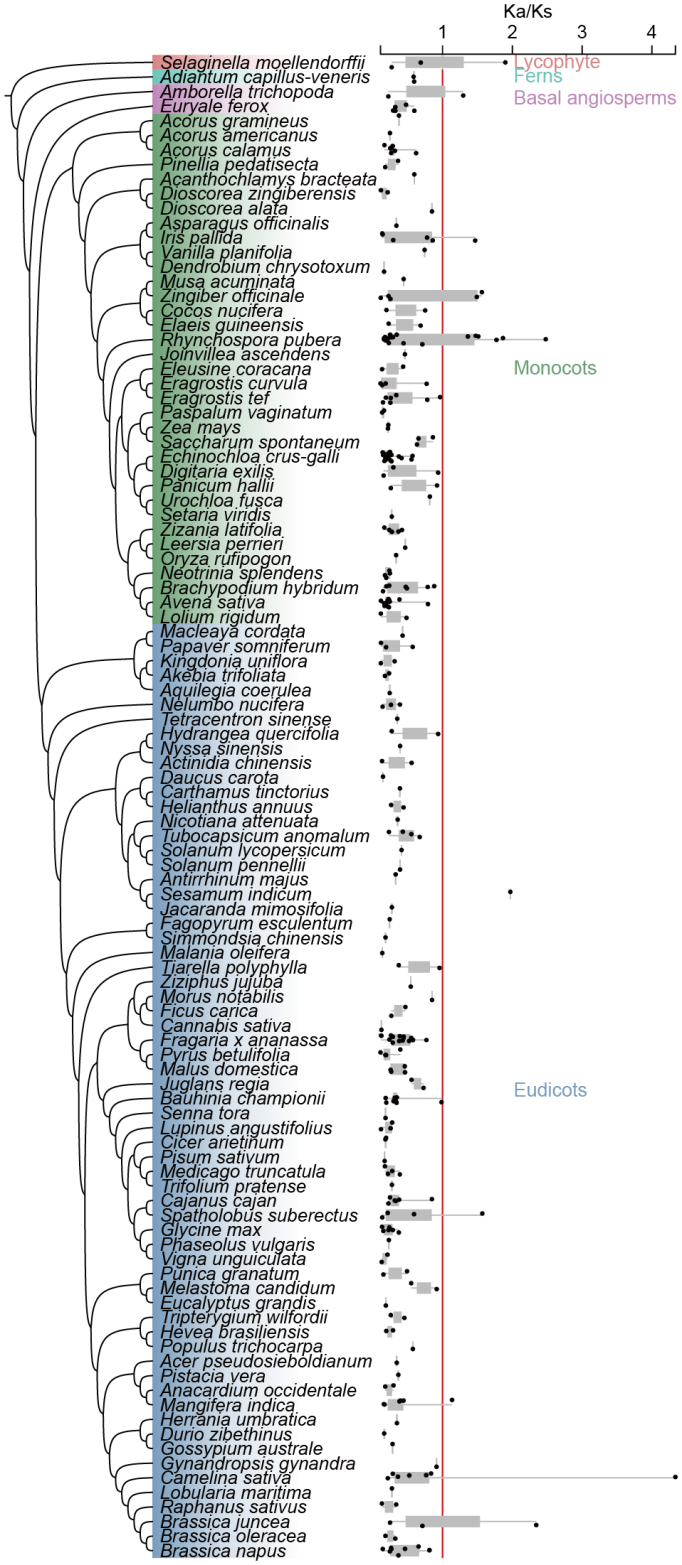
Ka/Ks values of 103 species studied. The box plot represents the Ka/Ks value and distribution of each species and the red line represents the location where the Ka/Ks value is equal to 1.

The diversification of the chalcone isomerase-fold proteins has been significantly enhanced by gene duplication and sub-functionalization, particularly among eudicots, where whole-genome duplication (WGD) and tandem duplication have served as the primary driving forces. To explore the selection pressures driving CHI-fold protein expansion and diversification, we calculated the Ka and Ks values for CHI gene pairs derived from WGD and TD events using the calculate_Ka_Ks_pipe.pl programme of DupGen_finder (**Supplementary Tables S10**, **S11**). Species with no detectable gene pairs generated by WGD and TD were removed, and Ka/Ks values were calculated for the remaining 103 species duplicate gene pairs. The results showed that homozygous gene pairs with Ka/Ks values more than 1 accounted for 6.14% of the total gene pairs, while homozygous gene pairs with Ka/Ks values less than 1 accounted for 93.86% of the total gene pairs, suggesting that the CHI-fold protein was subjected to strongly purifying selection. However, in lycophyte ferns, basal angiosperm, monocots, and eudicots there were individual species, respectively, that had CHI gene pairs with Ka/Ks values greater than 1. A group of type I gene pairs in *Camelina sativa* had a Ka/Ks value of type I gene pair of 4.3, suggesting that this gene pair may have experienced strong selection pressure in this species (**Figure 5**; **Supplementary Table S10**).

### Structural diversity and evolutionary analysis of the CHI-fold proteins

**Figure 6:**
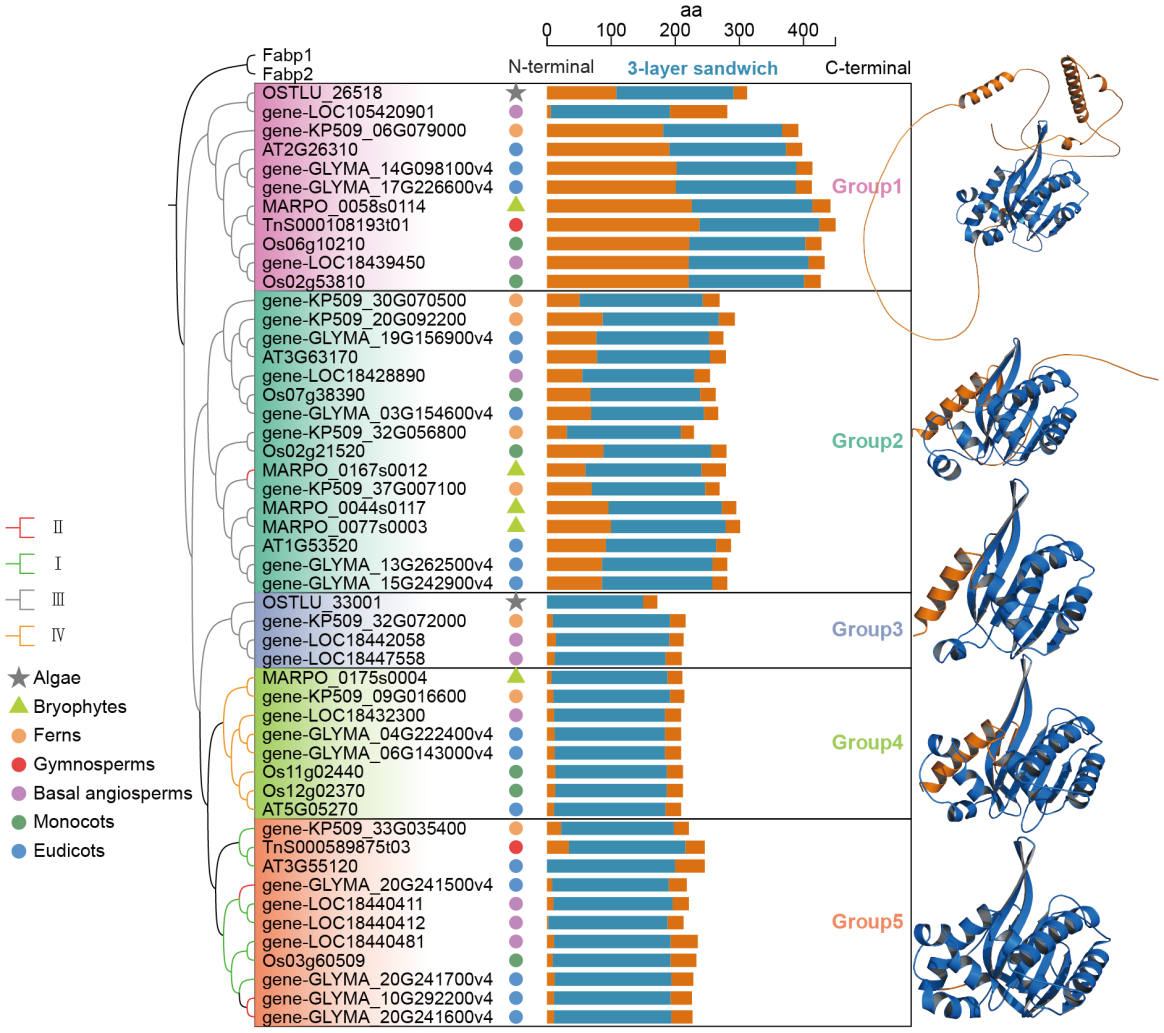
The structural clustering of the studied CHI-fold proteins and the representative 3D structure of each group. In the representative 3D structures of CHI proteins from each group, the blue regions represent the 3-layer sandwich region.

To explore the structural diversity of CHI proteins, protein structure clustering was referred to the method of Caixia Gao et al (Huang et al., 2023). the three-dimensional structures of 51 protein sequences from eight representative species were predicted by AlphaFold3, with two fabp proteins containing the PF00061 domain selected as outgroups. Protein structure alignment was performed using US-align based on TM-value, and a normalized similarity matrix was generated. The UPGMA hierarchical clustering method was used to construct a dendrogram to display structural similarities. The result of clustering revealed that CHI proteins were classified into five groups, with differences in the structure of each group (**Figure 6**). The 3-layer sandwich structure was located in the central region of all CHI family members and showed high structural conservation. N-terminal structures of all CHI proteins displayed abundant diversity, whereas the C-terminal region was conserved.

Group 1 exhibited the most complex protein folding structure, present in all lineages and consisting entirely of type III CHI. Group 2 was found in all lineages except algae, and displayed a similar structure to Group 1. However, Group 2 was characterized by a significantly longer and irregularly coiled structure at the N-terminal end. Furthermore, the irregularly coiled region of Group 1 comprises two segments of α-helices. Group 3, Group 4, and Group 5 had significantly shorter N-terminal irregularly curled structures compared to Group 1 and Group 2, but still retained some segments. In particular, Group 2 contains a type II CHI gene (MARPO_0167s0012) from the bryophyte *M. polymorpha*, which exhibited an overall structural similarity to the other members within the group. This observation implies that early active CHI proteins may have preserved an N-terminal disordered region akin to that found in type III CHI, or alternatively, the mechanisms underlying the retention of the N-terminal structure of these genes exhibit lineage-specificity across different plant lineages. The type III CHI genes were classified into three groups, each demonstrating notable variations in their N-terminal regions. Among them two type III CHI genes from algae were identified in Group 1 and Group 3, respectively, indicating that the structural diversity of type III CHI genes had emerged during the algae.

## Discussion

Chalcone isomerase is a key rate-limiting enzyme in the flavonoid biosynthesis pathway and is crucial for plant survival and adaptation (Yin et al., 2019; Wang et al., 2022b). Cross-lineage studies among gene families not only provide a more comprehensive understanding of gene family conservation and variability during evolution but also greatly reduce the possibility of biased conclusions due to the limitations of the results derived from studies on single or few species (Naake et al., 2021; Liu et al., 2024). In this study, a total of 1,738 CHI-fold proteins were identified across 259 species spanning the green plant lineage, and the basic information statistics showed that almost all of the CHI type III were longer than the other types, which was related to the irregularly curled structure at their N-terminal regions at the N-end (**Figure 6**). Subcellular localization analysis showed that types III and V are predominately localized in chloroplasts, consistent with their role in fatty acid synthesis (Ngaki et al., 2012). Whereas, types I, II, and IV CHIs are mainly localized in the cytoplasm, consistent with the fact that flavonoid biosynthesis takes place in the cytoplasm (Kitamura, 2006). 12 CHI genes were identified in *Glycine Max*, an important species for the study of the CHI family, consistent with the findings of Mehran DastMalchi et al (Dastmalchi and Dhaubhadel, 2015). where 9 and 5 CHIs were identified in maize and grape, respectively. The number of CHI family copies showed an increasing trend from lower to higher plants, especially during the plant terrestrialization, where the active CHI-fold protein members (types I and II) went from absent to abundantly expanded (**Figure 2A**; **Supplementary Figure S1**). Considering the vital role of stress resistance of flavonoid compounds in plants (Shomali et al., 2022). this might be closely related to the significant biotic and abiotic stresses that plants faced during their evolution from aquatic to terrestrial habitats.

Early evolutionary studies of the CHI family member in *A. thaliana* demonstrated that non-catalytically active type III CHI are proteins involved in fatty acid binding and elucidated, in terms of three-dimensional structure, the adaptive evolutionary process by which the non-catalytically active CHI ancestor gradually evolved catalytically active through plant evolution (Ngaki et al., 2012). In this study, phylogenetic analyses of CHI family members from cross-lineage species (**Figures 1**, **2**). Firstly, the large-scale identification of different lineages of CHI genes revealed that, although type II CHI was significantly enriched in legumes, it was also found in many other plants, initially in bryophytes (*M. polymorpha*), and then in vascular plants with repeated loss and reappearance. Therefore, type II CHI is not exclusive to leguminous plants (Wang et al., 2022b). This result disproves the theory that type II CHIs evolved with the emergence of leguminous plants (Shimada et al., 2003). Bryophytes are classified into liverworts, mosses, and hornworts (Wang et al., 2022c). Previous studies reported the presence of type IV CHI in the moss *Physcomitrella patens* (Wang et al., 2022b). This study further revealed that type IV CHI is also present in liverworts, particularly in the model species *M. polymorpha*. Type IV CHI, although not catalytically active, has been extensively shown to play an important role in the flavonoid biosynthesis pathway (Jiang et al., 2015; Waki et al., 2020; Sugimoto et al., 2024). Subsequently, all the genes of *M. polymorpha* were used to construct a phylogenetic tree (**Supplementary Figure S7**), to investigate the evolutionary relationship of CHI types II and IV. From the result, we can infer that in the bryophyte lineage, CHIs type II and type IV similarly evolved from type III CHI, consistent with those in Arabidopsis. It follows that, the evolutionary relationships between different types of CHI genes were the same as those in previous studies and that both type II and type IV CHIs evolved from type III CHIs (Kaltenbach et al., 2018). Moreover, the types II and IV CHI genes of *M. polymorpha* have the matching conserved gene structure of the types I and IV CHI genes of Arabidopsis, each being distinct and characteristic (**Figure 3**; **Supplementary Table S4**). The extant bryophyte and angiosperm lineages are the same age, dating back to their last common ancestor (McDaniel, 2021). Thus, it is probable that these genes were present in the last common ancestor of both lineages and have been maintained since then during lineage-specific evolution (with modifications to type II or I from whichever was the progenitor gene in the last common ancestor).

Motifs are short sequences appearing in the gene family with certain functions or structures, which may be related to the identification of transcription factors and regulation of gene expression (Bailey et al., 2009). In this study, we identified conserved motifs (**Figure 3**; **Supplementary Table S4**) in 15 representative species, which illustrate the differences and similarities among different types of CHI family members. Notably, the conserved strategy of motif 4, motif 8, motif 10, and motif 11 during evolution seems to reveal that some of the essential structures of the active CHIs (types I and II)gene originated from ancient algae. However, the origins of motif 12, motif 13, and motif 15 are relatively recent. Motif 13 is present in land plants, motif 12 is found in the lineages of bryophytes and ferns, while motif 15 is only present in seed plants. More specifically, motif 12 first appeared concurrently in the type III CHI branch of Group 2-1 and the type IV CHI of Group 2-2. Based on the inference, that type IV evolved from type III, motif 12 may have originated from CHI type III of Early land plants and evolved to appear in CHI type IV in a short period (Kaltenbach et al., 2018; Liu et al., 2024). Motif 15 originated in basal angiosperms and was accompanied by the loss of motif 12 in bryophytes and fern plants at the same position of gene structure. Thus, this could be the result of diversification of the auxiliary functions among CHI type IV due to severe selective pressure in angiosperms (Wu et al., 2020). E.g. in *A. thaliana* CHI type IV acts as a unique enhancer in synergistic collaboration with TT5 (a CHI type I) to promote flavonoid biosynthesis, which can also promote chalcone synthase and membrane-bound terpene transferase (PT1L) activity in *H. lupulus*, as well as a general role in plants that bind to chalcone synthase to reduce byproduct formation thereby correcting CHS non-specificity (Jiang et al., 2015; Waki et al., 2020). Interestingly, the motif structures of active CHI (types I and II) and type IV CHI genes are similar, with the difference that at the same position, active CHI lacks motif12 or motif15, and this position is vacant (**Figure 3**). In conclusion, the loss of motif 12 or motif 15 and the appearance of motif 13 in land plants may have gradually provided the conditions for CHI to possess catalytic activity.

Gene family diversification is mostly driven by gene duplication, where subsequent mutations following gene duplication lead to the possibility of divergence or the emergence of neofunctionalization of genes (Lynch and Conery, 2000). In this study, we conducted a detailed analysis of the replication types that contribute to the expansion and functional diversification of the CHI-fold proteins, revealing significant variation across different taxa (**Figure 5**). In algae and bryophytes, CHI gene duplication is predominantly driven by dispersed repeats, while in ferns and gymnosperms, duplication is primarily mediated by both dispersed and proximal repeats. Basal angiosperms and monocots exhibit CHI gene expansion mainly driven by WGD, whereas tandem duplications play a dominant role in eudicots. The function of CHI genes plays an important role in plant adaptive evolution. Due to their relatively compact genomes and stable ecological niches, WGD are infrequent in algae and bryophytes, making dispersed repeats the primary mode of gene duplication (Lynch and Conery, 2000; Liu et al., 2024). Conversely, WGDs are prevalent throughout angiosperms, enabling rapid generation of extensive gene copies that facilitate adaptive evolution through natural selection (Lyons et al., 2008; Soltis et al., 2009; Renny-Byfield and Wendel, 2014; Soltis et al., 2014). The evolutionary path of true dicotyledons is notably complex and diverse, with tandem duplications serving as a strategic mechanism for swift adaptation to environmental changes, exemplified by the sub-functionalization of type II CHI, a pivotal factor in their evolutionary process (Liu et al., 2024). This observation elucidates the mechanism behind CHI gene expansion in these plants.

For those sequences with low similarity that diverge significantly over evolutionary time, structural clustering offers a better approach for representing gene conservation and variability. With the assistance of accurate prediction of protein structure by artificial intelligence, we can study the relationship between the variability of the 3D folding structure of genes and their functions (Huang et al., 2023). Exploring the relationship between gene family structure and function based on structural clustering is an important tool for probing the evolution of gene families (Esposito et al., 2021; Duan et al., 2023; Liao et al., 2023). The results of this study show that the overall structure of CHI is diverse, with this diversity mainly arising from the non-conserved N-terminal region, and most notably observed in type III CHI. Moreover, structural clustering analysis showed that all active CHIs, except the MARPO_0167s0012 gene from bryophytes, retained minimal sequences at their N-terminal. Previous evolutionary studies suggest that catalytically active CHI proteins may have evolved from non-catalytic ancestors (Kaltenbach et al., 2018). Thus, it is probable that after diverging from the last common ancestor, the N-terminus of type II CHI evolved lineage specificity. The type II CHI in the bryophyte lineage retains the ancestral N-terminal structural features, whereas, in the vascular plant lineage, it gradually evolved to become shorter. The unique folding structure, 3-layer sandwich, of the CHI family is the basis for its catalytic activity (Jez et al., 2000). We found that such structure is present in all CHI genes including the ancient algae CHI, and is almost exclusively located in the center of the sequences (**Figure 6**). It is evident that despite non-conserved terminal regions, the critical role of CHI genes in plant adaptation underlines the strong selective pressure for conserving the 3-layer sandwich fold.

This study classified chalcone isomerases (CHIs) into five types (I–V) based on phylogenetic analysis of the gene family and screening of active sites. Type I and type II CHIs are the representative functional enzymes in the flavonoid biosynthesis pathway, both catalyzing the isomerization of naringenin chalcone, resulting in the formation of (2S)-naringenin (Jez et al., 2000; Yin et al., 2019). However, type II CHI possesses an additional function—it can also catalyze the conversion of isoliquiritigenin to (2S)-liquiritigenin, which serves as a key distinguishing feature between type I and II CHIs (Shimada et al., 2003; Cheng et al., 2018). Type III CHI does not participate in flavonoid biosynthesis but has been confirmed to be involved in fatty acid synthesis (Ngaki et al., 2012). Knockout of type III CHI in Arabidopsis thaliana disrupts fatty acid metabolism, leading to reproductive defects (Ngaki et al., 2012). Notably, in flavonoid biosynthesis, type IV CHI interacts with chalcone synthase (CHS) via protein-protein interactions, enhancing THC production while reducing CTAL formation (Waki et al., 2020; Wolf-Saxon et al., 2023). Researchers typically further validate CHIL based on this biological function (Xu et al., 2022; Lewis et al., 2024; Zhang et al., 2024). Due to the lack of key active sites, type V CHI is presumed to lack chalcone isomerase activity. In summary, the biological functions of different types of CHI genes exhibit significant differences, which may provide some valuable reference for the future screening of functional CHI genes.

## Materials and methods

### Genomic data file collection and processing

Genome files (.fa) and annotation files (gff/gff3) of 259 species were obtained from JGI (https://phytozome-next.jgi.doe.gov/) (Goodstein et al., 2012), Ensemble plant (https://plants.ensembl.org/), NCBI (https://www.ncbi.nlm.nih.gov/datasets/genome/) (Bolser et al., 2016), NGDC (https://download.cncb.ac.cn/gwh/Plants/), and Published Plant Genomes databases (https://www.plabipd.de/plant_genomes_pa.ep), and species were classified according to the NCBI classification system, which was quickly completed with the help of the R package taxize (Federhen, 2012; Chamberlain and Szöcs, 2013). Differential splicings were excluded, and only the longest transcripts of each gene were retained for subsequent analyses.

### Phylogenetic analysis of 259 species

Species phylogenetic trees were reconstructed using the coalescence method by first obtaining single-copy orthologous gene ensembles for each species from the benchmarking universal single-copy ortholog BUSCO gene sets (Simão et al., 2015), with the parameters “-m prot -l viridiplantae_odb10”. Then, single-copy orthologous genes with over 80% coverage among 259 species were obtained, and multiple sequence alignments for each of these single-copy orthologous genes were performed using MAFFT v7.429 software (Katoh and Standley, 2013), with the parameters “–maxiterate 1000 -localpair”. The results of multiple sequence alignments were adjusted using trimAl v1.5 software (Capella-Gutiérrez et al., 2009), with the parameter “-gt 0.5” to limit the number of gaps in the results. The phylogenetic trees were constructed for each adjusted multiple sequence alignments result using the IQ-TREE v1.6.11 tool of the ML (maximum likelihood) method (Nguyen et al., 2015), with the parameters “-bb 1000 -m TEST -nt AUTO”. Finally, the astral-tree-5.7.8-1 software was used to construct the species phylogenetic tree based on the multi-species coalescent model, which infers species phylogenetic trees from an extensive collection of gene phylogenies (Zhang et al., 2018), with the command “java -jar astral.5.7.8.jar -i input file -o output file”. The Timetree fossil time tree serves as a reference to ascertain the root position of the phylogenetic tree (Kumar et al., 2017). The figure’s beautification was made online using the Chilpot website (https://www.chiplot.online/) (Xie et al., 2023).

### CHI-fold proteins identification

First, a protein sequence library of the longest protein coding sequence of each species was constructed using the default parameters of the makeblastdb module of the ncbi -blast -2.9.0+ software (Johnson et al., 2008). The amino acid sequences of the seven CHI-fold protein members that have been identified and characterized were used as queries for monocots (Park et al., 2021), while the identified CHI-fold protein members in *A. thaliana* downloaded from the *TAIR* database (https://www.arabidopsis.org/) were used for other species. The ncbi -blast -2.9.0+ software’s blastp module, with an e-value of 10^-5^, was employed to search all protein libraries, and the search results were used as candidate gene family members. The seed alignment files for the CHI domain (PF021431, PF16036, PF16035), obtained from pfam database (https://www.ebi.ac.uk/interpro/entry/pfam/), were used to build an HMM file using the HMMER3 (v3.3) software package (Mistry et al., 2013). Subsequently, genes lacking CHI domains were excluded based on annotations from the Conserved Domain Database (https://www.ncbi.nlm.nih.gov/cdd/) and SMART (https://smart.embl.de/) (Marchler-Bauer et al., 2010; Letunic et al., 2021). Redundant genes were then eliminated using seqkit rmdup with parameter “-s” and finally obtained CHI-fold protein members (Shen et al., 2016). The information and sequences of gene length were extracted by script bash, information of isoelectric point and molecular was searched and obtained online by ExPASy (https://web.expasy.org/compute_pi/), and subcellular localization was predicted by WoLF PSORT (https://wolfpsort.hgc.jp/) website.

### Phylogenetic analysis of CHI-fold protein members

Following the integration of all identified members into a gene set, high-throughput rapid multiple sequence alignments were performed using the MAFFT v7.429 software with default parameters, and trimAl v1.5 software was used to adjust the result. Phylogenetic trees were constructed using the IQ-TREE v1.6.11 tool with the maximum likelihood (ML) method for each adjusted result. The appropriate models were automatically selected using the parameter “-TEST”, and the Bootstrap values with parameter “-bb 1000” to assess phylogenetic tree reliability (Horowitz, 2001). The phylogenetic tree was classified by the similarity of clustering relationship among genes and sequences of chalcone isomerase-fold proteins family members, whose functions had been verified, and then further classified using amino acid residues recognizing types I and II identities (Dastmalchi and Dhaubhadel, 2015; Ni et al., 2020). All the CHIs belonging to Group 3 were classified by a custom Python script for each type of gene, following a detailed sequential alignment performed by MAFFT v7.429 software with parameter “–maxiterate 1000 -localpair”.

### Conserved motif identification and analysis of the representative species

Members of the CHI-fold proteins from 15 representative species were analyzed using the MEME Suite online platform (https://meme-suite.org/meme/tools/meme) for motif prediction, with parameters “the maximum number of motifs is set to 15”, “motif length set to 6 to 200aa”, and “the maximum width set to 50” (Bailey et al., 2009). The logo plots of the motif were obtained directly from the program running file. The phylogenetic tree is done through Chiplot (Xie et al., 2023).

### Identification and analysis of the duplication types of CHI gene families

Initially, the whole-genome duplication genes of 259 species were identified using the DupGen_finder-unique.pl program, a component of the DupGen_finder tool, with the default parameter (Qiao et al., 2019). Setting a reference species for each taxon, including *Chlamydomonas reinhardtii* for with algae, *M. polymorpha* for with bryophyte, *Selaginella moellendorffii* for with ferns, *Gnetum montanum* for with Gymnosperms, and the angiosperms (*Amborella trichopoda* for with basal angiosperm, *Acorus gramineus* for with monocotyledon, *Vitis vinifera* for with dicotyledon, and two species of the orders *Chloranthales* and *Magnoliids* were referenced for with each other). Then, statistics on the type and number of replicates to which members of the CHI-fold proteins belonged were obtained through a Python custom script. The Ka and Ks values of duplication gene pairs of the CHI-fold proteins were obtained through the calculate_Ka_Ks_pipe.pl program of the DupGen_finder tool with default parameter. The chi-square independence test was calculated using the chi2_contingency function in the scipy library of Python, with a *P* value of <0.05 being considered as a significant difference, and the significance of the result was determined by the magnitude of the ratios of the number of replicated types to the number of genome-wide genes at the genome-wide level (Wang et al., 2022a). As the taxa *Chloranthales* and *Magnoliales* are represented by only one species, which is not considered representative, they are not shown in **Figures 4B–D**.

### Analysis of structural diversity of CHI-fold proteins

The protein folding structures of the representative species in CHI-fold protein members were predicted using the default setting of AlphaFold 3 (Abramson et al., 2024). All predictions with the highest-ranking score were selected for further analyses, and proteins with low confidence were screened using a ranking score ≥ 0.7 as a threshold. Protein structure clustering was referred to the method of Caixia Gao et al, where all predicted proteins were first annotated by InterPro to ensure the presence of CHI family structural domains (IPR016087) (Jones et al., 2014; Huang et al., 2023). Structural alignment was performed using the TM-score method of the US-align tool (Zhang et al., 2022). With parameters “-a 1 -outfmt 1”. followed by the construction of a whole structural similarity matrix based on the TM values and processed using the min-max scaling. The matrix with structural similarity was clustered by the Unweighted Pair-Group Method with Arithmetic Means (UPGMA) with the Bray-Curtis dissimilarity index as the basis for clustering, and the entire clustering process was done using the vegan and phangorn packages of the R language (Ihaka and Gentleman, 1996). Structural visualization of proteins was used PyMOL v4.6.0 (DeLano, 2002).

## Conclusion

This study examined 1,738 members of chalcone isomerase (CHI) across 259 species spanning the green plant lineages, revealing an increase in the number of gene copies during the evolutionary transition from aquatic to terrestrial species. The phylogenetic distribution of CHI genes throughout the plant kingdom indicates that the origin of type II CHI can be traced back to the last common ancestor shared by bryophytes and vascular plants. We discovered a gene set in the active CHI group with 190 amino acid residues different from those of type I and II CHIs, which were categorized as type V CHIs. The study explored the primary types of gene duplication driving CHI-fold protein expansion across different groups. It was found that dispersed duplication was the main driver in early algae and bryophytes, while ferns and gymnosperms primarily exhibited dispersed and proximal duplications. Basal angiosperms and monocots showed expansions mainly driven byWGD, whereas tandem duplication was predominant in eudicots. The findings also confirmed that CHI genes in plants are under strong purifying selection. The structural diversity of CHI family proteins is mainly attributed to the non-conserved nature of the N-terminal regions, which contribute to variations in the CHI-fold structure, most notably observed in type III CHI. This study enhances the understanding of the evolutionary patterns and functional diversification of the CHI-fold proteins, providing a theoretical basis for future investigations into their biological functions and potential applications.

## Data Availability

The original contributions presented in the study are included in the article/**Supplementary Material**. Further inquiries can be directed to the corresponding authors.

## Supplementary material

Supplementary Table 1 The genomic data source of 259 species.

Supplementary Table 2 The basic statistics of CHI-fold protein family members.

Supplementary Table 3 Statistics of subcellular localization results of different types of CHI-fold protein family.

Supplementary Table 4 The motif analysis of different CHI types in representative species.

Supplementary Table 5 The statistics and significance analysis of the number of CHI WGD duplication (WGD) genes in 259 species with P-value<0.05.

Supplementary Table 6 The statistics and significance analysis of the number of CHI tandem duplication (TD) genes in 259 species with P-value<0.05.

Supplementary Table 7 The statistics and significance analysis of the number of CHI proximal duplication (PD) genes in 259 species with P-value<0.05.

Supplementary Table 8 The statistics and significance analysis of the number of CHI transposed duplication (TRD) genes in 259 species with P-value<0.05.

Supplementary Table 9 The statistics and significance analysis of the number of CHI dispersed duplication (DSD)genes in 259 species with P-value<0.05.

Supplementary Table 10 The WGD duplicate gene pairs and Ka/Ks values in the CHI-fold protein family.

Supplementary Table 11 The tandem duplicate gene pairs and Ka/Ks values in the CHI-fold protein family.
