# Supplementary material for: Evolutionary landscape of plant chalcone isomerase-fold gene families
Source: Front Plant Sci. 2025 Mar 28;16:1559547. doi: 10.3389/fpls.2025.1559547 (PMC11985768; doi:10.3389/fpls.2025.1559547)
Supplement: Supplementary Table 1 — The genomic data source of 259 species. [file DataSheet1.zip › Supplementary_information/Figure S3.pdf]

Medicago	....
EVM0003632	....
gene-Apse012G0133800	....
OkoG031217	....
Acora.07G100700.v1.1	....
CHI	....
CHI_2	....
gene-QJS04_geneDACA014652	....
gene-CEY00_Acc03848	....
gene-CEY00_Acc03850	MEVTRA..
gene-CEY00_Acc27670	....
gene-GOP47_0009997	....
EVM0010650	....
gene-LOC109764886	....
EVM0015959	....
gene-LOC18440411	....
gene-LOC18440412	....
gene-LOC18440481	....
Anaoc.0011s0700.v0.9	....
Anaoc.0016s1186.v0.9	....
gene-ACMD2_09400	....
AnM01G09141	....
AnM01G09144	....
CHI	....
gene-AQUCO_01000652v1	....
AALP_AA5G189500	M...
gene-LOC109850014	....
AVESA.00010b.r2.4AG0615430	....
AVESA.00010b.r2.4DG0758920	....
AVESA.00010b.r2.7CG0710680	....
LXT01106	....
LXT01112	....
LXT05481	....
LXT05485	....
LXT05486	....
LXT43699	....
gene-LOC120080278	....
BVRB_2g036940	....
BVRB_2g036880	....
BPChr06G09462.v1.1	....
gene-F511_19977	....
Bnt03G004203	....
gene-BRADI_1g03840v3	....
Brahy.D01G0043800.v1.1	....
Brahy.S02G0385600.v1.1	....
BjuA09g18460S	....
BjuA09g18480S	....
BjuB05g27830S	....
BjuB08g05480S	....
BnaA07g37900D	....
BnaA09g34840D	....
BnaA09g34850D	....
BnaC08g26010D	....
BnaC08g26020D	M...
Bo6g068550	....
Bo8g088480	....
Bo8g089480	M...
C.cajan_29162	....
Csa02g076590	MSLA..
Csa04g041330	M...
Csa04g041350	....
Csa06g029680	M...
Csa09g066000	M...
Csa09g066040	M...
Csa09g066060	M...
Csa11g104750	MSLA..
Csa20g023690	....
maker-Chr6-pred_gff_AUGUSTUS-gene-125.71	M...
maker-Chr6-pred_gff_AUGUSTUS-gene-220.72	....
maker-Chr6-pred_gff_GeneMark.hmm-gene-225.16	....
gene_27594	....
gene-LOC17885140	M...
gene-LOC107852750	....
gene-FH972_001554	....
Ct_T15992	....
Ct_T15993	....
gene-CIPAW_01G280400	MSIEPLTSTRNWESC...PTV....STHVAF...........QSTHVNHNYL........
gene-CFOL_v3_20734	....
gene-KP509_33G035400	....
Cecan.1G005600.V3.1	....
Cecan.2G032100.V3.1	....
Cecan.2G068600.V3.1	....
AUR62020547	....
Cs02g00014	....
Cs10g00988	....
gene-LOC101501370	....
gene-CKAN_00532200	MGPLQCVTPVQVESLVFPPAVKPLGSSKTFFLGGAGARGLEIQGKFVKFTAIGVYLEEQA
CRGY0216791	....
Cla97C09G181670	....

Medicago	....
EVM0003632	....
gene-Apse012G0133800	....
OkoG031217	....
Acora.07G100700.v1.1	....
CHI	....
CHI_2	....
gene-QJS04_geneDACA014652	....
gene-CEY00_Acc03848	....
gene-CEY00_Acc03850	....
gene-CEY00_Acc27670	....
gene-GOP47_0009997	....
EVM0010650	....
gene-LOC109764886	....
EVM0015959	....
gene-LOC18440411	....
gene-LOC18440412	....
gene-LOC18440481	....
Anaoc.0011s0700.v0.9	....
Anaoc.0016s1186.v0.9	....
gene-ACMD2_09400	....
AnM01G09141	....
AnM01G09144	....
CHI	....
gene-AQUCO_01000652v1	....
AALP_AA5G189500	....
gene-LOC109850014	....
AVESA.00010b.r2.4AG0615430	....
AVESA.00010b.r2.4DG0758920	....
AVESA.00010b.r2.7CG0710680	....
LXT01106	....
LXT01112	....
LXT05481	....
LXT05485	....
LXT05486	....
LXT43699	....
gene-LOC120080278	....
BVRB_2g036940	....
BVRB_2g036880	....
BPChr06G09462.v1.1	....
gene-F511_19977	....
Bnt03G004203	....
gene-BRADI_1g03840v3	....
Brahy.D01G0043800.v1.1	....
Brahy.S02G0385600.v1.1	....
BjuA09g18460S	....
BjuA09g18480S	....
BjuB05g27830S	....
BjuB08g05480S	....
BnaA07g37900D	....
BnaA09g34840D	....
BnaA09g34850D	....
BnaC08g26010D	....
BnaC08g26020D	....
Bo6g068550	....
Bo8g088480	....
Bo8g089480	....
C.cajan_29162	....
Csa02g076590	....
Csa04g041330	....
Csa04g041350	....
Csa06g029680	....
Csa09g066000	....
Csa09g066040	....
Csa09g066060	....
Csa11g104750	....
Csa20g023690	....
maker-Chr6-pred_gff_AUGUSTUS-gene-125.71	....
maker-Chr6-pred_gff_AUGUSTUS-gene-220.72	....
maker-Chr6-pred_gff_GeneMark.hmm-gene-225.16	....
gene_27594	....
gene-LOC17885140	....
gene-LOC107852750	....
gene-FH972_001554	....
Ct_T15992	....
Ct_T15993	....
gene-CIPAW_01G280400	.....................................FNFYLPLIATVDHVTITNQTSDP
gene-CFOL_v3_20734	....
gene-KP509_33G035400	....
Cecan.1G005600.V3.1	....
Cecan.2G032100.V3.1	....
Cecan.2G068600.V3.1	....
AUR62020547	....
Cs02g00014	....
Cs10g00988	....
gene-LOC101501370	....
gene-CKAN_00532200	AVPFLSLKWKGKTGHELMDAVDFFRDVVTGPFEKFMQVTMILPLTGQ....QYSEKVTEN
CRGY0216791	....
Cla97C09G181670	....

Medicago	....
EVM0003632	....
gene-Apse012G0133800	....
OkoG031217	....
Acora.07G100700.v1.1	....
CHI	....
CHI_2	....
gene-QJS04_geneDACA014652	....
gene-CEY00_Acc03848	....
gene-CEY00_Acc03850	....
gene-CEY00_Acc27670	....
gene-GOP47_0009997	....
EVM0010650	....
gene-LOC109764886	....
EVM0015959	....
gene-LOC18440411	....
gene-LOC18440412	....
gene-LOC18440481	....
Anaoc.0011s0700.v0.9	....
Anaoc.0016s1186.v0.9	....
gene-ACMD2_09400	....
AnM01G09141	....
AnM01G09144	....
CHI	....
gene-AQUCO_01000652v1	....
AALP_AA5G189500	....
gene-LOC109850014	....
AVESA.00010b.r2.4AG0615430	....
AVESA.00010b.r2.4DG0758920	....
AVESA.00010b.r2.7CG0710680	....
LXT01106	....
LXT01112	....
LXT05481	....
LXT05485	....
LXT05486	....
LXT43699	....
gene-LOC120080278	....
BVRB_2g036940	....
BVRB_2g036880	....
BPChr06G09462.v1.1	....
gene-F511_19977	....
Bnt03G004203	....
gene-BRADI_1g03840v3	....
Brahy.D01G0043800.v1.1	....
Brahy.S02G0385600.v1.1	....
BjuA09g18460S	....
BjuA09g18480S	....
BjuB05g27830S	....
BjuB08g05480S	....
BnaA07g37900D	....
BnaA09g34840D	....
BnaA09g34850D	....
BnaC08g26010D	....
BnaC08g26020D	....
Bo6g068550	....
Bo8g088480	....
Bo8g089480	....
C.cajan_29162	....
Csa02g076590	..................................SNLV......................
Csa04g041330	....
Csa04g041350	....
Csa06g029680	....
Csa09g066000	....
Csa09g066040	....
Csa09g066060	....
Csa11g104750	..................................TNLL......................
Csa20g023690	....
maker-Chr6-pred_gff_AUGUSTUS-gene-125.71	....
maker-Chr6-pred_gff_AUGUSTUS-gene-220.72	....
maker-Chr6-pred_gff_GeneMark.hmm-gene-225.16	....
gene_27594	....
gene-LOC17885140	....
gene-LOC107852750	....
gene-FH972_001554	....
Ct_T15992	....
Ct_T15993	....
gene-CIPAW_01G280400	CIKPWA..................CHTQNQTPP...........................
gene-CFOL_v3_20734	....
gene-KP509_33G035400	.................................MS.........................
Cecan.1G005600.V3.1	....
Cecan.2G032100.V3.1	....
Cecan.2G068600.V3.1	....
AUR62020547	....
Cs02g00014	....
Cs10g00988	....
gene-LOC101501370	....
gene-CKAN_00532200	CVAYWKAVGLYTEAEAIAVEKFKQVFKEETFPPGSTIFFTLNPSGSLTIGFSKNSSIPEQ
CRGY0216791	....
Cla97C09G181670	....

**Medicago**	....
**EVM0003632**	....
**gene-Apse012G0133800**	....
**OkoG031217**	....
**Acora.07G100700.v1.1**	....
**CHI**	....
**CHI_2**	....
**gene-QJS04_geneDACA014652**	....
**gene-CEY00_Acc03848**	....
**gene-CEY00_Acc03850**	........................SFAATAMLAFLFAVLF....................
**gene-CEY00_Acc27670**	....
**gene-GOP47_0009997**	....
**EVM0010650**	....
**gene-LOC109764886**	....
**EVM0015959**	....
**gene-LOC18440411**	....
**gene-LOC18440412**	....
**gene-LOC18440481**	....
**Anaoc.0011s0700.v0.9**	....
**Anaoc.0016s1186.v0.9**	....
**gene-ACMD2_09400**	....
**AnM01G09141**	....
**AnM01G09144**	....
**CHI**	....
**gene-AQUCO_01000652v1**	....
**AALP_AA5G189500**	....
**gene-LOC109850014**	....
**AVESA.00010b.r2.4AG0615430**	....
**AVESA.00010b.r2.4DG0758920**	....
**AVESA.00010b.r2.7CG0710680**	....
**LXT01106**	....
**LXT01112**	....
**LXT05481**	....
**LXT05485**	....
**LXT05486**	....
**LXT43699**	....
**gene-LOC120080278**	....
**BVRB_2g036940**	....
**BVRB_2g036880**	....
**BPChr06G09462.v1.1**	....
**gene-F511_19977**	....
**Bnt03G004203**	....
**gene-BRADI_1g03840v3**	....
**Brahy.D01G0043800.v1.1**	....
**Brahy.S02G0385600.v1.1**	....
**BjuA09g18460S**	....
**BjuA09g18480S**	....
**BjuB05g27830S**	....
**BjuB08g05480S**	....
**BnaA07g37900D**	....
**BnaA09g34840D**	....
**BnaA09g34850D**	....
**BnaC08g26010D**	....
**BnaC08g26020D**	....
**Bo6g068550**	....
**Bo8g088480**	....
**Bo8g089480**	....
**C.cajan_29162**	....
**Csa02g076590**	....
**Csa04g041330**	....
**Csa04g041350**	....
**Csa06g029680**	....
**Csa09g066000**	....
**Csa09g066040**	....
**Csa09g066060**	....
**Csa11g104750**	....
**Csa20g023690**	....
**maker-Chr6-pred_gff_AUGUSTUS-gene-125.71**	....
**maker-Chr6-pred_gff_AUGUSTUS-gene-220.72**	....
**maker-Chr6-pred_gff_GeneMark.hmm-gene-225.16**	....
**gene_27594**	....
**gene-LOC17885140**	....
**gene-LOC107852750**	....
**gene-FH972_001554**	....
**Ct_T15992**	....
**Ct_T15993**	....
**gene-CIPAW_01G280400**	....
**gene-CFOL_v3_20734**	....
**gene-KP509_33G035400**	....
**Cecan.1G005600.V3.1**	....
**Cecan.2G032100.V3.1**	....
**Cecan.2G068600.V3.1**	....
**AUR62020547**	....
**Cs02g00014**	....
**Cs10g00988**	....
**gene-LOC101501370**	....
**gene-CKAN_00532200**	AAGVIENKQMSEAVLESMIGKHGVSPEAKQSLATRVSQLLKEFDVETGRVKVEGEEGEKL
**CRGY0216791**	....
**Cla97C09G181670**	....

1

**Medicago**	......................................M.....................
**EVM0003632**	...................................MG.ESA...................
**gene-Apse012G0133800**	...................................MH.P.....................
**OkoG031217**	....
**Acora.07G100700.v1.1**	...................................MAQS.....................
**CHI**	...................................MNDK.....................
**CHI_2**	....
**gene-QJS04_geneDACA014652**	...................................MAQS.....................
**gene-CEY00_Acc03848**	...................................MS.......................
**gene-CEY00_Acc03850**	...............AAATAHSAAPSSDRE.KTK.SS.L.....................
**gene-CEY00_Acc27670**	...................................MS.P.....................
**gene-GOP47_0009997**	...................................MT..............DAV.....E
**EVM0010650**	....
**gene-LOC109764886**	....
**EVM0015959**	...................................MA.L.....................
**gene-LOC18440411**	...................................MG.......................
**gene-LOC18440412**	....
**gene-LOC18440481**	...................................MA.T.....................
**Anaoc.0011s0700.v0.9**	...................................MK.P.....................
**Anaoc.0016s1186.v0.9**	...................................MN.P.....................
**gene-ACMD2_09400**	...................................MA.EA....................
**AnM01G09141**	....
**AnM01G09144**	....
**CHI**	....
**gene-AQUCO_01000652v1**	...................................MT.SSDL..................
**AALP_AA5G189500**	...........................STS.STS.PS.P.....................
**gene-LOC109850014**	...................................MA.......................
**AVESA.00010b.r2.4AG0615430**	....
**AVESA.00010b.r2.4DG0758920**	....
**AVESA.00010b.r2.7CG0710680**	....
**LXT01106**	...................................MA.Y.....................
**LXT01112**	...................................MA.Y.....................
**LXT05481**	...................................MS.G.....................
**LXT05485**	...................................MA.Y.....................
**LXT05486**	...................................MA.Y.....................
**LXT43699**	...................................MA.Y.....................
**gene-LOC120080278**	...................................MA.L.....................
**BVRB_2g036940**	...................................MA.T.....................
**BVRB_2g036880**	...................................MA.......................
**BPChr06G09462.v1.1**	...................................MV.S.....................
**gene-F511_19977**	...................................MS.A.....................
**Bnt03G004203**	...................................MA.S.....................
**gene-BRADI_1g03840v3**	....
**Brahy.D01G0043800.v1.1**	....
**Brahy.S02G0385600.v1.1**	....
**BjuA09g18460S**	...........................MFS.SGI.QT.P.....................
**BjuA09g18480S**	...........................MSS.SNC.PS.P.....................
**BjuB05g27830S**	...........................MFS.SSS.RS.P.....................
**BjuB08g05480S**	....
**BnaA07g37900D**	...........................MSS.SHC.PT.P.....................
**BnaA09g34840D**	...........................MSS.SNC.PS.P.....................
**BnaA09g34850D**	...........................MFS.SGI.QT.P.....................
**BnaC08g26010D**	...........................MSS.SNC.PS.P.....................
**BnaC08g26020D**	.......................FDQIIIS.SGV.QS.P.....................
**Bo6g068550**	...........................MSS.SHC.PT.P.....................
**Bo8g088480**	...........................MSS.SNC.PS.P.....................
**Bo8g089480**	.......................FDQIIIS.SGV.QS.P.....................
**C.cajan_29162**	...................................MATPPSSVTVVEVLHNVALPENLTF
**Csa02g076590**	.......................TPNLSWS.ASSPLP.......................
**Csa04g041330**	.........................SSSSV.VVS.PS.P.....................
**Csa04g041350**	....
**Csa06g029680**	.........................SSSSV.VVS.PS.P.....................
**Csa09g066000**	.........................SSSSV.VVS.PS.P.....................
**Csa09g066040**	.........................SSSSV.VVS.PS.P.....................
**Csa09g066060**	.........................SSSSV.VVY.PS.P.....................
**Csa11g104750**	.......................SPNLSWS.SSSPLP.......................
**Csa20g023690**	...........................MSL.STS.LL.P.....................
**maker-Chr6-pred_gff_AUGUSTUS-gene-125.71**	...............................SPS.QS.P.....................
**maker-Chr6-pred_gff_AUGUSTUS-gene-220.72**	......................................M.....................
**maker-Chr6-pred_gff_GeneMark.hmm-gene-225.16**	....
**gene_27594**	...................................MNLS.....................
**gene-LOC17885140**	.........................SSSSVDVVS.PP.P.....................
**gene-LOC107852750**	...................................MA.......................
**gene-FH972_001554**	...................................MV.S.....................
**Ct_T15992**	...................................MA.P.....................
**Ct_T15993**	...................................MA.A.....................
**gene-CIPAW_01G280400**	.......................RTVTTNI.LAK.MV.P.....................
**gene-CFOL_v3_20734**	...................................MG.P.....................
**gene-KP509_33G035400**	...............................AFSTMT..............NAC.....G
**Cecan.1G005600.V3.1**	...................................MA.Y.....................
**Cecan.2G032100.V3.1**	...................................MA.Y.....................
**Cecan.2G068600.V3.1**	....
**AUR62020547**	...................................MA.T.....................
**Cs02g00014**	...................................ML.EQ....................
**Cs10g00988**	...................................MG.P.....................
**gene-LOC101501370**	...................................MA.......................
**gene-CKAN_00532200**	KHPSLSLSAVPVNRSTTDQFFFSSKASIAS.DRE.MG.P.....................
**CRGY0216791**	...................................MA.P.....................
**Cla97C09G181670**	...................................MA.P.....................

```
                                                                          10                                20
                                                                           .                                 .
Medicago                                              ....AAS.IT......AITV.........ENLEYP.....A......VV.TSP.V....TG
EVM0003632                                            ...AVVA.TT......ALEI.........EGINFP.....A......TI.TPP.E....SS
gene-Apse012G0133800                                  ....SPP.VT......EVQV.........ENVTFS.....P......AV.KPP.G....ST
OkoG031217                                            .....MA.VS......EVEV.........DGVVFP.....P......LA.RPP.G....SG
Acora.07G100700.v1.1                                  ....LSL.VS......DLTV.........EDVHFP.....S......SV.VPP.G....SS
CHI                                                   ....PRT.YS......D............ED.HW...........................
CHI_2                                                 .............................................................
gene-QJS04_geneDACA014652                             ....LSL.VS......ELTV.........EDVHFP.....S......SV.VPP.G....SS
gene-CEY00_Acc03848                                   ....PPL.VT......GVQI.........ETVVFP.....P......TA.KPT.G....TD
gene-CEY00_Acc03850                                   ....LPL.VT......PIQV.........ESHVFP.....P......TV.KPP.G....MA
gene-CEY00_Acc27670                                   ....PTP.VT......GVQI.........ETVVFP.....S......TA.KPP.G....TA
gene-GOP47_0009997                                    N..KQVE.FG......ALEV.........EGIKFA.....P......SV.AAL.G....ST
EVM0010650                                            .....MA.VS......ELEV.........DGVVFP.....P......LA.RPP.G....SE
gene-LOC109764886                                     .....MA.VS......ELEV.........DGVVFP.....P......LA.RPP.G....SE
EVM0015959                                            ....ERV.VS......ELQV.........EDYVFP.....S......TV.KPL.G....ST
gene-LOC18440411                                      ....IVG.LP......GMEV.........EGVVFS.....A......TV.KPP.L....SS
gene-LOC18440412                                      ...............MEV.........EGVVFS.....T......TV.RPP.L....SS
gene-LOC18440481                                      ....VSP.VS......ELRV.........EGYSFP.....P......AV.KPP.G....SS
Anaoc.0011s0700.v0.9                                  ....SAS.LP......DVQV.........EHFTFA.....S......AV.KPP.G....SP
Anaoc.0016s1186.v0.9                                  ....SPS.IT......DGQV.........EHFTFS.....S......TI.KPP.A....SS
gene-ACMD2_09400                                      ...PIVV.VP......KLEI.........EGITFP.....A......VT.SPP.R....SS
AnM01G09141                                           .............................................................
AnM01G09144                                           ....MPA.IT......QIQV.........ESHVFP.....P......EV.KPP.G....SD
CHI                                                   ...............MEV.........AGIEFP.....E......AV.TAP.G....SS
gene-AQUCO_01000652v1                                 .SSSSSS.LA..RLNMELKV.........EDYTFC.....P......TLSTAP.G....SA
AALP_AA5G189500                                       ....LPS.VT......KLQV.........ESVTFP.....P......SV.NSP.A....SS
gene-LOC109850014                                     ....EAA.AT......KLEV.........EGVSFP.....A......LV.TPP.G....SS
AVESA.00010b.r2.4AG0615430                            .....MA.SS......ELEV.........DGVVFP.....P......LA.HPP.G....SA
AVESA.00010b.r2.4DG0758920                            .....MA.SS......ELEV.........DGVVFP.....P......LA.HPP.G....SA
AVESA.00010b.r2.7CG0710680                            .....MA.NS......ELEV.........DGVVFP.....P......LA.HPP.G....SA
LXT01106                                              ....TTC.LT......AVQV.........ENFEFP.....P......TA.KAP.G....ST
LXT01112                                              ....TSC.LT......AVQV.........ENFEFP.....P......TV.KAP.G....ST
LXT05481                                              ....LES.RE......RDHA.........GNILFNNLVQWP......TH.QPS.P....PF
LXT05485                                              ....TTC.LT......AVQV.........ENFEFP.....P......TA.KAP.G....ST
LXT05486                                              ....TSC.LT......AVQV.........ENFEFP.....P......TV.KAP.G....ST
LXT43699                                              ....ASS.LT......AVQV.........DNVEFL.....P......AV.KAP.G....SA
gene-LOC120080278                                     ....VAD.LG......GLQV.........ENVKFP.....A......EI.KPP.A....SA
BVRB_2g036940                                         ....STL.VS......EMKV.........EDYAFP.....S......TV.KAP.G....SN
BVRB_2g036880                                         ......P.VM......ELCV.........EDYVFP.....P......MV.KAP.G....SD
BPChr06G09462.v1.1                                    ....PPS.LT......GVQV.........ENVEFP.....P......TA.KPP.G....ST
gene-F511_19977                                       ....PLS.VT......KLQV.........ESCEFP.....P......SV.KPP.G....ST
Bnt03G004203                                          ...APSS.LA......AIQV.........ELATFP.....P......AV.KPP.G....SK
gene-BRADI_1g03840v3                                  .....MA.VS......ELEV.........DGVVFP.....P......LE.RPP.G....SA
Brahy.D01G0043800.v1.1                                .....MA.VS......ELEV.........DGVVFP.....P......LE.RPP.G....SA
Brahy.S02G0385600.v1.1                                .....MA.LS......ELEV.........DGVVFP.....P......LA.RPP.G....SA
BjuA09g18460S                                         ....LPS.VT......NLQV.........DSVNFP.....Q......SV.ISP.A....SS
BjuA09g18480S                                         ....LPT.VT......KLQV.........DSVTFP.....P......SV.ISP.A....SS
BjuB05g27830S                                         ....LPS.VT......NLQV.........DSVTFP.....P......SV.ISP.T....SS
BjuB08g05480S                                         .............................................................
BnaA07g37900D                                         ....LPS.VT......KLQV.........DSVTFP.....P......SV.ISP.A....SS
BnaA09g34840D                                         ....LPT.VT......KLQV.........DSVTFP.....P......SV.ISP.A....SS
BnaA09g34850D                                         ....LPS.VT......NLQV.........DSVNFP.....P......SV.ISP.A....SS
BnaC08g26010D                                         ....LPT.VT......KLQV.........DSVTFP.....P......SV.ISP.A....SS
BnaC08g26020D                                         ....LPS.VT......NLHV.........DSVNFP.....P......SV.ISP.A....SS
Bo6g068550                                            ....LPS.VT......KLQV.........DSVTFP.....P......SV.ISP.A....SS
Bo8g088480                                            ....LPT.VT......KLQV.........DSVTFP.....S......SV.ISP.A....SS
Bo8g089480                                            ....LPS.VT......NLQV.........DSVNFP.....P......SV.ISP.A....SS
C.cajan_29162                                         P..AAES.VT......AVHV.........ENVPFP.....P......AV.KPP.S....SA
Csa02g076590                                          ....LPS.VT......PLHV.........DAFTFP.....P......AV.ESP.A....SH
Csa04g041330                                          ....LPT.VA......ELHV.........DSVTFA.....P......SV.RSP.A....SS
Csa04g041350                                          ......M.YK......KRET.........KQKRVT.....P......RA.YTS.N....TR
Csa06g029680                                          ....LPT.VA......KLHV.........DSVTFA.....P......SV.KSP.A....SS
Csa09g066000                                          ....LPT.VA......ELHV.........DSVTFA.....P......SV.KSP.A....SS
Csa09g066040                                          ....LPT.VA......KLHV.........DSVTFA.....P......SV.KSP.A....SS
Csa09g066060                                          ....LPT.VA......ELHV.........DSVTFA.....P......SV.RSP.A....SS
Csa11g104750                                          ....LPS.VT......PLHV.........DVFTFP.....P......AV.ESP.A....SH
Csa20g023690                                          ....LPS.VT......QLQI.........DSVTFA.....P......AI.SSP.A....SH
maker-Chr6-pred_gff_AUGUSTUS-gene-125.71              ....SPS.VT......QVQI.........ESHVFP.....P......TV.KPP.G....TS
maker-Chr6-pred_gff_AUGUSTUS-gene-220.72              ....SPS.VT......QVQI.........ESHVFP.....P......TV.KPP.S....TV
maker-Chr6-pred_gff_GeneMark.hmm-gene-225.16          .............................................................
gene_27594                                            ....PLT.SS......GIQI.........EAAKFP.....P......AV.TPP.G....LD
gene-LOC17885140                                      ....LPT.VA......KLHV.........DSVTFA.....P......SV.KSP.A....SS
gene-LOC107852750                                     ......S.IT......KVQV.........DNNVFP.....PS.....VV.KPP.G....SN
gene-FH972_001554                                     ....PLS.ST......GVQV.........ENLEFP.....P......AA.KPP.G....SA
Ct_T15992                                             ...PPST.TT......SLQV.........ESVVFP.....P......SV.KAP.G....TA
Ct_T15993                                             ...LTPS.ST......SIQV.........ESIVFP.....P....................
gene-CIPAW_01G280400                                  ....APS.LA......GVHV.........DNVAFQ.....P......TT.KVP.G....SD
gene-CFOL_v3_20734                                    ....SPS.VT......ELQV.........ENVTFP.....P......SV.KPP.G....ST
gene-KP509_33G035400                                  C..KQLD.FG......SLDI.........EGIPFA.....A......TA.TTL.G....ST
Cecan.1G005600.V3.1                                   ....APS.LT......AVQV.........DNIEFL.....P......AV.KAP.G....ST
Cecan.2G032100.V3.1                                   ....APT.LT......AVQV.........ENVEFP.....P......AV.KAP.G....ST
Cecan.2G068600.V3.1                                   .............................................................
AUR62020547                                           ....STP.VT......EVKV.........EDYVFP.....P......TV.KAP.G....SD
Cs02g00014                                            ......TLLSNFWSN.NVKVNLLVVAIKKGLEIP..LAAPEALILATKKKSIKGRLKISG
Cs10g00988                                            ....LPS.VS......SLQV.........AGFIFS.....P......AV.QPP.G....SS
gene-LOC101501370                                     ....SLP.VT......ALEI.........ENIVFP.....P......TV.KPP.G....SC
gene-CKAN_00532200                                    ....LQC.VT......PVQV.........ESLVFP.....P......AV.KPL.G....SS
CRGY0216791                                           ........IS......EVQV.........ENVVFP.....P......SV.KPP.G....SS
Cla97C09G181670                                       ....VTD.LG......GLQV.........ENVKFP.....A......KI.KPP.A....SA
```

```
                                                            30        40        50
Medicago                                            ....KS..YFLGG.AGERGL.TIEGNFIKFTAI...........................
EVM0003632                                          ....KR..FFLGG.AGKRGL.VVEGKFKTFTAI...........................
gene-Apse012G0133800                                ....KP..HFLGG.AGERGL.EIQGKFIKFTAI...........................
OkoG031217                                          ....HA..HFLAG.AGVRGM.EIGGNFIKFTAI...........................
Acora.07G100700.v1.1                                ....NS..LFLGG.AGVREL.EIDGRFVKFTSI...........................
CHI                                                 ................VRGL.EIDGRFVKFTSI...........................
CHI_2                                               ..........MAP.SGVRGL.EIDGRFVKFTSI...........................
gene-QJS04_geneDACA014652                           ....HS..LFLGG.AGVRGL.EIDGRFVKFTSI...........................
gene-CEY00_Acc03848                                 ....KP..FFLGG.AGERGL.EIEGRFIKFTAI...........................
gene-CEY00_Acc03850                                 ....KS..FFLGG.AGVRGL.EIEGKFIKFTAT...........................
gene-CEY00_Acc27670                                 ....KP..FFLGG.AGERGL.EIEGRFIKFTAI...........................
gene-GOP47_0009997                                  ....KQ..LVLGG.AGFRGL.EISGNLVKFTAI...........................
EVM0010650                                          ....HA..HFLAG.AGVRGM.EIGGNFIKFTAI...........................
gene-LOC109764886                                   ....HA..HFLAG.AGVRGM.EIGGNFIKFTAI...........................
EVM0015959                                          ....NT..LFLGG.AGVRGL.EIQDKFIKFTAI...........................
gene-LOC18440411                                    SSSKKD..LALCG.AGVRGV.EKEGVFVKFTCT...........................
gene-LOC18440412                                    SSPKKD..LALGG.AGVRGV.ERGGVFVKSTCM...........................
gene-LOC18440481                                    ....QS..LFLAA.AGVRGL.EIQGKFIKFTAI...........................
Anaoc.0011s0700.v0.9                                ....KT..HFLAG.AGERGL.EIQGKFVKFTTI...........................
Anaoc.0016s1186.v0.9                                ....QT..HFLAG.AGVRGL.EIHGKFVKFTAI...........................
gene-ACMD2_09400                                    ....SSSPLFLAG.AGARGL.EIGGRFVNFTAI...........................
AnM01G09141                                         ............................................................
AnM01G09144                                         ....KT..FFLGG.AGVRGL.EIQGKFIKFTAI...........................
CHI                                                 ....KN..LFLGG.AGVRGL.EIGGNFIVVTAI...........................
gene-AQUCO_01000652v1                               ....NS..FFLGG.AGVRGL.QIQDKFIKFTAI...........................
AALP_AA5G189500                                     ....NP..LFLGG.AGVRGL.DIHGKFIVFTVI...........................
gene-LOC109850014                                   ....KT..HLLGG.AGVRGL.EIGGKFVSFTAI...........................
AVESA.00010b.r2.4AG0615430                          ....HA..HFLAG.AGVRGM.EIGGNFIKFTAI...........................
AVESA.00010b.r2.4DG0758920                          ....HA..HFLAG.AGVRGM.EIGGNFIKFTAI...........................
AVESA.00010b.r2.7CG0710680                          ....HA..HFLAG.AGVRGM.EIGGNFIKFTAI...........................
LXT01106                                            ....KS..VFLGG.AGVRGL.YEDGKFVKYTAV...........................
LXT01112                                            ....RS..FFLGG.AGVRGL.YEDGKFVKYTAI...........................
LXT05481                                            ....RW..RTFSG.AGVRGL.HEDGKFVKYTAI...........................
LXT05485                                            ....KS..IFLGG.AGVRGL.YEDGKFVKYTAV...........................
LXT05486                                            ....RS..FFLGG.AGVRGL.YEDGKFVKYTAI...........................
LXT43699                                            ....KN..FFLGG.AGVRGL.QIQGNFVKFTAI...........................
gene-LOC120080278                                   ....KT..LFLGG.AGVRAL.EIGGNTVKFTAI...........................
BVRB_2g036940                                       ....KT..FFLGG.AGVRGL.EIQGKFVKFTAI...........................
BVRB_2g036880                                       ....KN..FFLGG.AGERGRSDLEGKYVKYTAI...........................
BPChr06G09462.v1.1                                  ....KT..LFLGG.AGARGL.EIQGKFVKFTAI...........................
gene-F511_19977                                     ....KT..FFLGG.AGVRGL.EIEGKFIKFTAI...........................
Bnt03G004203                                        ....KS..LFLAG.AGARGL.EIQGKFVKFTAI...........................
gene-BRADI_1g03840v3                                ....HA..HFLAG.AGVRGM.DIGGNFIKFTAI...........................
Brahy.D01G0043800.v1.1                              ....HA..HFLAG.AGVRGM.DIGGNFIKFTAI...........................
Brahy.S02G0385600.v1.1                              ....HA..HFLAG.AGVRGM.DIGGKFIKFTAI...........................
BjuA09g18460S                                       ....NR..LFLAG.AGVQGL.DIQGEFVISTVI...........................
BjuA09g18480S                                       ....NP..LFLGG.AGVRGL.DIQGKFVIFTVI...........................
BjuB05g27830S                                       ....NP..LFLAG.TGVQGL.NIQGEFVISTVI...........................
BjuB08g05480S                                       ................MRGL.NIQGEFVISTLI...........................
BnaA07g37900D                                       ....NP..LFLGG.AGVRGL.DIHGKFVIFTVI...........................
BnaA09g34840D                                       ....NP..LFLGGAAGVRGL.DIQGKFVIFTVI...........................
BnaA09g34850D                                       ....NR..LFLAG.AGVQGL.DIQGEFVISTVI...........................
BnaC08g26010D                                       ....NP..LFLGG.AGVRGL.DIQGKFVIFTVI...........................
BnaC08g26020D                                       ....NR..LFLAG.AGVQGL.DIQGEFVISTVI...........................
Bo6g068550                                          ....NP..LFLGG.AGVRGL.DIHGKFVIFTVI...........................
Bo8g088480                                          ....NP..LFLGG.AGVRGL.DIQGKFVIFTVI...........................
Bo8g089480                                          ....NR..LFLAG.AGVQGL.DIQGEFVISTVI...........................
C.cajan_29162                                       ....NT..HFLGG.AGVRGL.QIQDNFVKFTAI...........................
Csa02g076590                                        ....KK..LFLGG.AGIRWF.AIEGKFVIVTVI...........................
Csa04g041330                                        ....NP..LFLGG.AGARGL.KIQEEFIIFSVV...........................
Csa04g041350                                        ....NR..NY.......................C..........................
Csa06g029680                                        ....NP..LFLGG.AGVRGL.DIQGKFIIFTVIGVYLDANAVPSLSLKWKGKTAEELTES
Csa09g066000                                        ....NP..LFLGG.AGV......QGKFTTYTVV...........................
Csa09g066040                                        ....NP..LFLGG.AGVRGL.EIQGKFIIFTVI...........................
Csa09g066060                                        ....NP..LFLGG.AGVRGL.EDRGEVIILSVV...........................
Csa11g104750                                        ....KK..LFLGG.AGIRWF.DIEGKFVIVTVI...........................
Csa20g023690                                        ....NT..LFLGG.AGVRGL.DMHGKFVIFTVT...........................
maker-Chr6-pred_gff_AUGUSTUS-gene-125.71            ....KP..FFLGG.AGERGL.EIQGKFIKFTAI...........................
maker-Chr6-pred_gff_AUGUSTUS-gene-220.72            ....KS..FFLGG.AGERGL.EIKGKFIKFTAI...........................
maker-Chr6-pred_gff_GeneMark.hmm-gene-225.16        ............................................................
gene_27594                                          ....KT..LFLSG.AGVRAL.EIQGNLVKFTSI...........................
gene-LOC17885140                                    ....NP..LFLGG.AGVRGL.EIQGKFVIFTLI...........................
gene-LOC107852750                                   ....TT..LFLAG.AGIRGV.DIEGKFVKCTAI...........................
gene-FH972_001554                                   ....NT..LFLGG.AGARGL.EIQGKLVKFTAI...........................
Ct_T15992                                           ....TT..LFLAG.AGVRGM.EIEGKFVKLTGI...........................
Ct_T15993                                           ............................SFTGI...........................
gene-CIPAW_01G280400                                ....KT..LFLGG.AGVRGL.EIQGNFVKFTAI...........................
gene-CFOL_v3_20734                                  ....KT..LFLGG.AGERGL.EIQGKFIKFTAI...........................
gene-KP509_33G035400                                ....TE..LILGG.AGFRGL.EIQGKLIKFTAI...........................
Cecan.1G005600.V3.1                                 ....KS..FFLGG.AGVRGL.QIQENFVKFTAI...........................
Cecan.2G032100.V3.1                                 ....KN..FFLGG.AGVRGL.QIQENFVKFTAI...........................
Cecan.2G068600.V3.1                                 ............................................................
AUR62020547                                         ....KS..FFLGG.AGVRGL.EIQGNFVKFTAI...........................
Cs02g00014                                          ....KS...FVAS.RDMRSR.SCEGSLLECQKL...........................
Cs10g00988                                          ....KT..HFLGG.AGVRGL.EIQGKFIKFTAI...........................
gene-LOC101501370                                   ....DT..LFLGG.AGVRGL.QIHDKFVKFTAI...........................
gene-CKAN_00532200                                  ....KT..FFLGG.AGARGL.EIQGKFVKFTAI...........................
CRGY0216791                                         ....NE..LFLGG.AGVRGL.DIQGKFVKFTAI...........................
Cla97C09G181670                                     ....NT..LSLGG.AGVRAL.EIGGNTVKFTAI...........................
```

```
                                                                                           60
                                                                                           .
Medicago                                      ................................GVYLED.IAVASLAA.KWK........G
EVM0003632                                    ................................GVYLEE.NAVAELAG.KWK........G
gene-Apse012G0133800                          ................................GVYLED.DAVSSLAV.KWK........G
OkoG031217                                    ................................GVYLQADAAVSALAK.KWA........G
Acora.07G100700.v1.1                          ................................GVYLEE.SAIQSLAA.TWK........G
CHI                                           ................................GVYLEE.SAIQSLAA.TWK........G
CHI_2                                         ................................GVYLEE.SAIQSLAA.TWK........G
gene-QJS04_geneDACA014652                     ................................GVYLEE.SAIESLAA.TWK........G
gene-CEY00_Acc03848                           ................................GVYLEE.SAVPSLAV.KWK........G
gene-CEY00_Acc03850                           ................................GVYLEE.KAVPSLAI.KWK........G
gene-CEY00_Acc27670                           ................................GVYLEE.SAVPSLAV.KWK........G
gene-GOP47_0009997                            ................................GIYVDE.AIIPHLSP.KLH........G
EVM0010650                                    ................................GVYLQADAAVSALAA.KWA........G
gene-LOC109764886                             ................................GVYLQADAAVSALAA.KWA........G
EVM0015959                                    ................................GVYLEE.KAIPFLAV.KWK........G
gene-LOC18440411                              ................................ALYMEE.AAVYDLEP.KWK........G
gene-LOC18440412                              ................................ALYMEE.AAVYDLEP.KWK........G
gene-LOC18440481                              ................................GVYLET.DAIPHLAL.KWK........G
Anaoc.0011s0700.v0.9                          ................................GVYVED.SAVSSLAG.KWK........G
Anaoc.0016s1186.v0.9                          ................................GVYVEC.SAVTSVAG.KWT........G
gene-ACMD2_09400                              ................................GVYLEE.GALRSLAG.KWG........G
AnM01G09141                                   ............................................................
AnM01G09144                                   ................................AVYIAD.DAVSSLAV.KWK........G
CHI                                           ................................GVYLEAGAALKVLAE.KWK........G
gene-AQUCO_01000652v1                         ................................GVYLEEQKAISSLAV.KWN........G
AALP_AA5G189500                               ................................GVYLDA.IAVPSLSV.KWK........G
gene-LOC109850014                             ................................GVYLES.GAVSVLAE.KWK........G
AVESA.00010b.r2.4AG0615430                    ................................GVYLQADAAVSALAA.KWA........G
AVESA.00010b.r2.4DG0758920                    ................................GVYLQADAAVSALAA.KWA........G
AVESA.00010b.r2.7CG0710680                    ................................GVYLQADAAVSALAA.KWA........G
LXT01106                                      ................................GVYLED.IAVPSLAG.KWK........S
LXT01112                                      ................................GVYLED.NAVPSLAG.KWK........S
LXT05481                                      ................................GVYLED.NAVPLLAD.KWK........S
LXT05485                                      ................................GVYLED.IAVPSLAG.KWK........S
LXT05486                                      ................................GVYLED.NAVPSLAG.KWK........S
LXT43699                                      ................................GVYLED.NAVPSLAG.KWK........S
gene-LOC120080278                             ................................GVYLEE.KAVPLLAS.KWS........G
BVRB_2g036940                                 ................................GVYLEE.NGIAALAS.KWK........G
BVRB_2g036880                                 ................................GVYLEEDKATSSLAA.KWK........G
BPChr06G09462.v1.1                            ................................GVYLED.TAVPSLAV.KWK........G
gene-F511_19977                               ................................AVYLED.TAVASLAE.KWK........G
Bnt03G004203                                  ................................GVYLED.DAVKCLAA.KWK........G
gene-BRADI_1g03840v3                          ................................GVYLQADAAVSSLAA.KWA........A
Brahy.D01G0043800.v1.1                        ................................GVYLQADAAVSSLAA.KWA........A
Brahy.S02G0385600.v1.1                        ................................GVYLQADAAASSLAA.RWA........G
BjuA09g18460S                                 ................................GVYLDP.NALEGLPF............C
BjuA09g18480S                                 ................................GVYLDP.VSVTSLSV.KWK........G
BjuB05g27830S                                 ................................GVYLYP.NAVRRLTFGGWR........C
BjuB08g05480S                                 ................................GVYLDP.NAIRRLTFGGWR........C
BnaA07g37900D                                 ................................GVYLDA.VAVPSLSV.KWK........G
BnaA09g34840D                                 ................................GVYLDP.VSVTSLSV.KWK........G
BnaA09g34850D                                 ................................GVYLDP.NALEGLPF............C
BnaC08g26010D                                 ................................GVYLDP.VSVPSLSV.KWK........G
BnaC08g26020D                                 ................................GVYLDP.NAVGSLTSFGWG........C
Bo6g068550                                    ................................GVYLDA.VAVPSLSV.KWK........G
Bo8g088480                                    ................................GVYLDP.VSVPSLSV.KWK........G
Bo8g089480                                    ................................GVYLDP.NAVGSLTSFGWG........C
C.cajan_29162                                 ................................GVYLQH.DALSFLAD.KWT........A
Csa02g076590                                  ................................GVYLEA.MAVPSLSV.KWK........A
Csa04g041330                                  ................................GVYLDA.NAVPSLAL.KWK........G
Csa04g041350                                  ...............................Y............................
Csa06g029680                                  VPFFREIVTGDYETSVNWTAILGESNGEXTVVGVYLDA.NAVPSLSL.KWK........G
Csa09g066000                                  ................................GVYLDA.NAIPSLSL.KWK........G
Csa09g066040                                  ................................GVYLDA.NAVPSLSL.KWK........G
Csa09g066060                                  ................................GVYLDA.NAVPSLSL.KWK........G
Csa11g104750                                  ................................GVYLEA.MAGPSVSV.KWK........G
Csa20g023690                                  ................................GLYLEA.NAVQSLSV.KWK........G
maker-Chr6-pred_gff_AUGUSTUS-gene-125.71      ................................GVYLED.SAIPSLAV.KWK........G
maker-Chr6-pred_gff_AUGUSTUS-gene-220.72      ................................GVYLED.SAIPSLAV.KWK........G
maker-Chr6-pred_gff_GeneMark.hmm-gene-225.16  ............................................................
gene_27594                                    ................................GVYLED.SAVTWLSG.KWK........G
gene-LOC17885140                              ................................GVYLDA.KAVPSLSL.KWK........G
gene-LOC107852750                             ................................GVYMEE.SAVPFLAA.KWK........G
gene-FH972_001554                             ................................GVYLED.TAVPSLSV.KWK........G
Ct_T15992                                     ................................GLYLED.KAIPSLAV.KWK........G
Ct_T15993                                     ................................GVYLED.KAIPSLAG.KWK........G
gene-CIPAW_01G280400                          ................................GVYLED.TAVPSLSI.KWS........G
gene-CFOL_v3_20734                            ................................GVYLEG.DAVASLAV.KWK........G
gene-KP509_33G035400                          ................................GIYVEA.AIIPHLSQ.KLA........G
Cecan.1G005600.V3.1                           ................................GVYLED.NAVPSLAG.KWK........S
Cecan.2G032100.V3.1                           ................................GVYLED.NAVPSLAG.KWK........S
Cecan.2G068600.V3.1                           ............................................................
AUR62020547                                   ................................GVYLEE.TAISALAS.KWK........G
Cs02g00014                                    ................................G.....SSVGGISS.KAPLQGSKLVSG
Cs10g00988                                    ................................GVYLEA.EAIPSLAG.KWK........G
gene-LOC101501370                             ................................AIYLQY.TSIPFLAD.KWK........G
gene-CKAN_00532200                            ................................GVYLEE.QAVPLLSP.KWK........G
CRGY0216791                                   ................................GVYLES.SAVTTLSG.KWK........G
Cla97C09G181670                               ................................GVYLEE.KAVPSLAG.KWS........G
```

```
                                                        70        80                       90        100
Medicago                                              KSSEE.LLETLDFYRDIIS..............GPFEKLIRGSKI.....RELSGPEYSR
EVM0003632                                            KNAEE.LAGSPGFFRDIVS..............GPFEKFTRITMV.....LPLTGQQYAE
gene-Apse012G0133800                                  KSAEE.LTDSVEFFRDIVT..............GPFEKFMQVTMI.....LPLTGGQYSE
OkoG031217                                            KAAGE.LASDAAFFRDVVT..............GEFEKFTRVTMI.....LPLTGEQYSE
Acora.07G100700.v1.1                                  KAADE.LFASGDFFIDVVK..............GPFEKFTRVSMI.....LPLTGQQYSN
CHI                                                   KAADE.LFGSGDFFMDVVK..............GPFEKFTRVSMI.....LPLTGQQYSD
CHI_2                                                 KAADE.LFASGDFFIDVVK..............GPFEKFTRVSMI.....LPLTGQQYSD
gene-QJS04_geneDACA014652                             KAADE.LFASGDFFIDVVK..............GPFEKFTRVSMI.....LPLTGQQYSD
gene-CEY00_Acc03848                                   KSAEE.LTESVEFFRDIVS..............GPFEKFTQVTMI.....LPLTGKQYSE
gene-CEY00_Acc03850                                   KSAEE.LTNSIEFFRDIVT..............GPFGKFTQVRMI.....LPLTGKEYSE
gene-CEY00_Acc27670                                   KSAEE.LTESVEFFRDIVS..............GPFEKFTQVTMI.....LPLTGKQYSE
gene-GOP47_0009997                                    KSIEE.LCNNELLFEEVLA..............APFEKLVRVVFL.....LPLTGPQYSE
EVM0010650                                            KPAAD.LASEAAFFRDVVT..............GEFEKFTRVTMI.....LPLTGAQYSD
gene-LOC109764886                                     KPAAD.LASDAAFFRDVVT..............GEFEKFTRVTMI.....LPLTGAQYSD
EVM0015959                                            KSADD.LTDSVDFYMDIVT..............GPFEKFVRVTMI.....LPLTGHQYSE
gene-LOC18440411                                      KSAEE.LLASLNFFMDIIN..............CPFEKMSHVAMI.....STLDGKEFSG
gene-LOC18440412                                      KSAEE.LLASPNFFMDIVN..............CPFQKMSHMAMI.....STLDGEEFSG
gene-LOC18440481                                      KSAAE.LANSDQFYSDVIK..............CPFEKFTRITTI.....LPLTGEQYSE
Anaoc.0011s0700.v0.9                                  KSAQE.LSDSVAFFRDIVT..............GPFEKFVRVTMI.....LPLTGPQYSE
Anaoc.0016s1186.v0.9                                  KSADE.LSESVAFYRDIVT..............GSFEKFVRVTMI.....LPLTGQQYSE
gene-ACMD2_09400                                      AAAEE.LAGDEHFYRDIVA..............GPFEKFTRVTML.....LPLTGQQYSE
AnM01G09141                                           ...............................................MI.....LPLMGR..SE
AnM01G09144                                           KTADE.LTDSVDFFKDIVT..............GPFEKFTQVTMI.....LPLTGQQYSE
CHI                                                   KSPEE.LAGSVGFFRDIAT..............GPFEKLTRVTMV.....LPLTGQQYSE
gene-AQUCO_01000652v1                                 KSAEE.LTDSIEFFRDIVT..............GPFEKFIMVTMI.....LPLTGQQYSD
AALP_AA5G189500                                       KNTEE.LTESVPFFREIVT..............GGFEKFIKVTMK.....LPLTGQQYSE
gene-LOC109850014                                     KDAEE.IAGSFEFFQDVSA..............GPFEKFTKVTMI.....LPLSGQQYSE
AVESA.00010b.r2.4AG0615430                            KAADE.LAADNAFFHDVVT..............GEFEKFTRVTMI.....LPLTGAQYSE
AVESA.00010b.r2.4DG0758920                            KAADE.LAADNAFFRDVVT..............GEFEKFTRVTMI.....LPLTGAQYSE
AVESA.00010b.r2.7CG0710680                            KAADE.LAADNAFFHDVVT..............GEFEKFTRVTMI.....LPLTGAQYSD
LXT01106                                              KSAEE.LNESHEFFRDIVT..............GPFEKFVQVTMI.....LPLTGKQYSE
LXT01112                                              KSAEE.LNESHEFFRDIVT..............GPFEKFVQVTMI.....LPLTGKQYSE
LXT05481                                              KSAEE.LNESFEFFRDIVT..............GPFEKFVQVRMI.....LPLTGQQYSE
LXT05485                                              KSAEE.LNESHEFFRDIVT..............GPFEKFVQVTMI.....LPLTGKQYSE
LXT05486                                              KSAEE.LNESHEFFRDIVT..............GPFEKFVQVTMI.....LPLTGKQYSE
LXT43699                                              KSAEE.LIETDEFFRAIVT..............GPFEKFVRVTMI.....LPLTGQQYAE
gene-LOC120080278                                     KSAAE.LMDSVEFFRDVVT..............GPFEKLTNVTLI.....LPLTGEEYAE
BVRB_2g036940                                         KGTDE.LVNSVEFFRDIVT..............GPFEKFTQVTMI.....LPLTGQQYSE
BVRB_2g036880                                         KSAIE.LADSVDFFRDIIT..............GPFEKFTQITMI.....QPLTGQQYSV
BPChr06G09462.v1.1                                    KSAEE.LTESVEFFRDIVT..............GPFEKFTRVTMI.....LPLTGQQYSE
gene-F511_19977                                       KSAQE.LGDSAEFFKDIVT..............GSFEKFTQVRMI.....LPLTGQQYSE
Bnt03G004203                                          KTAEE.LTESVEFFRDVVT..............GPFEKFTRITMI.....LPLTGLQYSE
gene-BRADI_1g03840v3                                  KPAAD.LAADAAFFRDVVT..............GEFEKFTRVTMI.....LPLTGAQYAE
Brahy.D01G0043800.v1.1                                KPAAD.LAADASFFRDVVT..............GEFEKFTRVTMI.....LPLTGAQYAE
Brahy.S02G0385600.v1.1                                KPAAD.LAADAAFFRDVVT..............GEFEKFTRVTMI.....LPLTGAQYAE
BjuA09g18460S                                         GTTEE.LAESVPFFRQIVT..............GPFEKFIKVTMK.....LPLTGQQYSE
BjuA09g18480S                                         KTTEE.LTESVPFFREIVT..............GSFEKFIKVTMK.....LPLTGQQYSE
BjuB05g27830S                                         KMTEE.LTESVSFFREIVTVLPHTTYFTDTLFKRSFEKFIKVTMK.....LPLMGQQYSE
BjuB08g05480S                                         ETVEE.LTESVPFFCEIVT.........................................
BnaA07g37900D                                         KTTEE.LTESVPFFREIVT..............GSFEKFIKVTMK.....LPLTGQQYSE
BnaA09g34840D                                         KTTEE.LTESVPFFREIVT..............GSFEKFIKVTMK.....LPLTGQQYSE
BnaA09g34850D                                         GTTEE.LAESVPFFRQIVT..............GPFEKFIKVTMK.....LPLTEQQYSE
BnaC08g26010D                                         KTTEE.LTESVPFFREIVT..............GSFEKFIKVTMK.....LPLTGQQYSE
BnaC08g26020D                                         KTTEE.LTESVNFFRHIVT..............GSFEKFIKVTMK.....LPLTGQQYSE
Bo6g068550                                            KTTEE.LTESVPFFREIVT..............GSFEKFIKVTMK.....LPLTGQQYSE
Bo8g088480                                            KTTEE.LTESVPFFREIVT..............GSFEKFIKVTMK.....LPLTGQQYSE
Bo8g089480                                            KTTEE.LTESVNFFRHIVT..............GSFEKFIKVTMK.....LPLTGQQYSE
C.cajan_29162                                         KSAHE.LTESVEFFRDIVT..............GPFEKFARVTMI.....LPLTGQQYAD
Csa02g076590                                          RMPRR...............................EFEKFVRVTMK.....VKLTGIQYSE
Csa04g041330                                          KTAEE.LTESVSFFHEIIS..............GAFEKFIKLTMI.....QPLFGHKYAE
Csa04g041350                                          ..................................................LPFSGQQYSE
Csa06g029680                                          KTAEE.LTESVPFFREIVT..............GAFEKFIKVTMK.....LPLTGQQYSE
Csa09g066000                                          KTAEE.LTESVSFFREIVT..............GAFEKFIKLTMK.....QPLFGHKYAE
Csa09g066040                                          KTAEE.LTESVPFFREIVT..............GAFEKFIKVTMK.....LPLTGQQYSE
Csa09g066060                                          KTAEE.LAESVPFFREIVT..............GAFEKFIKLTMK.....QPLFGHKYAE
Csa11g104750                                          KDAKT.LTESVPFFRQLVT..............GEFEKFVRVTMK.....VKLTGIQYSE
Csa20g023690                                          KSTKE.LTGSVPFFRDIVT..............GEFEKFIKVTMK.....RPLTGKEYSM
maker-Chr6-pred_gff_AUGUSTUS-gene-125.71              KTAEE.LTDSVDFFRDIVS..............GPFEKFTQVTMI.....LPLTGQQYSE
maker-Chr6-pred_gff_AUGUSTUS-gene-220.72              KSAEE.LTDSVEFFRDIVS..............GPFEKFTQVTMI.....LPLTGQQYSE
maker-Chr6-pred_gff_GeneMark.hmm-gene-225.16          ...............................................MI.....LPLTGQQYSE
gene_27594                                            KTSEE.LTESVEFFREIVT..............GPFHKFTRITMI.....LPLTGKQYSE
gene-LOC17885140                                      KTAEE.LTDSIPFFREIVT..............GAFEKFIKVTMK.....LPLTGQQYSE
gene-LOC107852750                                     KSSKE.LTDSVEFFRDIVT..............GPFEKFFRVTMI.....TPLTGKQYSE
gene-FH972_001554                                     KSREE.LTESVEFFRDIVT..............GPFEKFTRVTMI.....LPLTGQQYSE
Ct_T15992                                             KTAAE.LMDSVHFYSDIIN..............GPFEKLAEVAMI.....VPLVGMQHAE
Ct_T15993                                             KTAAE.LTDSVQFFRDIVT..............GPFEKFTQVTMI.....LPLTGKQYSE
gene-CIPAW_01G280400                                  KSADE.LSESVEFFRDIVT..............GPFEKFIRVTMI.....LPLTGHQYSE
gene-CFOL_v3_20734                                    KSKEE.LTDSVEFFRDIVT..............GPFEKFTQVTTI.....LPLTGQQYSE
gene-KP509_33G035400                                  KSLEE.LCENDLIFNEVVA..............APHDKLVRVTFL.....APLTGPQYSE
Cecan.1G005600.V3.1                                   KSAEK.LIESHEFFREIVT..............GPFEKFMRVTMI.....LPLTGQQYSE
Cecan.2G032100.V3.1                                   KSAEK.LIESHEFFREIVT..............GPFEKFLRVTMI.....LPLTGQQYSE
Cecan.2G068600.V3.1                                   .........................................MQVTMI.....LPLTGQQYSE
AUR62020547                                           KTSTE.LVESVEFFRDIVT..............GPFEKFTHVTMI.....LPLTGQQYSE
Cs02g00014                                            GAARPGLLA....F...................GTCQRRKSQRQLPSVSSLQVDGFIFSP
Cs10g00988                                            KTGTE.MTECLDFFRDVVT..............GPFQKFTRVTMI.....LPLTGQQYSE
gene-LOC101501370                                     KSATE.LTETVPFFRDIVT..............GPFEKFMQVTMI.....LPLTGQQYSE
gene-CKAN_00532200                                    KTGQE.LTDAVDFFRDVVT..............GPFEKFMQVTMI.....LPLTGQQYSE
CRGY0216791                                           KNVEE.LADSVDFYRDIVT..............GPFEKFTKVTML.....SPLTGKMYSE
Cla97C09G181670                                       KSAAE.LMDSVEFFRDVVT..............GSFEKFTNVTLI.....LPLTGKQYAE
```

```
                                                   110       120                                         130
                                                    .         .                                           .
Medicago                                           KVMENCVAHLKSVGTYGDAE................................AEAMQKFA
EVM0003632                                         KVTENCVAIWKAMGVYTEAE................................AAAVEKFR
gene-Apse012G0133800                               KVTENCIAFWKSIGIYTDAE................................AKATDKFI
OkoG031217                                         KVTENCVAYWKATGVYTDAE................................AVAVDKFK
Acora.07G100700.v1.1                               KVAENCEKYWKAIGIYTEEE................................AAAIKKFK
CHI                                                KVAENCEKYWKAIGIYTEEE................................AAAIEKFK
CHI_2                                              KVAENCEKYWKAIGIYTEEE................................AAAIEKFK
gene-QJS04_geneDACA014652                          KVAENCEKYWKAIGIHTEEE................................AAALEKFK
gene-CEY00_Acc03848                                KVTENCVAYWKAVGIYTDAE................................AKAVEKFI
gene-CEY00_Acc03850                                KVAENCVVYLKAVGTYTNGE................................AKAVTKFL
gene-CEY00_Acc27670                                KVTENCVAFWKAVGIHTDAE................................AKAVEKFI
gene-GOP47_0009997                                 KVVERMGLL....P.NPGIK................................EESTKQFL
EVM0010650                                         KVTENCVAYWKATGVYTDAE................................AAAVDKFK
gene-LOC109764886                                  KVTENCVAYWKATGVYTDAE................................AAAVDKFK
EVM0015959                                         KVTENCVAYWNAVGIYTEAE................................AKAVEKFK
gene-LOC18440411                                   KVVEKCKAIMEGSGTFSPQQ...............................SPLALHKFM
gene-LOC18440412                                   KVVDKLKAIMEASGTFSPQQ...............................SLLALHKFL
gene-LOC18440481                                   KVAENSVAGLKVAGIFGEEE................................AAAVDKFK
Anaoc.0011s0700.v0.9                               KVAENCIAIWKSLGLYTDAE................................SKAIEQFI
Anaoc.0016s1186.v0.9                               KVAENCVAIWKSLGRYTDAE................................SKAIEQFI
gene-ACMD2_09400                                   KVAENCLAHWKAAGVYTEAE................................GAAVDKFK
AnM01G09141                                        RTALLTGNKLECTPTLSPKQ................................PKS.....
AnM01G09144                                        KMVENCVAHWKSVGTYTDAE................................SEATQKFL
CHI                                                KVAENCAAAWKAAGVYTDEE................................ESAINDFL
gene-AQUCO_01000652v1                              KVVENCVAAWKAMGIYTEAE................................SKAVEEFI
AALP_AA5G189500                                    KVTENCVAIWKSLGIYTECE................................AKAVEKFL
gene-LOC109850014                                  KVSENCVAFWKAIGTYTDAE................................AAAVEKFK
AVESA.00010b.r2.4AG0615430                         KVTENCVAYWKAVGAYTDAE................................AAAVDKFK
AVESA.00010b.r2.4DG0758920                         KVTENCVAYWKAVGAYTDAE................................AAAVDKFK
AVESA.00010b.r2.7CG0710680                         KVTENCVAYWKAVGAYTDAE................................AAAVDKFK
LXT01106                                           KVSEVCMGICKSFGHYTDAE................................AKAIEEFV
LXT01112                                           KVSEVCMGICKSFGHYTDAE................................AKAIEEFV
LXT05481                                           KVSETCVGICKSFGQYTDVE................................VKAIEKFV
LXT05485                                           KVSEICMGICKSFGHYTDAE................................AKAIEEFV
LXT05486                                           KVSETCMGICKSFGHYTDAE................................AKAIEKFV
LXT43699                                           KVSENCVAIWKSLGLYTDAE................................AKAIEQFV
gene-LOC120080278                                  KVAENCAAAWKSMGIYTDEG................................AEAIRKFI
BVRB_2g036940                                      KVTENCVAFWKAIGVYTDNE................................AKAVEKFL
BVRB_2g036880                                      NMARTFVERWKDIGIYTDEE................................AKAVQIYL
BPChr06G09462.v1.1                                 KVSENCVAFWKSVGVYTDAE................................AKAIEKFL
gene-F511_19977                                    KVVENCVAYWKAIGTFSDAE................................SEATSKFL
Bnt03G004203                                       KVSENCVAIWKSIGIYTDAE................................EKAIQKFL
gene-BRADI_1g03840v3                               KVTENCVKYWQATGAYTDAE................................AAAVEKFR
Brahy.D01G0043800.v1.1                             KVTENCVKYWQATGAYTDAE................................AAAVEKFR
Brahy.S02G0385600.v1.1                             KVTENCVKYWKATGAYTDAE................................AAAVEKFR
BjuA09g18460S                                      KVTENCEAIWEPLGINA...................................GHAVVRFL
BjuA09g18480S                                      KVTENCVAIWKSLGIYTDSE................................AKAVERFL
BjuB05g27830S                                      RVTRG.RAIWES............................................................
BjuB08g05480S                                      ...................................................VERFL
BnaA07g37900D                                      KVTENCVAIWKSLGIYTDSE................................AKAVKKFL
BnaA09g34840D                                      KVTENCVAIWKSLGIYTDSE................................AKAVERFL
BnaA09g34850D                                      KVTENCEAIWESLGINAYSDSHICEHCDPPPSAKLRFNKITKTFGPYRHYSRGHAVVRFL
BnaC08g26010D                                      KVTENCVAIWKSLGIYTDSE................................AKAVERFL
BnaC08g26020D                                      KVTENCEAIWESLGINSNSDI....SCGPPPSAKLRFNKITKTFGPSRHYSRGQAVKRFL
Bo6g068550                                         KVTENCVAIWKSLGIYTDSE................................ANAVKKFL
Bo8g088480                                         KVTENCVAIWKSLGIYTDSE................................AKAVERFL
Bo8g089480                                         KVTENCEAIWESLGINSNSDI....SCGPPPSAKLRFNKITKTFGPSRHYSRGQAVKRFL
C.cajan_29162                                      KVSENCVAIWKSLGIYTDAE................................AKAMEQFV
Csa02g076590                                       KVVEYCEEILKASGKYTRSE................................AKAIDIFL
Csa04g041330                                       GGF...........IYTVRE................................AKAVERFL
Csa04g041350                                       KVTENCVAIWKSLGIYTECE................................AKAVEKFS
Csa06g029680                                       KVTENCVAIWKSLGIYTECE................................AKAVEKFL
Csa09g066000                                       GVTKKCVAILKVRGTYTDCE................................ANAVEKFL
Csa09g066040                                       KVTENCVAIWKSLGIYTECE................................AKAVEKFL
Csa09g066060                                       GVTKKCVALWKFRGIYTQCE................................AIAVEMFL
Csa11g104750                                       KVVEYCEEILKASGKYTRSE................................AKAIHKFL
Csa20g023690                                       KVAKNCVMIWKSLGIYTQSE................................AKALKRFL
maker-Chr6-pred_gff_AUGUSTUS-gene-125.71           KVTENCVAYWKAVGTYTDAE................................AKAIEKFI
maker-Chr6-pred_gff_AUGUSTUS-gene-220.72           KVTENCVAYWKAVGTYTDAE................................AKAIEKFI
maker-Chr6-pred_gff_GeneMark.hmm-gene-225.16       KVTENCVAYWKAVGTYTDAE................................AKAIEKFI
gene_27594                                         KVAENCVAIWKSLGIYSDAE................................AKAIDKFL
gene-LOC17885140                                   KVTENCVAIWKSLGVYTDCE................................AKAVEKFL
gene-LOC107852750                                  KVAENCVANWKALGTYGDAE................................SEAIEKFL
gene-FH972_001554                                  KVSENCVAFWKSVGIYTDAE................................AKAIEKFL
Ct_T15992                                          TLSKMCVAIWKAEGTYTDAD................................SATIAKYL
Ct_T15993                                          KVSEMCVGVWKAQGTYTDAD................................TATIEKFL
gene-CIPAW_01G280400                               KVTENCVAFWKSVGIYTDAE................................AKAIEEFL
gene-CFOL_v3_20734                                 KVSENCVAFWKSVGIYTDAE................................AKAIEKFI
gene-KP509_33G035400                               KVVERIGLI....E.NSGVK................................EESLKLFL
Cecan.1G005600.V3.1                                KVSENCVAIWKSLGLYTDAE................................AKAIEQFV
Cecan.2G032100.V3.1                                KVSEHCVAIWKSLGLYTDAE................................AKAIEQFV
Cecan.2G068600.V3.1                                KVSENCVAIWKSLGLYTDAE................................AKAIEQFV
AUR62020547                                        KVTENCVAFWKAIGIYTDDE................................AKAVEKFL
Cs02g00014                                         AVQPPDSSKTHSLG......................................GAAVEKIK
Cs10g00988                                         KVTENCVAYWKSIGTYTEAE................................AAAVEKFK
gene-LOC101501370                                  KVSENCVAIWKSLGIYTDEE................................AKAIEKFL
gene-CKAN_00532200                                 KVTENCVAYWKAVGLYTEAE................................AIAVEKFK
CRGY0216791                                        KVAENCVAIWKALGLYTDAE................................AEAIDKFT
Cla97C09G181670                                    KVAENCAAAWKSMGIYTDEG................................AEAIQKFI
```

```
                                               140       150       160
Medicago                                      EAFKPVNFPPGASVFYRQSPD..GIL.GLS...FSPDT......................
EVM0003632                                    EVFKEEVFPHGSSILFTSSPS..G.L.KIG...FSKDG......................
gene-Apse012G0133800                          EVFKDENFPPGTSILFTQSPH..GSL.TIS...FSKDG......................
OkoG031217                                    EAFKPQSFPPGASILFTHSPT..GVL.TVA...FSKDS......................
Acora.07G100700.v1.1                          AAFKDQTFPPGAAILFTQSPS..STL.TIA...FSKDG......................
CHI                                           ASFKDQTFPPGAAILFTQSPS..GSL.TIA...FSKDG......................
CHI_2                                         ATFKDQTFPPGAAILFTQSPS..GSL.TIA...FSKDG......................
gene-QJS04_geneDACA014652                     AAFKDQTFPPGAAILFTQSPS..GSL.TIA...FSKDG......................
gene-CEY00_Acc03848                           EVFKDETFPPGASIMFTQSPH..GSL.TIS...FSKDC......................
gene-CEY00_Acc03850                           KVFKNETFPPGASVLFTQSTH..GSL.TIS...FSKDG......................
gene-CEY00_Acc27670                           EVFKDETFPPGASILFTQSPL..GSL.TIS...FSKDG......................
gene-GOP47_0009997                            EIFKPENFPPRTSLICSFTEE...AL.KLA...FMKTD......................
EVM0010650                                    EAFGPHSFAPGASILFTHSPA..GLL.TVA...FSKDS......................
gene-LOC109764886                             EAFGPHSFAPGASILFTHSPA..GLL.TVA...FSKDS......................
EVM0015959                                    EVFKEETFPPGSSILFTQSPL..GTL.TIG...FSKDG......................
gene-LOC18440411                              EAFKGKMLTYGSAIFFTYLEA..G.L.SIA...IAEDG......................
gene-LOC18440412                              EAFKGKMLTYGSAIFFAYSKA..G.L.LIA...ITEDG......................
gene-LOC18440481                              EVFKGLNFPPSSSILFTYSTS..GSL.TIG...ISNDG......................
Anaoc.0011s0700.v0.9                          EAFKGENFPPGASILFTISPA..GSL.TIG...FSKNE......................
Anaoc.0016s1186.v0.9                          KVFKDENFPPGASILFTISPI..GSL.TIG...FSKHE......................
gene-ACMD2_09400                              EAFKAETFPPGSSILFTHSPS..GSL.TIA...FSSDS......................
AnM01G09141                                   .SFKDQTFPPGASIIFTQSPL..GSL.TIS...FSKDGSLPDEKGDELIENEQLSLTEKG
AnM01G09144                                   QVFKNETFPPGASILFTQSPL..GSL.TIS...FSKDG......................
CHI                                           QAFKPRSFPPGASIFFTHSPS..GSL.SIG...FSKEG......................
gene-AQUCO_01000652v1                         QVFKDETFPPGSSILFTQSS...PAL.TIG...FSKDG......................
AALP_AA5G189500                               EVFKDQSFAPGASILFALSPT..GSL.TIA...FSKDD......................
gene-LOC109850014                             EVFEPENFPPGSSILFTHSPA..GSL.TIA...FSKDG......................
AVESA.00010b.r2.4AG0615430                    EAFKAETFPPGASILFTHSSA..GVL.TVA...FSKDS......................
AVESA.00010b.r2.4DG0758920                    EAFKDETFPPGASILFTHSPA..GVL.TVA...FSKDS......................
AVESA.00010b.r2.7CG0710680                    DAFKAETFPPGASILFTHSPA..GVL.TVA...FSKDS......................
LXT01106                                      SIFKNQTFPPGSSTLFTQSPN..GSL.TVS...FSKHD......................
LXT01112                                      SIFKNQTFPPGSSTLFTQSPN..GSL.TVS...FSKHD......................
LXT05481                                      SIFKDETFPPGYSTIFTQSPN..GPL.TVS...FSKHE......................
LXT05485                                      SIFKNQTLPPGSSTLFTQSPN..GSL.TVS...FSKHD......................
LXT05486                                      SIFKNQTFPPGSSTLFTQSPN..GSL.TVS...FSKHD......................
LXT43699                                      SIFKDETFPPGSSILFTLSPN..GSL.TIS...FSKDV......................
gene-LOC120080278                             DAFKNENFPPSSSILFTHFPP..NTL.SIS...FSKDG......................
BVRB_2g036940                                 EAFKNEKFPAGHSILFTQSPL..GSL.TIG...FCKHD......................
BVRB_2g036880                                 EAFDDKKFSPGESILFTQSPD..GKF.TIA...FSEHN......................
BPChr06G09462.v1.1                            EVFKDEKFPPGASILFTQSPN..GSL.TIG...FSKDG......................
gene-F511_19977                               EIFKNETFPPGASICFTQSPL..GAL.SVSGSVFSKDG......................
Bnt03G004203                                  EVFKDENFPPGSSVLFTQSPT..GSL.AIS...FSKDQ......................
gene-BRADI_1g03840v3                          EAFQPHSFAPGGSILFTHSPA..GVL.TVA...FSKDS......................
Brahy.D01G0043800.v1.1                        EAFQPHSFAPGGSILFTHSPA..GVL.TVA...FSKDS......................
Brahy.S02G0385600.v1.1                        DAFQPHSFPPGASILFTHSPA..GVL.TVA...FSKDS......................
BjuA09g18460S                                 EIFKHKKFPPGASILFAISPK..GSL.TVA...FSSDD......................
BjuA09g18480S                                 EVFKDETFPPGASILFALSPE..GSL.TVA...FSKDD......................
BjuB05g27830S                                 ............................VV...FLKDD......................
BjuB08g05480S                                 EIFKNKNFPPGASILFALSHE..DSL.TVA...VSNDD......................
BnaA07g37900D                                 EVFKDQTFPPGASILFALSPN..GSL.TVI...FTK........................
BnaA09g34840D                                 EVFKDETFPPGASILFALSPE..GSL.TVA...FSKDD......................
BnaA09g34850D                                 EIFKHKKFPPGASILFAISPK..GSL.TVA...FSSDD......................
BnaC08g26010D                                 EVFKDETFPPGASILFALASK..GSL.TVA...FSKDD......................
BnaC08g26020D                                 EIFKPKKFPPGASILFAISPK..GSL.TVA...FSNDD......................
Bo6g068550                                    EVFKDQTFPPGASILFALSPN..GSL.TIA...FSKDD......................
Bo8g088480                                    EVFKDEKFPPGASILFALASK..GSL.TVA...FSKDD......................
Bo8g089480                                    EIFKPKKFPPGAFILFAISPK..GSL.TVA...FSSDD......................
C.cajan_29162                                 TAFKDETFPPGSSILFTVFPK..GSL.AIS...FSKDG......................
Csa02g076590                                  LIFKDQDFPPGSSVLFALCSK..GSL.TIA...FSKDE......................
Csa04g041330                                  EVFKDQAFLPGASILFAFSPN..GSL.TVA...FSKDD......................
Csa04g041350                                  EIFKEETFPPGASILFALSPH..GSL.TVA...FSKDD......................
Csa06g029680                                  EIFKEETFPPGASILFALSPH..GSL.TVA...FSKDD......................
Csa09g066000                                  EVFKDETFLPGASILFALSPN..GSL.TVA...FSKDD......................
Csa09g066040                                  EIFKEETFPPGASILFALSPH..GSL.TVA...FSKDD......................
Csa09g066060                                  EVFKDQAFLPGASILFAFSPN..GSLTTVA...FSKDD......................
Csa11g104750                                  LIFKDQDFPPGSSVLFALCSK..GSL.TV.......NE......................
Csa20g023690                                  KIFKYQTFPPGASILFALSPK..GSL.TIA...FSKDD......................
maker-Chr6-pred_gff_AUGUSTUS-gene-125.71      EVFKDETFPPGSSILFTQSPL..GSL.TIA...FSKDG......................
maker-Chr6-pred_gff_AUGUSTUS-gene-220.72      EVFKDETFPPGGSILFTQSPL..GSL.TIA...FSKDS......................
maker-Chr6-pred_gff_GeneMark.hmm-gene-225.16  EVFKDETFPPGGSILFTQSPL..GSL.TIA...FSKDS......................
gene_27594                                    EIFKDENFLPGSSILFTQSPN..GFL.KIS...FSKDE......................
gene-LOC17885140                              EVFKDETFPPGASILFALSPN..GSL.TVA...FSKDD......................
gene-LOC107852750                             SAFQSENFPPGASILFTQSPA..GSL.TIS...FSKDD......................
gene-FH972_001554                             EVFKDETFPPGSSILFTQSPN..GSL.TIS...FSKDG......................
Ct_T15992                                     EAFKDQKFSPGSSILYTTTPD..GLV.MVS...FVKDG......................
Ct_T15993                                     H...........................IS...FSKDG......................
gene-CIPAW_01G280400                          KVFKDEKFPPGSSILFTQSPN..GSL.TIS...FSKDE......................
gene-CFOL_v3_20734                            EVFKEETFPPGSSILFTQSPN..GAL.TIA...FSKDG......................
gene-KP509_33G035400                          EIFKTENFPPGTSVVVSFTKS...AL.KIA...FTKDN......................
Cecan.1G005600.V3.1                           SIFKDETFPPGSSILFTLSPN..GSL.TIS...FSKDG......................
Cecan.2G032100.V3.1                           SIFKDETFPPGSSTLFTLSPN..GSL.TIS...FSKDG......................
Cecan.2G068600.V3.1                           SIFKDETFPPGSSILFTLSPN..GSL.TIS...FSKDG......................
AUR62020547                                   ETFKDEKFPPGHSILFTQSPH..GSL.TIG...FSKHD......................
Cs02g00014                                    EVFKNETFPPVSAILFTQVSS..GSL.TIA...FVKDS......................
Cs10g00988                                    EVFKNETFPPGSAILFTQAPS..GSL.TIA...FSKDS......................
gene-LOC101501370                             SVFKDQTFPSGSSILFTVSPKGAGSL.TIS...FSKDG......................
gene-CKAN_00532200                            QVFKEETFPPGSTIFFTLNPS..GSL.TIG...FSKDS......................
CRGY0216791                                   EVFKDETFPPASSILFTHSPH..GAL.TIS...FSKDG......................
Cla97C09G181670                               DVFKNEHFPPGSSILFTHLPP..NTL.SIS...FSKNE......................
```

```
                                                 170       180       190       200
Medicago                                .........SIPE...KEA.A.L...IENKAVSSAV..LETMIGEHAVSPDLKRCLAARL
EVM0003632                              .........SIPQ...TTV.K.V...IENRAISEAV..LESIIGEHGVSRDARQSLAFRL
gene-Apse012G0133800                    .........STPE...VGN.A.V...IDNKLLSEAI..LESMIGKHGVSPAAKKSLAARL
OkoG031217                              .........SVPE...AGS.A.A...IENRPLCEAV..LESIIGEHGVSPAAKLSLATRV
Acora.07G100700.v1.1                    .........SIPD...SSN.A.V...IENKALSEAV..LESIIGQHGVSPAAKKSLASRV
CHI                                     .........SIPD...SSH.A.V...IENMALSEAV..LESIIGQHGVSPVAKKSLASRV
CHI_2                                   .........SIPD...SSN.A.V...IENKALSEAV..LESIIGQHGVSPAAKKSLASRV
gene-QJS04_geneDACA014652               .........SIPN...SGN.V.V...IENKALSEAV..LESIIGQHGVSPLAKKSLASRV
gene-CEY00_Acc03848                     .........SVPE...TGN.A.V...IENKHLSEAV..LESIIGKHGVSPAAKQSLAARM
gene-CEY00_Acc03850                     .........SIPK...TRN.A.I...IKNKQLAEAV..LESIIGKDGVSPAAKQSLAARM
gene-CEY00_Acc27670                     .........SIPE...TGN.A.V...IENKHLSEAV..LESIIGKHGVSPAAKRSLAARM
gene-GOP47_0009997                      .........DFPE...DAD.A.V...IEDKWLARAF..LASIIGKDGVSPLAKLSFAERM
EVM0010650                              .........SVPE...SGG.V.A...IDNARLCEAV..LESIIGEHGVSPAAKLSLATRV
gene-LOC109764886                       .........SVPE...SGG.V.A...IDNARLCEAV..LESIIGEHGVSPAAKLSLATRV
EVM0015959                              .........SIPE...VGK.A.V...IENKPLSQAV..LESIIGKHGVSPEAKQSLAVRV
gene-LOC18440411                        .........SIPD...KGD.V.M...IEDKALAQAL..LDSIIGEHGVSPAAKRSVAERL
gene-LOC18440412                        .........SVPE...QED.V.M...IEDKALTQAL..LDSIIGEHGVSPAAKRSVAKRL
gene-LOC18440481                        .........SSPE...DGI.T.V...IDNKGLAETI..LESIIGKHGVSPEATKCLAERI
Anaoc.0011s0700.v0.9                    .........SIGE...SGK.T.V...IENKLLAESV..LESIIGKNGVSPAAKKSLAERL
Anaoc.0016s1186.v0.9                    .........STGE...SGKVA.V...IDNKLLAESV..LESIIGKNGVSPAAKKNLAERF
gene-ACMD2_09400                        .........SVLE...VGN.A.V...IENRALCEAI..LESIIGEHGVSPATKRSLALRV
AnM01G09141                             IEVIENIQLSLPK...KGI.E.V...IENKQLSEAV..LESIIGKHGVSPEAKQSLAVRL
AnM01G09144                             .........SLPE...NGK.E.V...IENKQLSEAV..LQSIIGKHGVSPEAKKSLAARL
CHI                                     .........GVPAAEAAAA.A.V...IKNEKLKDAV..LESIIGENGVSPAVKRSLAQRI
gene-AQUCO_01000652v1                   .........SIPK...EGK.A.T...IANKPLSEAV..LESIIGKHGVSPEAKHSLAVRI
AALP_AA5G189500                         .........SIPE...TGK.A.V...IENKLLAEAV..LESIIGKNGVSPGARLSVAKRL
gene-LOC109850014                       .........SVPE...AGV.T.V...IENKALTRAI..LESIIGEHGVSPAAKKSLATRM
AVESA.00010b.r2.4AG0615430              .........SVPE...SGG.V.A...IENRPLCEAV..LESIIGEHGVSPAAKLSLATRV
AVESA.00010b.r2.4DG0758920              .........SVPE...SGG.V.A...IENRPLCEAV..LESIIGEHGVSPAAKLSLATRV
AVESA.00010b.r2.7CG0710680              .........SVPE...SGG.V.A...IENRPLCEAV..LESIIGEHGVSPAAKLSLATRV
LXT01106                                .........SIPE...VGV.A.V...IENKLISEAI..LESIVGKNGVSPAARQSLASRL
LXT01112                                .........SIPE...VGV.A.V...IENKLISEAI..LESIVGKNGVSPCSKTKPGLQI
LXT05481                                .........SIPE...AGI.A.V...IENKVVSEVI..LESIVGKNGVSPATRLSVASRL
LXT05485                                .........SIPE...VGV.A.V...IENKLISEAI..LESIVGKNGVSPAARQSLASRL
LXT05486                                .........SIPE...VGV.A.V...IENKLISEAI..LESIVGKNGVSPAARQSLASRL
LXT43699                                .........SIPE...VGA.A.V...IENRLLSESV..LESMIGKHGVSPLAKQSLATRL
gene-LOC120080278                       .........CSGE...KQE.E.MVRKIENKLLSESV..LESIIGKNGVSPAARLSLATRF
BVRB_2g036940                           .........LIPE...AGN.V.V...IENKNLSEAV..LESIIGQHGVSPEAKQSLATRV
BVRB_2g036880                           .........AIPK...DGV.A.V...IQNKTLPEEI..LNSIIGETGVCPQARQILAERI
BPChr06G09462.v1.1                      .........SIPE...IGN.A.V...IENKLLSEAV..LESIIGKHGVSPEAKQSLATRS
gene-F511_19977                         .........SLPE...KEN.A.V...IENKQLSEAV..LESIIGKHGVSPEAKRSLGERI
Bnt03G004203                            .........SIPE...KEN.V.V...IENKLLAESV..LESIVGKHGVSPEARKCMAARL
gene-BRADI_1g03840v3                    .........SVPA...SGA.A.A...IENRPLCEAV..LESIIGEHGVSPAAKLSLATRV
Brahy.D01G0043800.v1.1                  .........SVPE...SGA.T.A...IQNRPLCEAV..LESIIGEHGVSPAAKLSLATRV
Brahy.S02G0385600.v1.1                  .........SVPE...SGA.A.A...IENRPLCEAV..LESIIGEHGVSPAAKLSLATRV
BjuA09g18460S                           .........GIPK...RGN.T.A...IENKFLAEAI..LESIIGKNGVSPGARLSVAERL
BjuA09g18480S                           .........SIPE...TGK.V.V...IENKLLAEAV..LESIIGKNGVSPGARLSVAERL
BjuB05g27830S                           .........SIPK...TSK.T.V...IKNKLLAEAV..LESIIGKNGVSPGARFSVAERL
BjuB08g05480S                           .........GIPK...RGK.T.A...IANKFLAEAV..LESII.................
BnaA07g37900D                           ......................I...LYNIDLCACVYMLYSMILFLHIEP..........
BnaA09g34840D                           .........SIPE...TGK.V.V...IENKLLAEAV..LESIIGKNGVSPGARLSVAERL
BnaA09g34850D                           .........GIPK...RGN.T.A...IENKFLAEAI..LESIIGKNGT............
BnaC08g26010D                           .........SIPE...TGK.A.V...IENKLLAEAV..LESIIGKNGVSPGARLSVAERL
BnaC08g26020D                           .........GIPK...RGN.T.A...IENKFLAEAV..LESIIGKNGVSPGARLSVAERL
Bo6g068550                              .........SIPK...TGK.A.V...IENKLLAEAV..LESIIGKKGVSPGARLSLAERL
Bo8g088480                              .........SIPE...TGK.A.V...IENKLLAEAV..LESIIGKNGVSPGARLSVAERL
Bo8g089480                              .........GIPK...RGN.T.A...IENKFLAEAV..LESIIGKNGVSPGARLSVAERL
C.cajan_29162                           .........SIPE...VGS.T.I...IENKLLSEAV..LESMIGKHGVSPQAKQSLATRL
Csa02g076590                            .........RVPR...NGK.A.V...IKNKLLGEAV..LESMIGKNGVSPATRKSLAERL
Csa04g041330                            .........SIPE...TGR.A.V...IQNKLLAEGV..LESFIERNGASAGARLSAAERL
Csa04g041350                            .........SIPE...TGK.A.V...IENKLLAEAV..LESIIGKNCVSPGTRLSVAERL
Csa06g029680                            .........SIPE...TGK.A.V...IENKLLAEAV..LESIIGKNGVSPGTRLSVAERL
Csa09g066000                            .........NIPE...TGK.A.V...IQNKLLAEDV..LESFIGKNGASPGVRLSVAERL
Csa09g066040                            .........SIPE...TGK.A.V...IENKLLAEAV..LESIIGKNGVSPGTRSSVAERL
Csa09g066060                            .........SIPQ...TGR.AHV...IRNKLLAEAV..LESFIEKNGASPGVRLSVAERL
Csa11g104750                            .........RVPR...NGK.A.V...IKNKLLGEAV..LESMIGKNGVSPATRKSLAERL
Csa20g023690                            .........TIPK...AGQ.A.V...VKNKLLAEAV..LESIIGKEKTKQLMRNSRGN..
maker-Chr6-pred_gff_AUGUSTUS-gene-125.71      .........SLPE...TGT.M.V...IENKQLSEAV..LESIIGKHGVSPAAKKSLAARM
maker-Chr6-pred_gff_AUGUSTUS-gene-220.72      .........SLPE...TGT.A.V...IENKQLSEAV..LESIIGKHGVSPAAKKSLAARV
maker-Chr6-pred_gff_GeneMark.hmm-gene-225.16  .........SLPE...TGT.V.V...IENRQLSEAV..LESIIGKHGVSPAAKKSLAARV
gene_27594                              .........SIPE...TWV.S.E...IESKLLSEAI..LESIIGKNGVSPAARQSIASTL
gene-LOC17885140                        .........SIPE...TGK.A.V...IENKLLAEAV..LESIIGKNGVSPGTRSSVAERL
gene-LOC107852750                       .........SVPG...TGN.A.V...IENKQLSEAV..LESIIGKHGVSPAAKNSLAKRV
gene-FH972_001554                       .........SLPE...IGN.A.V...IENKLLSEAV..LESIIGKHGVSPEAKQSLAARL
Ct_T15992                               .........IIPE...TPI.V.V...LENKKLGHAL..FESVIGKNGVSPEAKQSLASRL
Ct_T15993                               .........AIPE...AAT.I.V...LENEKLAHAV..IESVIGEHGVSPAAKQSLASRL
gene-CIPAW_01G280400                    .........SIPE...SGN.A.V...IKNKLLSEAV..LESIIGKHGVSPAAKKSLATRV
gene-CFOL_v3_20734                      .........VIPE...VGK.A.V...IENKLLAEGL..LESIIGKHGVSPVAKQCLATRL
gene-KP509_33G035400                    .........EIPK...EPA.A.V...IEDEAFACGF..LASIIGKDGVSPAAKNSFAKRI
Cecan.1G005600.V3.1                     .........SIPE...VGA.A.V...IENKLLSEAV..LESMIGKHGVSPEAKQSLASRL
Cecan.2G032100.V3.1                     .........SIPE...VGA.A.V...IENKLLSEAF..LESVIGEHGVSPEAKQSLASRL
Cecan.2G068600.V3.1                     .........SIPE...VGA.A.V...IENKLLSEAV..LESMIGKHGVSPEAKQSLASRL
AUR62020547                             .........SIPT...VGS.A.V...IENKNLSEAV..LESIIGKHGVSPEAKQCIAARV
Cs02g00014                              .........SILG...QGT.I.V...IKNKQLAEAV..LESIIGKYGVSPDAKQSLATRV
Cs10g00988                              .........SVPE...QGT.T.V...IENKQLAEAV..LESIIGKHGVSPEAKQSLATRV
gene-LOC101501370                       .........SIPE...VES.A.V...IENKLLSEAV..LESMIGAHGVSPAAKQSLASRL
gene-CKAN_00532200                      .........SIPE...QAA.G.V...IENKQMSEAV..LESMIGKHGVSPEAKQSLATRV
CRGY0216791                             .........CLPE...VGN.A.V...IENKLLTEAV..LESIIGKHGVSPAAKKSLAARL
Cla97C09G181670                         .........CIGE...EEE.G.MVRKIENKLLSESV..LESIIGKHGVSPAARLSLAKRL
```

```
                                                        210       220
                                                          .         .
Medicago                                                  PALLNEGAFK.I.................GN.............................
EVM0003632                                                SESLK.......................................................
gene-Apse012G0133800                                      SKLLNECNNK.V.................PVDG..........K...........SEA..
OkoG031217                                                AELLKGATLT.........................A.......EP........VSVSV..
Acora.07G100700.v1.1                                      SDLLKELDSQ.K.................AQV............................
CHI                                                       SNLLKELDSQ.K.................AQVESVN........................
CHI_2                                                     SDLLKELDSQ.K.................ALV............................
gene-QJS04_geneDACA014652                                 SDLLKEFDSQ.K.................AQVESVN........................
gene-CEY00_Acc03848                                       SELLKECDNE.A.................PETV..........K...........PQT..
gene-CEY00_Acc03850                                       SELLKQCDNK.T.................LEKV..........K...........TKS..
gene-CEY00_Acc27670                                       SEVLKEYGNE.A.................PETT...........................
gene-GOP47_0009997                                        SRCL........................................................
EVM0010650                                                AELLEGA...........................AA.......EP........VSVSV..
gene-LOC109764886                                         AELLEGAA...L.................AGGEPAA.......EA........VSVSV..
EVM0015959                                                SELLKDGYGE.R..................A.............................
gene-LOC18440411                                          SEVLSIA.....................................................
gene-LOC18440412                                          SEALSKA.....................................................
gene-LOC18440481                                          SQLLSNHSIP.T.................PKLPVDQNNI..............IPV....
Anaoc.0011s0700.v0.9                                      SKLLNHGEEKSK.................VDNCGREF................KAVDS..
Anaoc.0016s1186.v0.9                                      SKLLNAEVKS.E.................ADHCAKEF................KGMDSMI
gene-ACMD2_09400                                          SELLKEFGGA.K.................EAEVE.........KP........VEVSA..
AnM01G09141                                               SDLFKLHDN..........................PK................LE.....
AnM01G09144                                               SDLINQHENP.K.................KE.............................
CHI                                                       FELLR.......................................................
gene-AQUCO_01000652v1                                     SEMMKNQGDT.V.................EEGMV..........................
AALP_AA5G189500                                           AQLMKNNKIE.E.................EEETK.TD....QEEAKD......VSVGDKL
gene-LOC109850014                                         SEFMGGVEEV.K.................ERVQV.................E.QAVIA..
AVESA.00010b.r2.4AG0615430                                AELLK.......................................................
AVESA.00010b.r2.4DG0758920                                AELLK.......................................................
AVESA.00010b.r2.7CG0710680                                AELLKGAA...P.................VD............EP........VSA....
LXT01106                                                  SELLKEGGKA.N................................................
LXT01112                                                  IRVTERGWQS..................................................
LXT05481                                                  SELLKSN.....................................................
LXT05485                                                  SELLKEGGKA.N................................................
LXT05486                                                  SELLKEGGKA.N................................................
LXT43699                                                  SQLLKEDGKA.T.................A..............................
gene-LOC120080278                                         SQLFNHYEPN.T.................PNICN.....PKQI.................
BVRB_2g036940                                             SELLNKEKLE.S.................NSAILGVA....NEKT........THVVHEL
BVRB_2g036880                                             YELLNKEVLA.G.................GK.............................
BPChr06G09462.v1.1                                        SELLKENDDK.V.................AENG..........K...........SEI..
gene-F511_19977                                           SKVLKSSSTE..................................................
Bnt03G004203                                              SELLNAIDGD.E.................NLHCEKVCE...EQK.........ICLEAA.
gene-BRADI_1g03840v3                                      AELLKAA....................................EP........VSVSL..
Brahy.D01G0043800.v1.1                                    AELLKAA....................................EP........VSVSL..
Brahy.S02G0385600.v1.1                                    AELLKAGAG..P.................VGGEPAA.......EP........LSVSL..
BjuA09g18460S                                             AQLMKIERRA.ETLILADKKFMKNNEVKEDATKTKTD....QEEAND......LSLGDKL
BjuA09g18480S                                             AQLMNSDKIE.E.................DATKT..D....QEEAND......LPLGDKL
BjuB05g27830S                                             SQLMKNNKVE.E.................DATKT..........................
BjuB08g05480S                                             ............................................................
BnaA07g37900D                                             ............................................................
BnaA09g34840D                                             AQLMNSDKVE.E.................DATKT..D....QEEAND......LPLGDKL
BnaA09g34850D                                             .............LILADKKFMKNNEVKEDATKTKTD....QEEAND......LSLGDKL
BnaC08g26010D                                             AQLMNSDKVE.E.................DATKT..D....QEEANN......LPLGDKL
BnaC08g26020D                                             AQVMKNEGRA.ETLILADKKFMKNNEVKEDATKTKTD....QEEAND......LSLGDKL
Bo6g068550                                                SQLMKENKVE.K.................DATKT.DN....QDEAND......VSLGDKL
Bo8g088480                                                AQLMNSDKVE.E.................DATKT..D....QEEANN......LPLGDKL
Bo8g089480                                                AQVMKNEGRA.ETLILADKKFMKNNEVKEDATKAKTD....QEEAND......LSLGDKL
C.cajan_29162                                             AELFKPNTDS.I.................TK.............................
Csa02g076590                                              SKLMSNNDR...................................................
Csa04g041330                                              AQLMKENKVE.E.................EEN............................
Csa04g041350                                              AQLMKENKVE.E.................EEN............................
Csa06g029680                                              AQLMKENKVE.E.................EEN............................
Csa09g066000                                              AQLMNENKVE.E.................EEN............................
Csa09g066040                                              AQLMKKNKVE.E.................EEN............................
Csa09g066060                                              AQLMNENKVE.E.................EEN............................
Csa11g104750                                              SKLMSNNAR...................................................
Csa20g023690                                              ............................................................
maker-Chr6-pred_gff_AUGUSTUS-gene-125.71                  AELLKEKPEA.Q.................TAAAAAAE.......................
maker-Chr6-pred_gff_AUGUSTUS-gene-220.72                  SELLKEKPEA..................................................
maker-Chr6-pred_gff_GeneMark.hmm-gene-225.16              SELLKEKPEA..................................................
gene_27594                                                SKLLKENNGS.T.................NDHKAAGEID...........GD.INLKATK
gene-LOC17885140                                          AQLMEENKVE.G.................ETS..............D......LSLEDKS
gene-LOC107852750                                         SELLKSNAEA...................PVFEK..........................
gene-FH972_001554                                         AEALKDNDEK.V.................VGNG..........K................
Ct_T15992                                                 ADLMN.......................................................
Ct_T15993                                                 SDFMKQFDEK.A.................TANVESKLV......................
gene-CIPAW_01G280400                                      SELLKDSNDK.E.................AENQ..........K...........LMA..
gene-CFOL_v3_20734                                        SELL........................................................
gene-KP509_33G035400                                      YNHLK.......................................................
Cecan.1G005600.V3.1                                       SELLKEGGKA.T.................D..............................
Cecan.2G032100.V3.1                                       SELLKEGGKA.T.................D..............................
Cecan.2G068600.V3.1                                       SELLEEGGKA.T.................D..............................
AUR62020547                                               SGLLNPENAE.A.................NSTNG.VE....SEKS........AKVDTEK
Cs02g00014                                                SQLLDSGNRK.G................FDLRTLQRK....RRR................
Cs10g00988                                                SQLLDSGNID.I.................EKVCTE......GEKAAIHLPPSVLELQV..
gene-LOC101501370                                         SELFKEGSDT.S.................ISPK...........................
gene-CKAN_00532200                                        SQLFEGI.....................................................
CRGY0216791                                               SELLVEKDCE.A.................GGDE..........K...........I....
Cla97C09G181670                                           SQLLNHYEPN.N.................PKNCN.....PLEEDPTP......KHI....
```

```
Medicago                                           ...........................................................
EVM0003632                                         ...........................................................
gene-Apse012G0133800                               ...........EKCVKE..........................................
OkoG031217                                         ...........................................................
Acora.07G100700.v1.1                               ...........................................................
CHI                                                ...........................................................
CHI_2                                              ...........................................................
gene-QJS04_geneDACA014652                          ...........................................................
gene-CEY00_Acc03848                                ...........ETAAPK..........................................
gene-CEY00_Acc03850                                ...........QTTATKETSA......................................
gene-CEY00_Acc27670                                ............TAAPK..........................................
gene-GOP47_0009997                                 ...........................................................
EVM0010650                                         ...........................................................
gene-LOC109764886                                  ...........................................................
EVM0015959                                         ...........................................................
gene-LOC18440411                                   ...........................................................
gene-LOC18440412                                   ...........................................................
gene-LOC18440481                                   ...........................................................
Anaoc.0011s0700.v0.9                               ............TGQILS.........................................
Anaoc.0016s1186.v0.9                               IH........VETGRV...........................................
gene-ACMD2_09400                                   ...........................................................
AnM01G09141                                        ...........................................................
AnM01G09144                                        ...........................................................
CHI                                                ...........................................................
gene-AQUCO_01000652v1                              ........QKMENGEPVKIYP......................................
AALP_AA5G189500                                    A.........NEN..............................................
gene-LOC109850014                                  ...........................................................
AVESA.00010b.r2.4AG0615430                         ...........................................................
AVESA.00010b.r2.4DG0758920                         ...........................................................
AVESA.00010b.r2.7CG0710680                         ...........................................................
LXT01106                                           ...........................................................
LXT01112                                           ...........................................................
LXT05481                                           ...........................................................
LXT05485                                           ...........................................................
LXT05486                                           ...........................................................
LXT43699                                           ...........................................................
gene-LOC120080278                                  ...........................................................
BVRB_2g036940                                      VRD....QPDVKA..............................................
BVRB_2g036880                                      ...........................................................
BPChr06G09462.v1.1                                 ...........EKATE...........................................
gene-F511_19977                                    ...........................................................
Bnt03G004203                                       ...........EGKGNKEV........................................
gene-BRADI_1g03840v3                               ...........................................................
Brahy.D01G0043800.v1.1                             ...........................................................
Brahy.S02G0385600.v1.1                             ...........................................................
BjuA09g18460S                                      AE..........EN.............................................
BjuA09g18480S                                      A.........KEN..............................................
BjuB05g27830S                                      ...........................................................
BjuB08g05480S                                      ...........................................................
BnaA07g37900D                                      ...........................................................
BnaA09g34840D                                      A.........KEN..............................................
BnaA09g34850D                                      AE..........EN.............................................
BnaC08g26010D                                      A.........KEN..............................................
BnaC08g26020D                                      AE..........EN.............................................
Bo6g068550                                         A.........KEN..............................................
Bo8g088480                                         A.........KEN..............................................
Bo8g089480                                         AE.........ENKTDP..........................................
C.cajan_29162                                      ...........................................................
Csa02g076590                                       ...........................................................
Csa04g041330                                       ...........................................................
Csa04g041350                                       ...........................................................
Csa06g029680                                       ...........................................................
Csa09g066000                                       ...........................................................
Csa09g066040                                       ...........................................................
Csa09g066060                                       ...........................................................
Csa11g104750                                       ...........................................................
Csa20g023690                                       ...........................................................
maker-Chr6-pred_gff_AUGUSTUS-gene-125.71           ...........................................................
maker-Chr6-pred_gff_AUGUSTUS-gene-220.72           ...........................................................
maker-Chr6-pred_gff_GeneMark.hmm-gene-225.16       ...........................................................
gene_27594                                         AYKDKDVISIPIENGNGKHKVTNE...................................
gene-LOC17885140                                   A.........KGN..............................................
gene-LOC107852750                                  ........PASDQPAI...........................................
gene-FH972_001554                                  ...........................................................
Ct_T15992                                          ...........................................................
Ct_T15993                                          ...........................................................
gene-CIPAW_01G280400                               ...........ENQKLK..........................................
gene-CFOL_v3_20734                                 ...........................................................
gene-KP509_33G035400                               ...........................................................
Cecan.1G005600.V3.1                                ...........................................................
Cecan.2G032100.V3.1                                ...........................................................
Cecan.2G068600.V3.1                                ...........................................................
AUR62020547                                        LSG....QEDIKS..............................................
Cs02g00014                                         ...........................................................
Cs10g00988                                         ...........EGLVFPLLVKPLGSSKMLFLGGAGSRGLEIQGKFVKFTAIGVYLEGDA
gene-LOC101501370                                  ...........................................................
gene-CKAN_00532200                                 ...........................................................
CRGY0216791                                        ...........................................................
Cla97C09G181670                                    ...........................................................
```

**Medicago**	....
**EVM0003632**	....
**gene-Apse012G0133800**	....
**OkoG031217**	....
**Acora.07G100700.v1.1**	....
**CHI**	....
**CHI_2**	....
**gene-QJS04_geneDACA014652**	....
**gene-CEY00_Acc03848**	....
**gene-CEY00_Acc03850**	....
**gene-CEY00_Acc27670**	....
**gene-GOP47_0009997**	....
**EVM0010650**	....
**gene-LOC109764886**	....
**EVM0015959**	....
**gene-LOC18440411**	....
**gene-LOC18440412**	....
**gene-LOC18440481**	....
**Anaoc.0011s0700.v0.9**	....
**Anaoc.0016s1186.v0.9**	....
**gene-ACMD2_09400**	....
**AnM01G09141**	....
**AnM01G09144**	....
**CHI**	....
**gene-AQUCO_01000652v1**	....
**AALP_AA5G189500**	....
**gene-LOC109850014**	....
**AVESA.00010b.r2.4AG0615430**	....
**AVESA.00010b.r2.4DG0758920**	....
**AVESA.00010b.r2.7CG0710680**	....
**LXT01106**	....
**LXT01112**	....
**LXT05481**	....
**LXT05485**	....
**LXT05486**	....
**LXT43699**	....
**gene-LOC120080278**	....
**BVRB_2g036940**	....
**BVRB_2g036880**	....
**BPChr06G09462.v1.1**	....
**gene-F511_19977**	....
**Bnt03G004203**	....
**gene-BRADI_1g03840v3**	....
**Brahy.D01G0043800.v1.1**	....
**Brahy.S02G0385600.v1.1**	....
**BjuA09g18460S**	....
**BjuA09g18480S**	....
**BjuB05g27830S**	....
**BjuB08g05480S**	....
**BnaA07g37900D**	....
**BnaA09g34840D**	....
**BnaA09g34850D**	....
**BnaC08g26010D**	....
**BnaC08g26020D**	....
**Bo6g068550**	....
**Bo8g088480**	....
**Bo8g089480**	....
**C.cajan_29162**	....
**Csa02g076590**	....
**Csa04g041330**	....
**Csa04g041350**	....
**Csa06g029680**	....
**Csa09g066000**	....
**Csa09g066040**	....
**Csa09g066060**	....
**Csa11g104750**	....
**Csa20g023690**	....
**maker-Chr6-pred_gff_AUGUSTUS-gene-125.71**	....
**maker-Chr6-pred_gff_AUGUSTUS-gene-220.72**	....
**maker-Chr6-pred_gff_GeneMark.hmm-gene-225.16**	....
**gene_27594**	....
**gene-LOC17885140**	....
**gene-LOC107852750**	....
**gene-FH972_001554**	....
**Ct_T15992**	....
**Ct_T15993**	....
**gene-CIPAW_01G280400**	....VEN.....
**gene-CFOL_v3_20734**	....
**gene-KP509_33G035400**	....
**Cecan.1G005600.V3.1**	....
**Cecan.2G032100.V3.1**	....
**Cecan.2G068600.V3.1**	....
**AUR62020547**	....
**Cs02g00014**	....
**Cs10g00988**	ISFLAAKWKGKTGKELAVCADFYRDIVAGPFEKFTRVTMILPLTGQQYSEKVTENCVSYW
**gene-LOC101501370**	....
**gene-CKAN_00532200**	....
**CRGY0216791**	....
**Cla97C09G181670**	....

Medicago	....
EVM0003632	....
gene-Apse012G0133800	..............EKPVEL............................DGKDIKVA....
OkoG031217	....
Acora.07G100700.v1.1	....
CHI	....
CHI_2	....
gene-QJS04_geneDACA014652	....
gene-CEY00_Acc03848	....EISV...
gene-CEY00_Acc03850	....
gene-CEY00_Acc27670	....EISA...
gene-GOP47_0009997	....
EVM0010650	....
gene-LOC109764886	....
EVM0015959	....
gene-LOC18440411	....
gene-LOC18440412	....
gene-LOC18440481	....
Anaoc.0011s0700.v0.9	....
Anaoc.0016s1186.v0.9	....
gene-ACMD2_09400	....
AnM01G09141	....
AnM01G09144	....
CHI	....
gene-AQUCO_01000652v1	....LPN......
AALP_AA5G189500	....
gene-LOC109850014	....
AVESA.00010b.r2.4AG0615430	....
AVESA.00010b.r2.4DG0758920	....
AVESA.00010b.r2.7CG0710680	....
LXT01106	....
LXT01112	....
LXT05481	....
LXT05485	....
LXT05486	....
LXT43699	....
gene-LOC120080278	....
BVRB_2g036940	....
BVRB_2g036880	....
BPChr06G09462.v1.1	....
gene-F511_19977	....
Bnt03G004203	....EVKT...
gene-BRADI_1g03840v3	....
Brahy.D01G0043800.v1.1	....
Brahy.S02G0385600.v1.1	....
BjuA09g18460S	....
BjuA09g18480S	....
BjuB05g27830S	....
BjuB08g05480S	....
BnaA07g37900D	....
BnaA09g34840D	....
BnaA09g34850D	....
BnaC08g26010D	....
BnaC08g26020D	....
Bo6g068550	....
Bo8g088480	....
Bo8g089480	....LGDFSWF........
C.cajan_29162	....
Csa02g076590	....
Csa04g041330	....
Csa04g041350	....
Csa06g029680	....
Csa09g066000	....
Csa09g066040	....
Csa09g066060	....
Csa11g104750	....
Csa20g023690	....
maker-Chr6-pred_gff_AUGUSTUS-gene-125.71	....
maker-Chr6-pred_gff_AUGUSTUS-gene-220.72	....
maker-Chr6-pred_gff_GeneMark.hmm-gene-225.16	....
gene_27594	....
gene-LOC17885140	....
gene-LOC107852750	....
gene-FH972_001554	....
Ct_T15992	....
Ct_T15993	....
gene-CIPAW_01G280400	...........QKLEAEIKIL..........................KAGN.SEIGGHK.
gene-CFOL_v3_20734	....
gene-KP509_33G035400	....
Cecan.1G005600.V3.1	....
Cecan.2G032100.V3.1	....
Cecan.2G068600.V3.1	....
AUR62020547	....
Cs02g00014	....
Cs10g00988	KAIGSYTEAEAAAVEKFKEVFKDEIFPPGASILFTQAPSGSLTIGFSKDSSIPEQSAAVI
gene-LOC101501370	....
gene-CKAN_00532200	....
CRGY0216791	....
Cla97C09G181670	....

**Medicago**	....
**EVM0003632**	....
**gene-Apse012G0133800**	....
**OkoG031217**	....
**Acora.07G100700.v1.1**	....
**CHI**	....
**CHI_2**	....
**gene-QJS04_geneDACA014652**	....
**gene-CEY00_Acc03848**	....
**gene-CEY00_Acc03850**	....
**gene-CEY00_Acc27670**	....
**gene-GOP47_0009997**	....
**EVM0010650**	....
**gene-LOC109764886**	....
**EVM0015959**	....
**gene-LOC18440411**	....
**gene-LOC18440412**	....
**gene-LOC18440481**	....
**Anaoc.0011s0700.v0.9**	....
**Anaoc.0016s1186.v0.9**	....
**gene-ACMD2_09400**	....
**AnM01G09141**	....
**AnM01G09144**	....
**CHI**	....
**gene-AQUCO_01000652v1**	....
**AALP_AA5G189500**	....
**gene-LOC109850014**	....
**AVESA.00010b.r2.4AG0615430**	....
**AVESA.00010b.r2.4DG0758920**	....
**AVESA.00010b.r2.7CG0710680**	....
**LXT01106**	....
**LXT01112**	....
**LXT05481**	....
**LXT05485**	....
**LXT05486**	....
**LXT43699**	....
**gene-LOC120080278**	....
**BVRB_2g036940**	....
**BVRB_2g036880**	....
**BPChr06G09462.v1.1**	....
**gene-F511_19977**	....
**Bnt03G004203**	....
**gene-BRADI_1g03840v3**	....
**Brahy.D01G0043800.v1.1**	....
**Brahy.S02G0385600.v1.1**	....
**BjuA09g18460S**	....
**BjuA09g18480S**	....
**BjuB05g27830S**	....
**BjuB08g05480S**	....
**BnaA07g37900D**	....
**BnaA09g34840D**	....
**BnaA09g34850D**	....
**BnaC08g26010D**	....
**BnaC08g26020D**	....
**Bo6g068550**	....
**Bo8g088480**	....
**Bo8g089480**	...............VRALCFYA....................
**C.cajan_29162**	....
**Csa02g076590**	....
**Csa04g041330**	....
**Csa04g041350**	....
**Csa06g029680**	....
**Csa09g066000**	....
**Csa09g066040**	....
**Csa09g066060**	....
**Csa11g104750**	....
**Csa20g023690**	....
**maker-Chr6-pred_gff_AUGUSTUS-gene-125.71**	....
**maker-Chr6-pred_gff_AUGUSTUS-gene-220.72**	....
**maker-Chr6-pred_gff_GeneMark.hmm-gene-225.16**	....
**gene_27594**	....
**gene-LOC17885140**	....
**gene-LOC107852750**	....
**gene-FH972_001554**	....
**Ct_T15992**	....
**Ct_T15993**	....
**gene-CIPAW_01G280400**	....
**gene-CFOL_v3_20734**	....
**gene-KP509_33G035400**	....
**Cecan.1G005600.V3.1**	....
**Cecan.2G032100.V3.1**	....
**Cecan.2G068600.V3.1**	....
**AUR62020547**	....
**Cs02g00014**	....
**Cs10g00988**	ENKQLAEAVLESIIGEHGVSPAAKQSLANRISELLKSDVEKAV
**gene-LOC101501370**	....
**gene-CKAN_00532200**	....
**CRGY0216791**	....
**Cla97C09G181670**	....

```
Medicago                               ............................................................
CICLE_v10032697mg                      ............................................................
gene-COCNU_12G007210                   ............................................................
gene-COCNU_12G007220                   ............................................................
gene-COCNU_12G007230                   ............................................................
gene-GSCOC_T00029776001                ............................................................
gene-IFM89_035037                      ............................................................
gene-IFM89_035040                      ............................................................
gene-CCACVL1_07583                     .....MSTSQSVTG.IQVENVAFPPTVKPPGSTKTLFLGGAGERGLEI..QGKFIKFTAI
Cav07g20270                            ............................................................
Cav07g20290                            ............................................................
gene-BT93_F1001                        ............................................................
MELO3C025484.2                         ............................................................
Csa_5G505170                           ............................................................
gene-DM860_013525                      .....M......................................................
geneJL025816                           ............................................................
geneJL015184                           ............................................................
CHI_2                                  ............................................................
Dinv31395                              ............................................................
CHI                                    ............................................................
CHI_2                                  ............................................................
CHI_3                                  ............................................................
gene-KFK09_000997                      ............................................................
gene-BAE44_0007788                     ............................................................
gene-BAE44_0020983                     ............................................................
Dexi3A01G0013540                       ............................................................
Dexi3B01G0013780                       ............................................................
D.long004984                           ............................................................
gene-IHE45_09G071500                   ............................................................
DRNTG_23933                            ............................................................
gene-J5N97_002300                      ............................................................
gene-LOC111309827                      ............................................................
scaffold13.535                         ............................................................
scaffold176.249                        ............................................................
scaffold36.570                         ............................................................
scaffold3.658                          ............................................................
scaffold65.276                         ............................................................
gene-LOC105035984                      ............................................................
gene-PR202_ga11488                     ............................................................
gene-PR202_ga13968                     ............................................................
gene-PR202_gb10465                     ............................................................
gene-EJB05_03819                       ............................................................
Et_4A_034194                           ............................................................
Et_4B_038342                           ............................................................
Et_6A_046252                           ............................................................
Et_9A_062905                           ............................................................
Et_9B_065446                           ............................................................
EUGRSUZ_F03816                         ............................................................
EVM20prediction20Chr12.1151            ............................................................
Casp15914                              ............................................................
EUTSA_v10004922mg                      ............................................................
EUTSA_v10010658mg                      ............................................................
EVM_prediction_Fe1.6811                ............................................................
EVM_prediction_Fe3.10426               ............................................................
EVM_prediction_Fe4.5584                ............................................................
EVM_prediction_Fe5.14515               ............................................................
EVM_prediction_Fe5.14516               ............................................................
FCD_00011569                           ............................................................
FCD_00011572                           ............................................................
FCD_00011567                           ............................................................
FCD_00011574                           ............................................................
maker-Fvb7-2-augustus-gene-221.49      ............................................................
maker-Fvb7-3-augustus-gene-78.40       ............................................................
EVM20prediction20scaffold_9.28         ............................................................
gene-GLYMA_20G241700v4                 ............................................................
TnS000078355t12                        MEDEQICLELEQLPGVQVEHIEFPETHKAVGTLKPTLLLGAGVRGLPVGPGGAFVSFTAI
TnS000589875t03                        ............................................................
gene-EPI10_010037                      ............................................................
GG16G068580                            ............................................................
GG16G068530                            ............................................................
gene-CDL12_22415                       ............................................................
gene-CDL12_26251                       ............................................................
gene-LOC110910959                      ............................................................
gene-LOC110913037                      ............................................................
gene-LOC110913039                      ............................................................
gene-LOC110425216                      ............................................................
gene-P3X46_014162                      ............................................................
gene-ZWY2020_018680                    ............................................................
Hyque.04G056200.v1.1                   ............................................................
gene-LOC109186979                      ............................................................
itb05g04360                            ............................................................
gene-M6B38_184735                      ............................................................
gene-M6B38_299855                      ............................................................
gene-M6B38_364490                      ............................................................
gene-M6B38_399615                      ............................................................
Jmimo22322                             ............................................................
HT24166                                ............................................................
Joasc.10G103600.v1.1                   .....MNVVAWLK................................LQL............
Jr07_35750                             ............................................................
```

```
Medicago                              ............................................................
CICLE_v10032697mg                     ............................................................
gene-COCNU_12G007210                  ............................................................
gene-COCNU_12G007220                  ............................................................
gene-COCNU_12G007230                  ............................................................
gene-GSCOC_T00029776001               ............................................................
gene-IFM89_035037                     ............................................................
gene-IFM89_035040                     ............................................................
gene-CCACVL1_07583                    GVYLEGNAVECLATKWKG.KSDEELTESVEFFKDIVTGAFEKFIRVTMILPLTGQQYSEK
Cav07g20270                           ............................................................
Cav07g20290                           ............................................................
gene-BT93_F1001                       ............................................................
MELO3C025484.2                        ............................................................
Csa_5G505170                          ............................................................
gene-DM860_013525                     ............................................................
geneJL025816                          ............................................................
geneJL015184                          ............................................................
CHI_2                                 ............................................................
Dinv31395                             ............................................................
CHI                                   ............................................................
CHI_2                                 ............................................................
CHI_3                                 ............................................................
gene-KFK09_000997                     ............................................................
gene-BAE44_0007788                    ............................................................
gene-BAE44_0020983                    ............................................................
Dexi3A01G0013540                      ............................................................
Dexi3B01G0013780                      ............................................................
D.long004984                          ............................................................
gene-IHE45_09G071500                  ............................................................
DRNTG_23933                           ............................................................
gene-J5N97_002300                     ............................................................
gene-LOC111309827                     ............................................................
scaffold13.535                        ............................................................
scaffold176.249                       ............................................................
scaffold36.570                        ............................................................
scaffold3.658                         ............................................................
scaffold65.276                        ............................................................
gene-LOC105035984                     ............................................................
gene-PR202_ga11488                    ............................................................
gene-PR202_ga13968                    ............................................................
gene-PR202_gb10465                    ............................................................
gene-EJB05_03819                      ............................................................
Et_4A_034194                          ............................................................
Et_4B_038342                          ............................................................
Et_6A_046252                          ............................................................
Et_9A_062905                          ............................................................
Et_9B_065446                          ............................................................
EUGRSUZ_F03816                        ............................................................
EVM20prediction20Chr12.1151           ............................................................
Casp15914                             ............................................................
EUTSA_v10004922mg                     ............................................................
EUTSA_v10010658mg                     ............................................................
EVM_prediction_Fe1.6811               ............................................................
EVM_prediction_Fe3.10426              ............................................................
EVM_prediction_Fe4.5584               ............................................................
EVM_prediction_Fe5.14515              ............................................................
EVM_prediction_Fe5.14516              ............................................................
FCD_00011569                          ............................................................
FCD_00011572                          ............................................................
FCD_00011567                          ............................................................
FCD_00011574                          ............................................................
maker-Fvb7-2-augustus-gene-221.49     ............................................................
maker-Fvb7-3-augustus-gene-78.40      ............................................................
EVM20prediction20scaffold_9.28        ............................................................
gene-GLYMA_20G241700v4                ............................................................
TnS000078355t12                       AVYAEPSAIPYLRSRWGTCSTSQELLTNLDFFMDIITSPMEKLLKVVMLKPLSGAQYSGK
TnS000589875t03                       ............................................................
gene-EPI10_010037                     ............................................................
GG16G068580                           ............................................................
GG16G068530                           ............................................................
gene-CDL12_22415                      ............................................................
gene-CDL12_26251                      ............................................................
gene-LOC110910959                     ............................................................
gene-LOC110913037                     ............................................................
gene-LOC110913039                     ............................................................
gene-LOC110425216                     ............................................................
gene-P3X46_014162                     ............................................................
gene-ZWY2020_018680                   ............................................................
Hyque.04G056200.v1.1                  ............................................................
gene-LOC109186979                     ............................................................
itb05g04360                           ............................................................
gene-M6B38_184735                     ............................................................
gene-M6B38_299855                     ............................................................
gene-M6B38_364490                     ............................................................
gene-M6B38_399615                     ............................................................
Jmimo22322                            ............................................................
HT24166                               ............................................................
Joasc.10G103600.v1.1                  ...................RT.......................................
Jr07_35750                            ............................................................
```

```
Medicago                             ............................................................
CICLE_v10032697mg                    ............................................................
gene-COCNU_12G007210                 ............................................................
gene-COCNU_12G007220                 ............................................................
gene-COCNU_12G007230                 ............................................................
gene-GSCOC_T00029776001              ............................................................
gene-IFM89_035037                    ............................................................
gene-IFM89_035040                    ............................................................
gene-CCACVL1_07583                   VAENCVAIWKSLGLYTDEEAKAIEKFVDVFKNENFPL..GSSILFTISAEGSLTIGFSKD
Cav07g20270                          ............................................................
Cav07g20290                          ............................................................
gene-BT93_F1001                      ............................................................
MELO3C025484.2                       ............................................................
Csa_5G505170                         ............................................................
gene-DM860_013525                    ...................................PPLI.....................
geneJL025816                         ............................................................
geneJL015184                         ............................................................
CHI_2                                ............................................................
Dinv31395                            ............................................................
CHI                                  ............................................................
CHI_2                                ............................................................
CHI_3                                ............................................................
gene-KFK09_000997                    ............................................................
gene-BAE44_0007788                   ............................................................
gene-BAE44_0020983                   ............................................................
Dexi3A01G0013540                     ............................................................
Dexi3B01G0013780                     ............................................................
D.long004984                         ............................................................
gene-IHE45_09G071500                 ............................................................
DRNTG_23933                          ............................................................
gene-J5N97_002300                    ............................................................
gene-LOC111309827                    ............................................................
scaffold13.535                       ............................................................
scaffold176.249                      ............................................................
scaffold36.570                       ............................................................
scaffold3.658                        ............................................................
scaffold65.276                       ............................................................
gene-LOC105035984                    ............................................................
gene-PR202_ga11488                   ............................................................
gene-PR202_ga13968                   ............................................................
gene-PR202_gb10465                   ............................................................
gene-EJB05_03819                     ............................................................
Et_4A_034194                         ............................................................
Et_4B_038342                         ............................................................
Et_6A_046252                         ............................................................
Et_9A_062905                         ............................................................
Et_9B_065446                         ............................................................
EUGRSUZ_F03816                       ............................................................
EVM20prediction20Chr12.1151          ............................................................
Casp15914                            ............................................................
EUTSA_v10004922mg                    ............................................................
EUTSA_v10010658mg                    ............................................................
EVM_prediction_Fe1.6811              ............................................................
EVM_prediction_Fe3.10426             ............................................................
EVM_prediction_Fe4.5584              ............................................................
EVM_prediction_Fe5.14515             ............................................................
EVM_prediction_Fe5.14516             ............................................................
FCD_00011569                         ............................................................
FCD_00011572                         ............................................................
FCD_00011567                         ............................................................
FCD_00011574                         ............................................................
maker-Fvb7-2-augustus-gene-221.49    ............................................................
maker-Fvb7-3-augustus-gene-78.40     ............................................................
EVM20prediction20scaffold_9.28       ............................................................
gene-GLYMA_20G241700v4               ............................................................
TnS000078355t12                      VAEGCLSLWKEAGIQ.EDNNRALEEFKNAF..............................
TnS000589875t03                      ............................................................
gene-EPI10_010037                    ............................................................
GG16G068580                          ............................................................
GG16G068530                          ............................................................
gene-CDL12_22415                     ............................................................
gene-CDL12_26251                     ............................................................
gene-LOC110910959                    ............................................................
gene-LOC110913037                    ............................................................
gene-LOC110913039                    ............................................................
gene-LOC110425216                    ............................................................
gene-P3X46_014162                    ............................................................
gene-ZWY2020_018680                  ............................................................
Hyque.04G056200.v1.1                 ............................................................
gene-LOC109186979                    ............................................................
itb05g04360                          ............................................................
gene-M6B38_184735                    ............................................................
gene-M6B38_299855                    ............................................................
gene-M6B38_364490                    ............................................................
gene-M6B38_399615                    ............................................................
Jmimo22322                           ............................................................
HT24166                              ............................................................
Joasc.10G103600.v1.1                 .......................................SGLLLLGRT..GGL.......
Jr07_35750                           ............................................................
```

**Medicago**	....
**CICLE_v10032697mg**	....
**gene-COCNU_12G007210**	....
**gene-COCNU_12G007220**	....
**gene-COCNU_12G007230**	....
**gene-GSCOC_T00029776001**	....
**gene-IFM89_035037**	....
**gene-IFM89_035040**	....
**gene-CCACVL1_07583**	SSVPEVGTAVIENKLLANSVLESIIGKAGVSPAAKQSLASRLPALFNDSTNQIALMGNES
**Cav07g20270**	....
**Cav07g20290**	....
**gene-BT93_F1001**	....
**MELO3C025484.2**	....
**Csa_5G505170**	....
**gene-DM860_013525**	....N
**geneJL025816**	....
**geneJL015184**	....
**CHI_2**	....
**Dinv31395**	....
**CHI**	....
**CHI_2**	....
**CHI_3**	....
**gene-KFK09_000997**	....
**gene-BAE44_0007788**	....
**gene-BAE44_0020983**	....
**Dexi3A01G0013540**	....
**Dexi3B01G0013780**	....
**D.long004984**	....
**gene-IHE45_09G071500**	....
**DRNTG_23933**	....
**gene-J5N97_002300**	....
**gene-LOC111309827**	....
**scaffold13.535**	....
**scaffold176.249**	....
**scaffold36.570**	....
**scaffold3.658**	....
**scaffold65.276**	....
**gene-LOC105035984**	....
**gene-PR202_ga11488**	....
**gene-PR202_ga13968**	....
**gene-PR202_gb10465**	....
**gene-EJB05_03819**	....
**Et_4A_034194**	....
**Et_4B_038342**	....
**Et_6A_046252**	....
**Et_9A_062905**	....
**Et_9B_065446**	....
**EUGRSUZ_F03816**	....
**EVM20prediction20Chr12.1151**	....
**Casp15914**	....
**EUTSA_v10004922mg**	....
**EUTSA_v10010658mg**	....
**EVM_prediction_Fe1.6811**	....
**EVM_prediction_Fe3.10426**	....
**EVM_prediction_Fe4.5584**	....
**EVM_prediction_Fe5.14515**	....
**EVM_prediction_Fe5.14516**	....
**FCD_00011569**	....
**FCD_00011572**	....
**FCD_00011567**	....
**FCD_00011574**	....
**maker-Fvb7-2-augustus-gene-221.49**	....
**maker-Fvb7-3-augustus-gene-78.40**	....
**EVM20prediction20scaffold_9.28**	....
**gene-GLYMA_20G241700v4**	....
**TnS000078355t12**	....
**TnS000589875t03**	....
**gene-EPI10_010037**	....
**GG16G068580**	....
**GG16G068530**	....
**gene-CDL12_22415**	....
**gene-CDL12_26251**	....
**gene-LOC110910959**	....
**gene-LOC110913037**	....
**gene-LOC110913039**	....
**gene-LOC110425216**	....
**gene-P3X46_014162**	....
**gene-ZWY2020_018680**	....
**Hyque.04G056200.v1.1**	....
**gene-LOC109186979**	....
**itb05g04360**	....
**gene-M6B38_184735**	....
**gene-M6B38_299855**	....
**gene-M6B38_364490**	....
**gene-M6B38_399615**	....
**Jmimo22322**	....
**HT24166**	....
**Joasc.10G103600.v1.1**	....RVTS
**Jr07_35750**	....

```
                                                         1
Medicago                                 .............M..............................................
CICLE_v10032697mg                        ..........MN.P..............................................
gene-COCNU_12G007210                     ............................................................
gene-COCNU_12G007220                     ............................................................
gene-COCNU_12G007230                     ..........MG.GG.............................................
gene-GSCOC_T00029776001                  ..........MS.L..............................................
gene-IFM89_035037                        ............................................................
gene-IFM89_035040                        ..........MA................................................
gene-CCACVL1_07583                       SSSF......MS................................................
Cav07g20270                              ..........MV.S..............................................
Cav07g20290                              ..........MV.S..............................................
gene-BT93_F1001                          ..........MA.PP.............................................
MELO3C025484.2                           ..........MP.P..............................................
Csa_5G505170                             ............................................................
gene-DM860_013525                        HTALIDKKA.MS.A..............................................
geneJL025816                             ..........MA................................................
geneJL015184                             ..........MA................................................
CHI_2                                    ..........MS................................................
Dinv31395                                ..........MS.P..............................................
CHI                                      ............................................................
CHI_2                                    ............................................................
CHI_3                                    ............................................................
gene-KFK09_000997                        ..........MA.E..............................................
gene-BAE44_0007788                       ............................................................
gene-BAE44_0020983                       ..........MA................................................
Dexi3A01G0013540                         ..........MA................................................
Dexi3B01G0013780                         ..........MA................................................
D.long004984                             ..........M.................................................
gene-IHE45_09G071500                     ..........MG.EA.............................................
DRNTG_23933                              ..........MG.EA.............................................
gene-J5N97_002300                        ..........MG.DK.............................................
gene-LOC111309827                        ..........MS.T..............................................
scaffold13.535                           ..........MA................................................
scaffold176.249                          ............................................................
scaffold36.570                           ..........MA................................................
scaffold3.658                            ............................................................
scaffold65.276                           ............................................................
gene-LOC105035984                        ..........MG.EG.............................................
gene-PR202_ga11488                       ..........MA................................................
gene-PR202_ga13968                       ............................................................
gene-PR202_gb10465                       ............................................................
gene-EJB05_03819                         ............................................................
Et_4A_034194                             ............................................................
Et_4B_038342                             ............................................................
Et_6A_046252                             ............................................................
Et_9A_062905                             ..........MA................................................
Et_9B_065446                             ..........MA................................................
EUGRSUZ_F03816                           ..........MA.PP.............................................
EVM20prediction20Chr12.1151              ..........MA.L..............................................
Casp15914                                ..........MA................................................
EUTSA_v10004922mg                        ..........MP................................................
EUTSA_v10010658mg                        ...MSSSAC.PS.P..............................................
EVM_prediction_Fe1.6811                  ..........MI................................................
EVM_prediction_Fe3.10426                 ..........ME................................................
EVM_prediction_Fe4.5584                  ............................................................
EVM_prediction_Fe5.14515                 ..........MA.S..............................................
EVM_prediction_Fe5.14516                 ..........MA.S..............................................
FCD_00011569                             ..........MS................................................
FCD_00011572                             ..........MS................................................
FCD_00011567                             ..........MV................................................
FCD_00011574                             ..........MP................................................
maker-Fvb7-2-augustus-gene-221.49        ..........MA................................................
maker-Fvb7-3-augustus-gene-78.40         ..........MA................................................
EVM20prediction20scaffold_9.28           ............................................................
gene-GLYMA_20G241700v4                   ..........MAMA..............................................
TnS000078355t12                          ......SDRNMA.PGSSILFNVSPAGLLIGFSAGNSVPERHEVAEIKNRALAEAFSATII
TnS000589875t03                          ..........ME...................................NRV..........
gene-EPI10_010037                        ..........MS.T..............................................
GG16G068580                              ..........MS.SS.............................................
GG16G068530                              ..........MS.SS.............................................
gene-CDL12_22415                         ..........MS................................................
gene-CDL12_26251                         ..........MS.S..............................................
gene-LOC110910959                        ..........MS.K..............................................
gene-LOC110913037                        ..........M.................................................
gene-LOC110913039                        ..........MA.K..............................................
gene-LOC110425216                        ..........MS.T..............................................
gene-P3X46_014162                        ..........MS.P..............................................
gene-ZWY2020_018680                      ............................................................
Hyque.04G056200.v1.1                     .......MS.SS.P..............................................
gene-LOC109186979                        ..........MS.A..............................................
itb05g04360                              ..........MS.A..............................................
gene-M6B38_184735                        ..........MS.AQYM...........................................
gene-M6B38_299855                        ..........MG.EV.............................................
gene-M6B38_364490                        ..........MS.AQYM...........................................
gene-M6B38_399615                        ..........MG.EV.............................................
Jmimo22322                               ..........MS.A..............................................
HT24166                                  ..........MS.S..............................................
Joasc.10G103600.v1.1                     KNAVCISCS.LALTLRCTYTY..................................QPP..
Jr07_35750                               ..........MV.P..............................................
```

```
                                                                                        10
Medicago                                  ......................AA.S.............................ITAITV
CICLE_v10032697mg                         ......................SP.S.............................VTELQV
gene-COCNU_12G007210                      .............................................................
gene-COCNU_12G007220                      .............................................................
gene-COCNU_12G007230                      ......................AA.A.............................LPKLEV
gene-GSCOC_T00029776001                   ......................SL.S.............................VGEVHV
gene-IFM89_035037                         .............................................................
gene-IFM89_035040                         .....................QLQ.K.............................ISEVKV
gene-CCACVL1_07583                        ....FRPI............HMLP.S.............................VAAIQV
Cav07g20270                               ......................PL.S.............................STGVQV
Cav07g20290                               ......................PL.P.............................STGVQV
gene-BT93_F1001                           .....................PPV.A.............................VSEVQV
MELO3C025484.2                            ......................VP.D.............................LPGLQV
Csa_5G505170                              ......................MA.R.............................LGGLQV
gene-DM860_013525                         ......................PP.S.............................VTEIEV
geneJL025816                              ......................ET.P.............................ATPVEV
geneJL015184                              ......................ET.P.............................ATPVEV
CHI_2                                     .......................P.S.............................VTEIQV
Dinv31395                                 ......................SL.S.............................VTEVQV
CHI                                       .............................................................
CHI_2                                     .............................................................
CHI_3                                     .............................................................
gene-KFK09_000997                         ......................TP.A.............................AIVVEV
gene-BAE44_0007788                        .............................................................
gene-BAE44_0020983                        .........................V.............................SSEVTV
Dexi3A01G0013540                          .........................V............................SSSELTV
Dexi3B01G0013780                          .........................V............................SSSELTV
D.long004984                              ......................SP.N.............................VTEVQV
gene-IHE45_09G071500                      .....................TEK.I.............................SPELEI
DRNTG_23933                               .....................TEK.I.............................TPELEI
gene-J5N97_002300                         .....................AAS.I.............................APELEV
gene-LOC111309827                         ......................SP.S.............................VTEIQV
scaffold13.535                            .........................A.............................SPEVTV
scaffold176.249                           .......................M.A.............................VPELAV
scaffold36.570                            .........................A.............................SPEVTV
scaffold3.658                             .......................M.A.............................VSELAV
scaffold65.276                            .......................M.A.............................VSGLTV
gene-LOC105035984                         ......................AA.L.............................LPKLEV
gene-PR202_ga11488                        .........................M.............................LEEVIV
gene-PR202_ga13968                        ......................MA.A.............................VSEVAV
gene-PR202_gb10465                        .......................M.T.............................VSEVAV
gene-EJB05_03819                          .......................M.A.............................VSEVAV
Et_4A_034194                              .......................M.A.............................VSEVAV
Et_4B_038342                              .......................M.A.............................GSEVAV
Et_6A_046252                              .......................M.A.............................LSEVAV
Et_9A_062905                              .......................................................LPEVVV
Et_9B_065446                              .......................................................LPEVVV
EUGRSUZ_F03816                            .....................PSV.P.............................VSEVQV
EVM20prediction20Chr12.1151               ......................SP.S.............................VTEIQV
Casp15914                                 ......................SA.V.............................TTPITV
EUTSA_v10004922mg                         ......................LP.S.............................VTPIHV
EUTSA_v10010658mg                         ......................LP.S.............................VTKLQV
EVM_prediction_Fe1.6811                   .........................................................RLLL
EVM_prediction_Fe3.10426                  ......................RPRK..........................D.FLT....
EVM_prediction_Fe4.5584                   .............................................................
EVM_prediction_Fe5.14515                  ......................SI.T.............................VSSVAV
EVM_prediction_Fe5.14516                  ......................SI.T.............................VSSIAI
FCD_00011569                              ......................SM.T.............................LTGVQV
FCD_00011572                              ......................PM.T.............................LTGVQV
FCD_00011567                              .........................A.............................AA....
FCD_00011574                              ......................PM.T.............................LTGVQV
maker-Fvb7-2-augustus-gene-221.49         .......................Q.S.............................VTGIQI
maker-Fvb7-3-augustus-gene-78.40          .......................Q.S.............................VTGIQI
EVM20prediction20scaffold_9.28            .......................................................MNAVKV
gene-GLYMA_20G241700v4                    ......................FP.S.............................VTSVTV
TnS000078355t12                           GKSAVSPQTKASIADRFFNL.YLP.AVAKTGAAD....................QLTEMEV
TnS000589875t03                           .....................RLP...........LWTLLALWAGFGGVNKYEWHLGELVL
gene-EPI10_010037                         ......................SP.S.............................VTELQV
GG16G068580                               .....................SKS.S.............................LTEVHV
GG16G068530                               .....................SSS.S.............................VTEVQV
gene-CDL12_22415                          ......................PP.S.............................VTEVQV
gene-CDL12_26251                          ......................LP.S.............................VTEVQV
gene-LOC110910959                         ......................PN.S.............................TTSLQV
gene-LOC110913037                         .........................A.............................TTGLQV
gene-LOC110913039                         ......................PQ.S.............................SMGLQV
gene-LOC110425216                         ......................SP.C.............................VAGIQV
gene-P3X46_014162                         .....................AAT.S.............................LTHINV
gene-ZWY2020_018680                       .......................M.A.............................VSELDV
Hyque.04G056200.v1.1                      ......................SP.A.............................VTEIQV
gene-LOC109186979                         ......................PP.C.............................VAEVKV
itb05g04360                               ......................PP.C.............................VAEVKV
gene-M6B38_184735                         .....................SAL.V.............................LTKLVI
gene-M6B38_299855                         .....................SPV.P.............................VTELEV
gene-M6B38_364490                         .....................SAL.V.............................LTKLVI
gene-M6B38_399615                         .....................SPV.P.............................VTELEV
Jmimo22322                                ......................PP.L.............................VTELQI
HT24166                                   ......................SP.T.............................VTPLKV
Joasc.10G103600.v1.1                      ....YNSVQLQPLLFSSRVY.AGM.A.............................VSELEV
Jr07_35750                                ......................AP.S.............................LPVVHV
```

```
                                                                          20                  30
                                                                           .                   .
Medicago                            ENLEY.P...A.......VV.TSP.V...T.G...KS.YFL.GGAG..............
CICLE_v10032697mg                   ENVTF.T...P.......SV.QPP.G...S.T...KS.HFL.GGAG..............
gene-COCNU_12G007210                ............................................................
gene-COCNU_12G007220                ............................................................
gene-COCNU_12G007230                EGIVF.P...P.......VV.SPP.G...S.S...KT.LFL.GGAG..............
gene-GSCOC_T00029776001             DGHVF.P...P.......AAKKPP.G...S.D...QN.FFL.GGAG..............
gene-IFM89_035037                   ............................................................
gene-IFM89_035040                   GDYTF.S...P.......KLTNAL.G...S.T...NT.FFL.GGAG..............
gene-CCACVL1_07583                  ENVTF.P...S.......TV.KPP.G...S.T...KT.LFL.GGAG..............
Cav07g20270                         ENLEF.P...P.......AA.KPP.G...S.T...NT.LFL.GGAG..............
Cav07g20290                         ENHEF.P...P.......AA.KPP.G...S.T...NT.LFL.GGAG..............
gene-BT93_F1001                     EFVKF.P...P.......SV.KPP.D...S.A...KT.LFL.GGAG..............
MELO3C025484.2                      ENLQF.P...P.......EI.KPP.A...S.S...KT.LFL.GGAG..............
Csa_5G505170                        ETVNF.P...A.......EI.KPP.A...S.A...NT.LFL.GGAG..............
gene-DM860_013525                   EGNVF.P...A.......TV.TPP.G...S.A...KA.FIL.GGAG..............
geneJL025816                        EGVTF.P...A.......EF.TSP.A...T.S...KP.LFL.GGAG..............
geneJL015184                        EGVTF.P...A.......EF.TSP.A...T.S...KP.L.....................
CHI_2                               ENHLF.P...A.......TV.KPP.A...T.C...HT.LFL.AGAG..............
Dinv31395                           ESHVF.P...P.......TV.KPP.G...T.T...KT.LFL.GGSG..............
CHI                                 ............................................................
CHI_2                               ............................................................
CHI_3                               ............................................................
gene-KFK09_000997                   EGVKF.P...T.......EV.TSP.V...T.S...KS.LFL.GGAG..............
gene-BAE44_0007788                  ............................................................
gene-BAE44_0020983                  EGVVF.P...P.......VA.RPP.G...S.V...RA.HFL.AGGG..............
Dexi3A01G0013540                    EGIVF.P...P.......LI.RPP.G...S.V...RS.HFL.AGGG..............
Dexi3B01G0013780                    EGIVF.P...P.......VI.RPP.G...S.D...RS.HFL.AGGG..............
D.long004984                        ENVTF.S...P.......SV.KPP.G...S.T...KL.HFL.GGAG..............
gene-IHE45_09G071500                EGFVF.P...A.......EV.SIP.G...F.S...EP.LFL.GGAG..............
DRNTG_23933                         EGFVF.P...A.......EV.SIP.G...F.S...EP.LFL.GGAG..............
gene-J5N97_002300                   EGFVF.P...A.......VA.SIP.G...S.S...ES.LLL.GGAG..............
gene-LOC111309827                   ENVTF.P...P.......TI.KPP.G...S.T...KT.LFL.GGAG..............
scaffold13.535                      EGVVF.P...P.......VA.RPP.G...S.V...RT.HFL.AGAG..............
scaffold176.249                     DGVVF.P...P.......VA.RPP.G...S.G...NS.HFL.AGAG..............
scaffold36.570                      EGVVF.P...P.......VA.RPP.G...S.V...RT.HFL.AGAG..............
scaffold3.658                       DGVVF.P...P.......VA.RPP.G...S.G...NS.HFL.AGAG..............
scaffold65.276                      EGVVF.P...PA......VA.RPP.G...S.G...NS.HFL.AGAG..............
gene-LOC105035984                   EGIVF.P...P.......VV.SPP.G...S.S...KT.LFL.GGAG..............
gene-PR202_ga11488                  EGVVF.P...P.......VV.RPP.G...S.N...RT.HFL.AGAG..............
gene-PR202_ga13968                  DGVVF.P...PA......VA.RPP.G...S.G...RS.HLL.AGAG..............
gene-PR202_gb10465                  DGVVF.P...PA......VA.RPP.G...S.G...RS.HLL.AGAG..............
gene-EJB05_03819                    DGVVF.P...P.......VA.RPP.G...S.G...RQ.HFL.AGAG..............
Et_4A_034194                        DGVVF.P...P.......VV.RPP.G...S.G...RS.HFL.AGAG..............
Et_4B_038342                        DGVVF.P...P.......VV.RPP.G...S.G...RS.HFL.AGAG..............
Et_6A_046252                        DGVVF.T...P.......VS.RPP.G...S.S...HS.HFL.AGAG..............
Et_9A_062905                        EGVVF.P...P.......VV.CPP.G...S.G...RS.HFL.AGAG..............
Et_9B_065446                        EGVVF.P...P.......VA.RPP.G...S.G...RT.HFL.AGAG..............
EUGRSUZ_F03816                      ESVKF.P...P.......SI.KPP.G...S.A...KI.LFL.GGAG..............
EVM20prediction20Chr12.1151         DSRVF.S...P.......TV.KPP.G...I.E...KA.FFL.AGAG..............
Casp15914                           ETVSF.S...P.......AV.KPP.A...S.N...KS.LFL.AGAG..............
EUTSA_v10004922mg                   GSFTF.P...P.......AI.TSP.A...S.S...KK.LFL.GGA...............
EUTSA_v10010658mg                   DSVTF.P...P.......SV.NSP.A...S.S...NP.LFL.GGAG..............
EVM_prediction_Fe1.6811             ......P..SPVRVRLLAAV.........S.......HLFH....G..............
EVM_prediction_Fe3.10426            GSVAFRPGR............RPP..LVPSPDLPPRS.......AGRRPSCTLPPPLSTV
EVM_prediction_Fe4.5584             ............................................................
EVM_prediction_Fe5.14515            EDFVF.P...P.......YV.RPP.A...T.E...KS.FFL.GGAG..............
EVM_prediction_Fe5.14516            EDFVF.P...P.......SV.RPP.A...T.E...KS.FFL.GGAG..............
FCD_00011569                        EKAAF.P...P.......TV.TPP.G...S.A...KT.LFL.GGAG..............
FCD_00011572                        EKAAF.P...P.......TV.TPP.G...S.A...KT.LFL.GGAG..............
FCD_00011567                        ..........S.......SL.TPTRG.........RN....RGISA..............
FCD_00011574                        ETAAF.A...P.......AV.TPP.G...S.A...KT.LFL.GGAG..............
maker-Fvb7-2-augustus-gene-221.49   GGTSF.P...P.......AV.KPP.G...S.G...NT.FFL.GGAG..............
maker-Fvb7-3-augustus-gene-78.40    GGTSF.P...P.......AV.KPP.G...S.G...NT.LFL.GGSG..............
EVM20prediction20scaffold_9.28      ENVTF.L...A.......EV.TSP.A...T.S...KP.VFL.GGAG..............
gene-GLYMA_20G241700v4              ENVTF.P...P.......TV.KPP.C...S.P...NT.FFL.AGAG..............
TnS000078355t12                     EGNLF.L...R.......SV.KAV.E...S.E...KE.LLL.AGAG..............
TnS000589875t03                     EGVLF.P...A.......SM.DFV.G...S.N...ET.LLL.AGAG..............
gene-EPI10_010037                   ENFTF.P...P.......TV.KPP.G...S.T...KT.LFL.GGAG..............
GG16G068580                         DAVKF.P...P.......AV.KPP.A...S.S...KT.LFL.GGAG..............
GG16G068530                         DAVKF.P...P.......AV.KPP.A...S.S...KT.LFL.GGTG..............
gene-CDL12_22415                    ESFVF.P...P.......AV.KPP.G...S.I...KT.FFL.GGAG..............
gene-CDL12_26251                    DSFVF.P...P.......AV.KPP.G...S.I...KT.FFL.AGAG..............
gene-LOC110910959                   ESIVF.P...P.......SA.KPP.G...A.T...NT.LFL.GGAG..............
gene-LOC110913037                   ESILF.P...P.......SV.KPP.G...A.S...AT.FFL.GGAG..............
gene-LOC110913039                   ESIVF.P...P.......SV.KPP.G...A.T...ST.FFL.GGAG..............
gene-LOC110425216                   ENVTF.P...P.......NV.KPP.G...S.T...KT.LFL.GGAG..............
gene-P3X46_014162                   ENIAF.S...P.......AV.KPP.A...S.N...KT.LFL.GGAG..............
gene-ZWY2020_018680                 DGVVF.P...P.......LA.RPP.G...S.A...HA.HFL.AGAG..............
Hyque.04G056200.v1.1                EKYVF.P...A.......AV.KPP.G...T.T...KT.LFL.GGAG..............
gene-LOC109186979                   ESYVF.P...A.......TV.KPP.G...T.A...KT.LIL.GGAG..............
itb05g04360                         ESYVF.P...A.......TA.KPP.G...T.A...KT.LIL.GGAG..............
gene-M6B38_184735                   AGFLF.P...A.......TI.TSP.G...S.S..NTT.LFL.GGAG..............
gene-M6B38_299855                   DGVLF.P...P.......VL.TPP.G...S.S...KA.HFL.GGAG..............
gene-M6B38_364490                   AGFLF.P...A.......TI.TSP.G...S.S..NTT.LFL.GGAG..............
gene-M6B38_399615                   DGVLF.P...P.......VL.TPP.G...S.S...KA.HFL.GGAG..............
Jmimo22322                          ESYVF.P...P.......AL.KPP.G...S.A...KT.FFL.GGAG..............
HT24166                             DSYLF.P...A.......AV.KPP.G...S.T...NT.FFL.GGAG..............
Joasc.10G103600.v1.1                EGIVF.P...Q.......KT.RPP.G...S.G...GT.LFL.AGAG..............
Jr07_35750                          DNVAF.Q...P.......TT.KVP.G...S.D...KA.LFL.GGAG..............
```

```
                                                40        50        60        70        80
Medicago                           ....ERGLTIEG.NFI.KFTAI.GVYL.ED..IA.VASLA.AKWK.GKSSEELLETLDFY
CICLE_v10032697mg                  ....ERGLEIEG.KFV.KFTAI.GVYL.ED..DA.VPLLA.GKWK.GKTAEELTESVEFF
gene-COCNU_12G007210               ............................................................
gene-COCNU_12G007220               ............................................................
gene-COCNU_12G007230               ....CRGLEIGG.RFI.TFTAI.GIYL.ED..EA.VRSLS.EKWK.GKTADELAASHDFF
gene-GSCOC_T00029776001            ....ARGLEIEG.KFI.KFTAI.GVYL.EE..TA.IPSLA.VKWK.GKTAEELTESVEFF
gene-IFM89_035037                  ............................................................
gene-IFM89_035040                  ....VRGLQIQD.KFI.KFTAI.GIYLEEE..KA.VPLLA.SKWK.GKTADKLTDSVEFF
gene-CCACVL1_07583                 ....ERGLEIQG.KFI.KFTAI.GVYL.ED..NA.VECLG.VKWK.GKSTEELAESIEFF
Cav07g20270                        ....ARGLEIQG.KLV.KFTAI.GVYL.ED..TA.VPSLS.VKWK.GKSREELTESVEFF
Cav07g20290                        ....ARGLEIQG.KLV.KFTAI.GVYL.ED..TA.VASLS.VKWK.GKSREELTESVEFF
gene-BT93_F1001                    ....VRALEIQG.KSI.KFTAI.GVYL.ED..AA.LPSLA.PKWS.GKSAEELADSIEFF
MELO3C025484.2                     ....VRALEIGG.NTV.KFTAI.GVYL.ED..KA.VPSLA.GKWS.GKSAAELMDSVEFF
Csa_5G505170                       ....VRALEIGG.NTV.KFTAI.GIYL.ED..KA.VPSLA.GKWS.GKSAAELMDSVEFF
gene-DM860_013525                  ....ERGLNLDG.KFV.KFTAI.GVYL.EA..DA.VSSLA.VKWN.GKSPDELMDSVEFY
geneJL025816                       ....ARGIEVGG.KFI.AVTVI.GVYL.EA..AA.ISAIA.GKWK.GKKAEELAGSVEYY
geneJL015184                       ............................................................
CHI_2                              ....VRGLEIQG.KFI.KFTAI.GVYL.ED..SA.IPSLA.LKWK.GKTADELMKSVGFF
Dinv31395                          ....ARGLEIEG.KFI.KFTAI.AVYL.ED..NA.IPSLA.VKWK.GKSAEELTDSVEFF
CHI                                ............................................................
CHI_2                              ............................................................
CHI_3                              ............................................................
gene-KFK09_000997                  ....VRGVEVGG.KFI.AVTVI.GVYL.EA..AA.IPAID.GKWK.GKTAEELSSSAEFY
gene-BAE44_0007788                 ............................................................
gene-BAE44_0020983                 ....VRGMEAEG.NFF.KIAAI.GVYL.ED..AA.VPALA.GKWA.GKTADELASDPAFF
Dexi3A01G0013540                   ....VRGMEAEG.NFV.KIAAI.GVYL.ED..AA.VASLA.GKWA.GKSADELASDPAFF
Dexi3B01G0013780                   ....VRGMEAEG.NFV.KIAAI.GVYL.ED..AA.VASLA.GKWA.GKSADELASDPAFF
D.long004984                       ....ERGLEIQG.KFI.KFTAI.GVYL.ED..VA.VSWLA.VKWK.GKTAEELTESVEFF
gene-IHE45_09G071500               ....VRGLEIGD.KFI.KFTAI.GVYL.GN..GV.VASLA.GKWK.GKSGDELSNSVEFF
DRNTG_23933                        ....VRGLEIGD.KFI.KFTAI.GVYL.GN..GA.VASLA.GKWK.GKSGDELSNSVEFF
gene-J5N97_002300                  ....VRGMQIGD.KFI.KFTAI.GVYL.GK..GA.VASLA.GKWK.GKSEEELSNSVDFF
gene-LOC111309827                  ....ERGLEIQG.KFI.KFTTI.GVYL.EY..NA.VECLA.VKWK.DKSADELTESVEFF
scaffold13.535                     ....VRGMEADG.NFV.KIAAI.GVYL.ED..AA.APALA.GKWA.GKSADELASDPAFF
scaffold176.249                    ....VRGMDIGG.NFI.KFTAI.GVYL.EEAAAA.VPALA.KKWA.GKSADELASDVAFF
scaffold36.570                     ....VRGMEADG.NFV.KIAAI.GVYL.ED..AA.APALA.GKWA.GKTADELASDPAFF
scaffold3.658                      ....VRGMEIGG.NFI.KFTAI.GVYL.EE.AAA.VSALA.KKWA.GKSADELASDVAFF
scaffold65.276                     ....VRGMEIGG.NFI.KFTAI.GVYL.EE.AAA.VSALA.KKWA.GKSADELASDVAFF
gene-LOC105035984                  ....CRGLEIGG.RFI.TFTAI.GIYL.ED..EA.LRSLS.GKWK.GKTTDELAASLEFF
gene-PR202_ga11488                 ....VRGLEIDG.NFV.KFAAI.GIYL.ED..AA.VLALS.SKWS.GKTADELAADAAFF
gene-PR202_ga13968                 ....VRGMEIGG.NFI.KFTAI.GVYM.ED..AA.VPALA.KRWA.GKTADELAADTAFF
gene-PR202_gb10465                 ....VRGMEIGG.KFI.KFTAI.GVYV.ED..AA.VPALA.KRWA.GKTADELAADTAFF
gene-EJB05_03819                   ....VRGMEIGG.NFI.KFTAI.GVYV.ED..AA.VTALA.KRWA.GKTADELAADAAFF
Et_4A_034194                       ....VRGMEIGG.NFI.KFTAI.GVYV.ED..AA.VTALA.KRWA.GKTADELAADAAFF
Et_4B_038342                       ....VRGMEIGG.NFI.KFTAI.GVYV.ED..TA.VTALA.KRWA.GKTADELAADAAFF
Et_6A_046252                       ....VRGLEIGG.NFI.KFTAI.GVYL.ED..MA.VTVLA.KRWA.GKTAEELAADAAFF
Et_9A_062905                       ....VRGIEIEG.NFI.KFASI.GVYL.ED..AA.AAALS.GKWS.GKAADELAGDPAFF
Et_9B_065446                       ....VRGIEIEG.NFI.KFASI.GVYL.ED..AA.AAALSAGKWS.GKTADELAADPAFF
EUGRSUZ_F03816                     ....ARGLEIQG.KFI.KFTAI.GVYL.ED..AA.LPSLA.AKWS.GKSAEELADSVEFF
EVM20prediction20Chr12.1151        ....VRGLDIQG.NFV.KFTAI.GVYL.ED..DA.VTSLS.GKWK.GKTAEELTESVEFF
Casp15914                          ....ARGMEIEG.KFV.KFTAI.GVYL.EE..EA.VPLLA.VKWK.GKNAEELTDSVEFF
EUTSA_v10004922mg                  ...........G.KFV.IVTVI.GVYL.EA..LA.VPSLS.VKWK.GKNEKELTESISFF
EUTSA_v10010658mg                  ....VRGLDIHG.KFV.IFTVI.GVYL.DA..AS.LPSLS.VKWK.GKTTEELTESVPFF
EVM_prediction_Fe1.6811            ....VPSASLDGCDF.............E............HWLV...............
EVM_prediction_Fe3.10426           ASSCRR.......RLLPPPVAVPSSFL........IPFL...................FF
EVM_prediction_Fe4.5584            ............................................................
EVM_prediction_Fe5.14515           ....VRGLTIEG.KFI.TFSAI.GIYF.EE..TA.VASLA.DKWK.GKSATELTESVEFF
EVM_prediction_Fe5.14516           ....VRGLTIQG.TFI.SFTAI.GIYF.EE..TA.VASLA.DKWK.GKSATELAESVEFF
FCD_00011569                       ....VRGMEIQG.KFV.KFTAI.GVYL.ES..NA.VTWLA.GKWK.GKSADELTDSVQFF
FCD_00011572                       ....VRGMEIQG.KFV.KFTAI.GVYL.ES..NA.VTWLA.GKWK.GKSADELTDSVQFF
FCD_00011567                       ....IGGLVEDG.IFV.KYSVI.GVYL.AE..SS.LPWLA.VKWK.GKTAKELVDFLKEY
FCD_00011574                       ....VRGLEIQG.KFV.KFTAI.GVYL.EF..NA.VTWLA.GKWK.GKSAEELNDSVEFY
maker-Fvb7-2-augustus-gene-221.49  ....ARGMEIQG.NFV.KFTAI.GVYL.EG..KA.VPALA.VKWK.GKTAEELTESVEFF
maker-Fvb7-3-augustus-gene-78.40   ....VRGMEIQG.NFV.KFTAI.GVYL.ED..KA.VPALS.VKWK.GKTAEELTESVEFF
EVM20prediction20scaffold_9.28     ....VRSIEAGG.KFI.AVTAI.GVYL.EE..AA.IPVIT.GKWK.GRTAEELYGSAEFY
gene-GLYMA_20G241700v4             ....VRGLQIHH.AFV.KFTAI.CIYL.QY..DA.LSFLS.VKWK.TKSTHQLTESDQFF
TnS000078355t12                    ....IRGVPIQG.KFT.PVTTI.GIYI.DK..DMIFNHLQ.NKWK.GKSGEQLNHSDEFI
TnS000589875t03                    ....VRGMKSDG.KFV.VFTLS.GWYL.EE..RH.IEKLR.HKWR.GKSAEQLLQSDEFF
gene-EPI10_010037                  ....ERGLEIQG.KFI.KFTAI.GVYL.ED..SA.LNCLG.VKWK.GKSAVELTESVEFF
GG16G068580                        ....LRGLDIEG.KFV.KFTAI.GVYL.ED..DA.VPFLS.VKWN.GKTAEELTDSVAFF
GG16G068530                        ....LRGLDVEG.KFV.TFTAI.GVYL.ED..DA.VPLLS.VKWN.GKTVEELTDSVAFF
gene-CDL12_22415                   ....ARGLEIEG.KFV.KFTAI.GVYL.ED..NA.VQSLA.AEWK.GKSTEELTNSVEFF
gene-CDL12_26251                   ....ARGLEIEG.KFV.KFTAI.GVYL.ED..NV.VQSLA.AKWK.GKSTEELTNSVDFF
gene-LOC110910959                  ....VRGLDIQG.NFV.KFSGI.GVYL.EY..KA.ISSLA.VKWK.GKSIEELTDSIEFY
gene-LOC110913037                  ....VRGLEIQG.NFV.KFTGI.GVYL.ED..KA.ISSLA.VKWK.GKTAQELTDSVEFY
gene-LOC110913039                  ....VRGMNIEG.DFV.KFTGH.GVYL.EY..KA.ISLLA.VKWK.GKSVEELTDSVEFY
gene-LOC110425216                  ....ERGLEIQG.KFV.KFTAI.GVYL.ED..IA.VESLA.VKWK.GKSAEVLTESVEFF
gene-P3X46_014162                  ....VRGLEIQG.KFV.KFTAI.GVYL.ED..EA.VPLLA.VKWK.GKSAQELTDSVEFF
gene-ZWY2020_018680                ....VRGMEIGG.NFI.KFTAI.GVYL.QA.DAA.VSALA.AKWA.GKPAADLASDAAFF
Hyque.04G056200.v1.1               ....SRGLEIEG.KFI.KFTAI.GVYL.ED..NA.VPSLA.VKWK.GKSAEELTESVEFF
gene-LOC109186979                  ....ARGLNIDG.KFV.KFTAI.GVYL.EA..DA.VPSLA.VKWN.GKSAEELTDSVQFF
itb05g04360                        ....ARGLNIDG.KFV.KFTAI.GVYL.EA..DA.VPSLA.VKWN.GKSAEELTDSVQFF
gene-M6B38_184735                  ....RRGLEMGG.KFV.TFTAI.GVYL.EE..AA.VPSLA.HKWK.RKTADQLLASNHFF
gene-M6B38_299855                  ....VRGMDIGG.NFV.RFTSI.GVYL.EK.GAA.IEALA.AKWK.GKTAEELVASVEFF
gene-M6B38_364490                  ....RRGLEMGG.KFV.TFTAI.GVYL.EE..AA.VPSLA.HKWK.RKTADQLLASNHFF
gene-M6B38_399615                  ....VRGMDIGG.NFV.RFTSI.GVYL.EK.GAA.IEALS.AKWK.GKTAEELVASVEFF
Jmimo22322                         ....ARGLEIEG.KFI.KFTAI.GVYL.ED..SA.VPSLA.VKWK.GKSADELSDSVDFF
HT24166                            ....VRGLEIEG.RFI.KFTAI.GVYL.QD..NA.VSSLA.VKWK.GKTAEELTDSVDFF
Joasc.10G103600.v1.1               ....ARGLEIGG.NFV.KFTAI.GVYL.EE..AA.VPALA.GKWG.GKAGDELAADAGFF
Jr07_35750                         ....ERGLEIQG.NFV.KFTAI.GVYL.ED..TA.VPSLA.IKWS.GKSADELSESVEFF
```

```
                                                90        100       110       120       130
Medicago                              .RDIIS.....GP.FEKLIRGSKI..RELSGPEYSRKVMENCVAHLKSVGTYGDAEAEA.
CICLE_v10032697mg                     .RDVVT.....GP.FEKFMKVTMI..LPLTGAQYSEKVAENCIAIWKFFGIYTDAEAKA.
gene-COCNU_12G007210                  ...MET.....GS.FEKFTRVTLI..LTLTGEQYSEKVAENCIAIWKAAGIYTEAEGIA.
gene-COCNU_12G007220                  ......................MV..LPLTGQQYSEKVTENCVAAWKAAGIYTEAEGMA.
gene-COCNU_12G007230                  .RDIFS.....GS.FEKFTRVTMV..LPLTGQQYSEKVTENCVAAWKAAGIYTEAEGMA.
gene-GSCOC_T00029776001               .RDIVT.....GP.FEKFIRVTMI..LPLTGRQYSEKVAENCAAYWKAVGIYTDAEGKA.
gene-IFM89_035037                     ...MHA.....GP.FEKIVLVKMI..SQLMGQQYAMGLTESCIRFWKTLGNYTESKDKGL
gene-IFM89_035040                     .RDVVT.....GP.FEKFIIITMI..LPLTGQQYSEKVKERCLAVWKALGIYTEAEGKA.
gene-CCACVL1_07583                    .RDIVT.....GA.FEKFIRVTMI..LPLTGQQYSEKVAENCVAIWKSLGLYTDEEAKA.
Cav07g20270                           .RDIVT.....GP.FEKFTRVTMI..LPLTGQQYSEKVSENCVAFWKSVGIYTDAEAKA.
Cav07g20290                           .RDIVT.....GP.FEKFTRVTMI..LPLTGQQYSEKVSENCVAFWKSVGIYTDAEAKA.
gene-BT93_F1001                       .RDIVT.....GP.FEKFSRVTMI..LPLTGAQYSEKVTENCVKYWQSVGTYTDAEAAA.
MELO3C025484.2                        .RDVVT.....GG.FEKFTNVTLI..LPLTGEQYAMKVAENCEAAWKSMGIYSDEGAEA.
Csa_5G505170                          .RDVVT.....GG.FEKFTNVTLI..LPLTGEQYAMKVAENCEAAWKSMGIYSDEGAEA.
gene-DM860_013525                     .RDLVT.....GP.FEKLTQIRMI..LPLSGKQYSEKVTENCIAHWKELGIYGDAEIDA.
geneJL025816                          .RDIIT.....GS.FEKLTRVTML..LPLTGQQYSEKVAENCIAAWKAAGVYTGEEEAA.
geneJL015184                          ...........GP.FEKLTRVTML..LPLTGQQYSEKVAENCIAAWKAAGVYTGEEEAA.
CHI_2                                 .NDIVT.....GP.FDKFTRVTTI..LPLTGKQYSEKVAENCVAHWKAIGSYTDEEAKA.
Dinv31395                             .RDIVS.....GP.FEKFTKVTMI..LPLTGKQYSEKVTENCVAYWKAVGSYTDAESKA.
CHI                                   ......................ML..LPLTGKQYSEKVAENCVAAWKAAGVYTEEEEAA.
CHI_2                                 ......................ML..LPLTGQQYSEKVTDNCIAAWKAADVYTGEE.AA.
CHI_3                                 ......................ML..LPLTGQQYSKKVTENCVAAWKAADVYTGEEEAA.
gene-KFK09_000997                     .RDIIT.....GS.FEKLTRVTML..LPLTGQQYSEKVAENCVAAWKAAGVYTEEEEAA.
gene-BAE44_0007788                    ......................MI..LPLTGEQYLEKVTENCGAYWKATGVYTDAEGVA.
gene-BAE44_0020983                    .RDVYT.....GE.FEKFTRVTFIWPNTVPAEEFAAKVMESRVAYLESTGAYTDAEAVA.
Dexi3A01G0013540                      .RDVYT.....GE.FEKFTRVTFIWPKTVAAEEFAAKVMESRVEFLKAAGAYTDAEAVA.
Dexi3B01G0013780                      .RDVYT.....GE.FEKFTRVTFIWPKTVAAEEFAAKVMESRVEYLKAAGAYTDAEAVA.
D.long004984                          .RDIVT.....GP.FEKFMKVTMI..LPLTGQQYSEKVTENCIAFWKSIGIYTDAEARA.
gene-IHE45_09G071500                  .RDVIT.....GP.FEKLTQVTMI..LPLTGQQYSEKVAENCISSWKEAGVYTEAEAKA.
DRNTG_23933                           .RDVIT.....GP.FEKLTQVTMI..LPLTGQQYSEKVAENCISFWKEAGVYTEAEAKA.
gene-J5N97_002300                     .RDIIT.....GP.FEKLTQVTMI..LPLTGQQYSEKVTENCISAWKAAGVYTEAEAKA.
gene-LOC111309827                     .RDIVT.....GA.FEKFIRVTMI..LPLTGQQYSEKVAENCVAIWKSLGLYTDAEAKA.
scaffold13.535                        .RDVYA.....GE.FEKLTRVTFVWPDTVAAEEFAAKVMESRVAYLKAAGAYTDAEAAA.
scaffold176.249                       .RDVVT.....GD.FEKFTRLTTL..KPLTGEEFAAKVMENCVAIMKAAGAYTDAEAAA.
scaffold36.570                        .RDVYA.....GE.FEKLTRVTFVWPDTVAAEEFAAKVMESRVAYLKAAGAYTDAEAAA.
scaffold3.658                         .RDVVT.....GG.FEKFTRVTMI..LPLTGEQFAGKVTENCVAYWKATGAYTDAEAAA.
scaffold65.276                        .RDVVT.....GD.FEKFTRVTMI..LPLTGEQYAGKVTENCVAYWKATGAYTDAEGAA.
gene-LOC105035984                     .RDIFT.....GS.FEKFTRVTMV..LPLTGQQYSEKVAENCVAAWKAAGIYTEAEGMA.
gene-PR202_ga11488                    .RDIYT.....GE.FEKFTRVTFI.GRPLDAKDFAGKVMESRVEYLKSACTYTDAEAAA.
gene-PR202_ga13968                    .RDIVT.....GE.FEKFTRVTTI..LPLTGEQYSEKVTENCVAYWKATGVYTDAEGVA.
gene-PR202_gb10465                    .RDIVT.....GE.FEKFTRVTMI..LPLTGEQYSEKVTENCVAYWKATGVYTDAEGVA.
gene-EJB05_03819                      .RDVVT.....GE.FEKFTRVTMI..LPLTGEQYSGKVTENCVAYWKATGVYTDAEGVA.
Et_4A_034194                          .RDVVT.....GE.FEKFTQVTMI..LPLTGEQYSDKVTENCVAYWKATGVYTDAEGAA.
Et_4B_038342                          .RDVVT.....GE.FEKFTRVTMI..LPLTGEQYSDKVTENCVAYWKATGVYTDAEGVA.
Et_6A_046252                          .RDVVT.....GE.FEKFTRVTMI..RLLTGEQYSEKVAENCMAYWKATGGYTDAEGEA.
Et_9A_062905                          .RDIYT.....GE.FEKFTRVAFI.GRPVNGEEFIAKVMESRVAYLKSAGTYTDAEAAA.
Et_9B_065446                          .RDIYT.....GE.FEKFTRVAFI.GRPVNGEEFVAKVMERRVAYLKAAGTYTDAEAAA.
EUGRSUZ_F03816                        .RDVVT.....GP.FEKFSRVTMI..LPLTGAQYAEKVTENCVKYWQSVGTYTDAEAVA.
EVM20prediction20Chr12.1151           .TDIVT.....GP.FGKFTQVTII..LPLTGKQYSEKVAENCVASWKATGTYTDAGGKA.
Casp15914                             .REIVT.....GP.FEKFVRVTMI..LPLSGQQYSEKVSENCVAIWKSMGIYSEAEAKA.
EUTSA_v10004922mg                     .RQIIT.....GV.FEKFVRVTMK..VNLTGTQYSEKVAEYCEEILKSSGKYTKTETKA.
EUTSA_v10010658mg                     .REIVT.....GA.FEKFIKVTMK..LPLTGQQYSEKVTENCVAIWKSLGIYTDCEAKA.
EVM_prediction_Fe1.6811               ...VVE.....NP.DEKATRDEII..........................ASYIETLAKV.
EVM_prediction_Fe3.10426              ......HSGNHGSRSEDLLHITLV..KNLTSAQYSAKVAENCVAICKAIGTYFEAKEKV.
EVM_prediction_Fe4.5584               ............................................................
EVM_prediction_Fe5.14515              .RDVVT.....GP.FEILMQITLV..KHLTGAQYSAKVAENCVAICKAIGTYSEAEEKA.
EVM_prediction_Fe5.14516              .RDVVT.....GQ.FEKFIQITML..KPLTGAQYSEKVSENCVAIWKAIGIYSEAEEKA.
FCD_00011569                          .RDIVT.....GP.FEKFTRITMI..LPLTGKIYSEKVAENCEAIWKSLGIYTETEEKA.
FCD_00011572                          .RDIVT.....GP.FEKFTRISMI..LPLTGKVYSEKVAENCEAIWKSLGIYTETEEKA.
FCD_00011567                          .RQ...........FEKFYRVTLV..YPLTGSKFVEKVEANSIAIMKSAGVYGDAEMRA.
FCD_00011574                          .RDIVT.....GP.FEKFTRITMI..LPLTGQQYSEKVSENCVAIWKSFGIYTEAEEKA.
maker-Fvb7-2-augustus-gene-221.49     .REIVT.....GP.FEKFTQVTMI..LPLTGQQYSEKVSENCVAIWKKFGIYTDAEAKA.
maker-Fvb7-3-augustus-gene-78.40      .REIVT.....GP.FEKFTQVTMI..LPLTGQQYSEKVSENCVAIWKKFGIYTDAEAKA.
EVM20prediction20scaffold_9.28        .RDMIT.....GS.FEKLTRVVMI..LPLTGQQYSEKVAENCVAAWKEADSYTEAEEAA.
gene-GLYMA_20G241700v4                .SDIVT.....GP.FEKFMQVTMI..KPLTGQQYSEKVAENCVAIWRSLGIYTDSEAEA.
TnS000078355t12                       .TDIVS.....SP.YQKMARVALL..YPLTGVEFSAKIAHNSKTILEQSGKFAEAEAKA.
TnS000589875t03                       VQDLSR.....SN.FKKTSRLVFA..THLTGPEFAAKVTGKCKNILQKWGMYGDAEEQA.
gene-EPI10_010037                     .RDVVT.....GD.FEKFVRVTMI..LPLTGQQYSEKVSENCVAIWKSLGIYTDAEAKA.
GG16G068580                           .RDLVT.....GP.FEKFIRVTMI..LPLTGVQYSEKVTENCVRIWKSLGIYTDSEAKA.
GG16G068530                           .RDLVT...............................................DSEAKA.
gene-CDL12_22415                      .KDIVT.....GP.FEKFTKVTMI..LPLTGQQYSEKVVENCVAYWKAVGKYTDAEAEA.
gene-CDL12_26251                      .KDIVT.....GP.FEKFTKVTMI..LPLTGQQYSEKVVENCVAYWKAVGKYTDAEAEA.
gene-LOC110910959                     .RDIVT.....GP.FEKFSQVTLI..QTLTGKQFSEKVAENCVAIWKSQGSYTEADAKT.
gene-LOC110913037                     .RDIVT.....GP.FEKFAQVTMI..LPLTGKQYAEKVSENCVAVWKALGIYTDADAKT.
gene-LOC110913039                     .RDIVT.....GP.FEKFAHVTMI..LPLTGKQFSEKVAENCVAIWKSFGIYTEADAKT.
gene-LOC110425216                     .RDIVT.....GA.FEKFIRVTTI..LPLTGQQYSEKVSENCVAIWKSLGLYTDAEAKA.
gene-P3X46_014162                     .RDIVT.....GP.FEKFIRVTTI..LPLTGEQYSEKVSENCAAIWKSLGIYTDAEAKA.
gene-ZWY2020_018680                   .RDVVT.....GE.FEKFTRVTMI..LPLTGAQYSDKVTENCVAYWKAAGVYTDAEAAA.
Hyque.04G056200.v1.1                  .RDIVS.....GP.FEKFMKVTMI..LPLTGKQYSEKVSENCVAYWKAVGTYTDAEGKA.
gene-LOC109186979                     .RDIVT.....GP.FEKLTRITMI..LPLSGKQYSEKVTENCVAFWKAAGMYGDAERKA.
itb05g04360                           .RDIVT.....GP.FEKLTRITMI..LPLSGKQYSEKVSENCVAFWKAAGIYGDAESKA.
gene-M6B38_184735                     .RDIYK.....GR.FEKLTKVTMV..LPLTGEQYSDKLTENCIAYWKGMGTYNKAKQAA.
gene-M6B38_299855                     .RDITM.....GS.FDKFTKVTMI..LPLTGKQYAEKVCENCVAHWKAIGIYTDAEAAA.
gene-M6B38_364490                     .RDIYK.....GR.FEKLTKVTMV..LPLTGEQYSDKLTENCIAYWKGMGTYNKAKQAA.
gene-M6B38_399615                     .RDITM.....GS.FDKFTKVTMI..LPLTGKQYAEKVCENCVAHWKAIGIYTDAEAAA.
Jmimo22322                            .RDIVT.....GP.FGKFTRTKLI..LPLKGEQYSEKVAENCVAYMKSMGKYTDAESEA.
HT24166                               .NDVIT.....GP.FEKFTRVTTI..LPLTGEQYSEKVAENCVAHWKATGTYTDAEAKA.
Joasc.10G103600.v1.1                  .GDVVT.....GE.FDKFTRVTMI..LPLTGEQYSEKVTENCVAYWKATGVYTDAEGEA.
Jr07_35750                            .RDIVT.....GP.FEKFIRVTMI..LPLTGHQYSEKVSENCVAYWKSVGIYTDAEAKA.
```

```
                                            140         150                   160         170
Medicago                                MQKFAEAFKPVNFPP..GASVFYRQSPD..........GILGLSFSP..DTSIPE..KEA
CICLE_v10032697mg                       IEKFTEVFKDEIFPP..GSSILFTQSP...........GSLTISFSK..DGSIPK..DGV
gene-COCNU_12G007210                    IEKFKEAFKAETFGP..GSSILFTNSPS..........GALTIAFSK..DSSVPE..AGC
gene-COCNU_12G007220                    VDKFKEAFKAKTFAP..GSSILFTHSPS..........GALTIAFSE..DGSVPE..AGR
gene-COCNU_12G007230                    VDKFKEAFKPETFAP..GSSILFTHSPS..........GALTIAFSE..DGSVPE..AGR
gene-GSCOC_T00029776001                 IEMFLDIFQNESFPP..GASILFTQSPL..........GSLTISFSK..DSSIPE..VSN
gene-IFM89_035037                       LEELIQVFQDEVFPP..NSTILFTQSS...........PTLTIGFSE..DGSIPK..EGK
gene-IFM89_035040                       MEEFIQVFKDENFPP..GSSILFTQSS...........PTLTIAFSK..DGSMPV..EAK
gene-CCACVL1_07583                      IEKFVEVFKNENFPP..GSSILFTISAE..........GSLTIGFSK..DSSVPE..SGT
Cav07g20270                             IEKFLEVFKDETFPP..GSSILFTQSPN..........GSLTISFSK..DGSLPE..IGN
Cav07g20290                             IEKFIEVFKDETFPP..GSSILFTQSPN..........GSLTISFSK..DGSLPE..IGN
gene-BT93_F1001                         VEKFRDVFKDQSFPP..GSSILFTLSPN..........GSLTVAFSE..DGSVPK..TSN
MELO3C025484.2                          IKKFIDAFKNENFPP..GSSILFTHLPP..........NTLSISFSK..DGNIGE..KEE
Csa_5G505170                            IQKFIDIFKNENFPP..GSSILFTHLPP..........NTLSISFSK..DGSIGE..KEE
gene-DM860_013525                       IDKFLQVFSDQMFPP..AASILFSHSHS..........GSLTISFSK..DGAIPK..TPN
geneJL025816                            INKFLEIFKPKSFPP..GTSIIFTHSPH..........GSLTIGFLE..EGGDPV..AET
geneJL015184                            INKFLEIFKPKSFPP..GTSIIFTHSPH..........GSLTIGFLE..EGGGPV..AET
CHI_2                                   IDKFLE....................................IGFSK..ETSIPK..DGK
Dinv31395                               IEKFIEVFKDEIFPP..GASILFTQSPL..........GSLTIGFSK..DGSLPE..EGI
CHI                                     IVKFLEIFKPKNFPP..GTSIVFSHSPS..........GTLTIGFLE..LGGVPA..AES
CHI_2                                   IIKLLEIFKPKSFPP..VTSTVFSHSPS..........GTLTIGFLE..LGGVPA..AES
CHI_3                                   IIKLLEI......................................LE..LGGVPA..AES
gene-KFK09_000997                       VVKFREIFKPKNFPP..GTSIVFSHSPS..........GTLTIGFLE..LGGVPA..AES
gene-BAE44_0007788                      VEKFKEAFKPEKFPP..GASILFTHSPT..........RVLTVAFSK..DSSVLE..FSG
gene-BAE44_0020983                      VEEFKVTFKPHSLAP..GDSVLFTHSPA..........GVLTVAFSG..DSSVPV..AGI
Dexi3A01G0013540                        VEEFKAALKNQSLAP..GASVLFTHSPA..........GVLTVAFSD..DSSVPE..AGI
Dexi3B01G0013780                        VEEFKAALKNQRLAP..GASVLFTHSPA..........GVLTVAFSD..DSSVPE..TGI
D.long004984                            TEKFTKIFKDETFPP..GTSILFTQSPH..........GSLTISFSK..DGTIPE..VGN
gene-IHE45_09G071500                    VEKFKEIFHHETFPH..AASIIFSHSFS..........GSVSIGFSK..DGSIPK..TWN
DRNTG_23933                             VEKFKEIFHHETFPH..AASIIFNHSLS..........GSVSIGFSK..DGSLPK..SWN
gene-J5N97_002300                       TDNFKEIFNPETFPH..AASIIFNHSPS..........GSLSIAFSK..DSSIPK..TWN
gene-LOC111309827                       IEKFIEVFKDDNFPP..GSSILFTLSAQ..........GSLTIGFSK..DGSVPE..VGT
scaffold13.535                          VEEFKA....................................VAFSG..DSSVPG..AGV
scaffold176.249                         VDKFKETFKPETLPP..GKSILFMQSPT..........GVLTVAFSK..DSSVPE..TGG
scaffold36.570                          VEEFQAAFRTRTLAP..GASVLFTHSPA..........GVLTVTFSG..DSSVPG..AGV
scaffold3.658                           VEKFKEAFKPETFPP..GASILYTHSPN..........GALTVAFSK..DSSVPE..SGG
scaffold65.276                          VEKFKEAFKPETFPP..GASILFTHSPT..........GALTVAFSK..DSSVPE..SGG
gene-LOC105035984                       IDKFKEAFKAENFAP..GSSILFTHPPS..........GALTIAFSE..DGSVPE..AGR
gene-PR202_ga11488                      VDEFKAVFKGQTLPP..GASALFTHSPA..........GVITVAVSE..DSSVPE..TGA
gene-PR202_ga13968                      VEKFKEAFKPETFPP..GASILFTHSPA..........GNLTVAFSK..DSSVPE..TGG
gene-PR202_gb10465                      VEKFKEAFKPETFPP..GASILFTHSPA..........GVLTVAFSK..DSSVPE..TGG
gene-EJB05_03819                        VEKFKEAFKPETFPP..GASILFTHSPA..........GVLTVAFSK..DSSVPE..AGG
Et_4A_034194                            VEKFKEAFKPETFPP..GASILFTHSPA..........GVLTVAFSK..DSSVPE..AGV
Et_4B_038342                            VEKFKDAFKPETFAP..GASILFTHSSA..........GVLTVAFSK..DSSVPE..AGV
Et_6A_046252                            VVKFKEAFKPETFPP..GASVLFTYSP..............VIAFSE..DSSVPE..ADG
Et_9A_062905                            VDEFKAAFKQQTFPP..GASVLFTHSPA..........GVITVAFDE..DSSAPD..MVV
Et_9B_065446                            VEEFKAAFKQQTFPP..GASVLFTHSPA..........GVITVAFDK..GSSAPD...IV
EUGRSUZ_F03816                          VEKFREVFKDQSFPP..GASILFTLSPN..........GSLTIALSE..DGSVPE..ASN
EVM20prediction20Chr12.1151             VEKFLDVFKDQTFPP..GSSILFTTSPL..........GSLTISFTK..DGSFPE..IGH
Casp15914                               IDKFLDLFKDEKFPP..GSSILFTLLPH..........GSLAITFWK..EGSMPE..TEN
EUTSA_v10004922mg                       IDKFLKVFKDQDFPP..GSSILFAICPK..........GSLTIAFSK..DEKVPR..SRN
EUTSA_v10010658mg                       VERFLEVFKDQNFLP..GASILFALSHN..........GSLTVAFSK..DDSIPE..TGK
EVM_prediction_Fe1.6811                 VRRVWEA...................................LEIAFEK..HDVILE..AYV
EVM_prediction_Fe3.10426                VEKFMEIFKEENFSH..ETSLLFKQCPPKSLANLNFACGTLEIVFGK..HDIIPE..AYV
EVM_prediction_Fe4.5584                 ....MEIFKEENFPP..GTSILFKQCPPKSLGDLNFACGTLEIAFGK..HDVILE..ACV
EVM_prediction_Fe5.14515                VEKFMEIFKEENFPP..GTSVLFKQCPPKSLANLKFACGTLEIAFGK..HDVIPE..ADV
EVM_prediction_Fe5.14516                IEKFTEIFKEQNFPP..GTSILFKQCAP..........KSLRIAFGK..HDAIPE..ADV
FCD_00011569                            LEKFLQVFKDQNLLP..GSSVLFTQSSS..........GSLTISFSK..DESIPE..KEN
FCD_00011572                            LEKFLQIFKDQNLLP..GSSVLFTQSPS..........GSLMISFSK..DESIPE..KEN
FCD_00011567                            TENFVELFQKENLPPKTGFSIIYTQSPA..........GSLPVRFSN..DGSIPE..EGN
FCD_00011574                            IEMFLQVFKDKNFPP..GSSVLFTQSPS..........GSLTISFSK..DESIPE..KWD
maker-Fvb7-2-augustus-gene-221.49       IEKFIEVFKDQTFPP..GASILFTQSPN..........GSLTIGFSK..DGCIPE..VGN
maker-Fvb7-3-augustus-gene-78.40        IEKFMEVFKDQTFPP..GASILFTQSPN..........GSLTIGFSK..DGSIPE..VGN
EVM20prediction20scaffold_9.28          INSFLEIFKPRSFIH..GSSIVFAHSPN..........GSLTIEFLE..EGREGE..AVP
gene-GLYMA_20G241700v4                  IDKFLSVFKDLTFPP..GSSILFTVSPN..........GSLTISFSG..DETIPE..VTS
TnS000078355t12                         IEEFTEAFKDENLPA..RASAMFSVSPS..........GLV.ISICK..KHGEEQ..KMK
TnS000589875t03                         LQEFRDVFRDEDLYP..GACGFFNISPA..........GLG.IAVANN.TEVVPA..TLK
gene-EPI10_010037                       IEQFIEVFKDENFPP..GSSILFTISGQ..........GSLTIGFSK..DSSVPE..GGK
GG16G068580                             VEKFLEIFKEETFPP..GSSILFALSRE..........SLLTIAFSK..DDSIPE..TGK
GG16G068530                             VEKFLEIFKEETF...........................LRIAFSK..DDSIPE..TGK
gene-CDL12_22415                        TEKFLQVFRDETFPP..GASILFTQSPA..........GSLTISFSQ..DGSIPE..RGK
gene-CDL12_26251                        TEKFLQVFREETFPP..GASILFTQSPA..........GSLTISFSQ..DGSIPE..RGE
gene-LOC110910959                       IDKFLEIFKDQIFPP..GSSILFTISLL..........GSLTITFSK..DGSIPE..TGD
gene-LOC110913037                       IDKFLEIFSDQNFPP..GSSILFTISPA..........GSLTISFSK..DGSIPE..TGN
gene-LOC110913039                       IDKFLEIFKDQNFPP..GSSILFTISLV..........GSLTISFSK..DGCIPE..AGS
gene-LOC110425216                       IEKFLEVFRDENFPP..GSSILFTLSAH..........GSLTISFSK..DSSVPQ..VGT
gene-P3X46_014162                       IDKFLEVFKAQTFPP..GSSILFTQLPN..........GSLAIRFSK..DGSIPE..AEN
gene-ZWY2020_018680                     VDKFKEAFGPHSFAP..GASILFTHSPA..........GVLTVAFSK..DSSVPE..SGG
Hyque.04G056200.v1.1                    IEKFIDVFENETFPP..GASILFTQSPL..........GSLTVAFSK..DGSLPE..EGV
gene-LOC109186979                       IDKFNDVFSDQMFPP..GASIFFNQSPL..........GSLTISFSK..DGSTPE..TAS
itb05g04360                             IEKFNDVFSDQMFPP..GASIFFTQSPL..........GSLTISFSK..DGSMPE..IAS
gene-M6B38_184735                       IDRLKEVLKPKTFSH..GDSIVFAHSTN..........GSLAIGFLKNNNDSVPADSGGA
gene-M6B38_299855                       VEKFKQVLEPETFPP..GSSILFHHSAT..........GTLTIAFSK..DESIPE..AGV
gene-M6B38_364490                       IDRLKEVLKPKTFSH..GDSIVFAHSTN..........GSLAIGFLNKNNDSVPADSGGA
gene-M6B38_399615                       VEKFKQVLEPETFPP..GSSILFHHSAT..........GTLTIAFSK..DESIPE..AGV
Jmimo22322                              TQKFRQAFKDETFSP..GASIFYTQSPS..........GSLTIGFST..DDSIPE..EGN
HT24166                                 IQKFLEVFKNETFPP..GSSILFTQSPT..........GSLTISFSK..GDSLPE..TGN
Joasc.10G103600.v1.1                    VEKFKETFRSETFPP..GSSILFTHSSN..........GTLTIAFSK..DASAPE..VGS
Jr07_35750                              IEEFLKVFKDEKFPP..GSSILFTQSPN..........GSLTISFSK..DESIPE..SGN
```

```
                                                180       190       200       210
                                                  .         .         .         .
Medicago                                   .AL...IENKAVSSAVLETMIGEHAV....SPDLKRCLAARL..PAL.............
CICLE_v10032697mg                          .AV...IESNLLSEAVLESMIGKNGV....SPAAKKSLAERL..SAL.............
gene-COCNU_12G007210                       .AV...IENKALCEAVLESIIGEHGV....SPAAKRSLALRV..SEL.............
gene-COCNU_12G007220                       .VV...IENKTLCEAVLESIIGEHGV....SPAAKRSLALRV..SEL.............
gene-COCNU_12G007230                       .VV...IENKTLCEAVLESIIGEHGV....SPAAKRSLALRV..SEL.............
gene-GSCOC_T00029776001                    .AV...VENKLLSEAVLESIIGKNGV....SPDTKKSLAVRL..SDL.............
gene-IFM89_035037                          .AN...IENKRLSEAILGSAICKGGV....SPATRRSLAVRL..CDL.............
gene-IFM89_035040                          .AK...IVNKPLSEAFLESVIGKHGV....SPEAKRSLAVRI..SEL.............
gene-CCACVL1_07583                         .AV...IDNKLLANSVLESIIGKGGV....SPAAKQSLASRL..PEL.............
Cav07g20270                                .AV...IENKLLSEAVLESIIGKHGV....SPEAKQSLAARL..AES.............
Cav07g20290                                .AV...IENKLLSEAILESIIGKHGV....SPEAKQSLATRK..TKMNVSGNVFITMATT
gene-BT93_F1001                            .AV...IENKQLAEAIFESMIGKHGV....SPEAKTSLASRI..SEL.............
MELO3C025484.2                             .EMVRKIENKLLSESVLESIIGKNGV....SPAARLSLATRL..SHL.............
Csa_5G505170                               .EMVKKIENKLLSESVLESIVGKNGV....SPAARLSLASRL..SHL.............
gene-DM860_013525                          .AV...IENKNLSEAVLESIIGVNGV....SPAAKRSLALRL..SEL.............
geneJL025816                               .GV...VENKKLTNAVLESIIGEKGV....SPAAKQSLAQRI..SEF.............
geneJL015184                               .GV...VENKKLTNAVLDSIIGEKGV....SPAAKQSLAQRI..SEF.............
CHI_2                                      .AV...IHNKQLAEAVLDSIIGEHGV....SPAAKHSLAVRI..SDL.............
Dinv31395                                  .AV...IENKQLSEAVLESIIGKHGV....SPEAKQSLASRI..SLL.............
CHI                                        .GV...IENKKLTNAVLESIIGEKGV....SPAAKQSLAQRI..SEF.............
CHI_2                                      .GV...IENKKLTNAVLESIIGEKGV....SSAAKQSLAKRI..SEF.............
CHI_3                                      .GV...IENKKLTNAVLESIIGEKGV....SPAAKQSLAKRI..SEF.............
gene-KFK09_000997                          .GV...IENKKLTNAVLESIIGEKGV....SPAAKQSLAQRI..SEF.............
gene-BAE44_0007788                         .VA...IENKPLCEAVLESIIGEHGV....SPAAKLSIAGRV..SEL.............
gene-BAE44_0020983                         .AA...IENKALCEAVLESIIGERSV....SPATKQSIATRL..PEI.............
Dexi3A01G0013540                           .AA...VENRALCEAVLESIIGERSV....SPATKQSIAARA..PEI.............
Dexi3B01G0013780                           .AA...IDNRALCEAVLESIIGERSV....SPATKQSIATRA..PEI.............
D.long004984                               .AV...IENKLLSEAILESMIGKHGV....SPPAKKSLAARL..SIL.............
gene-IHE45_09G071500                       .AV...IENKRLSEAILESIIGEHGV....SPAAKKSLAFRL..SKL.............
DRNTG_23933                                .AV...IENKRLSEAILESIIGEHGV....SPPAKKSLAFRL..SKL.............
gene-J5N97_002300                          .TV...IENKKISEAVLESIIGEHGV....SPEAKKSLASWL..SKS.............
gene-LOC111309827                          .AV...IENKLLANSVLESIVGKNGV....SPVAKQSLASRL..SAL.............
scaffold13.535                             .AAI..IENKALCEAVLESIIGERSV....SPAMKRSIAARL..PEI.............
scaffold176.249                            .VA...IENKPLSEAVLDSIIGERGV....SPAAKLSIAARM..SEL.............
scaffold36.570                             .AA...IENRALCEAVLESIIGERSV....SPATKQSIAARV..PEI.............
scaffold3.658                              .VA...IENKPLCEAVLESIIGESGV....SPAAKLSIAARV..SEL.............
scaffold65.276                             .VA...IENKRLCEAVLESIIGERGV....SPAAKLSIAARV..SEL.............
gene-LOC105035984                          .VV...IDNKTLCEAVLESIIGEHGV....SPAAKRSLALRV..SEL.............
gene-PR202_ga11488                         RAA...IENKVLCEAVMESIIGERIV....SPATKLSIATRA..REL.............
gene-PR202_ga13968                         .VA...IENKPLCEAVLESIIGEHGV....SPAAKLSIATRV..SGL.............
gene-PR202_gb10465                         .VA...IANKPLCEAVLESIIGEHGV....SPAAKLSIATRV..SGL.............
gene-EJB05_03819                           .VA...IENKPLCEAVLESIIGEHGV....SPAAKLSIATRV..SEL.............
Et_4A_034194                               .VA...IENRPICEAVLESIIGEHGV....SPAAKLSIATRV..SEL.............
Et_4B_038342                               .VA...IENKPICEAVLESIIGEHGV....SPAAKLSIATRV..AEL.............
Et_6A_046252                               .VA...VENKQLCEAVLESIIGEHGV....SPAAKLSIAARM..SEL.............
Et_9A_062905                               .AA...VENKALCEAVLESIIGERSV....SPATKQSIASRV..PEL.............
Et_9B_065446                               .AA...VENKALCEAVLESIIGERSV....SPATKQSIASRV..PEL.............
EUGRSUZ_F03816                             .TV...IENRQLTEAIFESMIGKHGV....SPEAKTSLASRI..SEL.............
EVM20prediction20Chr12.1151                .VE...IESKQLSEALLDSIIGKNGV....SPAAKKSIATRI..SDL.............
Casp15914                                  .GV...IENKLLSEAVLESIIGKHGV....SPEARSSIASRR..S...............
EUTSA_v10004922mg                          .AV...IKNKLLGEAIIESMIGKNGV....SPETKGISNARF..KRS.............
EUTSA_v10010658mg                          .AV...IENKLLAEAVLESIIGKKGV....SPGTRLSLAERL..AQL.............
EVM_prediction_Fe1.6811                    .VV...IENEPFSQAVLESIIGKDRV....SPAARESLTARL..YQQ.............
EVM_prediction_Fe3.10426                   .GV...IENEPFPQAVLESIIWKDSS....SGRQGELGC.....................
EVM_prediction_Fe4.5584                    .FV...IENVPFSQAVLESIIGKDGV....STAVRESLAARNSLKNI.............
EVM_prediction_Fe5.14515                   .AV...IENGPFSQAVLESIIGTDGV....SPATRESLAARL..HQL.............
EVM_prediction_Fe5.14516                   .AV...IENGPLSQSVLESIIGKNGV....SPAARESLAVRL..HEL.............
FCD_00011569                               .DV...IENKLLSEAILESIIGKHGV....SPETRRSLASSL..AEL.............
FCD_00011572                               .AI...IENKLLSEAILESIIGKHGV....SPEARRSLASSL..AEL.............
FCD_00011567                               ..V...IVNKALLEAVLDSIVGKNGV....SPAAKRSLAERL..SEL.............
FCD_00011574                               .VV...IENRLLSEVILESIIGKHGV....SPDAKRSMASIL..AEL.............
maker-Fvb7-2-augustus-gene-221.49          .AV...IENKLLSESVLESIIGKQGV....SPEARKSVATRL..SEL.............
maker-Fvb7-3-augustus-gene-78.40           .AV...IENKLLSESVLESIIGKQGV....SPEARKSVATRL..SEL.............
EVM20prediction20scaffold_9.28             .AV...IKNRKLSNAVLESIIGEKGV....SPAAKLSLAQRI..SGL.............
gene-GLYMA_20G241700v4                     .AV...IENKLLSEAVLESMIGKNGV....SPAAKQSLASRL..SHL.............
TnS000078355t12                            .RK...IESRLLGEAVLLSMIGKDGV....SPHARASVASRL..ATL.............
TnS000589875t03                            .KQ...IGNAALAQAYMASVLGMHGV....SPAARVSIASRL..AAV.............
gene-EPI10_010037                          .TV...IENKLLANSVLESIIGKNGV....SPAAKESLASRL..SPL.............
GG16G068580                                .AV...IENKLLAEAVLESIIGKNGV....SPATKQSLGNRL..SLL.............
GG16G068530                                .AV...IENKLLAEAVLESIIGKMEFLLRRNRVSKQIIAAAE..EGR.............
gene-CDL12_22415                           .AT...IENKQLSEAVLESIIGKHGV....SPAAKQSLAARL..SDL.............
gene-CDL12_26251                           .AT...IENKQLSEAVLESIIGKHGV....SPAAKQSLAARL..SDL.............
gene-LOC110910959                          .VV...IEDGKLGEAILESQIGKHGV....SPATKKSLASRL..YNL.............
gene-LOC110913037                          .AV...LESDKLGEAILESMIGKHGV....SPAAKQSLASRL..SDL.............
gene-LOC110913039                          .VV...IEDEKLGEAILESMIGKHGV....SPATKKSLASRL..FDL.............
gene-LOC110425216                          .AV...IENKLLANAVLESIVGKDGV....SPVARKSMASRL..SAL.............
gene-P3X46_014162                          .AV...IQNKLLSEAVLESIIGKNGV....SPATRETMATRL..TEL.............
gene-ZWY2020_018680                        .VA...IENARLCEAVLESIIGEHGV....SPAAKLSLANRV..AEL.............
Hyque.04G056200.v1.1                       .VV...IENKQLSEAVLESMIGKHGV....SPEAKQSLATRI..AGL.............
gene-LOC109186979                          .AI...IENKPLSEAVLESIIGSKGV....SPEAKQSLAVRL..SDL.............
itb05g04360                                .AV...IENKPLSEAVLESIIGVKGV....SPEAKQSLAVRL..SEL.............
gene-M6B38_184735                          .AV...IKSRALSEAVLESIIGKNGV....SPAAKKSLAMRI..SAL.............
gene-M6B38_299855                          .AV...IENRPLTVAILESIIGEHGV....SPAAKQSLALRV..ASH.............
gene-M6B38_364490                          .AV...IKSRALSEAVLESIIGKNGV....SPAAKKSLAMRI..SAL.............
gene-M6B38_399615                          .AV...IENRPLTVAILESIIGEHGV....SPAAKQSLALRV..ASH.............
Jmimo22322                                 .AV...IENKQLSEAVLESIIGKHGV....SPTAKLSLAARL..SEL.............
HT24166                                    .AV...IENKQLSAAVLQSIIGKHGV....SPEAKKSLAARI..LDL.............
Joasc.10G103600.v1.1                       .AV...IENKAFCEAILESIIGEHGV....SPEAKRSLATRV..SEL.............
Jr07_35750                                 .AV...IKNKLLSEAVLESIIGKHGV....SPAAKKSLATRV..SEL.............
```

Medicago	....
CICLE_v10032697mg	....
gene-COCNU_12G007210	....
gene-COCNU_12G007220	....
gene-COCNU_12G007230	....
gene-GSCOC_T00029776001	....
gene-IFM89_035037	....
gene-IFM89_035040	....
gene-CCACVL1_07583	....
Cav07g20270	....
Cav07g20290	CALRSAVLKTLDFRSSPKDKESPFEKFTRVTMILPLTGQQYSEKVSENCVAFWKSVGIYT
gene-BT93_F1001	....
MELO3C025484.2	....
Csa_5G505170	....
gene-DM860_013525	....
geneJL025816	....
geneJL015184	....
CHI_2	....
Dinv31395	....
CHI	....
CHI_2	....
CHI_3	....
gene-KFK09_000997	....
gene-BAE44_0007788	....
gene-BAE44_0020983	....
Dexi3A01G0013540	....
Dexi3B01G0013780	....
D.long004984	....
gene-IHE45_09G071500	....
DRNTG_23933	....
gene-J5N97_002300	....
gene-LOC111309827	....
scaffold13.535	....
scaffold176.249	....
scaffold36.570	....
scaffold3.658	....
scaffold65.276	....
gene-LOC105035984	....
gene-PR202_ga11488	....
gene-PR202_ga13968	....
gene-PR202_gb10465	....
gene-EJB05_03819	....
Et_4A_034194	....
Et_4B_038342	....
Et_6A_046252	....
Et_9A_062905	....
Et_9B_065446	....
EUGRSUZ_F03816	....
EVM20prediction20Chr12.1151	....
Casp15914	....
EUTSA_v10004922mg	....
EUTSA_v10010658mg	....
EVM_prediction_Fe1.6811	....
EVM_prediction_Fe3.10426	....
EVM_prediction_Fe4.5584	....
EVM_prediction_Fe5.14515	....
EVM_prediction_Fe5.14516	....
FCD_00011569	....
FCD_00011572	....
FCD_00011567	....
FCD_00011574	....
maker-Fvb7-2-augustus-gene-221.49	....
maker-Fvb7-3-augustus-gene-78.40	....
EVM20prediction20scaffold_9.28	....
gene-GLYMA_20G241700v4	....
TnS000078355t12	....
TnS000589875t03	....
gene-EPI10_010037	....
GG16G068580	....
GG16G068530	....
gene-CDL12_22415	....
gene-CDL12_26251	....
gene-LOC110910959	....
gene-LOC110913037	....
gene-LOC110913039	....
gene-LOC110425216	....
gene-P3X46_014162	....
gene-ZWY2020_018680	....
Hyque.04G056200.v1.1	....
gene-LOC109186979	....
itb05g04360	....
gene-M6B38_184735	....
gene-M6B38_299855	....
gene-M6B38_364490	....
gene-M6B38_399615	....
Jmimo22322	....
HT24166	....
Joasc.10G103600.v1.1	....
Jr07_35750	....

**Medicago**	....
**CICLE_v10032697mg**	....
**gene-COCNU_12G007210**	....
**gene-COCNU_12G007220**	....
**gene-COCNU_12G007230**	....
**gene-GSCOC_T00029776001**	....
**gene-IFM89_035037**	....
**gene-IFM89_035040**	....
**gene-CCACVL1_07583**	....
**Cav07g20270**	....
**Cav07g20290**	DAEAKAIEKFIEVFKDETFPPGSSILFTQSPNGSLTISFSKDGSLPEIGNAVIENKLLSE
**gene-BT93_F1001**	....
**MELO3C025484.2**	....
**Csa_5G505170**	....
**gene-DM860_013525**	....
**geneJL025816**	....
**geneJL015184**	....
**CHI_2**	....
**Dinv31395**	....
**CHI**	....
**CHI_2**	....
**CHI_3**	....
**gene-KFK09_000997**	....
**gene-BAE44_0007788**	....
**gene-BAE44_0020983**	....
**Dexi3A01G0013540**	....
**Dexi3B01G0013780**	....
**D.long004984**	....
**gene-IHE45_09G071500**	....
**DRNTG_23933**	....
**gene-J5N97_002300**	....
**gene-LOC111309827**	....
**scaffold13.535**	....
**scaffold176.249**	....
**scaffold36.570**	....
**scaffold3.658**	....
**scaffold65.276**	....
**gene-LOC105035984**	....
**gene-PR202_ga11488**	....
**gene-PR202_ga13968**	....
**gene-PR202_gb10465**	....
**gene-EJB05_03819**	....
**Et_4A_034194**	....
**Et_4B_038342**	....
**Et_6A_046252**	....
**Et_9A_062905**	....
**Et_9B_065446**	....
**EUGRSUZ_F03816**	....
**EVM20prediction20Chr12.1151**	....
**Casp15914**	....
**EUTSA_v10004922mg**	....
**EUTSA_v10010658mg**	....
**EVM_prediction_Fe1.6811**	....
**EVM_prediction_Fe3.10426**	....
**EVM_prediction_Fe4.5584**	....
**EVM_prediction_Fe5.14515**	....
**EVM_prediction_Fe5.14516**	....
**FCD_00011569**	....
**FCD_00011572**	....
**FCD_00011567**	....
**FCD_00011574**	....
**maker-Fvb7-2-augustus-gene-221.49**	....
**maker-Fvb7-3-augustus-gene-78.40**	....
**EVM20prediction20scaffold_9.28**	....
**gene-GLYMA_20G241700v4**	....
**TnS000078355t12**	....
**TnS000589875t03**	....
**gene-EPI10_010037**	....
**GG16G068580**	....
**GG16G068530**	....
**gene-CDL12_22415**	....
**gene-CDL12_26251**	....
**gene-LOC110910959**	....
**gene-LOC110913037**	....
**gene-LOC110913039**	....
**gene-LOC110425216**	....
**gene-P3X46_014162**	....
**gene-ZWY2020_018680**	....
**Hyque.04G056200.v1.1**	....
**gene-LOC109186979**	....
**itb05g04360**	....
**gene-M6B38_184735**	....
**gene-M6B38_299855**	....
**gene-M6B38_364490**	....
**gene-M6B38_399615**	....
**Jmimo22322**	....
**HT24166**	....
**Joasc.10G103600.v1.1**	....
**Jr07_35750**	....

```
                                                                220
Medicago                             ...........................LNEGAF.K.I....GN.................
CICLE_v10032697mg                    ...........................LNVTSD.K.M...................K...
gene-COCNU_12G007210                 ...........................LKESQD.V.E....E...K.VA.......NP..
gene-COCNU_12G007220                 ...........................LKESQD.V.E....E...K.VG.......NP..
gene-COCNU_12G007230                 ...........................LKESQD.V.E....E...K.VG.......NP..
gene-GSCOC_T00029776001              ...........................LKVFDN.N.N....NNVTA.DN...........
gene-IFM89_035037                    ...........................FKVEEQ.K.A....AKC................
gene-IFM89_035040                    ...........................LRNEDG.E.AVEYIKEGDL..............
gene-CCACVL1_07583                   ...........................FNGATV.N.G....KTESE..............
Cav07g20270                          ...........................LKDNDD.K.V....VGNG...........K...
Cav07g20290                          AILESIIGKHGVSPEAKQSLATRLAESLKDNDD.K.V....VGNG...........K...
gene-BT93_F1001                      ...........................LKDCEK.K.A....DEVDK.NA.....APKADK
MELO3C025484.2                       ...........................LTQSQP.N.T....PNICN......PVQNPNSK
Csa_5G505170                         ...........................FNLSQP.N.I....SNPFQ......DPKSEPNR
gene-DM860_013525                    ...........................LKKGKE.I.G....EENV...............
geneJL025816                         ...........................LNKKEE.E.E..................EGI..
geneJL015184                         ...........................LNKKEE.E.E....EEEE..........EGI..
CHI_2                                ...........................LKHHDH.E.V....TGNGK..............
Dinv31395                            ...........................LKECDD.K.V....PGKAT..............
CHI                                  ...........................LNKKEE.K.E....KEEEK.........EEI..
CHI_2                                ...........................LNKKEE.K.E....EEEEE.EEE....KEEI..
CHI_3                                ...........................LNKKEE.E.E....EE...........KEEI..
gene-KFK09_000997                    ...........................LNKKEE.K.E....KEEEK.........EEI..
gene-BAE44_0007788                   ...........................LKLKGA.K.Q....AGDAP.QAEPA........
gene-BAE44_0020983                   ...........................LKGGGG.A.........................
Dexi3A01G0013540                     ...........................LKGGA............................
Dexi3B01G0013780                     ...........................LKGGA............................
D.long004984                         ...........................LNDKEP.E.G....GE.................
gene-IHE45_09G071500                 ...........................LSEYN............................
DRNTG_23933                          ...........................LSEYN............................
gene-J5N97_002300                    ...........................LLESKD.V.........................
gene-LOC111309827                    ...........................FNDCGE.K.A....AADNG..........K...
scaffold13.535                       ...........................LKSVA............................
scaffold176.249                      ...........................LEGATA.A.....................NP..
scaffold36.570                       ...........................LQGGA............................
scaffold3.658                        ...........................LEGTTA.N.P....AGDAP.QA.......EH..
scaffold65.276                       ...........................LNGATA.N.P....AGDGP.QA.......EP..
gene-LOC105035984                    ...........................LKESQD.V.E....E...K.VG.......NP..
gene-PR202_ga11488                   ...........................LNRA.............................
gene-PR202_ga13968                   ...........................LKENTR........GGDAPLQA.......EP..
gene-PR202_gb10465                   ...........................LKETTP........GGDAPPQA.......EP..
gene-EJB05_03819                     ...........................LKGTTS.N.P....G.DAP.QA.......EP..
Et_4A_034194                         ...........................LKGTTT.T.P....GDDAP.QA.......EP..
Et_4B_038342                         ...........................LKETST.T.P....G.DAP.QA.......EP..
Et_6A_046252                         ...........................LNGTST.T.A....G.DAL.QA.......D...
Et_9A_062905                         ...........................LKSPA............................
Et_9B_065446                         ...........................LKSPA............................
EUGRSUZ_F03816                       ...........................LKDCEK.K.A....DEVDK.IA.....APKAAA
EVM20prediction20Chr12.1151          ...........................LSQYAD.K.T....TKNEI.VET....NTKD..
Casp15914                            .................................................................
EUTSA_v10004922mg                    ...........................S................................
EUTSA_v10010658mg                    ...........................MKKNKV.E.E....EEEAT.RTD....QEEATD
EVM_prediction_Fe1.6811              ............................S...............................
EVM_prediction_Fe3.10426             .................................................................
EVM_prediction_Fe4.5584              ...........................IQTNKE..DVI..SS..................
EVM_prediction_Fe5.14515             ...........................LNPSNG.E.A....QTKEI.ASI..........
EVM_prediction_Fe5.14516             ...........................LNPTKV.A.N....GEAEI.KVA....NGEAKA
FCD_00011569                         ...........................LIDQNK.I.P....HHLPY........GEKKMS
FCD_00011572                         ...........................LIDQNK.I.P....HHLPY........GEKKMS
FCD_00011567                         ...........................FKAKAN.SVA.......................
FCD_00011574                         ...........................LMDQNK.T.H....AV.........PSGEKKLL
maker-Fvb7-2-augustus-gene-221.49    ...........................LKDSDH.C.V....AGNG...........K...
maker-Fvb7-3-augustus-gene-78.40     ...........................LKESDH.C.V....AGNG...........K...
EVM20prediction20scaffold_9.28       ...........................LSKEEK...........................
gene-GLYMA_20G241700v4               ...........................FKEPGVC..D....PQSHK..............
TnS000078355t12                      ...........................FSNSN............................
TnS000589875t03                      ...........................FSDNQH.A.S.......................
gene-EPI10_010037                    ...........................FNDCGA.D.S....EK.................
GG16G068580                          ...........................LKKEDI.K.S....AEEEA.QD...........
GG16G068530                          ...........................YKERRR.R.S....SRRIC.RREIS..HGELR.
gene-CDL12_22415                     ...........................LKQYE............................
gene-CDL12_26251                     ...........................LKQYE............................
gene-LOC110910959                    ...........................MNQKAN.D.H.......................
gene-LOC110913037                    ...........................MTSEAA.T.L....SENGL..............
gene-LOC110913039                    ...........................MNQKAN.N.........................
gene-LOC110425216                    ...........................FDDSVE.K.A....AQNG...........K...
gene-P3X46_014162                    ...........................FEKNAE.I.N....GTNH...........KLST
gene-ZWY2020_018680                  ...........................LKGAA....H....AGGEP.AA.......EP..
Hyque.04G056200.v1.1                 ...........................FGSDDK.V.A....GKTI...........P...
gene-LOC109186979                    ...........................FMNGGD.A.I....SGKVG.G............
itb05g04360                          ...........................FKNGIN.G.G....DAISG.KVG..........
gene-M6B38_184735                    ...........................L................................
gene-M6B38_299855                    ...........................LNQVQS.G.Q....T..................
gene-M6B38_364490                    ...........................L................................
gene-M6B38_399615                    ...........................LNQVQS.G.Q....T..................
Jmimo22322                           ...........................LKQYEP.T.A.......................
HT24166                              ...........................FKQELE.S.N....APNQG.L............
Joasc.10G103600.v1.1                 ...........................LKESPV.T.G....VAKMP..............
Jr07_35750                           ...........................LKESND.K.E....AENH...........K...
```

**Medicago**	....
**CICLE_v10032697mg**	....
**gene-COCNU_12G007210**	........VAVIA...
**gene-COCNU_12G007220**	........VAVIA...
**gene-COCNU_12G007230**	........VAVIA...
**gene-GSCOC_T00029776001**	........KKLEADGA...IAAEAPGEKQVNGVQVP........................
**gene-IFM89_035037**	....
**gene-IFM89_035040**	...................LKAENGEGT................................
**gene-CCACVL1_07583**	....
**Cav07g20270**	....
**Cav07g20290**	....
**gene-BT93_F1001**	........VIVAESNG....GAEDDGNKPGVVEEK..............VELNGR.....
**MELO3C025484.2**	........PHLNN...
**Csa_5G505170**	........IHLST...
**gene-DM860_013525**	....
**geneJL025816**	........LAVEKG......KFEQVEVA................................
**geneJL015184**	........LAVEKG......KFEQVEVA................................
**CHI_2**	........AEVGKMASK.DTETETQVQV................................
**Dinv31395**	....
**CHI**	........LVVEKG......KLEQVEVA................................
**CHI_2**	........LVLEKG......KWSKLRLLDCSLIGSM........................
**CHI_3**	........LVLEKG......KLEQVEVA................................
**gene-KFK09_000997**	........LVVEKG......KLEQVEVA................................
**gene-BAE44_0007788**	........VPVSASAQR...
**gene-BAE44_0020983**	....
**Dexi3A01G0013540**	....
**Dexi3B01G0013780**	....
**D.long004984**	..........AEA.........EKCVKE................................
**gene-IHE45_09G071500**	....
**DRNTG_23933**	....
**gene-J5N97_002300**	....
**gene-LOC111309827**	..........PESK..
**scaffold13.535**	....
**scaffold176.249**	..........VSA...
**scaffold36.570**	....
**scaffold3.658**	........VPVSA...
**scaffold65.276**	........VPISA...
**gene-LOC105035984**	........VIVSA...
**gene-PR202_ga11488**	....
**gene-PR202_ga13968**	........VPASA...
**gene-PR202_gb10465**	........VPASA...
**gene-EJB05_03819**	........VAVSS...
**Et_4A_034194**	........VAVSS...
**Et_4B_038342**	........VAVSS...
**Et_6A_046252**	........VAISS...
**Et_9A_062905**	....
**Et_9B_065446**	....
**EUGRSUZ_F03816**	........AKVDVA..
**EVM20prediction20Chr12.1151**	........VSAETTMV.....LENGGVEDVGQVKNG........................
**Casp15914**	....
**EUTSA_v10004922mg**	....
**EUTSA_v10010658mg**	........VSLGDKLA.....KEN....................................
**EVM_prediction_Fe1.6811**	....
**EVM_prediction_Fe3.10426**	....
**EVM_prediction_Fe4.5584**	....
**EVM_prediction_Fe5.14515**	....
**EVM_prediction_Fe5.14516**	E.......TKVANGKAE...TKENGVEIKE..............................
**FCD_00011569**	SVG.....GNM...
**FCD_00011572**	SGG.....GNMQQGLQLI...RESKFHTDMLS............................
**FCD_00011567**	....
**FCD_00011574**	AV..
**maker-Fvb7-2-augustus-gene-221.49**	..........VDEC........TKEAEV................................
**maker-Fvb7-3-augustus-gene-78.40**	..........VDEC........TKEAEV................................
**EVM20prediction20scaffold_9.28**	....
**gene-GLYMA_20G241700v4**	....
**TnS000078355t12**	....
**TnS000589875t03**	....
**gene-EPI10_010037**	..........PQS...
**GG16G068580**	........ASVGEKSA.....MEN....................................
**GG16G068530**	........TQISYWM...
**gene-CDL12_22415**	....
**gene-CDL12_26251**	....
**gene-LOC110910959**	....
**gene-LOC110913037**	....
**gene-LOC110913039**	....
**gene-LOC110425216**	..........PECQ..
**gene-P3X46_014162**	ISPAVTSVSQIEV.........ETVAFPPAVKPPASNKTLFLGGAGARGLEIQGKFVKFT
**gene-ZWY2020_018680**	........VPVSV...
**Hyque.04G056200.v1.1**	......VAVEPEG.........ESVKTV................................
**gene-LOC109186979**	......................ENDVIPQTVVVSK.........................
**itb05g04360**	.....................CENDAIPQAVV.SK.........................
**gene-M6B38_184735**	....
**gene-M6B38_299855**	........VNA...
**gene-M6B38_364490**	....
**gene-M6B38_399615**	........VNA...
**Jmimo22322**	....
**HT24166**	....
**Joasc.10G103600.v1.1**	........MSVSV...
**Jr07_35750**	..........LKA.........ENQKLE................................

```
Medicago                              ............................................................
CICLE_v10032697mg                     ............................................................
gene-COCNU_12G007210                  ............................................................
gene-COCNU_12G007220                  ............................................................
gene-COCNU_12G007230                  ............................................................
gene-GSCOC_T00029776001               ............................................................
gene-IFM89_035037                     ............................................................
gene-IFM89_035040                     ............................................................
gene-CCACVL1_07583                    ............................................................
Cav07g20270                           ............................................................
Cav07g20290                           ............................................................
gene-BT93_F1001                       ............................................................
MELO3C025484.2                        ............................................................
Csa_5G505170                          ............................................................
gene-DM860_013525                     ............................................................
geneJL025816                          ............................................................
geneJL015184                          ............................................................
CHI_2                                 ............................................................
Dinv31395                             ............................................................
CHI                                   ............................................................
CHI_2                                 ............................................................
CHI_3                                 ............................................................
gene-KFK09_000997                     ............................................................
gene-BAE44_0007788                    ............................................................
gene-BAE44_0020983                    ............................................................
Dexi3A01G0013540                      ............................................................
Dexi3B01G0013780                      ............................................................
D.long004984                          ............................................................
gene-IHE45_09G071500                  ............................................................
DRNTG_23933                           ............................................................
gene-J5N97_002300                     ............................................................
gene-LOC111309827                     ............................................................
scaffold13.535                        ............................................................
scaffold176.249                       ............................................................
scaffold36.570                        ............................................................
scaffold3.658                         ............................................................
scaffold65.276                        ............................................................
gene-LOC105035984                     ............................................................
gene-PR202_ga11488                    ............................................................
gene-PR202_ga13968                    ............................................................
gene-PR202_gb10465                    ............................................................
gene-EJB05_03819                      ............................................................
Et_4A_034194                          ............................................................
Et_4B_038342                          ............................................................
Et_6A_046252                          ............................................................
Et_9A_062905                          ............................................................
Et_9B_065446                          ............................................................
EUGRSUZ_F03816                        ............................................................
EVM20prediction20Chr12.1151           ..................................DI........................
Casp15914                             ............................................................
EUTSA_v10004922mg                     ............................................................
EUTSA_v10010658mg                     ............................................................
EVM_prediction_Fe1.6811               ............................................................
EVM_prediction_Fe3.10426              ............................................................
EVM_prediction_Fe4.5584               ............................................................
EVM_prediction_Fe5.14515              ............................................................
EVM_prediction_Fe5.14516              ............................................................
FCD_00011569                          ............................................................
FCD_00011572                          ............................................................
FCD_00011567                          ............................................................
FCD_00011574                          ............................................................
maker-Fvb7-2-augustus-gene-221.49     ............................................................
maker-Fvb7-3-augustus-gene-78.40      ............................................................
EVM20prediction20scaffold_9.28        ............................................................
gene-GLYMA_20G241700v4                ............................................................
TnS000078355t12                       ............................................................
TnS000589875t03                       ............................................................
gene-EPI10_010037                     ............................................................
GG16G068580                           ............................................................
GG16G068530                           ............................................................
gene-CDL12_22415                      ............................................................
gene-CDL12_26251                      ............................................................
gene-LOC110910959                     ............................................................
gene-LOC110913037                     ............................................................
gene-LOC110913039                     ............................................................
gene-LOC110425216                     ............................................................
gene-P3X46_014162                     AIGVYLEDEAVPVLAVRWKGKSGKELTDSVEFFRDIVTGPFEKFIRVTMILPLTGQQYSE
gene-ZWY2020_018680                   ............................................................
Hyque.04G056200.v1.1                  ............................................................
gene-LOC109186979                     ............................................................
itb05g04360                           ............................................................
gene-M6B38_184735                     ............................................................
gene-M6B38_299855                     ............................................................
gene-M6B38_364490                     ............................................................
gene-M6B38_399615                     ............................................................
Jmimo22322                            ............................................................
HT24166                               ............................................................
Joasc.10G103600.v1.1                  ............................................................
Jr07_35750                            ............................................................
```

Medicago	....
CICLE_v10032697mg	....
gene-COCNU_12G007210	....
gene-COCNU_12G007220	....
gene-COCNU_12G007230	....
gene-GSCOC_T00029776001	....VQVP............
gene-IFM89_035037	....
gene-IFM89_035040	....
gene-CCACVL1_07583	....
Cav07g20270	....
Cav07g20290	....
gene-BT93_F1001	....TSAP............
MELO3C025484.2	....
Csa_5G505170	....
gene-DM860_013525	....
geneJL025816	....
geneJL015184	....
CHI_2	....
Dinv31395	....
CHI	....
CHI_2	....EFA
CHI_3	....
gene-KFK09_000997	....
gene-BAE44_0007788	....
gene-BAE44_0020983	....
Dexi3A01G0013540	....
Dexi3B01G0013780	....
D.long004984	........................DKAVEL.............................G
gene-IHE45_09G071500	....
DRNTG_23933	....
gene-J5N97_002300	....
gene-LOC111309827	....
scaffold13.535	....
scaffold176.249	....
scaffold36.570	....
scaffold3.658	....
scaffold65.276	....
gene-LOC105035984	....
gene-PR202_ga11488	....
gene-PR202_ga13968	....
gene-PR202_gb10465	....
gene-EJB05_03819	....
Et_4A_034194	....
Et_4B_038342	....
Et_6A_046252	....
Et_9A_062905	....
Et_9B_065446	....
EUGRSUZ_F03816	....
EVM20prediction20Chr12.1151	....
Casp15914	....
EUTSA_v10004922mg	....
EUTSA_v10010658mg	....
EVM_prediction_Fe1.6811	....
EVM_prediction_Fe3.10426	....
EVM_prediction_Fe4.5584	....
EVM_prediction_Fe5.14515	....
EVM_prediction_Fe5.14516	....
FCD_00011569	....
FCD_00011572	....
FCD_00011567	....
FCD_00011574	....
maker-Fvb7-2-augustus-gene-221.49	........................KA..................................
maker-Fvb7-3-augustus-gene-78.40	........................KA..................................
EVM20prediction20scaffold_9.28	....
gene-GLYMA_20G241700v4	....
TnS000078355t12	....
TnS000589875t03	....
gene-EPI10_010037	....
GG16G068580	....
GG16G068530	....
gene-CDL12_22415	....
gene-CDL12_26251	....
gene-LOC110910959	....
gene-LOC110913037	....
gene-LOC110913039	....
gene-LOC110425216	....
gene-P3X46_014162	KVSENCVAIWKSLGIYTDAEAKAIDKFLEIFKAETFPPGSSILFTLLPHGALAITFSKDG
gene-ZWY2020_018680	....
Hyque.04G056200.v1.1	....
gene-LOC109186979	....
itb05g04360	....
gene-M6B38_184735	....
gene-M6B38_299855	....
gene-M6B38_364490	....
gene-M6B38_399615	....
Jmimo22322	....
HT24166	....
Joasc.10G103600.v1.1	....
Jr07_35750	..TEN.........LKLKAENHKLETENKIL..........................KAE

**Medicago**	....
**CICLE_v10032697mg**	....
**gene-COCNU_12G007210**	....
**gene-COCNU_12G007220**	....
**gene-COCNU_12G007230**	....
**gene-GSCOC_T00029776001**	....
**gene-IFM89_035037**	....
**gene-IFM89_035040**	.VPEMEN..
**gene-CCACVL1_07583**	....
**Cav07g20270**	....
**Cav07g20290**	....
**gene-BT93_F1001**	....
**MELO3C025484.2**	....
**Csa_5G505170**	....
**gene-DM860_013525**	....
**geneJL025816**	....
**geneJL015184**	....
**CHI_2**	....
**Dinv31395**	....
**CHI**	....
**CHI_2**	ASPPVKKVRIETGDVTIAVDELVVAPNDYLL.IVNPLAGHTGVTIITDGYNDPMIL...G
**CHI_3**	....
**gene-KFK09_000997**	....
**gene-BAE44_0007788**	.....................................TGRDGICR...............
**gene-BAE44_0020983**	....
**Dexi3A01G0013540**	....
**Dexi3B01G0013780**	....
**D.long004984**	NDIKVA..
**gene-IHE45_09G071500**	....
**DRNTG_23933**	....
**gene-J5N97_002300**	....
**gene-LOC111309827**	....
**scaffold13.535**	....
**scaffold176.249**	....
**scaffold36.570**	....
**scaffold3.658**	....
**scaffold65.276**	....
**gene-LOC105035984**	....
**gene-PR202_ga11488**	....
**gene-PR202_ga13968**	....
**gene-PR202_gb10465**	....
**gene-EJB05_03819**	....
**Et_4A_034194**	....
**Et_4B_038342**	....
**Et_6A_046252**	....
**Et_9A_062905**	....
**Et_9B_065446**	....
**EUGRSUZ_F03816**	....
**EVM20prediction20Chr12.1151**	....
**Casp15914**	....
**EUTSA_v10004922mg**	....
**EUTSA_v10010658mg**	....
**EVM_prediction_Fe1.6811**	....
**EVM_prediction_Fe3.10426**	....
**EVM_prediction_Fe4.5584**	....
**EVM_prediction_Fe5.14515**	....
**EVM_prediction_Fe5.14516**	....
**FCD_00011569**	....
**FCD_00011572**	...............................L............................
**FCD_00011567**	....
**FCD_00011574**	....
**maker-Fvb7-2-augustus-gene-221.49**	....
**maker-Fvb7-3-augustus-gene-78.40**	....
**EVM20prediction20scaffold_9.28**	............................YT..............................
**gene-GLYMA_20G241700v4**	....
**TnS000078355t12**	....
**TnS000589875t03**	....
**gene-EPI10_010037**	....
**GG16G068580**	....
**GG16G068530**	....
**gene-CDL12_22415**	....
**gene-CDL12_26251**	....
**gene-LOC110910959**	....
**gene-LOC110913037**	....
**gene-LOC110913039**	....
**gene-LOC110425216**	....
**gene-P3X46_014162**	SIPEVESTMIENKLLSEAVLE..............SIIGKHGVSPATRETMATRLAELF.
**gene-ZWY2020_018680**	....
**Hyque.04G056200.v1.1**	...EKENGKL..
**gene-LOC109186979**	....
**itb05g04360**	....
**gene-M6B38_184735**	....
**gene-M6B38_299855**	....
**gene-M6B38_364490**	....
**gene-M6B38_399615**	....
**Jmimo22322**	....
**HT24166**	....
**Joasc.10G103600.v1.1**	....
**Jr07_35750**	N.SEIGGPK...

**Medicago**	. . .
**CICLE_v10032697mg**	. . .
**gene-COCNU_12G007210**	. . .
**gene-COCNU_12G007220**	. . .
**gene-COCNU_12G007230**	. . .
**gene-GSCOC_T00029776001**	. . .
**gene-IFM89_035037**	. . .
**gene-IFM89_035040**	. . .
**gene-CCACVL1_07583**	. . .
**Cav07g20270**	. . .
**Cav07g20290**	. . .
**gene-BT93_F1001**	A A V
**MELO3C025484.2**	. . .
**Csa_5G505170**	. . .
**gene-DM860_013525**	. . .
**geneJL025816**	. . .
**geneJL015184**	. . .
**CHI_2**	. . .
**Dinv31395**	. . .
**CHI**	. . .
**CHI_2**	K L R
**CHI_3**	. . .
**gene-KFK09_000997**	. . .
**gene-BAE44_0007788**	S N M
**gene-BAE44_0020983**	. . .
**Dexi3A01G0013540**	. . .
**Dexi3B01G0013780**	. . .
**D.long004984**	. . .
**gene-IHE45_09G071500**	. . .
**DRNTG_23933**	. . .
**gene-J5N97_002300**	. . .
**gene-LOC111309827**	. . .
**scaffold13.535**	. . .
**scaffold176.249**	. . .
**scaffold36.570**	. . .
**scaffold3.658**	. . .
**scaffold65.276**	. . .
**gene-LOC105035984**	. . .
**gene-PR202_ga11488**	. . .
**gene-PR202_ga13968**	. . .
**gene-PR202_gb10465**	. . .
**gene-EJB05_03819**	. . .
**Et_4A_034194**	. . .
**Et_4B_038342**	. . .
**Et_6A_046252**	. . .
**Et_9A_062905**	. . .
**Et_9B_065446**	. . .
**EUGRSUZ_F03816**	. . .
**EVM20prediction20Chr12.1151**	. . .
**Casp15914**	. . .
**EUTSA_v10004922mg**	. . .
**EUTSA_v10010658mg**	. . .
**EVM_prediction_Fe1.6811**	. . .
**EVM_prediction_Fe3.10426**	. . .
**EVM_prediction_Fe4.5584**	. . .
**EVM_prediction_Fe5.14515**	. . .
**EVM_prediction_Fe5.14516**	. . .
**FCD_00011569**	. . .
**FCD_00011572**	. . .
**FCD_00011567**	. . .
**FCD_00011574**	. . .
**maker-Fvb7-2-augustus-gene-221.49**	. . .
**maker-Fvb7-3-augustus-gene-78.40**	. . .
**EVM20prediction20scaffold_9.28**	. . .
**gene-GLYMA_20G241700v4**	. . .
**TnS000078355t12**	. . .
**TnS000589875t03**	. . F
**gene-EPI10_010037**	. . .
**GG16G068580**	. . .
**GG16G068530**	. . .
**gene-CDL12_22415**	. . .
**gene-CDL12_26251**	. . .
**gene-LOC110910959**	. . .
**gene-LOC110913037**	. . .
**gene-LOC110913039**	. . .
**gene-LOC110425216**	. . .
**gene-P3X46_014162**	. . I
**gene-ZWY2020_018680**	. . .
**Hyque.04G056200.v1.1**	. . .
**gene-LOC109186979**	. . .
**itb05g04360**	. . .
**gene-M6B38_184735**	. . M
**gene-M6B38_299855**	. . .
**gene-M6B38_364490**	. . M
**gene-M6B38_399615**	. . .
**Jmimo22322**	. . .
**HT24166**	. . .
**Joasc.10G103600.v1.1**	. . .
**Jr07_35750**	. . .

```
Medicago                        ............................................................
Jr07_35760                      ............................................................
Kaladp0060s0328.v1.1            ............................................................
geneMaker00000028               ............................................................
gene-GIB67_014039               ............................................................
gene-GIB67_016345               ............................................................
gene-GIB67_037506               ............................................................
gene-GIB67_041777               ............................................................
gene-FCM35_KLT21231             ............................................................
Lsat_1_v5_gn_9_66221.v5         ............................................................
LPERR03G33170                   ............................................................
Liphi.11G022900.v1.1            ............................................................
Lchi23460                       ............................................................
Lchi33204                       ............................................................
Lchi33866                       ............................................................
Lchi34914                       ............................................................
gene-Leryth_002794              ............................................................
Spe07827                        ............................................................
Spe20163                        .....................M......................................
KYUSg_chr4.5001                 ............................................................
gene-LOC124680986               ............................................................
gene-LOC124695032               ............................................................
Lj1A930T84                      ............................................................
Luann.0362s0045.v1.1            ............................................................
Luann.0427s0040.v1.1            ............................................................
TanjilG_30460                   ............................................................
TanjilG_24711                   ............................................................
gene-LOC122057215               ............................................................
gene-BVC80_1651g70              ............................................................
gene-LOC131167055               .....................MTTT...................................
gene-LOC131167056               ............................................................
Mamar.0037s0511.v1.1            .....................M......................................
Mamar.0043s0032.v1.1            .....................MSLT...................................
MD01G0093100                    MRNISVVLLCLIQINKYGCRLMAPTPSVAG..............................
MD01G0093200                    MH..........................................................
MD01G0093300                    ............................................................
MD07G0154300                    ............................................................
MD07G0154400                    ............................................................
gene-LOC123192628               ............................................................
gene-LOC123227022               ............................................................
gene-MANES_07G107200v8          ............................................................
gene-LOC11445536                ............................................................
gene-LOC11446745                ............................................................
Mde013561.1                     ............................................................
gene-MLD38_024177               ............................................................
gene-MERR_LOCUS28634            ............................................................
Migut.D00159.v2.0               .....................MS.....................................
gene-LOC111006117               ............................................................
MoBGI036118g0111                ............................................................
gene-L484_017264                ............................................................
gene-LOC103981836               ............................................................
gene-LOC104594676               .....................MAPRLLL................................
gene-LOC104599237               ............................................................
CHI                             ............................................................
NC6G0256760                     .....................MLFGGWGGD..............................
gene-F0562_031805               ............................................................
ORUFI03G40690                   ............................................................
ORUFI05G23340                   ............................................................
protein_coding_16416            ............................................................
gene-PAHAL_3G142600             ............................................................
gene-PAHAL_9G032500             ............................................................
C5167_000623                    ............................................................
C5167_044917                    ............................................................
C5167_046772                    .....................MN.....................................
gene-PanWU01x14_043220          ............................................................
gene-BS78_01G031900             ............................................................
gene-BS78_01G032000             ............................................................
gene-C2S52_017353               ............................................................
gene-LOC110026679               ............................................................
Phala.01G033500.v1.1            ............................................................
gene-PHAVU_002G276500g          .....................M......................................
gene-PHAVU_007G008500g          ............................................................
gene-LOC103696411               ............................................................
gene-LOC108511484               ............................................................
gene-PHJA_002477300             ............................................................
PIPE19196                       ............................................................
PIPE22647                       ............................................................
gene-LOC116107972               ............................................................
gene-LOC116107975               ............................................................
Psat6g237840                    ............................................................
Ptrif.0004s0460.v1.3.1          ............................................................
gene-LOC118059901               ............................................................
Potri.010G213000.v4.1           ............................................................
FUN_004111.v1.0                 ............................................................
PvLHv1_097350                   .....................MCSMDHCNPREFLFFSLSLGLLLLTSEGIVPTSCSSRVT
Pav_sc0007510.1_g020.1.mk       ............................................................
Prudul26B016396                 ............................................................
PRUPE_2G225200                  ............................................................
gene-LOC116194939               .....................MAQS...................................
```

```
Medicago                      ............................................................
Jr07_35760                    ............................................................
Kaladp0060s0328.v1.1          ............................................................
geneMaker00000028             ............................................................
gene-GIB67_014039             ............................................................
gene-GIB67_016345             ............................................................
gene-GIB67_037506             ............................................................
gene-GIB67_041777             ............................................................
gene-FCM35_KLT21231           ............................................................
Lsat_1_v5_gn_9_66221.v5       ............................................................
LPERR03G33170                 ............................................................
Liphi.11G022900.v1.1          ............................................................
Lchi23460                     ............................................................
Lchi33204                     ............................................................
Lchi33866                     ............................................................
Lchi34914                     ............................................................
gene-Leryth_002794            ............................................................
Spe07827                      ............................................................
Spe20163                      ............................................................
KYUSg_chr4.5001               ............................................................
gene-LOC124680986             ............................................................
gene-LOC124695032             ............................................................
Lj1A930T84                    ............................................................
Luann.0362s0045.v1.1          ............................................................
Luann.0427s0040.v1.1          ............................................................
TanjilG_30460                 ............................................................
TanjilG_24711                 ............................................................
gene-LOC122057215             ............................................................
gene-BVC80_1651g70            ............................................................
gene-LOC131167055             ............................................................
gene-LOC131167056             ............................................................
Mamar.0037s0511.v1.1          ............................................................
Mamar.0043s0032.v1.1          ............................................................
MD01G0093100                  .LQVETTAFPPSVKPPGSSNTLFLGGAGVRGLEI.QGNFVKFTAIGVYLEDNVVPQLAVK
MD01G0093200                  ............................................................
MD01G0093300                  ............................................................
MD07G0154300                  ............................................................
MD07G0154400                  ............................................................
gene-LOC123192628             ............................................................
gene-LOC123227022             ............................................................
gene-MANES_07G107200v8        ............................................................
gene-LOC11445536              ............................................................
gene-LOC11446745              ............................................................
Mde013561.1                   ............................................................
gene-MLD38_024177             ............................................................
gene-MERR_LOCUS28634          ............................................................
Migut.D00159.v2.0             ............................................................
gene-LOC111006117             ............................................................
MoBGI036118g0111              ............................................................
gene-L484_017264              ............................................................
gene-LOC103981836             ............................................................
gene-LOC104594676             ...............................LTN.QLPLVLF..................
gene-LOC104599237             ............................................................
CHI                           ............................................................
NC6G0256760                   .......................VGFAGVRGLNI.QGTFVKFTAIGVYVEAAAVDALRPK
gene-F0562_031805             ............................................................
ORUFI03G40690                 ............................................................
ORUFI05G23340                 ............................................................
protein_coding_16416          ............................................................
gene-PAHAL_3G142600           ............................................................
gene-PAHAL_9G032500           ............................................................
C5167_000623                  ............................................................
C5167_044917                  ............................................................
C5167_046772                  ............................................................
gene-PanWU01x14_043220        ............................................................
gene-BS78_01G031900           ............................................................
gene-BS78_01G032000           ............................................................
gene-C2S52_017353             ............................................................
gene-LOC110026679             ............................................................
Phala.01G033500.v1.1          ............................................................
gene-PHAVU_002G276500g        ..................................LDGATFHF..................
gene-PHAVU_007G008500g        ............................................................
gene-LOC103696411             ............................................................
gene-LOC108511484             ............................................................
gene-PHJA_002477300           ............................................................
PIPE19196                     ............................................................
PIPE22647                     ............................................................
gene-LOC116107972             ............................................................
gene-LOC116107975             ............................................................
Psat6g237840                  ............................................................
Ptrif.0004s0460.v1.3.1        ............................................................
gene-LOC118059901             ............................................................
Potri.010G213000.v4.1         ............................................................
FUN_004111.v1.0               ............................................................
PvLHv1_097350                 SVQVEGHVFPATVKAPGSTASFFLGGAGVRGLDI.NGTFTKFTAIGVYLEESAMSSLALK
Pav_sc0007510.1_g020.1.mk     ............................................................
Prudul26B016396               ............................................................
PRUPE_2G225200                ............................................................
gene-LOC116194939             ............................................................
```

```
Medicago                       ............................................................
Jr07_35760                     ............................................................
Kaladp0060s0328.v1.1           ............................................................
geneMaker00000028              ............................................................
gene-GIB67_014039              ............................................................
gene-GIB67_016345              ............................................................
gene-GIB67_037506              ............................................................
gene-GIB67_041777              ............................................................
gene-FCM35_KLT21231            ............................................................
Lsat_1_v5_gn_9_66221.v5        ............................................................
LPERR03G33170                  ............................................................
Liphi.11G022900.v1.1           ............................................................
Lchi23460                      ............................................................
Lchi33204                      ............................................................
Lchi33866                      ............................................................
Lchi34914                      ............................................................
gene-Leryth_002794             ............................................................
Spe07827                       ............................................................
Spe20163                       ............................................................
KYUSg_chr4.5001                ............................................................
gene-LOC124680986              ............................................................
gene-LOC124695032              ............................................................
Lj1A930T84                     ............................................................
Luann.0362s0045.v1.1           ............................................................
Luann.0427s0040.v1.1           ............................................................
TanjilG_30460                  ............................................................
TanjilG_24711                  ............................................................
gene-LOC122057215              ............................................................
gene-BVC80_1651g70             ............................................................
gene-LOC131167055              ..............F.............................................
gene-LOC131167056              ............................................................
Mamar.0037s0511.v1.1           ............................................................
Mamar.0043s0032.v1.1           ............................................................
MD01G0093100                   WKGKTAEELTESVEFFRDIVTGPFEKFIQVTTILPLTGQQYSHKVSENCVAFWKSIGIYT
MD01G0093200                   ......................................................SIGIYT
MD01G0093300                   ............................................................
MD07G0154300                   ............................................................
MD07G0154400                   ............................................................
gene-LOC123192628              ............................................................
gene-LOC123227022              ............................................................
gene-MANES_07G107200v8         ............................................................
gene-LOC11445536               ............................................................
gene-LOC11446745               ............................................................
Mde013561.1                    ............................................................
gene-MLD38_024177              ............................................................
gene-MERR_LOCUS28634           ............................................................
Migut.D00159.v2.0              ............................................................
gene-LOC111006117              ............................................................
MoBGI036118g0111               ............................................................
gene-L484_017264               ............................................................
gene-LOC103981836              ............................................................
gene-LOC104594676              ............................................................
gene-LOC104599237              ............................................................
CHI                            ............................................................
NC6G0256760                    WAPKSVDDLETSEEFFKDIIDGDFEKFTRITFIKLLRGEEFTSKVVENCVAIWKSAGIYT
gene-F0562_031805              ............................................................
ORUFI03G40690                  ............................................................
ORUFI05G23340                  ............................................................
protein_coding_16416           ............................................................
gene-PAHAL_3G142600            ............................................................
gene-PAHAL_9G032500            ............................................................
C5167_000623                   ............................................................
C5167_044917                   ............................................................
C5167_046772                   ............................................................
gene-PanWU01x14_043220         ............................................................
gene-BS78_01G031900            ............................................................
gene-BS78_01G032000            ............................................................
gene-C2S52_017353              ............................................................
gene-LOC110026679              ............................................................
Phala.01G033500.v1.1           ............................................................
gene-PHAVU_002G276500g         ......................PF.......LYLPFIN......................
gene-PHAVU_007G008500g         ............................................................
gene-LOC103696411              ............................................................
gene-LOC108511484              ............................................................
gene-PHJA_002477300            ............................................................
PIPE19196                      ............................................................
PIPE22647                      ............................................................
gene-LOC116107972              ............................................................
gene-LOC116107975              ............................................................
Psat6g237840                   ............................................................
Ptrif.0004s0460.v1.3.1         ............................................................
gene-LOC118059901              ............................................................
Potri.010G213000.v4.1          ............................................................
FUN_004111.v1.0                ............................................................
PvLHv1_097350                  WKGKSGEDLTKSVEFFRDIVSGPFEKFTQVRMILPLTGKEYSEKVAENCVAFWKSIGTYT
Pav_sc0007510.1_g020.1.mk      ............................................................
Prudul26B016396                ............................................................
PRUPE_2G225200                 ............................................................
gene-LOC116194939              .................................IPATEQ...............SIHL..
```

```
Medicago                        ............................................................
Jr07_35760                      ............................................................
Kaladp0060s0328.v1.1            ............................................................
geneMaker00000028               ............................................................
gene-GIB67_014039               ............................................................
gene-GIB67_016345               ............................................................
gene-GIB67_037506               ............................................................
gene-GIB67_041777               ............................................................
gene-FCM35_KLT21231             ............................................................
Lsat_1_v5_gn_9_66221.v5         ............................................................
LPERR03G33170                   ............................................................
Liphi.11G022900.v1.1            ............................................................
Lchi23460                       ............................................................
Lchi33204                       ............................................................
Lchi33866                       ............................................................
Lchi34914                       ............................................................
gene-Leryth_002794              ............................................................
Spe07827                        ............................................................
Spe20163                        ............................................................
KYUSg_chr4.5001                 ............................................................
gene-LOC124680986               ............................................................
gene-LOC124695032               ............................................................
Lj1A930T84                      ............................................................
Luann.0362s0045.v1.1            ............................................................
Luann.0427s0040.v1.1            ............................................................
TanjilG_30460                   ............................................................
TanjilG_24711                   ............................................................
gene-LOC122057215               ............................................................
gene-BVC80_1651g70              ............................................................
gene-LOC131167055               ......................HLSLLFLHS.............................
gene-LOC131167056               ............................................................
Mamar.0037s0511.v1.1            ............................................................
Mamar.0043s0032.v1.1            .......................NNLL.................................
MD01G0093100                    DAEGKAIEKFLEVFKDQNFPP.GASILFTQSPKGSLMISFSRDASVPEAANTVIENKLLS
MD01G0093200                    DAEGKAIEKFLEVFKDQNFPP.GASILFTQSPKGSLTISFSRDASVPEAANTVIENKLLS
MD01G0093300                    ............................................................
MD07G0154300                    ............................................................
MD07G0154400                    ............................................................
gene-LOC123192628               ............................................................
gene-LOC123227022               ............................................................
gene-MANES_07G107200v8          ............................................................
gene-LOC11445536                ............................................................
gene-LOC11446745                ............................................................
Mde013561.1                     ............................................................
gene-MLD38_024177               ............................................................
gene-MERR_LOCUS28634            ............................................................
Migut.D00159.v2.0               ............................................................
gene-LOC111006117               ............................................................
MoBGI036118g0111                ............................................................
gene-L484_017264                ............................................................
gene-LOC103981836               ............................................................
gene-LOC104594676               .......................SNLLFSLH.............................
gene-LOC104599237               ............................................................
CHI                             ............................................................
NC6G0256760                     DAEAQAAEKLKEVFKEQVFPP.GSSIAMKHSTTGSLTIAFSKDTSVPENGVAVIENKALT
gene-F0562_031805               ............................................................
ORUFI03G40690                   ............................................................
ORUFI05G23340                   ............................................................
protein_coding_16416            ............................................................
gene-PAHAL_3G142600             ............................................................
gene-PAHAL_9G032500             ............................................................
C5167_000623                    ............................................................
C5167_044917                    ............................................................
C5167_046772                    ............................................................
gene-PanWU01x14_043220          ............................................................
gene-BS78_01G031900             ............................................................
gene-BS78_01G032000             ............................................................
gene-C2S52_017353               ............................................................
gene-LOC110026679               ............................................................
Phala.01G033500.v1.1            ............................................................
gene-PHAVU_002G276500g          ...................PPL...FIFNFS.............................
gene-PHAVU_007G008500g          ............................................................
gene-LOC103696411               ............................................................
gene-LOC108511484               ............................................................
gene-PHJA_002477300             ............................................................
PIPE19196                       ............................................................
PIPE22647                       ............................................................
gene-LOC116107972               ............................................................
gene-LOC116107975               ............................................................
Psat6g237840                    ............................................................
Ptrif.0004s0460.v1.3.1          ............................................................
gene-LOC118059901               ............................................................
Potri.010G213000.v4.1           ............................................................
FUN_004111.v1.0                 ............................................................
PvLHv1_097350                   EAEKLAIQKFLKAFETKNFPP.GASILFTQSPKGSLTIGFSFDDRFPESGEAVIDNKQLA
Pav_sc0007510.1_g020.1.mk       ............................................................
Prudul26B016396                 ............................................................
PRUPE_2G225200                  ............................................................
gene-LOC116194939               ....................P.HPKRFFSQA..GT.........................
```

```
Medicago                         ............................................................
Jr07_35760                       ............................................................
Kaladp0060s0328.v1.1             ............................................................
geneMaker00000028                ............................................................
gene-GIB67_014039                ............................................................
gene-GIB67_016345                ............................................................
gene-GIB67_037506                ............................................................
gene-GIB67_041777                ............................................................
gene-FCM35_KLT21231              ............................................................
Lsat_1_v5_gn_9_66221.v5          ............................................................
LPERR03G33170                    ............................................................
Liphi.11G022900.v1.1             ............................................................
Lchi23460                        ............................................................
Lchi33204                        ............................................................
Lchi33866                        ............................................................
Lchi34914                        ............................................................
gene-Leryth_002794               ............................................................
Spe07827                         ......................................................MSCSTS
Spe20163                         ......................................................SSSTTC
KYUSg_chr4.5001                  ............................................................
gene-LOC124680986                ............................................................
gene-LOC124695032                ............................................................
Lj1A930T84                       ............................................................
Luann.0362s0045.v1.1             ............................................................
Luann.0427s0040.v1.1             ......................................................MSCSAS
TanjilG_30460                    ............................................................
TanjilG_24711                    ............................................................
gene-LOC122057215                ............................................................
gene-BVC80_1651g70               ............................................................
gene-LOC131167055                ...........................LL.....................ATVQTSAPPS
gene-LOC131167056                ............................................................
Mamar.0037s0511.v1.1             .....................................................SSSSVVS
Mamar.0043s0032.v1.1             ..................................................SSNLSWSSSS
MD01G0093100                     DAVLESIVGKHGVSPAAKQSLAGRLSELLNG.CNE............SNDAKAGNEKDCQ
MD01G0093200                     EAVLESIVGKHGVSPAAKQSLAERLSKLLNG.CNE............SKDAKAGNEKDCQ
MD01G0093300                     ............................................................
MD07G0154300                     ............................................................
MD07G0154400                     ............................................................
gene-LOC123192628                ............................................................
gene-LOC123227022                ............................................................
gene-MANES_07G107200v8           ............................................................
gene-LOC11445536                 ............................................................
gene-LOC11446745                 ............................................................
Mde013561.1                      .......................................................MSNSP
gene-MLD38_024177                ............................................................
gene-MERR_LOCUS28634             ......................................................MSSSAC
Migut.D00159.v2.0                .........................................................AEK
gene-LOC111006117                ............................................................
MoBGI036118g0111                 ............................................................
gene-L484_017264                 ............................................................
gene-LOC103981836                ............................................................
gene-LOC104594676                ..........................LFI.....................QPWQCRATMH
gene-LOC104599237                ............................................................
CHI                              ............................................................
NC6G0256760                      LSFLESVIGKHGVSPAAKRSVAERISGLLKEG...WMDCTNEGEPAPSVHGTKSPSSRLL
gene-F0562_031805                ..........................................................MY
ORUFI03G40690                    ............................................................
ORUFI05G23340                    ............................................................
protein_coding_16416             ............................................................
gene-PAHAL_3G142600              ............................................................
gene-PAHAL_9G032500              ............................................................
C5167_000623                     ............................................................
C5167_044917                     ............................................................
C5167_046772                     ............................................................
gene-PanWU01x14_043220           ............................................................
gene-BS78_01G031900              ............................................................
gene-BS78_01G032000              ............................................................
gene-C2S52_017353                ............................................................
gene-LOC110026679                ............................................................
Phala.01G033500.v1.1             ............................................................
gene-PHAVU_002G276500g           ...............................................FHFTSTHQIHSLL
gene-PHAVU_007G008500g           ............................................................
gene-LOC103696411                ............................................................
gene-LOC108511484                ............................................................
gene-PHJA_002477300              ............................................................
PIPE19196                        ............................................................
PIPE22647                        ............................................................
gene-LOC116107972                ............................................................
gene-LOC116107975                ............................................................
Psat6g237840                     ............................................................
Ptrif.0004s0460.v1.3.1           ............................................................
gene-LOC118059901                ............................................................
Potri.010G213000.v4.1            ............................................................
FUN_004111.v1.0                  ............................................................
PvLHv1_097350                    EAILESIIGLQAYKPV..........FFVNF.............................
Pav_sc0007510.1_g020.1.mk        ............................................................
Prudul26B016396                  ............................................................
PRUPE_2G225200                   ............................................................
gene-LOC116194939                .........................................................KRE
```

```
                                     1
                                     .
Medicago                         ....M....................................................AAS
Jr07_35760                       .MV.P....................................................APS
Kaladp0060s0328.v1.1             .MA.AAAAIDV...........................................ETHHPL
geneMaker00000028                .MT.T....................................................LPS
gene-GIB67_014039                .MA.QQH................FAS.ALLFFFVLLVT..............DL..EALS
gene-GIB67_016345                .MK.ERKKENLRSTESYGSTAFCICS.SILLCF..LVT..............DL..EALS
gene-GIB67_037506                .MG........................................................H
gene-GIB67_041777                .MG........................................................H
gene-FCM35_KLT21231              .MG.EVSS................................................FALP
Lsat_1_v5_gn_9_66221.v5          .MA.P....................................................PPS
LPERR03G33170                    .MA.......................................................PA
Liphi.11G022900.v1.1             .MS.P....................................................PPP
Lchi23460                        .MG.S....................................................SPI
Lchi33204                        .MG.S....................................................SPI
Lchi33866                        .MG.S....................................................SPI
Lchi34914                        .MG.S....................................................SPI
gene-Leryth_002794               .MA.M....................................................SQS
Spe07827                         .PS.P....................................................LPP
Spe20163                         .LS.P....................................................LPS
KYUSg_chr4.5001                  ..........................................................MA
gene-LOC124680986                ..........................................................MA
gene-LOC124695032                ..........................................................MA
Lj1A930T84                       .MS.P....................................................LTS
Luann.0362s0045.v1.1             .MP......................................................LPS
Luann.0427s0040.v1.1             .PS.S....................................................LPS
TanjilG_30460                    .MA......................................................APT
TanjilG_24711                    .MH.PKLHSYV..........LAIAC.NIMIF...IVSVNANHNHNLKHCSKSH..RLKT
gene-LOC122057215                .MA.P...................................................GAVM
gene-BVC80_1651g70               .MA.SVEQ.......................................QQQQQLE..QHKL
gene-LOC131167055                .SA.A....................................................KPS
gene-LOC131167056                .MA.S....................................................TPP
Mamar.0037s0511.v1.1             .PS.P....................................................LPT
Mamar.0043s0032.v1.1             PMP......................................................LPS
MD01G0093100                     .IA.P....................................................PPS
MD01G0093200                     .IA.P....................................................PPS
MD01G0093300                     .MA.P....................................................TPS
MD07G0154300                     .MA.P....................................................PPS
MD07G0154400                     .M..........................................................
gene-LOC123192628                .MN.P....................................................SAS
gene-LOC123227022                .MN.P....................................................SPV
gene-MANES_07G107200v8           .MS.P...................................................AATP
gene-LOC11445536                 .MA......................................................LPS
gene-LOC11446745                 .MA......................................................TPS
Mde013561.1                      PMP......................................................LPS
gene-MLD38_024177                .MR.................................................DYC....T
gene-MERR_LOCUS28634             .LS.P....................................................LPS
Migut.D00159.v2.0                .VS.E....................................................KLS
gene-LOC111006117                .MA.P....................................................VAD
MoBGI036118g0111                 .MA.PTPSAAEVQ.........................VEAV.............NFLPS
gene-L484_017264                 .MA......................................................LTT
gene-LOC103981836                .MS.GG..................................................TGSP
gene-LOC104594676                .MT.....................................................RPTT
gene-LOC104599237                .MS.P....................................................APF
CHI                              ............................................................
NC6G0256760                      .MA.P....................................................LPS
gene-F0562_031805                .PS.P....................................................SPS
ORUFI03G40690                    .........................................................MAA
ORUFI05G23340                    .ME.........................................................
protein_coding_16416             .MS.K....................................................SPS
gene-PAHAL_3G142600              .MA........................................................V
gene-PAHAL_9G032500              ..........................................................MA
C5167_000623                     .MA.P....................................................MAQ
C5167_044917                     .MA.P....................................................MAQ
C5167_046772                     .MKFTSLS...NSVPVF....L....ISLLIF............SLYADKCDAT..FWPF
gene-PanWU01x14_043220           .MA.P...................................................APST
gene-BS78_01G031900              ..........................................................MA
gene-BS78_01G032000              ..........................................................MA
gene-C2S52_017353                .MS.A....................................................TSS
gene-LOC110026679                .MA......................................................ETV
Phala.01G033500.v1.1             ..........................................................MA
gene-PHAVU_002G276500g           PMS......................................................LPS
gene-PHAVU_007G008500g           .MS......................................................LPS
gene-LOC103696411                .MG.EG...................................................AVA
gene-LOC108511484                ............................................................
gene-PHJA_002477300              .MS.T....................................................PPS
PIPE19196                        .MA.PA..................................................GLPA
PIPE22647                        .MA.PA..................................................GLPA
gene-LOC116107972                .MN.P....................................................SPS
gene-LOC116107975                .MN.P....................................................SPS
Psat6g237840                     .MT......................................................TPS
Ptrif.0004s0460.v1.3.1           .MN.P....................................................SPF
gene-LOC118059901                .MS.L....................................................AVP
Potri.010G213000.v4.1            .MS.P....................................................AVP
FUN_004111.v1.0                  .ME.A....................................................TPA
PvLHv1_097350                    .TS.E....................................................GIM
Pav_sc0007510.1_g020.1.mk        .MA.A....................................................LPN
Prudul26B016396                  .MA.A....................................................LPN
PRUPE_2G225200                   .MA.A....................................................LPN
gene-LOC116194939                PMKRPSPR...............................................NAMVA
```

```
                                            10          20            30
                                             .           .             .
Medicago                   ....IT........AITVENLEYPA.VV.TSPV....TGKSYFLGGA..............
Jr07_35760                 ....LP........VVHVDNVAFQP.TT.KVPG....SDKALFLGGA..............
Kaladp0060s0328.v1.1       ....AS........AIQVENLTFPA.KVEAPPG....SKNAFILGGA..............
geneMaker00000028          ....VT........DIQVEAVTFPA.AA.KPPA....STKTHFLGGA..............
gene-GIB67_014039          ....LK........GLVIEGVTFPV.TV.KPPG....SNNTLFLGGA..............
gene-GIB67_016345          ....LK........GLVIEGVTFPL.TV.KPPG....PSNTLFHGGA..............
gene-GIB67_037506          ....TS........ELEIEGVLFPS.TV.KPLG....STNTLFLGGA..............
gene-GIB67_041777          ....TS........ELEIEGVLFPS.TV.KPLG....STNTLFLGGA..............
gene-FCM35_KLT21231        ....PT........PLHIDGVTFPA.FA.TPPEYSSSSKSPLFLAGA..............
Lsat_1_v5_gn_9_66221.v5    ....PT........SLQIESVVFPP.SV.KPPG....ATNALFLAGA..............
LPERR03G33170              ....VS........ELEVDGVVFPPAVT.RPPG....SGNTHFLAGA..............
Liphi.11G022900.v1.1       ....IT........DVQVESCVFPA.AV.NPPG....SAKTFFLGGA..............
Lchi23460                  ....LT........PVQIESVVFPP.TA.KPPG....SSKTLFLGGA..............
Lchi33204                  ....LT........PVQIESVVFPP.TA.KPPG....SSKTLFLGGA..............
Lchi33866                  ....LT........PVQIESVVFPP.TA.KPPG....SSKTLFLGGA..............
Lchi34914                  ....LT........PVQIESVVFPP.TA.KPPG....SSKTLFLGGA..............
gene-Leryth_002794         ....VT........SVQVENHVFPA.AV.TPPD....SSNTLFLGGA..............
Spe07827                   ....VT........KLHVDSVTFPP.SV.IPPA....SSKTLFLGGA..............
Spe20163                   ....VT........QLQVDAVTFLP.SV.NSPA....SSNHLFLSGA..............
KYUSg_chr4.5001            ....VS........ELEVDGVVFPP.LA.RPPG....TAHAHFLAGA..............
gene-LOC124680986          ....VS........ELEVDGVVFPP.LA.RPPG....TAHAHFLAGA..............
gene-LOC124695032          ....VS........ELEVDGVVFPP.LA.RPPG....TAHAHFLAGA..............
Lj1A930T84                 ....IT........GIQVESHVFPS.SV.KPPG....TANYMFLGGA..............
Luann.0362s0045.v1.1       ....VT........PLRVDAFTFPP.AI.ESPA....SHKKLFLGGA..............
Luann.0427s0040.v1.1       ....VT........KLQVDSVTFPP.SV.ISPA....SSNPLFLGGA..............
TanjilG_30460              ....VT........SVEIENVVFPS.TV.KPPG....SANAFFLGGA..............
TanjilG_24711              ....VK........PVKVENVTFPP.FV.NATG....SNNNFFLGGA..............
gene-LOC122057215          ....VP........KLQVEEVTFPG.SV.KPPG....STNILFLGGA..............
gene-BVC80_1651g70         ....VS........ELKVEEFVFPP.TV.KPPG....STNTLFLGGA..............
gene-LOC131167055          ....VA........GLSVGGYFFSP.TV.KPPG....STNTLFLGGA..............
gene-LOC131167056          ....VS........GVQVESLLFPP.TA.KPPG....SANTLFLGGA..............
Mamar.0037s0511.v1.1       ....VA........KLEVDSVTFAP.SI.KSPA....SSNQLFLGGA..............
Mamar.0043s0032.v1.1       ....VT........PLHVDAFTFPP.AV.ESPA....SHKKLFLGGA..............
MD01G0093100               ....LA........GLQVETTAFPP.SV.KPPG....SPNTLFLGGA..............
MD01G0093200               ....LA........GLQVETTAFPP.SV.KPPG....SSNTLFLGGA..............
MD01G0093300               ....LA........GLQVETTAFPP.SA.KPPG....SSNTLFLGGA..............
MD07G0154300               ....LA........GLQVGATAFPP.SV.KPPG....SSNTLFLGGA..............
MD07G0154400               .............................................................
gene-LOC123192628          ....VS........DGQVEHFTFSS.TI.QPPA....SSKTHFLGGA..............
gene-LOC123227022          SSHFVS........DVQIERFTFSS.TV.KPPG....SSKIHFLGGA..............
gene-MANES_07G107200v8     ....VT........HIEVETIAFSP.AV.KPPA....SDKTLFLSGA..............
gene-LOC11445536           ....VT........ALEIENYAFPP.TV.KPPG....STNNFFLGGA..............
gene-LOC11446745           ....VT........SLAIESIVFPP.TM.KAPG....STNNFFLGGA..............
Mde013561.1                ....VT........PLHVDVYTFPP.AI.ESHA....SHKKLFLGGA..............
gene-MLD38_024177          ....IAPGRRNPG.....RDRLLPP.IR.EAPG....SSNTLFLGGA..............
gene-MERR_LOCUS28634       ....VT........KLQVDSVTFSP.SV.NSPA....SSKPLFLGGA..............
Migut.D00159.v2.0          ....VT........QVQVESCVFPP.AI.NPPG....SAKTFFLGGA..............
gene-LOC111006117          ....LG........GVQVENVPFPP.QI.KPPA....SAKTLFLGGA..............
MoBGI036118g0111           ....VT........EVQVEAVTFPP.SV.KPPG....STKTLFLGGA..............
gene-L484_017264           ....VA........GVQVEIATFPP.AA.TPPG....SDKTLFLGGA..............
gene-LOC103981836          ....LP........MLEVEGVVFPP.VV.TPPG....STKAHFLGGA..............
gene-LOC104594676          ....LT........GLSVQGFVYPP.FV.KPPG....SSNTLFLGGA..............
gene-LOC104599237          ....VS........GLQVESFSFPP.TV.KPPG....STKTLFLGGA..............
CHI                        ....ME........SITIENYVFPSTMV.KPPG....STNSFFLAGA..............
NC6G0256760                ....VS........DLTVEEYVFPA.TV.TPPE....SEKTLFLAGAGPSSDRCSSLPLYI
gene-F0562_031805          ....VT........ELQVDSYVFPP.TV.KPPG....TTKTLFLGGA..............
ORUFI03G40690              ....VS........EVEVDGVVFPP.VA.RPPA....SARTHFLAGA..............
ORUFI05G23340              .........................................KHLAL..A.............Y.
protein_coding_16416       ....VT........GVQVETQLFPP.TV.KPPG....TTNTLFLAGA..............
gene-PAHAL_3G142600        ....SS........EVAVEGVVFPP.VA.CPPG....SSRKHFLAGG..............
gene-PAHAL_9G032500        ....VS........EVAVDGVVFPP.VA.HPPG....SGRSHFLAGA..............
C5167_000623               ....LS........EIQVEQFVFPP.TM.TPPS....STESLFLGGA..............
C5167_044917               ....LS........EIQVEQFVFPP.TM.TPPS....STESLFLGGA..............
C5167_046772               ....LK........AIEIEGCKFRP.FV.TPPG....STQILFLAGS..............
gene-PanWU01x14_043220     ....LT........GVQIETATFPP.AV.TPPG....SAKALSLGGA..............
gene-BS78_01G031900        ....VS........EVVVDGVAFPP.VA.RPPG....SGRSHFLAGA..............
gene-BS78_01G032000        ....VS........EVVVDGVVFPP.VV.HPPV....SGRSHFLAGA..............
gene-C2S52_017353          ....VT........QVQVGSVVFPP.SV.KPPG....SNNTLFLGGA..............
gene-LOC110026679          ....AT........PIEVEGVKFPA.EI.SSPA....TSKPLFLGGA..............
Phala.01G033500.v1.1       ....VS........ALEVEGIAFPP.TS.HPPG....CDATHFLAGA..............
gene-PHAVU_002G276500g     ....VT........ALDVDNVTFPP.TV.NPPS....SATAFFLAGA..............
gene-PHAVU_007G008500g     ....VT........AVDVDTVTFPP.AV.NPPS....SSVAFFLAGA..............
gene-LOC103696411          ....LP........KLEVEGFVFPP.VV.RPPG....SSKTLFLGGA..............
gene-LOC108511484          .............................................................
gene-PHJA_002477300        ....VT........EVQVESCVFPP.AA.KPPG....SAKTLFLGGA..............
PIPE19196                  ....VS........ELQVEGTLFPA.AV.KPPG....SSTNLFLAGA..............
PIPE22647                  ....VS........ELQVEGTLFPA.AV.KPPG....SSTNLFLAGA..............
gene-LOC116107972          ....VS........DGQVEHFTFSS.TV.KPPG....SSKTHFLGGS..............
gene-LOC116107975          ....VS........DGQVEHVTFSS.TV.KPPG....SSKTHFLGGS..............
Psat6g237840               ....VT........ALQIENYQFPP.TV.KPPG....SDNNFFLAGA..............
Ptrif.0004s0460.v1.3.1     ....VT........ELQVENVTFTP.SV.QPPG....STKSHFLGGA..............
gene-LOC118059901          ....LS........EIQVENVTFPP.AV.KPPA....SNNTLFLGGA..............
Potri.010G213000.v4.1      ....LS........EIQIENVTFPP.AV.KPPA....SNNTLFLGGA..............
FUN_004111.v1.0            ....VS........EVKVEDYVFPP.TA.KAPG....SDKLFFLAGA..............
PvLHv1_097350              ....SS........PIQIEEHVFPA.TV.RAPG....SAASFFLGGA..............
Pav_sc0007510.1_g020.1.mk  ....PT........GLQIEATSFPP.SV.KPPG....SANTLFLGGA..............
Prudul26B016396            ....LT........GLQIEATSFPP.SV.KPPG....SANTLYLGGA..............
PRUPE_2G225200             ....LT........GLQIEATSFPP.SV.KPPG....SANTLFLGGA..............
gene-LOC116194939          ....AT........EVQVESFTFPA.AV.KPQG....SAKTLFLGGA..............
```

```
                                                                   40        50         60
Medicago                     ..........................GER.GLTIEGNFIKFTAI.GVYL.E.D..IA.VA
Jr07_35760                   ..........................GER.GLEIQGNFVKFTAI.GVYL.E.D..TA.VP
Kaladp0060s0328.v1.1         ..........................GVR.GLEIQGRFIKFTAI.GVYL.E.D.GEA.LK
geneMaker00000028            ..........................GAR.GLEIEGKFIKFTAI.GVYL.D.D..SA.VP
gene-GIB67_014039            ..........................GYR.GLNIQNTFTKVTSI.GVYL.Q.G..TA.VS
gene-GIB67_016345            ..........................GYR.GLNIQNIFTKVTSI.GVYL.Q.G..TA.VS
gene-GIB67_037506            ..........................GAR.GLQVEDKFIKFTAI.GVYL.E.D..KG.VE
gene-GIB67_041777            ..........................GAR.GLQVEDKFIKFTAI.GVYL.E.D..KG.VE
gene-FCM35_KLT21231          ..........................GVR.GLEIGGKFVKFTAI.GIYL.E.E..SS.LG
Lsat_1_v5_gn_9_66221.v5      ..........................GVR.GMEIDGNFVKFTGI.GVYL.E.D..KA.IP
LPERR03G33170                ..........................GVR.GMEIAGNFIKFTAI.GVYL.E.E.DAA.VP
Liphi.11G022900.v1.1         ..........................GVR.GMEIQGKFVKFTAI.GVYL.E.D..NA.VP
Lchi23460                    ..........................GCR.GAEIQGKFIKFTAI.GVYL.E.D..DA.VP
Lchi33204                    ..........................GCR.GAEIQGKFIKFTAI.GVYL.E.D..DA.VP
Lchi33866                    ..........................GCR.GAEIQGKFIKFTAI.GVYL.E.D..DA.VP
Lchi34914                    ..........................GCR.GAEIQGKFIKFTAI.GVYL.E.D..DA.VP
gene-Leryth_002794           ..........................GVR.GMEIQGKFIAFTVI.AVYL.E.S..IA.IP
Spe07827                     ..........................GVR.GLDIQGKFVIFTVI.GVYL.D.P..NA.IS
Spe20163                     ..........................GVR.GLDIHGKFVIFTVI.GIYL.D.A..VA.VP
KYUSg_chr4.5001              ..........................GVR.GMELGGNFIKFTAI.GVYL.Q.A.DAA.VS
gene-LOC124680986            ..........................GVR.GMELGGNFIKFTAI.GVYL.Q.A.DAA.VF
gene-LOC124695032            ..........................GVR.GMELGGNFIKFTAI.GVYL.Q.A.DAA.VS
Lj1A930T84                   ..........................GVR.GLEIQGKFIKFTAI.GVYL.E.E..KA.VA
Luann.0362s0045.v1.1         ...................................GRFVIVTVI.GVYL.E.A..LA.VP
Luann.0427s0040.v1.1         ..........................GVR.GLDIQGKFVIFTVI.GIYL.D.S..NA.VP
TanjilG_30460                ..........................GVR.GLQIQDNFVKFTAI.GIYL.Q.H..HA.VS
TanjilG_24711                ..........................GVR.GLQEQGKFIKFTDI.GIYL.Q.D..NA.VS
gene-LOC122057215            ..........................GVR.GLEIQGQFIKFTTI.GIYL.G.Q..EA.LP
gene-BVC80_1651g70           ..........................GVR.GLQIQDKFIKFTAI.GVYL.G.E..ES.IP
gene-LOC131167055            ..........................GAR.GLEIQGKFVNVTAI.GVYL.E.D..KA.VP
gene-LOC131167056            ..........................GAR.GLEIQGKFVKFTAI.GVYL.E.D..SA.VP
Mamar.0037s0511.v1.1         ..........................GVR.GLDIHGKFIIFTVI.GVYL.D.A..NA.VP
Mamar.0043s0032.v1.1         ..........................GIR.WFDIEGKFVIVTVI.GVYL.E.A..MA.VP
MD01G0093100                 ..........................GVR.GLEIQGNFVKFTAI.GVYL.E.D..NA.VP
MD01G0093200                 ..........................GVR.GLEIQGNFVKFTAI.GVYL.E.D..NA.VP
MD01G0093300                 ..........................GVR.GLEIQGNFVKFTAI.GVYL.E.E..NA.VP
MD07G0154300                 ..........................GMR.GLEIQGNFVKVTAI.GVYL.E.D..NA.VP
MD07G0154400                 ..........................G.........NFI......LFYFCQMRDLKVFVS
gene-LOC123192628            ..........................GVR.GLEIQGKFVKFTAI.GVYV.E.Y..SA.VT
gene-LOC123227022            ..........................GER.GLEIQGKFVKFTAI.GVYV.E.D..SA.VS
gene-MANES_07G107200v8       ..........................GAR.GLEIQGKFVKFTAI.GVYL.E.D..EA.VP
gene-LOC11445536             ..........................GER.GIQIQDKFVKFTAI.GVYL.Q.D..IA.VP
gene-LOC11446745             ..........................GVR.GIQIQDKFVKFTAI.GVYL.Q.D..IA.IP
Mde013561.1                  ..........................GIR.WFDIEGKLVIVTVI.GVYL.E.A..LA.VP
gene-MLD38_024177            ..........................GFR.GIEIPGKFLKVTAI.GLYI.E.D..GA.VP
gene-MERR_LOCUS28634         ..........................GVR.GLDIQGKFVIFTVI.GVYL.D.A..DA.VP
Migut.D00159.v2.0            ..........................GVR.GMEIEGRFIKFTAI.GVYL.E.D..NA.VQ
gene-LOC111006117            ..........................GVR.ALEIGGNSVKFTAI.GVYL.E.E..DA.VA
MoBGI036118g0111             ..........................GER.GLEIQGQFVKFTAI.GVYL.E.D..TA.LP
gene-L484_017264             ..........................GAR.GLEIQGKFVKFTTI.GVYL.E.D..NA.VK
gene-LOC103981836            ..........................GVR.GLEIEGRFVAFTAI.GVYL.E.D..AA.VQ
gene-LOC104594676            ..........................GVR.GMIINGTFVKFTVT.GIYL.G.K..EA.VP
gene-LOC104599237            ..........................GAR.GLEIQGQFIKFTAI.GIYL.E.D..IA.IP
CHI                          ..........................GNR.GLEIEGKFVKFTAI.GVYM.E.E..TA.LP
NC6G0256760                  FFSSRKSYCFGLQHALWWVGGDVGFAGVR.GLNIQGTFVKFTAI.GVYV.E.A..AA.VD
gene-F0562_031805            ..........................GVR.GLEIEGKFIKFTAI.AVYL.E.D..NA.IP
ORUFI03G40690                ..........................GVR.GVEIAGNFIKFTAI.GVYL.E.E.GAA.VP
ORUFI05G23340                ...........................IRIKL............................
protein_coding_16416         ..........................GAR.GLDIQGKFVKFTAI.GVYL.E.D..SA.VG
gene-PAHAL_3G142600          ..........................GVR.RMEAEGNFVKIAAI.GVYL.E.D..AA.VA
gene-PAHAL_9G032500          ..........................GVR.GMEIGGNFIKFTAI.GVYL.E.E.GAA.VS
C5167_000623                 ..........................GVR.GLQIQDRFIKFTAI.GVYL.A.E..EA.IP
C5167_044917                 ..........................GVR.GLQIQGRFIKFTAI.GVYL.A.E..EA.IP
C5167_046772                 ..........................GVK.EEFGDSKSMKYSSC.AIYL.Q.P..TC.IL
gene-PanWU01x14_043220       ..........................GAR.GIEIQGNFVKFTAI.GVYL.E.D..NA.VT
gene-BS78_01G031900          ..........................GVR.GLEIGGNFIKFTAI.GVYL.E.D..AA.VP
gene-BS78_01G032000          ..........................GVR.GLEIDGSFVKFTAI.GVYL.E.D..AA.VS
gene-C2S52_017353            ..........................GVR.GMEIQGNFVKFTAI.AVYL.E.D..TA.IP
gene-LOC110026679            ..........................GAR.GIEVGGKFLAVTAI.GVYL.E.A..AV.IP
Phala.01G033500.v1.1         ..........................GVR.GLEIEGNFVKFTAI.GVYL.E.E..TA.LP
gene-PHAVU_002G276500g       ..........................GVR.GLQIQDNFVKFTAI.GVYL.Q.P..NA.VP
gene-PHAVU_007G008500g       ..........................GVR.GLQIQDKFVKFTAI.GIYL.Q.P..DA.VP
gene-LOC103696411            ..........................GVR.GLEIGGRFITFTAI.GVYL.E.D..EA.IR
gene-LOC108511484            ...........................MRQSL.............DGFYR..........
gene-PHJA_002477300          ..........................GVR.GLEIGGNFVKFTAI.GVYL.E.D..NA.VS
PIPE19196                    ..........................G..............FTAI.GVYL.G.A..AA.AT
PIPE22647                    ..........................G..............FTAI.GVYL.G.A..AA.AT
gene-LOC116107972            ..........................GVR.GLEIQGKFVKFTAI.GVYV.E.N..SA.VS
gene-LOC116107975            ..........................GVR.GLEIQGKFVKFTAI.GVYV.E.D..SA.VS
Psat6g237840                 ..........................GDR.GLQIQDKFVKFTAI.AVYL.Q.D..IA.VP
Ptrif.0004s0460.v1.3.1       ..........................GVR.GLEIEGKFVKFTAI.GVYL.E.E..NA.VP
gene-LOC118059901            ..........................GVR.GLEIEGKFIKFTAI.GVYL.E.D..KS.LQ
Potri.010G213000.v4.1        ..........................GVR.GLEIEGKFIKFTAI.GVYL.E.D..NS.LQ
FUN_004111.v1.0              ..........................GER.GIEIEGRFIKVTAI.GVYL.E.A..SA.IP
PvLHv1_097350                ..........................GVR.GLDIQGKFIKFTAI.GVYL.E.E..SA.IS
Pav_sc0007510.1_g020.1.mk    ..........................GVR.GLEIQGNFVKFTAI.GVYL.E.E..KA.VP
Prudul26B016396              ..........................GFR.GLEIQGNFVKFTAI.GVYL.E.D..KA.VP
PRUPE_2G225200               ..........................GVR.GLEIQGNFVKFTAI.GVYL.E.D..KA.VP
gene-LOC116194939            ..........................GAR.GLEIEGKFIKFTAI.GVYL.E.E..AA.LP
```

```
                                       70        80        90         100       110
Medicago                     SLAA.KWKGKSSEE.LLETLDFYRDIIS.GPFEKLIRGSKI..RELSGPEYSRKVMENCV
Jr07_35760                   SLAI.KWSGKSADE.LSESVEFFRDIVT.GPFEKFIRVTMI..LPLTGHQYSEKVSENCV
Kaladp0060s0328.v1.1         ALGG.KWSRKTAEE.LLDSSDFYLDIVT.GPFEKFTQVTTI..LPLTGQQYSEKVAENCV
geneMaker00000028            LLAV.KWKGKSAHE.LTDSVEFFRDIVT.GPFEKFIRVTMI..LPLTGQQYSEKVAENCI
gene-GIB67_014039            CLAP.KWAGKTKKE.LANSVKFFQDIFT.GPFEKFTRVTMI..MPLTGPQYSEKVAENCV
gene-GIB67_016345            CLTP.KWAGKTKKE.LANFVKFFQDIFT.SSFEKFTRVTMI..MPLTGPQYSKRVAENYV
gene-GIB67_037506            VLGA.KWKGKTADE.LTKSVEFVKDVVM.GPFEKFIRVTMI..LPLTGQMYAEKVTENCV
gene-GIB67_041777            VLGA.KWKGKTADE.LTESVEFVKDVVM.GPFEKFIRVTMI..LPLTGQMYAEKVTENCV
gene-FCM35_KLT21231          ALAE.KWTAKPADE.LAASPDFYADIIN.GPFEKFVRVTMI..LPLTGEMYSDKVSENCM
Lsat_1_v5_gn_9_66221.v5      SLAV.KWKGKTAAE.LTDSVEFFRDIVT.GPFEKLTQVTMI..LPLTGKQYSEKVSEMCV
LPERR03G33170                SLAK.TWAGKSADELLAGGGEFFRDVVT.GEFEKFTRVTMI..LPLTGEQYSDKVTENCV
Liphi.11G022900.v1.1         SLAV.KWKGKSAEE.LTDSVDFFSEIVT.GPFEKFTRVTTI..LPLTGQQYSEKVAENCV
Lchi23460                    LLAV.KWKGKTAQE.LTDSVEFFRDIVT.GPFEKFTRVTMI..LPLTGQQYSEKVTENCV
Lchi33204                    LLAV.KWKGKTAQE.LTDSVEFFRDIVT.GPFEKFTRVTMI..LPLTGQQYSEKVTENCV
Lchi33866                    LLAV.KWKGKTAQE.LTDSVEFFRDIVT.GPFEKFTRVTMI..LPLTGQQYSEKVTENCV
Lchi34914                    LLAV.KWKGKTAQE.LTDSVEFFRDIVT.GPFEKFTRVTMI..LPLTGQQYSEKVTENCV
gene-Leryth_002794           SLAV.KWKGKTPHE.LTDSVDFFKDIFG.GPFEKFTQVTMV..LPLSGKQYSEKVAENCV
Spe07827                     SLSV.KWEGKTKEE.LTESVPFFREIVT.GPFEKFIKVTMK..LPLTGQQYSEKVTENCV
Spe20163                     SLSV.KWKGKTTKE.LTESVPFFREIVT.GEFEKFIKVTMK..LPLTGPQYSEKVTENCV
KYUSg_chr4.5001              ALAA.KWAGKPADE.LAADNAFFRDVVT.GEFEKFTRVTMI..LPLTGAQYSEKVTENCV
gene-LOC124680986            ALAA.KWAGKPADE.LAADNAFFRDVVT.GEFEKFTRVTMI..LPLTGAQYSEKVTENCV
gene-LOC124695032            ALAA.KWAGKPADE.LAADNAFFRDVVT.GEFEKFTRVTMI..LPLTGAQYSEKVTENCV
Lj1A930T84                   SLAD.KWKGKTAEE.LADSVEFFADIVT.GPFEKFTQVTMI..LPLTGQQYSEKVAENCV
Luann.0362s0045.v1.1         SLSV.KWKGKNANE.LTESIPFFRQLVT.GAFEKFVRVTMK..VKLRGTQYSDKVAEYCE
Luann.0427s0040.v1.1         SLSV.KWKGKTTEE.LTESVPFFREIVT.GGFEKFIKVTMK..LPLTGKQYSEKVTENCV
TanjilG_30460                SLAV.KWNGKNAHE.LTESVEFFRDIVT.GPFDKFMQVTML..LPLTGQQYSEKVSENCV
TanjilG_24711                SLAD.KWHGKSTKK.LNKSNEFFKDIIK.GPFEKFMQVTLI..LPLSGPQYSEKVAENCA
gene-LOC122057215            SLAS.KWKGKTIDE.LTSSLDFFSDIVS.GEFEKFIKVTML..KPLTGQMYAEKVTENCV
gene-BVC80_1651g70           SLASNKWNSKTADE.LKDDLDFFMDIVT.GPFEKFTRITMI..LPLTGNQYAEKVMENCV
gene-LOC131167055            SLAV.KWKGKSAAE.LTESVDFFRDIVT.GPYEKFTRVTTI..LGLTGRQYSEKVAENCV
gene-LOC131167056            SLAV.KWKGKSAEE.LTESVDFFRDIVT.GPFEKFMQVTMI..LPLTGQQYSEKVTENCV
Mamar.0037s0511.v1.1         SLSV.KWKGKTTEE.LTESVPFFREIVT.GAFEKFIKVTMK..LPLTGQQYSEKVTENCV
Mamar.0043s0032.v1.1         SLSV.KWKGKNAKE.LTESVPFFRQLVT.GVFEKFVRVTMK..VKLTGVQYSEKVVEYCE
MD01G0093100                 QLAV.KWKGKTAKE.LTESVEFFRDIVT.GPFEKFIQVTTI..LPLTGQQYSEKVSENCI
MD01G0093200                 QLAV.KWKGKTAEE.LMESVEFFRDIVT.GPFEKFIQVTTI..LPLTGQQYSDKVSENCV
MD01G0093300                 LLAV.KWKGKTAEE.LTESVEFFRDIVT.GPFEKFIQVTMI..LPLTGQQYSDKVSENCV
MD07G0154300                 LLAV.KWKGKTAEE.LSESVEFFRDIVT.GPFEKFIQVTMI..LPLTGQQYSEKVSENCV
MD07G0154400                 TLDP.KLG..................LS.RPFEKFTQVTFI..QHLTGRQYSEKVAENCI
gene-LOC123192628            SLAG.KWKGKSAEE.LSESVAFFRDIIT.GPFEKFVRVTMI..LPLTGQQYSEKVAENCV
gene-LOC123227022            SLAG.KWKGKSAEE.LSESVAFYRDIVT.GPFEKFIRVTMI..LPLTGPQYSGKVAENCV
gene-MANES_07G107200v8       LLAV.KWKGKSALE.LTDSVEFFRDIVT.GPFEKFIRVSTI..LPLTGPQYSEKVSENCV
gene-LOC11445536             YLAE.KWKARSAHE.LTDTVPFFRDIVT.GPFEKFMRVTMI..LPLTGHQYSEKVSENCV
gene-LOC11446745             YLAA.KWKGKPPHK.LTESVPFFMDIVT.GPFEKFMRVTMI..RPLTGQEYSNKVSENCV
Mde013561.1                  SLSV.KWKGKNAKE.LTESIPFFRQLVT.GEFEKFVRVTMK..VKITGKQYSEKVAEYCE
gene-MLD38_024177            FLAA.KWKGKAAEE.LAESPEFIHDVVA.APYERFLRVTML..LPLTGVQYSEKVSENCV
gene-MERR_LOCUS28634         SLSV.KWTGKTSEE.LTESVPFFREIVT.GAFEKFIKVTMK..LPLTGQQYSEKVTENCV
Migut.D00159.v2.0            SLAV.KWNGKTADE.LTDSLDFFGDIVT.GPFEKFTKVTTI..LPLTGKQYSEKVAENCV
gene-LOC111006117            PLAG.KWSGKSAEE.LMDSVEFFRDVVT.GPFEKFTNVTLI..LPLTGEQYAEKVAENCA
MoBGI036118g0111             WLNL.KWKCKSSED.LTDSVPFFRDIVT.GPFEKFTKVTMI..LPLTGQQYSEKVAENCV
gene-L484_017264             WLAG.KWKGKSVEE.LTESVEFFRDIVT.GPFEKFTRVTMI..LPLTGPQYSEKVSENCV
gene-LOC103981836            SLAS.KWKGKSADE.LDGAVEFFRDIFG.GPFEKFTRVTLL..KPLTGQQYAEKVSENCT
gene-LOC104594676            FLSV.KWKGKPANK.LLSSVKFFQDIAT.GPFDKFTRVVLI..VTLSGQEFVRKVAENCV
gene-LOC104599237            SLAV.KWKGKTASE.LTDSIDFFRDVVS.GPFDKFIRVTMI..KPLTGHQYSEKVTENCV
CHI                          FLAT.KWKSKSSEE.LANSLDFFRDIVT.GPFEKFTRVTMI..LPLTGKQYSEKVAENCV
NC6G0256760                  ALRP.KWASKSADD.LETSEEFFKDIIN.GAFEKFTRITLI..KPLRGEEYTSKVVENCV
gene-F0562_031805            SLAV.KWKGKSKEE.LMDSVEFFRDIVS.GPYEKFTKVTMI..LPLTGKQYSEKVAENCV
ORUFI03G40690                ALAK.KWAGKSADE.LAADAAFFRDVVT.GDFEKFTQVTMI..LPLTGEQYSDKVTENCV
ORUFI05G23340                .......................RDSVV.NP.............................
protein_coding_16416         SLAV.KWKGKTAEE.LTESVEFFRDVVT.GSFEKFTQVTMI..LPLTGKQYSEKVAENCV
gene-PAHAL_3G142600          ALAG.KWAGKTADE.LAADPAFFRDVYT.GEFEKFTRVTFIWPKTVAAEEFAGKVMESRV
gene-PAHAL_9G032500          ALAK.KWARKSADE.LASDVAFFRDVVT.GDFEKFTRVTMI..LPLTGEQYSDKVTENCV
C5167_000623                 SLSP.KWKSKSPEE.LNDDVEFFMDIVT.GPFEKFVKITMI..LPLTGDQYAEKVTENCV
C5167_044917                 SLSP.KWKSKSPEE.LTDDVEFFMDIVT.GPFEKFVKITMI..LPLTGDQYAEKVTENCI
C5167_046772                 YLAK.AWAQKSVVD.ITQSLNFFMDIAT.GPFEKYCRITML..ETAKGEDYAAMITKNCE
gene-PanWU01x14_043220       WLAG.KWSNKTAEE.LTESVEFFRDIVT.GPFEKFTRITMI..LPLTGQQYSEKVSENCV
gene-BS78_01G031900          ALAK.KWAGKTADE.LASDVAFFRDVVT.GDFEKFTQVTMI..LPLTGEQYSDKVTENCV
gene-BS78_01G032000          VLAN.KWRGKTADE.LASDVAFFRDVVT.GDFEKFTRVTLI..RLLTGEQYSDKVTENCV
gene-C2S52_017353            ALAL.KWKGKTADQ.LTDSDDFIAQIIT.GPFEKLTKVTMI..LPLTGQQFAEKVAENCT
gene-LOC110026679            AIAG.KWTGKKAEK.LTDSVDFYRDIIT.GSFEKLTRVTML..LPLTGQQYSEKVSGNCV
Phala.01G033500.v1.1         ELAG.KWAGKTSDE.LAAEAAFFRDVVT.GEFGKFTRITMI..LPLTGEQYSDKVAENCV
gene-PHAVU_002G276500g       LLSV.KWNGKSAPE.LTDSVEFFRDIIT.GPFEKFMQVTMI..LPLTGQQYSEKVSENCV
gene-PHAVU_007G008500g       LLSV.KWNAKSAPE.LTDSVEFFRDIVT.GPFEKFMQVTMI..LPLTGQQYSEKVSENCV
gene-LOC103696411            SLSG.KWRGKTADE.LAASVDFFRDIVA.GSFEKFTRVTMV..LPLTGQQYSEKVSENCV
gene-LOC108511484            .....FWGG...................VGDYH.....SLL..KDIR.....AMVNDGMV
gene-PHJA_002477300          SLAV.KWKGKSAEE.LTDSVDFFADIVT.GPFEKFTKVTTI..LPLTGQQYSEKVAENCV
PIPE19196                    SLAP.KWKGKTPEE.LACSEEFFLDVVT.GPYEKFTNVTLI..ATLTGEQYSDKVAENCV
PIPE22647                    SLAP.KWKGKTPEE.LACSEEFFLDVVT.GPYEKFTNVTLI..ATLTGEQYSDKVAENCV
gene-LOC116107972            SLAG.KWKGKSADE.LFESVPFFRDVVT.GPFEKFIRVTMI..LPLTGQQYSGKVAENCV
gene-LOC116107975            SLAS.KWKGKSADE.LFESVPFFRDVVT.GPFEKFIRVTMI..LPLTGQQYSEKVAENCV
Psat6g237840                 FLAE.KWKGKDVHE.LTETVPFFRDIVT.GPFEKFMQVTMI..LPLTGQQYSEKVSENCV
Ptrif.0004s0460.v1.3.1       LLAG.KWKGKTAEE.LTESVEFFRDVVT.GPFEKFMKVTMI..LPLTGAQYSEKVAENCI
gene-LOC118059901            SLSA.KWKGKTAKE.LTDSVEFFRDIVT.GPFEKFMRVTMI..LPLTGLQYSEKVAENCV
Potri.010G213000.v4.1        SLAA.KWKGKIAKE.LTDSVEFFRDIVR.GPFEKFMRVTMI..LPLTGLQYSEKVAENCV
FUN_004111.v1.0              ALAG.NWKGKTAAE.LADSVEFYRDIVT.GPFEKFTQVTMI..LPLTGPQYSEKVTENCV
PvLHv1_097350                ALAL.KWKGKSKEE.LTESVEFFRDIVA.GPFEKFTQVRTI..LPLTGKQYSEKVSENCV
Pav_sc0007510.1_g020.1.mk    LLAV.KWKGKTAQE.LTESVEFFREIIS................................
Prudul26B016396              LLAV.KWKGKTAQE.LTESVEFFREIVT.GPFEKFTQVTTI..LPLTGQQYSEKVSENCV
PRUPE_2G225200               LLAV.KWKGKTAQE.LTESVEFLREIVT.GPFEKFTQVTTI..LPLTGQQYSEKVSENCV
gene-LOC116194939            CLAT.KWKGKTAEE.LTDSVEFFRDIVT.GPYDKFMQVTMI..LPLTGAQYSEKVTENCV
```

```
                              120        130        140      150         160
Medicago                   AHLKSV..GTYGDAEAEAMQKFAEAFKPVNFPPGASVFYRQSPD..GIL....GLSFS..
Jr07_35760                 AYWKSV..GIYTDAEAKAIEEFLKVFKDEKFPPGSSILFTQSPN..GSL....TISFS..
Kaladp0060s0328.v1.1       AYWKSI..GSYMDAEEKAVDKFLQVFKDENFPPGSSILFTQSPH..GSL....TIGFS..
geneMaker00000028          AFWKSL..GLYTDAEAKAIEKFLEVFKDEKFPPGSSILITQLPH..GSL....AISFS..
gene-GIB67_014039          AYMKSI..GVYTQVEEQAVAMFLDAFKNKNFISGASVLFTQSPS..GTL....EIGFS..
gene-GIB67_016345          VYMKSI..GVYTQVEEKAVAMCLDAFKNKIFISGASVLFTQSPS..RTL....EIGFS..
gene-GIB67_037506          AIWKAM..GIYTDAEAKAVEKFIEVFKDETFPPGSSILFTQSPL..GTL....TIGFS..
gene-GIB67_041777          AIWKAM..GIYTDAEAKAVEKFIEVFKDETFPPGSSILFTQSPL..GTL....TIGFS..
gene-FCM35_KLT21231        AHWKAI..GILTEAEVDAVNKFKEVFKPETFPPGSSILFTHSTS..GAL....SIAFS..
Lsat_1_v5_gn_9_66221.v5    GVWKSN..GTYTDADAITIDKFLEVFKDQNFPPDSSILFTTSSI..GSL....SISFS..
LPERR03G33170              KAWKAA..GVYTDAEGAAAEKFKAAFKPHQFPPGSSILFTHSPS..GVL....TVAFS..
Liphi.11G022900.v1.1       AYWKAV..GKYTDAESEAIKKFLEVFKNENFPPGASILFTQSPV..GSL....TITFS..
Lchi23460                  AIWKAF..GSFTDAEATAVEKFKEAFHDQTFPPGASILFRHSSS..GSL....TIGFS..
Lchi33204                  AIWKAI..GSFTDAEATAIEKFKEAFHDQTFPPGASILFRHSSS..RSL....TIGFS..
Lchi33866                  AIWKAI..GSFTDAEATAIEKFKEAFHDQTFPPGASILFRHSSS..RSL....TIGFS..
Lchi34914                  AIWKAI..GSFTDAEATAVEKFKEAFHDQTFPPGASILFRHSSS..GSL....TIGFS..
gene-Leryth_002794         TYLKDI..GKYTEAESKAIDEFLGVFKNQNFTPGASVLFTHSSM..GSL....HIGFS..
Spe07827                   AIWKSL..GIYTESEAKAVVSFLNIFKDQTFPPGSSILFALSPN..GAL....TIAFS..
Spe20163                   AIWKSL..GVYTECEAKAVKRFLEVFKDQNFPPGSSILFALSPK..GSL....TIAFS..
KYUSg_chr4.5001            AYWKAV..GKYTDAEAAAVDKFKEAFKAESFPPGASILFTHSPA..GVL....TVAFS..
gene-LOC124680986          AYWKAV..GKYTDAEAAAVDKFKEAFKAESFPPGASILFTHSPA..GVL....TVAFS..
gene-LOC124695032          AYWKAV..GKYTDAEAAAVDKFKEAFRTESFPPGASILFTHSPA..GVL....TVAFS..
Lj1A930T84                 AHWKAL..GIYTEAEEKAIEKFVEVFKDETFPPGASILFTQSPL..GSL....TISFS..
Luann.0362s0045.v1.1       HFLKSS..GKYTISEAKAIDQFLAIFKDQDFPPGSSILFALCPK..GSL....TIAFS..
Luann.0427s0040.v1.1       AIWKSL..GIYTDREAKAVERFLEIFKDQTFPPGSSIIFALSPN..GSL....TIAFS..
TanjilG_30460              AIWKSL..GIYTDEEEKAIEKFVSVFKDETFPPGSSILFTVLPQ..GSL....VISFS..
TanjilG_24711              AILKSH..GVYTNEEEKATEKFLSVFKKETFAPGSSIFFTVLHQ..GSL....VISFS..
gene-LOC122057215          AYWKAV..GIYSDAEAKAVEEFTDTFKDENFPPLTSILFTLSPH..GSL....TIAFSKA
gene-BVC80_1651g70         AYLKAK..DTYTEAEAKAVEKFIEIFKDQTFPPASSILFTLSTL..GNL....TIGFS..
gene-LOC131167055          ALWKSA..GAYTVAEAKALAAFMEVFKDLNFPPASSILFTHSPL..GSL....TISFS..
gene-LOC131167056          AHWKAI..ESYTDAEAKAIEKFLEVFKDENFPPGSSILFTQSPL..GSL....TISFS..
Mamar.0037s0511.v1.1       AIWKSL..GIYTDCEAKAVEKFLEIFKDQTFPPGASILFALSPN..GAL....TIAFS..
Mamar.0043s0032.v1.1       EILKAS..GKYTRSEAKAIDKFLVVFKDQDFPPGSSVLFALCPK..GSL....TIAFS..
MD01G0093100               AFWKLV..GIYTDAEGKAIEMFLEVFKDQNFPPGASILFTQSPK..GSL....TISFS..
MD01G0093200               AFWKSI..GIYTDAEGKAIEKFLEVFKDQNFPPGASILFTQSPK..GSL....TISFS..
MD01G0093300               AFWKSI..GIYTDAEGKAIEKFLEVFKDQNFPPGASILFTQSPK..GSL....TISFS..
MD07G0154300               FFWKSV..GIYTDLEGKAIEQFIDVFKDQNFPPGASILFTQSPK..GSL....TISFS..
MD07G0154400               ASWKLA..GFYSGEEAKAIETFLEVFKDQTFPHGSSVSFTQSPPH.GSS....TISFS..
gene-LOC123192628          AIWKSL..GRYTDAESKAIEQFIKVFKDENFPPGASILFTISPL..GSL....TIGFS..
gene-LOC123227022          AIWKSL..GLYTDAESKAIEQFIDAFKDENFPPGASILFTLSPT..GSL....TIGFS..
gene-MANES_07G107200v8     AIWKSL..GIYTDAEAKAIDKFLEVFKSQTFPPGSSILFTQLPN..GSL....AISFS..
gene-LOC11445536           AIWKSL..GIYTDEEAKAIDKFVSVFKDETFPPGSSILFTVSPKGLGSL....TISFS..
gene-LOC11446745           AIWKSL..GIYTNEEAKAIKKFVSVFKDETFPPGSSILFTVSPKGLGSL....TISFS..
Mde013561.1                EILKSS..REYKRSEAKAIDKFLEVFKDKDFPPGSSVLFALCPK..GSL....IIAFS..
gene-MLD38_024177          AYWKSI..GKYTDAEAAAIEKFLETFKDETFAPGASILFTQSPD..GST....TIGFT..
gene-MERR_LOCUS28634       AIWKAL..GIYTDSEAKAVERFLEVFKDQTFPPGASILFALSPN..GSL....TIAFS..
Migut.D00159.v2.0          AYWKKV..EKYTDAESAAIEKFLQVFSDENFPPGSSILFTQSPE..GSL....TISFS..
gene-LOC111006117          AAWKSM..GIYTDEGAEAIQKFIDAFKNETFPPGSSILFTHLPP..NSL....SISFS..
MoBGI036118g0111           KYWKSI..GVYTDAEAKAIEKFLQLFQDETFPPGSSILFTQSPS..GSL....TISFS..
gene-L484_017264           AIWKTL..GIYTDAEEKAIEKFIEVFKDQNFPPGSSILFTQSPT..GSLKANKVISFS..
gene-LOC103981836          AQWKAA..GVYAEADGAALEQFKEAFRAETFPPGSSILFTHAPS..DSL....LIAFS..
gene-LOC104594676          NIMKAN..GKYGPKEEAAVHKFKEAFKGRNCPSGSSILITHTAS..GSA....TVAFS..
gene-LOC104599237          AYWKSV..GMYTDAESNAVEKFIEVFKDETFPPGASILFTQSPF..GSL....TIGFS..
CHI                        AHWKAI..GTYTDAESQAIEKFLNIFQNETFSPGASILFTQSPV..GAL....TISFI..
NC6G0256760                AIWKSA..GIYTDAEAQAVEKLKEVFKEQVFPPGSSIAMKHSTT..GSL....TIAFS..
gene-F0562_031805          AYWKAV..GIYTDAEGKAIEKFIEVFKDETFPPGASILFTQSPL..GSL....TIGFS..
ORUFI03G40690              AAWKAA..GVYTDAEGAADKFKEAFKPHSFPPGASILFTHSPA..GVL....TVAFS..
ORUFI05G23340              .........................FGKSE................GNY.......FE..
protein_coding_16416       AHWKAV..GIYTDAEAKAIEKFLEAFKDQTFQPGSSILFTQLPD..GSL....TISFS..
gene-PAHAL_3G142600        AYLKAT..GAYTDAEAAAVEEFNAAFKSHSLAPGASVLFTHSPA..GVL....TVAFS..
gene-PAHAL_9G032500        AYWKAV..GVYTDAEGAAVEKFKEAFKPETFPPGASILFTHSPA..GVL....TVAFS..
C5167_000623               EYLKSK..DMYTDAEAKAVERFIEIFKNEMFPPASSILFTISPA..GSL....TVGFS..
C5167_044917               QYLKSK..DMYTDAEAKAVERFIEIFKNEMFPPASSILFTMSPT..GSL....TVGFS..
C5167_046772               EMLTNS..KRYSETAKAALTKFSEAFNGRTLASGSSIHVTVSTS..NSV...........
gene-PanWU01x14_043220     AIWKSF..GIYTDAEAKAIEKFLEVFKDQNFAPGSSVLFTQSPS..GSL....TISFS..
gene-BS78_01G031900        AYWKAT..GVYTDAEGAAVDKFKEAFKPETFPPGASILFTHSPA..GVL....TVAFS..
gene-BS78_01G032000        AYWNATGGGVYTDAEGVAVDRFKEVFKPEMFPPGSSILFTYSPA..GVL....TVAFS..
gene-C2S52_017353          ACWKAV..GKYGDAESEAIDKFLQVFKDESFSPGASILYTQSPV..GSL....TIAFS..
gene-LOC110026679          AAWKAA..GEYTEEEATAINKFLEIFKPKNFLPGTSIIFTHSPH..GSL....TIGFL..
Phala.01G033500.v1.1       AGWEAA..GVYTEAEGKAVDKFKEAFKPQSFPPGSSILFSHSPN..GAL....TIAFS..
gene-PHAVU_002G276500g     AIWKSL..GIYTEAEAEAIDKFVSIFKDETFPPGSSILFTVLPK..GSL....LISFS..
gene-PHAVU_007G008500g     AIWKSL..GIYTDAEAEAIHKFVSVFKDQTFPPGSSILFTVLPK..GSL....LISFS..
gene-LOC103696411          AAWKAA..GIYTEAEGMAIEKFKEAFKAETYGPGSSILFTHSPS..GAL....TIASS..
gene-LOC108511484          LQAKHV..FTETNGAVDWVTSFVANHYGDHLWVGEA....ELPS..ALR....DIASS..
gene-PHJA_002477300        AYWQAV..GKYTDAESEAIKKFLEVFKPENFPPGASILFTQSPA..GSL....TISFS..
PIPE19196                  AIWKAA..GTYTDAEVRAVEEFKAAFRGRVFPPGSSILLAASSS..GTL....TIAFS..
PIPE22647                  AIWKAA..GTYTDAEVRAVEEFKAAFRGRVFPPGSSILLAASSS..GTL....TVRRR..
gene-LOC116107972          AIWKSL..GLYTDAESKAIEQFLEAFKDENFPPGASILFTLSPF..GSL....TIGFS..
gene-LOC116107975          AIWKSL..GLYTDAESKAIEQFLEAFKGENFPPGASILFTLSPF..GSL....TIGFS..
Psat6g237840               AIWKSL..GIYTEEEGKAIDKFVSIFKDETFPPGSSILFTVSPKGSGSL....SISFS..
Ptrif.0004s0460.v1.3.1     AIWKIF..GIYTDAEAKATEKFTEVFKDEIFPPGSSILFTQSP...GSL....TISFS..
gene-LOC118059901          RIWKSL..GIYTDAEAKAIEKFQEVFKEETFPPGSSILFTLLPH..GSL....AITFS..
Potri.010G213000.v4.1      SIWKSL..GIYTDAEAKAIEKFREVFKEETFPPGSSILFTLSPH..GSL....AISFS..
FUN_004111.v1.0            AFWKSI..GIYGEEEAKAVEKFCEVFKEEKFPSGHSILFTQSPH..GSL....TITFS..
PvLHv1_097350              AYWKSI..GTYTEAEALAIQQFIEVFETKNFPPGASILFTQSPI..GSL....TIGFS..
Pav_sc0007510.1_g020.1.mk  ........................................................FS..
Prudul26B016396            AIWKSI..GIYTDAEAKAIEKFLEVFKDQNFPPGASILFTQSPN..GSL....TISFS..
PRUPE_2G225200             AIWKSI..GIYTDAEAKAIEKFLEVFKDQNFPPGASILFTQSPN..GSL....TISFS..
gene-LOC116194939          AFWKSV..GTYTDAEARAVEKFIEVFKEETFPPGSSILFTQSPS..GSL....TIAFS..
```

```
                                 170             180       190       200       210
Medicago                         P.DTSIPE........KEAALIENKAVSSAVLETMIGEHAVSPDLKRCLAARLPALL.NE
Jr07_35760                       K.DESIPE........SGNAVIKNKLLSEAVLESIIGKHGVSPAAKKSLATRVSELL.KE
Kaladp0060s0328.v1.1             K.HDEIPK........EGNAVIDNKQLSEAVLESIIGQKGVSPEAKKSLATRLAELF.KE
geneMaker00000028                K.DGSMPE........VENAVIENRLLSEAVLESIVGKHGVSPEAKQSLAARLSAQM.IS
gene-GIB67_014039                K.DSSIPT........VQGVVIKNKPLSEAVLESIIGQNGVSPKVKRSLAKRLAKLL.QK
gene-GIB67_016345                K.HSSIPT........VQGVLIKNKPLSEAVLKSIIGQNGVSPKAKRSLAKRLAKLL.QK
gene-GIB67_037506                K.DGSIPE........VENAVIENRPLSGAILMSIIGPKGVSPDTKRSFAVRMSELL.KV
gene-GIB67_041777                K.DGSIPE........VENAVIENRPLSGAILMSIIGPKGVSPDTKRSFAVRMSELL.KV
gene-FCM35_KLT21231              K.DDSVPE........TNKLVIENRKLCRAVLESIIGEHGVSPAAKHSLAIRFCEHF.KS
Lsat_1_v5_gn_9_66221.v5          K.DGSIPE........APNVVLENEKLGQAVIESVIGRYGVSPATKQSLASRLSDFM.EQ
LPERR03G33170                    K.DSSVPEEE....DVAAAATIENRALCEAVLDSIIGEHGVSPAAKRSVAARLSELM.KA
Liphi.11G022900.v1.1             K.DDSIPE........KENAVIENKQLSEAVLESIIGKHGVSPSAKKSLALRLSELL.KQ
Lchi23460                        K.DSSIPE........QGNAVIENKQLKEAVLESIIGQHGVSPEAKQCLATRVSELL.KE
Lchi33204                        K.DSSIPE........QGNAVIENKQLKEAVLESIIGQHGVSPEAKQCLAMRVSELL.KE
Lchi33866                        K.DSSIPE........QGNAVIENKQLKEAVLESIIGQHGVSPEAKQCLAMRVSELL.KE
Lchi34914                        K.DSSIPE........QGNAVIENKQLKEAVLESIIGQHGVSPEAKQCLATRVSELL.KE
gene-Leryth_002794               K.DSSIPK........EGNVIIENQHLSEAVLESIIGRHGVSPQAKQSLAERLSELF.GK
Spe07827                         K.DDNIPE........TGEAVIENKLLAEAVLESIIGKEGVSPGARLSVAERLAHLM.KK
Spe20163                         K.GDNIPK........TGSAVIDNKLLAEAVLESIIGKNGVSPGARLSMAERLAQLM.KN
KYUSg_chr4.5001                  K.DSSLPE........SGGMAIENRPLCEAVLESIIGEHGVSPAAKLSLATRVAELL.KE
gene-LOC124680986                K.DSSLPE........SGGVAIENRPLCEAVLESIIGEHGVSPAAKLSLATRVAELL.KE
gene-LOC124695032                K.DSSVPE........SGGVAIENMPLCEAVLESILGEHGVSPAAKLSLATRVAELL.NE
Lj1A930T84                       K.SCCLPE........VGNAVIENKQLSQAILMSIIGKNGVSPETKQSLAERMSDLL.KK
Luann.0362s0045.v1.1             K.DERVPK........SGKAVIKNKLFAEAILESMIGKNGVSPATKNSLAHRLSKLM.MT
Luann.0427s0040.v1.1             K.DDNIPE........TGEAVIENKLLAEAVLESIIGKKGVSPGTRLSMAERLAQLM.KK
TanjilG_30460                    K.DASIPE........VETAIIENKLLSQAVLESMIGRHGVSPAAKQNLATRLSELL.KE
TanjilG_24711                    R.DAYIPK........VEAAIIKNKALSEAVLESMIGENGVSPAAKKSLATRLSKLF.KE
gene-LOC122057215                K.DGAIPE........VGSAVIENKQLAEAVLESIIGKHGVSPVAKQSLAERIHGLL.LE
gene-BVC80_1651g70               K.DDSIPE........VGNAVIENKALSEAILESIIGKNGVSPAAKHSLAVRISQLL.NK
gene-LOC131167055                KMNGTIPK........EGNAVLENKALSEAILESIIGKNGVSPGAKLSLAARLSNLL.SN
gene-LOC131167056                K.DGSIPE........VANSVIENKQLSEAVLESIIGKHGVSPATKQSLALRIEELL.KD
Mamar.0037s0511.v1.1             K.DDNIPE........TGNATIENKLLAEAVLESIIGKNGVSPGTRLSLAERLAHLMMKK
Mamar.0043s0032.v1.1             K.DERVPK........SGKAVIENKLLGEAVLESMIGKKGVSPATKKSLAERLSKLM.NK
MD01G0093100                     R.DASVPE........AANVVIENKLLSEAVLESIVGKHGVSPAAKQSLATRLSELL.NG
MD01G0093200                     R.DASVPE........AANTVIENKLLSEAVLESIVGKHGVSPAAKQSLAARLSELL.NG
MD01G0093300                     R.DASVPK........AANTVIENKLLSEAVLESIVGKHGVSPAAKQSLATRLSKLL.NG
MD07G0154300                     K.DASMPE........ATNAVIENKLLSETVLESIVGKHGVSPATKQSLAARLSQLL.NG
MD07G0154400                     K.DESIPQ........VGNEVMVNKLLGEAILESIIGKHGVSPAARQRLPQRLSDLL.TE
gene-LOC123192628                K.DECIRE........SGEVVIDNKLLAESVLESIIGKNGVSPAAKKNLAERFSILL.NG
gene-LOC123227022                K.NESIGE........SGKVVIDNKLLAESVLESIIGKNGVSPAAKKNLAERFSKLL.ND
gene-MANES_07G107200v8           K.DGAIPE........VENVVIQNKLLSEAVLESIIGKHGVSPETREIMATRLAELF.EN
gene-LOC11445536                 K.DGSIPE........VETAVIENKLLSQAVLESMIGAHGVSPAAKQSLASRLSKLF.KE
gene-LOC11446745                 K.DGSIPE........VETAVIENKLLSQAVLESMIGAHGVSPAAKQSLASRLSKLF.KE
Mde013561.1                      K.DERVPK........SGKAVINNKLFGEAILDSMIGKNGVSPATKKSLAHRLSKLI.NF
gene-MLD38_024177                N.DGTIPE........MGKAVITNRQLTDAILESIIGLKGVSPAARQSLASRLWGLM.GG
gene-MERR_LOCUS28634             K.DDSIPE........TGKAVIENKLLAEAVLESIIGKKGVSPGTRLSVAERLAQLM.KK
Migut.D00159.v2.0                K.DDSIPE........HGKAVIENKQLSEAVLESIIGRHGVSPSAKQSLAARISDLL.LK
gene-LOC111006117                K.DGSIPE........KGRATIENKLLSESVLESIIGKNGVSPAARLSLATRFSQLM.LQ
MoBGI036118g0111                 K.DGSIPE........TGNAVIENKLLAGAVLESIIGEHGVSPAAKRSLAARLAEVL.KE
gene-L484_017264                 K.DESIPE........KENVVIENKLLSEAVLESIIGKLGVSPAAKQSIASRLAELL.KE
gene-LOC103981836                K.DGSMPE........AGIAMIQNQPLSQGILESIIGENGVSPGAKRSLALRFSELL.KQ
gene-LOC104594676                N.GSFIPV........KGAAVIKNKALGEAYLLSVIGRQGVSPATKRSLARRLSNML.RS
gene-LOC104599237                K.DESIPE........EGNAVIENKPLSEAVLESIIGKHGVSPEAKQSLAERVSDLL.KD
CHI                              K.DDSVTG........TGNAVIENKQLSEAVLESIIGKHGVSPAAKCSIAERVSELF.KK
NC6G0256760                      K.DTSVPE........KGVAVIENKALTLSFLESVIGKHGVSPAAKRSVAERISGLL.KC
gene-F0562_031805                K.DGSLPE........EGTAIIENKHLSEAVLESIIGKHGVSPAAKQSLVARISELL.KE
ORUFI03G40690                    K.DSSVPE......DAVAAAAIENRALSEAVLDSIIGEHGVSPAAKRSIAARVSQLL.KA
ORUFI05G23340                    E.DQHTAECSVTHKVIVGLVQIENRTLSEAVLDSIIVEHGVSPAVKMSIAAHVSQLL.KA
protein_coding_16416             K.DHSMPE........GGNAVIKNKHLAEAILESIIGKHGVSPEARQSLAVRVSDLL.KK
gene-PAHAL_3G142600              D.DSSAPG........AGIAAIENKALCEAVLESIIGERSVSPATKQSIATRVPEIL.KG
gene-PAHAL_9G032500              K.DSSVPE........SGGVAIENKPLCEAVLESIIGEHGVSPAAKLSVAARVSELL.KE
C5167_000623                     K.DTSIPE........ARNAVIENKALSEAILESIIGKNGVSPAAKQSLAERISELL.KG
C5167_044917                     K.DTSIPE........ARNAVIENKALSESILESIIGKNGVSPAAKQSLAERISELL.KG
C5167_046772                     ..............TGDVTLDCKEVGEAFLMSTISLHTT...IRESMGSRISGLY.KS
gene-PanWU01x14_043220           K.DESIPE........AGNAVIENKLLSEAVLESIIGKNGVSPEARRSVASRLSELL.KE
gene-BS78_01G031900              K.DSSVPA........AGAVAIENKRLCEAVLESIIGEHGVSPAAKLSIAARVSELL.KG
gene-BS78_01G032000              K.DSSVPE........AGGVAIENKPLCEAVLESIIGEKGVSPAAKLSIAARVSELL.KG
gene-C2S52_017353                K.DASIPD........QGKAVIQNKLLAETILHSIIGKHGVSPAARQSLAARLSHLF.NQ
gene-LOC110026679                E.GDGVPV........AETDVIESKKLTNAVLESIIGENGVSPAAKQSLARRFSELL.NK
Phala.01G033500.v1.1             K.DTSVPE........DGSAVIENKALCEAVLESIIGEHGVSPAAKRSLAERVSVLL.KG
gene-PHAVU_002G276500g           K.DGAIPE........EASTVIENKLLSEAVLESMIGKHGVSPAAKQSLASRLSELF.KE
gene-PHAVU_007G008500g           K.DGSIPK........EVIAVIENKLLSEAVLESMIGKHGVSPAAKQSLASRLSELF.KQ
gene-LOC103696411                E.DGSMPE........AGRVVIENKALCGAILESIIGEHGVSPAAKRSLAVRLSELL.KE
gene-LOC108511484                E.DGSMPE........AGRGVIENKALCVAILESIIGEHGVSPAAERSLAVRLSERL.KE
gene-PHJA_002477300              R.DDSIPE........KANAVIENKQLSEAVLESIIGKHGVSPSAKQSLALRLSEML.KQ
PIPE19196                        K.DGVVPE........AGDVVVESSAMAEAFLESVIGQNGVSPAAKKSLAARVSDLL.KK
PIPE22647                        P.AGGGRR........GG....REQAMAEAFLESVIGQNGVSPAAKKSLAARVSDLL.KK
gene-LOC116107972                K.DESIGE........SGKVVIDNKLLAESVLESIIGKNGVSPAARKNLAERFSKLL.NG
gene-LOC116107975                K.DESIGE........SGKVVIDNKLLAESVLESIIGKNGVSPGARKNLAERFSKLL.NG
Psat6g237840                     K.DGSIPE........VESAVIENKLLSQAVLESMIGAHGVSPAAKQSLASRLPELF.KE
Ptrif.0004s0460.v1.3.1           K.DGSIPK........DGVAVIENNLLSEAVLESMIGKNGVSPAAKKSLAERLSALL.NV
gene-LOC118059901                K.DGSVPE........IENAVIENKLLSEAVLESMIGKHGVSPEAKQSLAATLSELL.KE
Potri.010G213000.v4.1            K.DGSVPE........IENAVIENKLLSEAVLESMIGKHGVSPAAKQSLATTLSELL.KE
FUN_004111.v1.0                  K.HDLIPE........TGKLVIENKHIAEAVLQSIIGKHGVSPAAKQSLASRLSKLL.SN
PvLHv1_097350                    F.DGLFPE........HGEVVIDNKQLSEAVLESIIGVHGVSPAAKQSLAARLSNIF...
Pav_sc0007510.1_g020.1.mk        K.DASVPE........AGNVVIENKLLSEAVLESIIGKHGVSPGARQSVAARLSELL.KY
Prudul26B016396                  K.DASVPE........AGNVVIENKLLSEAVLESIIGKHGVSPGARQSVAARLSELL.K.
PRUPE_2G225200                   K.DASVPE........AGNVVIENKLLSEAVLESIIGKHGVSPGARQSVAARLSELL.K.
gene-LOC116194939                K.DGKIPE........DGRAVIENKQLSEAVLESIIGKHGVSPEAKQCLATRISELL.KG
```

```
                                  220
                                    .
Medicago                          GAF.K.I.................GN..................................
Jr07_35760                        SND.K.E.................AE.NH............K................LK
Kaladp0060s0328.v1.1              KHG.H.S.................EE.NEV.ASG.....KPEPE............KSVA
geneMaker00000028                 PSI.A.K...................................................IQ
gene-GIB67_014039                 KHY.........................................................
gene-GIB67_016345                 KHY.........................................................
gene-GIB67_037506                 EKV.E.V.................E...................................
gene-GIB67_041777                 EEV.E.V.................E...................................
gene-FCM35_KLT21231               QSA.A.N.................QE.EVH.........VENP.............VTIN
Lsat_1_v5_gn_9_66221.v5           IEG.K.A.................TE.TES.VEL.....GKNSL................
LPERR03G33170                     GAT.G.D.................VP.PPP.AS........AA.............AAVS
Liphi.11G022900.v1.1              YEF.K.V.................VD.HE...............................
Lchi23460                         FDP.V.K.................EG..................................
Lchi33204                         FDP.V.K.................EG..................................
Lchi33866                         FDP.V.E.................EG...........................YLVSRLG
Lchi34914                         FDP.V.K.................EG..................................
gene-Leryth_002794                YKD.D.D.................EV.KQR.G............................
Spe07827                          NDI.V.K.................EE.EVT.KND.....HQEEGG...............
Spe20163                          NKV.K.E.................GQ.EAT.RTN.....QEKTRD...........LSHR
KYUSg_chr4.5001                   AA....P.................VG.EPA.VA........EP.............VSVS
gene-LOC124680986                 AA....P.................VS.EPA.VA........EP.............VSVS
gene-LOC124695032                 AA....P.................VG.Q.A.AA........EP.............VSVS
Lj1A930T84                        YDE.K.A.................SR.NGE............................LE
Luann.0362s0045.v1.1              NKD.E.HTIY..............EA.NKS..............................
Luann.0427s0040.v1.1              NKV.Q.D.................EA................................IE
TanjilG_30460                     GGA.N.......................................................
TanjilG_24711                     GCA.N.......................................................
gene-LOC122057215                 FDA.A.N.................VN.VNA.NAL.....NRDK.............VEVV
gene-BVC80_1651g70                GHG.D.K.................KMFRTY..............................
gene-LOC131167055                 ............................................................
gene-LOC131167056                 VRA.A.G.................NE.K..............................IE
Mamar.0037s0511.v1.1              NKA.D.E.................EA.S................D...........LSLE
Mamar.0043s0032.v1.1              QQG.P.TR....................................................
MD01G0093100                      CKE.S.NGV..............EAG.NE............R................AE
MD01G0093200                      CKE.S.YRA..............EAG.NE............K................VE
MD01G0093300                      CKE.S.YGA..............KAR.NE............K................VE
MD07G0154300                      CK..........................................................
MD07G0154400                      KPEL...................LGV.GKP.TNQ......................VEVG
gene-LOC123192628                 EVK.S.E.................AD.HCA.KEF......................KGVD
gene-LOC123227022                 GEK.KPE.................AD.NCA.KDF......................KGLD
gene-MANES_07G107200v8            NSQ.I.N.................GI.NHT.LQA.....IQRESIMSPA...TASVSQIE
gene-LOC11445536                  GGN.A.N.................N...................................
gene-LOC11446745                  EDE.D.S.................VL.Q................................
Mde013561.1                       T...........................................................
gene-MLD38_024177                 GCE.G.GKVVEGGEQKVECNGINGEA.GKA.EPVVV........................
gene-MERR_LOCUS28634              NKV.E.E.................EA.SDV..........................LSVE
Migut.D00159.v2.0                 PNE.P.E.................EV.VAA.TAK.....PQEN.................
gene-LOC111006117                 N...........................................................
MoBGI036118g0111                  CKN.T.G.................VV.LDE.KLE.....AKKNTE...........ETVE
gene-L484_017264                  TKD.........................................................
gene-LOC103981836                 HCE.A.E.................ET.KLV...........NP.............VAVI
gene-LOC104594676                 ............................................................
gene-LOC104599237                 VGA.E.K.................VD.GDT.....N..LVNK...........FIVEKVE
CHI                               SYA.D.A.................SV.CEN..............................
NC6G0256760                       AEA.........................................................
gene-F0562_031805                 CDD.K.G..................................R................ME
ORUFI03G40690                     EST.G.D.................V....A.PA........EP.............APVS
ORUFI05G23340                     EST.A.G................LDA.TGG.RGGVNAMAPRRETTS...MRRRRGGLDAT
protein_coding_16416              FDD.K.A.................A...................................
gene-PAHAL_3G142600               GA..........................................................
gene-PAHAL_9G032500               ASP.....................AG.GPPQAA........EPA............VPVS
C5167_000623                      YEN.K.P.................DD.SAA.AKT.....EEETTKA..............
C5167_044917                      YEN.K.P.................DE.SAA.AKN.....EEETTKA..............
C5167_046772                      KIII..D.................DG.PKN.DIN.....GPKNFH...........LDIN
gene-PanWU01x14_043220            NTT.I.A.................TA.TNT.TT..........................TE
gene-BS78_01G031900               TVD.....................AA.DAA.QA.........GA.............VPVS
gene-BS78_01G032000               TAD.D.D.................A....A.QE........GA.............VPVS
gene-C2S52_017353                 N...........................................................
gene-LOC110026679                 KED.Q.E.................EE.DGI..........................LDVE
Phala.01G033500.v1.1              ASD.A.G.................AT.PMP..........................MSVS
gene-PHAVU_002G276500g            GGV.P.E.................SH.N................................
gene-PHAVU_007G008500g            G...........................................................
gene-LOC103696411                 SQD.G.E.................E....K.VG........NP.............VPVR
gene-LOC108511484                 SQD.G.E.................EK.VGN.P........................VPVR
gene-PHJA_002477300               HEP.K.V.................AK.HE...............................
PIPE19196                         EHG.G.......................................................
PIPE22647                         EHG.G.......................................................
gene-LOC116107972                 KEK.S.E.................AD.NCA.KEF......................KGVD
gene-LOC116107975                 EEK.S.E.................AD.NCA.KEF......................KGVD
Psat6g237840                      AGD.A.N.................N...................................
Ptrif.0004s0460.v1.3.1            TSD.K.M..................................K..................
gene-LOC118059901                 SNE.N.G.................N...................................
Potri.010G213000.v4.1             SNE.N.G.................N...................................
FUN_004111.v1.0                   NEQ.E.T.................NS.AE...........................ISLG
PvLHv1_097350                     ............................................................
Pav_sc0007510.1_g020.1.mk         SCH.N.E.................AG.NGK..........................LETQ
Prudul26B016396                   ............................................................
PRUPE_2G225200                    ............................................................
gene-LOC116194939                 VSE.K.K.................NE..................................
```

```
Medicago                         ............................................................
Jr07_35760                       A.....................ENHKLE................................
Kaladp0060s0328.v1.1             PVDGD.............QKPSEQINL.................................
geneMaker00000028                V.....................EDVKFLPTTKPPASTKTHFLGGAGARGLEIEGKFIKFT
gene-GIB67_014039                ............................................................
gene-GIB67_016345                ............................................................
gene-GIB67_037506                ............................................................
gene-GIB67_041777                ............................................................
gene-FCM35_KLT21231              A...........................................................
Lsat_1_v5_gn_9_66221.v5          ............................................................
LPERR03G33170                    A...........................................................
Liphi.11G022900.v1.1             ............................................................
Lchi23460                        ............................................................
Lchi33204                        ............................................................
Lchi33866                        GNFFA..............LAR......................................
Lchi34914                        ............................................................
gene-Leryth_002794               ............................................................
Spe07827                         DKLA.................KEN....................................
Spe20163                         DQYA.................KEN....................................
KYUSg_chr4.5001                  A...........................................................
gene-LOC124680986                A...........................................................
gene-LOC124695032                A...........................................................
Lj1A930T84                       S.....................KKAILKEIVV............................
Luann.0362s0045.v1.1             ............................................................
Luann.0427s0040.v1.1             DKLA.................KEN....................................
TanjilG_30460                    ............................................................
TanjilG_24711                    ............................................................
gene-LOC122057215                KQENGDSV......DKVEVVKQENGDSVDKVE............................
gene-BVC80_1651g70               ............................................................
gene-LOC131167055                ............................................................
gene-LOC131167056                A.....................KKLPAE................................
Mamar.0037s0511.v1.1             EKLA.................KEN....................................
Mamar.0043s0032.v1.1             ............................................................
MD01G0093100                     A...........................................................
MD01G0093200                     A...........................................................
MD01G0093300                     A...........................................................
MD07G0154300                     ............................................................
MD07G0154400                     GN..........................................................
gene-LOC123192628                TNFIQ................VETGKV.................................
gene-LOC123227022                HKINQ................IETGKV.................................
gene-MANES_07G107200v8           V.....................ENIAFPPAAKPPASNKTLFLGGAGDRGLEIQGKFVKFT
gene-LOC11445536                 ............................................................
gene-LOC11446745                 ............................................................
Mde013561.1                      ............................................................
gene-MLD38_024177                ............................................................
gene-MERR_LOCUS28634             DKVA.................KEN....................................
Migut.D00159.v2.0                ............................................................
gene-LOC111006117                ............................................................
MoBGI036118g0111                 AH....................EKGIEVDAGKP...........................
gene-L484_017264                 ............................................................
gene-LOC103981836                V...........................................................
gene-LOC104594676                ............................................................
gene-LOC104599237                V.....................ESFSFPPSVKPPGSTKTLFLGGAGSRGLEIQGQFIKFT
CHI                              ...................PGIEKSSDPVIEEKPT.........................
NC6G0256760                      ............................................................
gene-F0562_031805                S...........................................................
ORUFI03G40690                    A...........................................................
ORUFI05G23340                    AMRQMEGRRRGWHG..............................................
protein_coding_16416             ............................................................
gene-PAHAL_3G142600              ............................................................
gene-PAHAL_9G032500              A...........................................................
C5167_000623                     ............................................................
C5167_044917                     ............................................................
C5167_046772                     LGTGQKPL...........FDLDNTET.................................
gene-PanWU01x14_043220           A.....................EKFVQVK...............................
gene-BS78_01G031900              A...........................................................
gene-BS78_01G032000              A...........................................................
gene-C2S52_017353                ............................................................
gene-LOC110026679                KA..................KLGQDGVV................................
Phala.01G033500.v1.1             L...........................................................
gene-PHAVU_002G276500g           ............................................................
gene-PHAVU_007G008500g           ............................................................
gene-LOC103696411                A...........................................................
gene-LOC108511484                A...........................................................
gene-PHJA_002477300              ............................................................
PIPE19196                        ............................................................
PIPE22647                        ............................................................
gene-LOC116107972                TNIIQ................IETGKV.................................
gene-LOC116107975                TNIIQ................VETGKV.................................
Psat6g237840                     ............................................................
Ptrif.0004s0460.v1.3.1           ............................................................
gene-LOC118059901                ............................................................
Potri.010G213000.v4.1            ............................................................
FUN_004111.v1.0                  AKLL.................SETHSQRQHLTA...........................
PvLHv1_097350                    ............................................................
Pav_sc0007510.1_g020.1.mk        V...........................................................
Prudul26B016396                  ............................................................
PRUPE_2G225200                   ............................................................
gene-LOC116194939                ............................................................
```

```
Medicago                         ............................................................
Jr07_35760                       ............................................................
Kaladp0060s0328.v1.1             ............................................................
geneMaker00000028                AIGVYLEDSAVPFLAVKWKGKSAQELTDSVEFFRDIVTGPFEKFIRVTMILPLTGQQYSE
gene-GIB67_014039                ............................................................
gene-GIB67_016345                ............................................................
gene-GIB67_037506                ............................................................
gene-GIB67_041777                ............................................................
gene-FCM35_KLT21231              ............................................................
Lsat_1_v5_gn_9_66221.v5          ............................................................
LPERR03G33170                    ............................................................
Liphi.11G022900.v1.1             ............................................................
Lchi23460                        ............................................................
Lchi33204                        ............................................................
Lchi33866                        ............................................................
Lchi34914                        ............................................................
gene-Leryth_002794               ............................................................
Spe07827                         ............................................................
Spe20163                         ............................................................
KYUSg_chr4.5001                  ............................................................
gene-LOC124680986                ............................................................
gene-LOC124695032                ............................................................
Lj1A930T84                       ............................................................
Luann.0362s0045.v1.1             ............................................................
Luann.0427s0040.v1.1             ............................................................
TanjilG_30460                    ............................................................
TanjilG_24711                    ............................................................
gene-LOC122057215                ............................................................
gene-BVC80_1651g70               ............................................................
gene-LOC131167055                ............................................................
gene-LOC131167056                ............................................................
Mamar.0037s0511.v1.1             ............................................................
Mamar.0043s0032.v1.1             ............................................................
MD01G0093100                     ............................................................
MD01G0093200                     ............................................................
MD01G0093300                     ............................................................
MD07G0154300                     ............................................................
MD07G0154400                     ............................................................
gene-LOC123192628                ............................................................
gene-LOC123227022                ............................................................
gene-MANES_07G107200v8           AIGVYLEDEAVPLLAVKWKGKSGKDLVDSIEFFRDIVTGPFEKCVRVTMILPLTGQQYSE
gene-LOC11445536                 ............................................................
gene-LOC11446745                 ............................................................
Mde013561.1                      ............................................................
gene-MLD38_024177                ............................................................
gene-MERR_LOCUS28634             ............................................................
Migut.D00159.v2.0                ............................................................
gene-LOC111006117                ............................................................
MoBGI036118g0111                 ............................................................
gene-L484_017264                 ............................................................
gene-LOC103981836                ............................................................
gene-LOC104594676                ............................................................
gene-LOC104599237                AIGVYLEDSTVPSLAVKWKGKTADELTDSVEFFRDIITGPFEKFIQVTMIKPLTGQQYSE
CHI                              ............................................................
NC6G0256760                      ............................................................
gene-F0562_031805                ............................................................
ORUFI03G40690                    ............................................................
ORUFI05G23340                    ............................................................
protein_coding_16416             ............................................................
gene-PAHAL_3G142600              ............................................................
gene-PAHAL_9G032500              ............................................................
C5167_000623                     ............................................................
C5167_044917                     ............................................................
C5167_046772                     ............................................................
gene-PanWU01x14_043220           ............................................................
gene-BS78_01G031900              ............................................................
gene-BS78_01G032000              ............................................................
gene-C2S52_017353                ............................................................
gene-LOC110026679                ............................................................
Phala.01G033500.v1.1             ............................................................
gene-PHAVU_002G276500g           ............................................................
gene-PHAVU_007G008500g           ............................................................
gene-LOC103696411                ............................................................
gene-LOC108511484                ............................................................
gene-PHJA_002477300              ............................................................
PIPE19196                        ............................................................
PIPE22647                        ............................................................
gene-LOC116107972                ............................................................
gene-LOC116107975                ............................................................
Psat6g237840                     ............................................................
Ptrif.0004s0460.v1.3.1           ............................................................
gene-LOC118059901                ............................................................
Potri.010G213000.v4.1            ............................................................
FUN_004111.v1.0                  ............................................................
PvLHv1_097350                    ............................................................
Pav_sc0007510.1_g020.1.mk        ............................................................
Prudul26B016396                  ............................................................
PRUPE_2G225200                   ............................................................
gene-LOC116194939                ............................................................
```

**Medicago**	....
**Jr07_35760**	..TEN.......................KIL..........................KAE
**Kaladp0060s0328.v1.1**	....
**geneMaker00000028**	KVAENCVAFWKSVGLYTDAEAKAIETFLEVFKEEKFPPGSSILFTQLPRGSLAISFSKDG
**gene-GIB67_014039**	....
**gene-GIB67_016345**	....
**gene-GIB67_037506**	....
**gene-GIB67_041777**	....
**gene-FCM35_KLT21231**	....
**Lsat_1_v5_gn_9_66221.v5**	....
**LPERR03G33170**	....
**Liphi.11G022900.v1.1**	....
**Lchi23460**	....
**Lchi33204**	....
**Lchi33866**	....
**Lchi34914**	....
**gene-Leryth_002794**	....
**Spe07827**	....
**Spe20163**	....
**KYUSg_chr4.5001**	....
**gene-LOC124680986**	....
**gene-LOC124695032**	....
**Lj1A930T84**	....
**Luann.0362s0045.v1.1**	....
**Luann.0427s0040.v1.1**	....
**TanjilG_30460**	....
**TanjilG_24711**	....
**gene-LOC122057215**	....
**gene-BVC80_1651g70**	....
**gene-LOC131167055**	....
**gene-LOC131167056**	........................QKPMAL..............................
**Mamar.0037s0511.v1.1**	....
**Mamar.0043s0032.v1.1**	....
**MD01G0093100**	....
**MD01G0093200**	....
**MD01G0093300**	....
**MD07G0154300**	....
**MD07G0154400**	....
**gene-LOC123192628**	....
**gene-LOC123227022**	....
**gene-MANES_07G107200v8**	KVTENCVAIWKSLGIYTDAEAKAIDKFLEVFKAETFPPGSSIIFTLLPRGALAISFSKDG
**gene-LOC11445536**	....
**gene-LOC11446745**	....
**Mde013561.1**	....
**gene-MLD38_024177**	....
**gene-MERR_LOCUS28634**	....
**Migut.D00159.v2.0**	....
**gene-LOC111006117**	....
**MoBGI036118g0111**	....
**gene-L484_017264**	....
**gene-LOC103981836**	....
**gene-LOC104594676**	....
**gene-LOC104599237**	KVTENCVAYWKSVGIYTESEAKAVEKFIEVFEDETFPPGASILFTQTPHGSLTIGFSKDG
**CHI**	....
**NC6G0256760**	....
**gene-F0562_031805**	....
**ORUFI03G40690**	....
**ORUFI05G23340**	....GT..
**protein_coding_16416**	....
**gene-PAHAL_3G142600**	....
**gene-PAHAL_9G032500**	....
**C5167_000623**	....
**C5167_044917**	....
**C5167_046772**	....
**gene-PanWU01x14_043220**	....
**gene-BS78_01G031900**	....
**gene-BS78_01G032000**	....
**gene-C2S52_017353**	....
**gene-LOC110026679**	....
**Phala.01G033500.v1.1**	....
**gene-PHAVU_002G276500g**	....
**gene-PHAVU_007G008500g**	....
**gene-LOC103696411**	....
**gene-LOC108511484**	....
**gene-PHJA_002477300**	....
**PIPE19196**	....
**PIPE22647**	....
**gene-LOC116107972**	....
**gene-LOC116107975**	....
**Psat6g237840**	....
**Ptrif.0004s0460.v1.3.1**	....
**gene-LOC118059901**	....
**Potri.010G213000.v4.1**	....
**FUN_004111.v1.0**	....
**PvLHv1_097350**	....
**Pav_sc0007510.1_g020.1.mk**	....
**Prudul26B016396**	....
**PRUPE_2G225200**	....
**gene-LOC116194939**	....

```
Medicago                        ....................................................
Jr07_35760                      N.SEIGGPK...........................................
Kaladp0060s0328.v1.1            ....................................................
geneMaker00000028               SQPEVENSLIENKLLSEAVLE..SIIGKHGVSPEAKHSLASRLSGFF.....
gene-GIB67_014039               ....................................................
gene-GIB67_016345               ....................................................
gene-GIB67_037506               ....................................................
gene-GIB67_041777               ....................................................
gene-FCM35_KLT21231             ....................................................
Lsat_1_v5_gn_9_66221.v5         ....................................................
LPERR03G33170                   ....................................................
Liphi.11G022900.v1.1            ....................................................
Lchi23460                       ....................................................
Lchi33204                       ....................................................
Lchi33866                       ....................................................
Lchi34914                       ....................................................
gene-Leryth_002794              ....................................................
Spe07827                        ....................................................
Spe20163                        ....................................................
KYUSg_chr4.5001                 ....................................................
gene-LOC124680986               ....................................................
gene-LOC124695032               ....................................................
Lj1A930T84                      ....................................................
Luann.0362s0045.v1.1            ................................................ATHH
Luann.0427s0040.v1.1            ....................................................
TanjilG_30460                   ....................................................
TanjilG_24711                   ....................................................
gene-LOC122057215               .VVKQENG....................DSICK..................Q
gene-BVC80_1651g70              ...................................................K
gene-LOC131167055               ....................................................
gene-LOC131167056               ..EPVSVGVV................GENGK.....................
Mamar.0037s0511.v1.1            ....................................................
Mamar.0043s0032.v1.1            ....................................................
MD01G0093100                    ....................................................
MD01G0093200                    ....................................................
MD01G0093300                    ....................................................
MD07G0154300                    ....................................................
MD07G0154400                    ....................................................
gene-LOC123192628               ....................................................
gene-LOC123227022               ....................................................
gene-MANES_07G107200v8          EIPEVENELIENKLLAEAVLE..SIIGKHGVSPAARESLATRLAELI....I
gene-LOC11445536                ....................................................
gene-LOC11446745                ....................................................
Mde013561.1                     ....................................................
gene-MLD38_024177               ....................................................
gene-MERR_LOCUS28634            ....................................................
Migut.D00159.v2.0               ....................................................
gene-LOC111006117               ....................................................
MoBGI036118g0111                ....................................................
gene-L484_017264                ....................................................
gene-LOC103981836               ....................................................
gene-LOC104594676               ....................................................
gene-LOC104599237               SIPEVGNAVIENRPLSEAVLE..SIIGKHGVSPEAKRNLAERVSKLL..EKF
CHI                             .IPE.............................................IGV
NC6G0256760                     ....................................................
gene-F0562_031805               ....................................................
ORUFI03G40690                   ....................................................
ORUFI05G23340                   .....................RYWSSGRGTI................TVDLL
protein_coding_16416            ....................................................
gene-PAHAL_3G142600             ....................................................
gene-PAHAL_9G032500             ....................................................
C5167_000623                    ....................................................
C5167_044917                    ....................................................
C5167_046772                    ....................................................
gene-PanWU01x14_043220          ....................................................
gene-BS78_01G031900             ....................................................
gene-BS78_01G032000             ....................................................
gene-C2S52_017353               ....................................................
gene-LOC110026679               ....................................................
Phala.01G033500.v1.1            ....................................................
gene-PHAVU_002G276500g          ....................................................
gene-PHAVU_007G008500g          ....................................................
gene-LOC103696411               ....................................................
gene-LOC108511484               ....................................................
gene-PHJA_002477300             ....................................................
PIPE19196                       ...................................................V
PIPE22647                       ...................................................V
gene-LOC116107972               ....................................................
gene-LOC116107975               ....................................................
Psat6g237840                    ....................................................
Ptrif.0004s0460.v1.3.1          ....................................................
gene-LOC118059901               ....................................................
Potri.010G213000.v4.1           ....................................................
FUN_004111.v1.0                 ....................................................
PvLHv1_097350                   ....................................................
Pav_sc0007510.1_g020.1.mk       ....................................................
Prudul26B016396                 ....................................................
PRUPE_2G225200                  ....................................................
gene-LOC116194939               ....................................................
```

**Medicago**	....
**Chr16.g28500**	....
**Chr1.g57423**	....
**Chr7.g32642**	MTSLPSVTP.VQVETMMFPPTTKPPGSANILFLGGARVRGLPIEGKLVKFTVIGVYLEDN
**QL08p057674**	....
**gene-CFP56_42925**	....
**gene-LOC108830048**	....
**gene-LOC108862091**	....
**gene-LOC115736770**	....
**gene-C3L33_05584**	....
**gene-LUZ62_010432**	MGT...
**gene-LUZ62_034283**	M...
**gene-LUZ62_049060**	M...
**gene-LUZ62_054959**	MGT...
**gene-LUZ62_079350**	M...
**gene-LUZ62_083640**	M...
**gene-LOC8264259**	....
**RchiOBHm_Chr1g0365111**	....
**RchiOBHm_Chr7g0221431**	....
**Rf070256510**	....
**Sspon.01G0031250-1A**	....
**Sspon.01G0031250-1T**	....
**Sspon.01G0031250-3C**	....
**ztl01450**	....
**Sapur.010G169300.v5.1**	....
**NDS_009150**	....
**NDS_061126**	MNEQGC..
**NDS_061132**	....
**Samuk03G0051900**	....
**Samuk03G0054000**	....
**Sb000535g0022**	MQTSPCVSK.IQVENVAFSPTIIPPGSTKTHFLGGAGERGLDIQGKFVKFTAIGVYVEDK
**EVM20prediction20contig126.452**	....
**SECCE5Rv1G0364630**	....
**gene-G2W53_032366**	....
**SETIT_024113mg**	....
**SETIT_036958mg**	....
**SEVIR_9G033800v2**	....
**Sc06g0001890**	....
**Sc06g0001900**	....
**Solyc05g010310.3**	....
**Solyc05g010320.3**	....
**gene-LOC107019217**	....
**gene-LOC107020630**	....
**SORBI_3001G035600**	....
**gene-E2542_SST22123**	....
**gene-LOC110795717**	....
**gene-SI8410_06008823**	....
**gene-STAS_11501**	MAKSLF...FTLI.......
**EVM0031032**	....
**TkA04G183730**	....
**gene-LOC104825165**	....
**gene-HHK36_016491**	....
**gene-FRX31_032955**	....
**TCM_042994**	MKNKQR....FNLQNV..
**Tel5E01G756500**	....
**gene-TAV2_LOCUS5378**	....
**gene-TAV2_LOCUS16152**	....
**EVM20prediction20LG01.1567**	....
**gene-TorRG33x02_127840**	....
**Tp57577_TGAC_v2_gene10384**	....
**gene-HS088_TW21G01722**	....
**TraesCS5A02G475600**	....
**TraesCS5B02G488900**	....
**TraesCS7B02G038200**	....
**Tubocapsicum_anomalum_10G026880**	....
**Tubocapsicum_anomalum_17G002220**	....
**Urofu.9G032800.v1.1**	....
**VducChr11G242040**	....
**vmacro07205_Vaccinium_macrocarpon_Stevens_v1**	....
**gene-HPP92_020856**	MAATGV..
**tung.gene.scaffold2716.00003**	....
**gene-LOC114190515**	....
**gene-VitviT2T_019145**	M...
**W.mirabilis.00703**	MGFSFEQFPAVEVEGIEFPQQEKAVGSLKPTFLAGAGVRGLTIGGTFVAFTAIGVYGEES
**gene-JRO89_XSUnG0164900**	....
**Zm00001eb062510**	....
**gene-ZIOFF_031498**	....
**gene-ZIOFF_035070**	....
**Zla09G001950**	....
**gene-FEM48_Zijuj03G0079900**	....
**gene-FEM48_Zijuj03G0079800**	....
**gene-ZOSMA_1G00350**	....
**AT3G55120**	M...
**Os03g60509**	....

**Medicago**	....
**Chr16.g28500**	....
**Chr1.g57423**	....
**Chr7.g32642**	AVPVLA...........LANSVEFFKDVVTGPFEKVTQVTFIQHLTGRQYSEKVAENCIA
**QL08p057674**	....
**gene-CFP56_42925**	....
**gene-LOC108830048**	....
**gene-LOC108862091**	....
**gene-LOC115736770**	....
**gene-C3L33_05584**	....
**gene-LUZ62_010432**	....
**gene-LUZ62_034283**	....
**gene-LUZ62_049060**	....
**gene-LUZ62_054959**	....
**gene-LUZ62_079350**	....
**gene-LUZ62_083640**	....
**gene-LOC8264259**	....
**RchiOBHm_Chr1g0365111**	....
**RchiOBHm_Chr7g0221431**	....
**Rf070256510**	....
**Sspon.01G0031250-1A**	....
**Sspon.01G0031250-1T**	....
**Sspon.01G0031250-3C**	....
**ztl01450**	....
**Sapur.010G169300.v5.1**	....
**NDS_009150**	....
**NDS_061126**	....
**NDS_061132**	....
**Samuk03G0051900**	....
**Samuk03G0054000**	....
**Sb000535g0022**	AVSSLAGKWKG.KSAEELTESVEFFRDIVTGPFEKFMQVTMILPLTGQQYSEKVSENCVA
**EVM20prediction20contig126.452**	....
**SECCE5Rv1G0364630**	....
**gene-G2W53_032366**	....
**SETIT_024113mg**	....
**SETIT_036958mg**	....
**SEVIR_9G033800v2**	....
**Sc06g0001890**	....
**Sc06g0001900**	....
**Solyc05g010310.3**	....
**Solyc05g010320.3**	....
**gene-LOC107019217**	....
**gene-LOC107020630**	....
**SORBI_3001G035600**	....
**gene-E2542_SST22123**	....
**gene-LOC110795717**	....
**gene-SI8410_06008823**	....
**gene-STAS_11501**	...................................LLFMSIMRPTHSR............
**EVM0031032**	....
**TkA04G183730**	....
**gene-LOC104825165**	....
**gene-HHK36_016491**	....
**gene-FRX31_032955**	....
**TCM_042994**	............HSQ.............VTCPY.......LPLPLSTN............
**Tel5E01G756500**	....
**gene-TAV2_LOCUS5378**	....
**gene-TAV2_LOCUS16152**	....
**EVM20prediction20LG01.1567**	....
**gene-TorRG33x02_127840**	....
**Tp57577_TGAC_v2_gene10384**	....
**gene-HS088_TW21G01722**	....
**TraesCS5A02G475600**	....
**TraesCS5B02G488900**	....
**TraesCS7B02G038200**	....
**Tubocapsicum_anomalum_10G026880**	....
**Tubocapsicum_anomalum_17G002220**	....
**Urofu.9G032800.v1.1**	....
**VducChr11G242040**	....
**vmacro07205_Vaccinium_macrocarpon_Stevens_v1**	....
**gene-HPP92_020856**	....
**tung.gene.scaffold2716.00003**	....
**gene-LOC114190515**	....
**gene-VitviT2T_019145**	....
**W.mirabilis.00703**	LFSYLQSKWASYRSPQDLQDNLDFFMDIVTCPFLKFIRVTMIKPLSGSQYSGKVCEGCIA
**gene-JRO89_XSUnG0164900**	....
**Zm00001eb062510**	....
**gene-ZIOFF_031498**	....
**gene-ZIOFF_035070**	....
**Zla09G001950**	....
**gene-FEM48_Zijuj03G0079900**	....
**gene-FEM48_Zijuj03G0079800**	....
**gene-ZOSMA_1G00350**	....
**AT3G55120**	....
**Os03g60509**	....

Medicago	....
Chr16.g28500	....
Chr1.g57423	....
Chr7.g32642	SWK...SAGFYSGEEAKAIETFLEVFKDQTFPP...GSSVSFVQSPPHGSLTISLSKDGS
QL08p057674	....
gene-CFP56_42925	....
gene-LOC108830048	....
gene-LOC108862091	....
gene-LOC115736770	....
gene-C3L33_05584	....
gene-LUZ62_010432	....
gene-LUZ62_034283	....
gene-LUZ62_049060	....
gene-LUZ62_054959	....
gene-LUZ62_079350	....
gene-LUZ62_083640	....
gene-LOC8264259	....
RchiOBHm_Chr1g0365111	....
RchiOBHm_Chr7g0221431	....
Rf070256510	....
Sspon.01G0031250-1A	....
Sspon.01G0031250-1T	....
Sspon.01G0031250-3C	....
ztl01450	....
Sapur.010G169300.v5.1	....
NDS_009150	....
NDS_061126	..............................YTPL.LLTTFIFTFT...............
NDS_061132	....
Samuk03G0051900	....
Samuk03G0054000	....
Sb000535g0022	FWK...SVGIYTDAEAKAIEQFIEIFKDKNFPP...GSSILFTQS.PHGSLTISFSKDGS
EVM20prediction20contig126.452	....
SECCE5Rv1G0364630	....
gene-G2W53_032366	....
SETIT_024113mg	....
SETIT_036958mg	....
SEVIR_9G033800v2	....
Sc06g0001890	....
Sc06g0001900	....
Solyc05g010310.3	....
Solyc05g010320.3	....
gene-LOC107019217	....
gene-LOC107020630	....
SORBI_3001G035600	....
gene-E2542_SST22123	....
gene-LOC110795717	....
gene-SI8410_06008823	....
gene-STAS_11501	FWTKGYTISIYNN....ILDK...............SDKIWFHYA..........SKDDD
EVM0031032	....
TkA04G183730	....
gene-LOC104825165	....
gene-HHK36_016491	....
gene-FRX31_032955	....
TCM_042994	...............PLPTSLFPNNYTATTFPPLHYKTHRFIPIS...............
Tel5E01G756500	....
gene-TAV2_LOCUS5378	....
gene-TAV2_LOCUS16152	....
EVM20prediction20LG01.1567	....
gene-TorRG33x02_127840	....
Tp57577_TGAC_v2_gene10384	....
gene-HS088_TW21G01722	....
TraesCS5A02G475600	....
TraesCS5B02G488900	....
TraesCS7B02G038200	....
Tubocapsicum_anomalum_10G026880	....
Tubocapsicum_anomalum_17G002220	....
Urofu.9G032800.v1.1	....
VducChr11G242040	....
vmacro07205_Vaccinium_macrocarpon_Stevens_v1	....
gene-HPP92_020856	....
tung.gene.scaffold2716.00003	....
gene-LOC114190515	....
gene-VitviT2T_019145	....FNLD...............
W.mirabilis.00703	LWE...AAGVYGEEKALAVEEFKGIF..................................
gene-JRO89_XSUnG0164900	....
Zm00001eb062510	....
gene-ZIOFF_031498	....
gene-ZIOFF_035070	....
Zla09G001950	....
gene-FEM48_Zijuj03G0079900	....
gene-FEM48_Zijuj03G0079800	....
gene-ZOSMA_1G00350	....
AT3G55120	....
Os03g60509	....

Medicago	....
Chr16.g28500	....
Chr1.g57423	....
Chr7.g32642	IPQVGNKVMENKLLGEAILESIIGKHGVSPAARQSLAERLSHLLTEKPELGPTNYHLYLN
QL08p057674	....
gene-CFP56_42925	....
gene-LOC108830048	....
gene-LOC108862091	....
gene-LOC115736770	....
gene-C3L33_05584	....
gene-LUZ62_010432	....
gene-LUZ62_034283	....
gene-LUZ62_049060	....
gene-LUZ62_054959	....
gene-LUZ62_079350	....
gene-LUZ62_083640	....
gene-LOC8264259	....
RchiOBHm_Chr1g0365111	....
RchiOBHm_Chr7g0221431	....
Rf070256510	....
Sspon.01G0031250-1A	....
Sspon.01G0031250-1T	....
Sspon.01G0031250-3C	....
ztl01450	....
Sapur.010G169300.v5.1	....
NDS_009150	....
NDS_061126	....
NDS_061132	....
Samuk03G0051900	....
Samuk03G0054000	....
Sb000535g0022	IPESGKFLIENKLLSEAILESMIGKHGVSPAAKKSLATRLSELLNAEGK...........
EVM20prediction20contig126.452	....
SECCE5Rv1G0364630	....
gene-G2W53_032366	....
SETIT_024113mg	....
SETIT_036958mg	....
SEVIR_9G033800v2	....
Sc06g0001890	....
Sc06g0001900	....
Solyc05g010310.3	....
Solyc05g010320.3	....
gene-LOC107019217	....
gene-LOC107020630	....
SORBI_3001G035600	....
gene-E2542_SST22123	....
gene-LOC110795717	....
gene-SI8410_06008823	....
gene-STAS_11501	LART..
EVM0031032	....
TkA04G183730	....
gene-LOC104825165	....
gene-HHK36_016491	....
gene-FRX31_032955	....
TCM_042994	.............................SPE............................
Tel5E01G756500	....
gene-TAV2_LOCUS5378	....
gene-TAV2_LOCUS16152	....
EVM20prediction20LG01.1567	....
gene-TorRG33x02_127840	....
Tp57577_TGAC_v2_gene10384	....
gene-HS088_TW21G01722	....
TraesCS5A02G475600	....
TraesCS5B02G488900	....
TraesCS7B02G038200	....
Tubocapsicum_anomalum_10G026880	....
Tubocapsicum_anomalum_17G002220	....
Urofu.9G032800.v1.1	....
VducChr11G242040	....
vmacro07205_Vaccinium_macrocarpon_Stevens_v1	....
gene-HPP92_020856	....
tung.gene.scaffold2716.00003	....
gene-LOC114190515	....
gene-VitviT2T_019145	....
W.mirabilis.00703	....
gene-JRO89_XSUnG0164900	....
Zm00001eb062510	....
gene-ZIOFF_031498	....
gene-ZIOFF_035070	....
Zla09G001950	....
gene-FEM48_Zijuj03G0079900	....
gene-FEM48_Zijuj03G0079800	....
gene-ZOSMA_1G00350	....
AT3G55120	....
Os03g60509	....

	1
**Medicago**	.......................M....................................
**Chr16.g28500**	....................M.......................................
**Chr1.g57423**	....................MA.P....................................
**Chr7.g32642**	PPFPTGNPISLSAQFINCQ.MA.P....................................
**QL08p057674**	....................MV.L....................................
**gene-CFP56_42925**	....................MV.L....................................
**gene-LOC108830048**	.............MSSSHC.PS.P....................................
**gene-LOC108862091**	.............MSSSDC.PS.P....................................
**gene-LOC115736770**	....................MT.PP...................................
**gene-C3L33_05584**	....................MS.SP...................................
**gene-LUZ62_010432**	....................EA......................................
**gene-LUZ62_034283**	....................VA......................................
**gene-LUZ62_049060**	....................VA......................................
**gene-LUZ62_054959**	....................EA......................................
**gene-LUZ62_079350**	....................VA......................................
**gene-LUZ62_083640**	....................VA......................................
**gene-LOC8264259**	....................MS......................................
**RchiOBHm_Chr1g0365111**	....................MA......................................
**RchiOBHm_Chr7g0221431**	....................MQ......................................
**Rf070256510**	....................MA......................................
**Sspon.01G0031250-1A**	....
**Sspon.01G0031250-1T**	....
**Sspon.01G0031250-3C**	....
**ztl01450**	....................MS.P....................................
**Sapur.010G169300.v5.1**	....................MS.T....................................
**NDS_009150**	....................MS.A....................................
**NDS_061126**	........FNSATYSDCEK.MS.A....................................
**NDS_061132**	....................MS.A....................................
**Samuk03G0051900**	....................MH.P....................................
**Samuk03G0054000**	....................MH.P....................................
**Sb000535g0022**	......SQAQEEQLPIRSN.MH.P....................................
**EVM20prediction20contig126.452**	....................MS.A....................................
**SECCE5Rv1G0364630**	....
**gene-G2W53_032366**	....................MA......................................
**SETIT_024113mg**	....................MA......................................
**SETIT_036958mg**	....................MK.ANH...PRA...L....SLVPILSQ...LPR......
**SEVIR_9G033800v2**	....
**Sc06g0001890**	....................MA.G....................................
**Sc06g0001900**	....................MR..............NGCC....................
**Solyc05g010310.3**	....................MA......................................
**Solyc05g010320.3**	....................MY......................................
**gene-LOC107019217**	....................MA......................................
**gene-LOC107020630**	....................MA......................................
**SORBI_3001G035600**	....
**gene-E2542_SST22123**	....................MA......................................
**gene-LOC110795717**	....................MA.S....................................
**gene-SI8410_06008823**	....................MV.TV...................................
**gene-STAS_11501**	......QSGSQPADLSATT.MS.A....................................
**EVM0031032**	....................MP.K....................................
**TkA04G183730**	....................MA.P....................................
**gene-LOC104825165**	....................MSSP....................................
**gene-HHK36_016491**	....................MA.L....................................
**gene-FRX31_032955**	....................MG.EL...................................
**TCM_042994**	......KKKKEKGLFNCRN.MS.T....................................
**Tel5E01G756500**	....
**gene-TAV2_LOCUS5378**	....................MP......................................
**gene-TAV2_LOCUS16152**	.............MSSSAC.PS.P....................................
**EVM20prediction20LG01.1567**	....................MS.S....................................
**gene-TorRG33x02_127840**	....................MA.P....................................
**Tp57577_TGAC_v2_gene10384**	....................MY.F....................................
**gene-HS088_TW21G01722**	....
**TraesCS5A02G475600**	....
**TraesCS5B02G488900**	....
**TraesCS7B02G038200**	....................ME......................................
**Tubocapsicum_anomalum_10G026880**	....................MA......................................
**Tubocapsicum_anomalum_17G002220**	....
**Urofu.9G032800.v1.1**	....
**VducChr11G242040**	....................MS.S....................................
**vmacro07205_Vaccinium_macrocarpon_Stevens_v1**	....................MS.L....................................
**gene-HPP92_020856**	....QSVVEGFRTQATADP.EA.V....................................
**tung.gene.scaffold2716.00003**	....................MS.P....................................
**gene-LOC114190515**	....................MS......................................
**gene-VitviT2T_019145**	.......CGLAHGEAISPK.MS.P....................................
**W.mirabilis.00703**	................SEKTMA.AGSSILFTVSPE.....GLLVAFAEGSSLAEISEVKE
**gene-JRO89_XSUnG0164900**	....................MN.P....................................
**Zm00001eb062510**	....
**gene-ZIOFF_031498**	....................ME.KQIK.................................
**gene-ZIOFF_035070**	....................ME.KQIK.................................
**Zla09G001950**	....
**gene-FEM48_Zijuj03G0079900**	....................MA.P....................................
**gene-FEM48_Zijuj03G0079800**	....................MA.P....................................
**gene-ZOSMA_1G00350**	....................MS.SN...................................
**AT3G55120**	...........SSSNACAS.PS.P....................................
**Os03g60509**	....

**Medicago**	....AA.S........IT......
**Chr16.g28500**	....
**Chr1.g57423**	....TP.S........LA......
**Chr7.g32642**	....PP.S........LA......
**QL08p057674**	....AA.S........LP......
**gene-CFP56_42925**	....AA.S........LP......
**gene-LOC108830048**	....LP.S........VT......
**gene-LOC108862091**	....LP.T........AP......
**gene-LOC115736770**	....PSA.A........VS......
**gene-C3L33_05584**	....LAP.S........VT......
**gene-LUZ62_010432**	....CSM.T........VT......
**gene-LUZ62_034283**	....LVP.K........PT......
**gene-LUZ62_049060**	....LVP.K........PT......
**gene-LUZ62_054959**	....CSM.T........VT......
**gene-LUZ62_079350**	....LVP.K........PT......
**gene-LUZ62_083640**	....LVP.K........PT......
**gene-LOC8264259**	....PL.S........AT......
**RchiOBHm_Chr1g0365111**	....Q.S........VT......
**RchiOBHm_Chr7g0221431**	....K...............
**Rf070256510**	....P.P........IT......
**Sspon.01G0031250-1A**	....M.A........VP......
**Sspon.01G0031250-1T**	....M.A........VP......
**Sspon.01G0031250-3C**	....M.A........VP......
**zt101450**	....LV.P........LS......
**Sapur.010G169300.v5.1**	....AV.P........LS......
**NDS_009150**	....TP.T........VA......
**NDS_061126**	....TP.S........VT......
**NDS_061132**	....TP.T........VT......
**Samuk03G0051900**	....SL.P........VA......
**Samuk03G0054000**	....SL.P........VA......
**Sb000535g0022**	....SP.S........IT......
**EVM20prediction20contig126.452**	....SP.S........VT......
**SECCE5Rv1G0364630**	....M.A........VS......
**gene-G2W53_032366**	....PP.S........LT......
**SETIT_024113mg**	....L........SS......
**SETIT_036958mg**	.........P.AP......YASISFICYPACVRSVPSWCATM.A........VS......
**SEVIR_9G033800v2**	....M.A........VS......
**Sc06g0001890**	....ASQ.A........VT......
**Sc06g0001900**	....VTGSNTGT
**Solyc05g010310.3**	....C........VT......
**Solyc05g010320.3**	....
**gene-LOC107019217**	....C........VT......
**gene-LOC107020630**	....V........VT......
**SORBI_3001G035600**	....M.A........VP......
**gene-E2542_SST22123**	....PA.S........VT......
**gene-LOC110795717**	....ST.P........VT......
**gene-SI8410_06008823**	....ELP.A........VS......
**gene-STAS_11501**	....LP.P........VT......
**EVM0031032**	....LP.S........VT......
**TkA04G183730**	....PP.S........TT......
**gene-LOC104825165**	....SP.S........VA......
**gene-HHK36_016491**	....AP.S........VS......
**gene-FRX31_032955**	....IES.M........FS......
**TCM_042994**	....SP.C........VA......
**Tel5E01G756500**	....M.A........VS......
**gene-TAV2_LOCUS5378**	....LP.S........VT......
**gene-TAV2_LOCUS16152**	....LP.S........VS......
**EVM20prediction20LG01.1567**	....PP.S........LT......
**gene-TorRG33x02_127840**	....APS.T........LT......
**Tp57577_TGAC_v2_gene10384**	....IL.H.........A......
**gene-HS088_TW21G01722**	....
**TraesCS5A02G475600**	....M.A........VS......
**TraesCS5B02G488900**	....M.A........VS......
**TraesCS7B02G038200**	.....................................DTTFPAG........IT......
**Tubocapsicum_anomalum_10G026880**	....S........VA......
**Tubocapsicum_anomalum_17G002220**	....
**Urofu.9G032800.v1.1**	....M.A........VS......
**VducChr11G242040**	....QP.A........VT......
**vmacro07205_Vaccinium_macrocarpon_Stevens_v1**	....QP.A........VT......
**gene-HPP92_020856**	....MP.V........VR......
**tung.gene.scaffold2716.00003**	....AT.S........VT......
**gene-LOC114190515**	....LP.S........VT......
**gene-VitviT2T_019145**	....VP.S........VT......
**W.mirabilis.00703**	MKNRAMGEALLATMIGENAVSPQTKASIADRFLTL....YLP.AVQQAKSEDLP......
**gene-JRO89_XSUnG0164900**	....SP.L........VT......
**Zm00001eb062510**	....M.A........VP......
**gene-ZIOFF_031498**	....VAT.S........LP......
**gene-ZIOFF_035070**	....VAT.S........LP......
**Zla09G001950**	....M.D........VT......
**gene-FEM48_Zijuj03G0079900**	....TL.S........VT......
**gene-FEM48_Zijuj03G0079800**	....AL.S........IT......
**gene-ZOSMA_1G00350**	....DTS.L........LD......
**AT3G55120**	....FP.A........VT......
**Os03g60509**	....MA.A........VS......

```
                                                                10        20        30        40        50
Medicago                                            ..AITVENLEYP..A..VV.TSPVTG........KS.YFLGGAGERGLTIEGNFIKFTAI
Chr16.g28500                                        .....................................LFM....................
Chr1.g57423                                         ..GLQIETTAFP..P..SV.KPPGSS........NT.LFLGGAGVRGLEIQGNFVKFTAI
Chr7.g32642                                         ..GLQVEATAFP..P..SV.KPPGSS........NT.LFLGGAGVRGLEIQGNFVKFTAI
QL08p057674                                         ..GVQVEHVAFP..S..TA.KPPGST........NT.LFLGGAGVRGLEIQGKFVKFTAI
gene-CFP56_42925                                    ..GVQVEHVAFP..S..TA.KSPGST........NT.LFLGGAGVRGLEIQGKFVKFTAI
gene-LOC108830048                                   ..KLQVDCVTFP..P..SV.ISPASS........TP.LFLGGAGVRGLDIHGKFVIFTVI
gene-LOC108862091                                   ..KLQVDSVTFP..P..SV.ISPASS........NP.LFLGGAGVRGLEIQGKFVIFTVI
gene-LOC115736770                                   ..EVQVESVKFP..P..SV.KPPGSS........KT.FFLGGAGERGLEIQGKFIKFTAI
gene-C3L33_05584                                    ..EIIIEGHVFP..P..AV.KPPGTT........KS.FFLGGAGERGLEIQGKFIKFTAI
gene-LUZ62_010432                                   ..EIELEGIMFP..S..AL.TPPRSG........KT.LFLGGAGVRGLDINGEFKVFTVT
gene-LUZ62_034283                                   ..PLDVDGVAFP..A..FV.TPPDSS........KPPLFLAGAGVRGLDIAGRFVKFTAI
gene-LUZ62_049060                                   ..PLDVDGVAFP..A..FV.TPPDSS........KPPLFLAGAGVRGLDIAGRFVKFTAI
gene-LUZ62_054959                                   ..EIELEGIMFP..S..AL.TPPFSG........KT.LFLGGAGVRGLDINGEFKVFTVT
gene-LUZ62_079350                                   ..PLDVDGVAFP..A..FV.TPPDSS........KPPLFLAGAGVRGLDIAGRFVKFTAI
gene-LUZ62_083640                                   ..PLDVDGVAFP..A..FV.TPPDSS........KPPLFLAGAGVRGLDIAGRFVKFTAI
gene-LOC8264259                                     ..HIVIEAVSFP..P..AV.KPPASD........KT.LFLAGAGVRGLEIEGKFVKFTAI
RchiOBHm_Chr1g0365111                               ..GIQVEATTFP..P..AV.KPPGSA........NT.LFLAGAGARGLEIQGNFVKFTAI
RchiOBHm_Chr7g0221431                               ...........PK.P.............................LKSL............
Rf070256510                                         ..GIQIEATSFP..P..TV.KPPGSG........NT.LFLGGAGVRGLEIQGNFVKFTAI
Sspon.01G0031250-1A                                 ..EVVVEGVVFP..S..VA.RPPGSA........GS.HFLGGAGVRGLEIGGNFVKFTAI
Sspon.01G0031250-1T                                 ..EVVVEGVVFP..P..VA.RPPGSA........VS.HFLGGAGLRGLEIGGNFIKFTAI
Sspon.01G0031250-3C                                 ..EVVVEGVVFP..P..VA.RPPGSA........VS.HFLGGAGLRGLEIGGNFIKFTAI
ztl01450                                            ..EIKIENVTFP..A..AV.KPPASN........NT.LFLGGAGVRGLEIEGKFIKFTAI
Sapur.010G169300.v5.1                               ..KIKVENVTFP..A..AV.KPPASN........KT.LFLGGAGVRGLEIEGRFIKFTAI
NDS_009150                                          ..QLQVESVVFP..P..TV.KPPGSD........KT.LFLGGAGVRGMEIQGNFVKFTAI
NDS_061126                                          ..QLQVESVVFP..P..TA.KPPGSD........KT.LFLGSAGVRGMEIQGNFVKFTAI
NDS_061132                                          ..QLQVESVVFP..P..TA.KPPGSD........KT.LFLGGAGVRGMEIQGNFVKFTAI
Samuk03G0051900                                     ..EVQVENITFS..P..AV.KPPGSA........KL.HFLGGAGDRGLEIQGKFIKFTAI
Samuk03G0054000                                     ..EVQVENITFS..Q..AV.KPPGSA........KL.HFLGGAGDRGLEIQGKFIKFTAI
Sb000535g0022                                       ..KIQIENVTFS..P..TV.RPPGST........KT.LFLGGAGERGLEIQGKFVKFTAI
EVM20prediction20contig126.452                      ..KVLVESIEFS..P..AA.KPPGSS........NT.LFLGGAGVRGMEIQGNFVKFTAI
SECCE5Rv1G0364630                                   ..ELEVDGVVFP..P..LA.RPPGTE........HS.HFLAGAGVRGMDIGGNFIKFTAI
gene-G2W53_032366                                   ..AVKVEDVEFP..P..AV.KAPGSD........KS.FFLAGGGVRGLQIHDKFVKFTAI
SETIT_024113mg                                      ..EVTVEGIVFP..P..VA.RPPGSA........LT.HFLAGGGVRGMEAEGNFVKIAAI
SETIT_036958mg                                      ..ELAVDGVVFP..P..VA.RPPGSA........RP.HFLAGAGVRGLEIGGNFIKFTAI
SEVIR_9G033800v2                                    ..ELAVDGVVFP..P..VA.RPPGSA........RP.HFLAGAGVRGLEIGGNFIKFTAI
Sc06g0001890                                        ..LVQVEDYVFP..P..RLLHPLGSL........KP.FLLAGAGERGLEIEGRFIKFTAI
Sc06g0001900                                        GGRLRV.....P.FQ..AC.EPLGSF........KS.LFLAGAGVRRLEIEGRFKKFTAI
Solyc05g010310.3                                    ..KLQVENNVFP..SK.VV.KPPSSN........NT.FFLGGAGHRGLEVEGKFVKFSVI
Solyc05g010320.3                                    ......NEFNF..............D...........MCL...GNRGLEIEGKFVKFTAI
gene-LOC107019217                                   ..KLQVENIVFP..SK.VV.KPPSSN........NT.FFLGGAGHRGLEVEGKFVKFSVI
gene-LOC107020630                                   ..KLQVENHVFP..PT.MV.NPLGSS........NM.FLLAGAGSRGLEIEGKFVKFTAI
SORBI_3001G035600                                   ..EVVVEGVVFP..P..VA.RPPGSA........GS.HFLGGAGVRGIEIGGNFIKFTAI
gene-E2542_SST22123                                 ..SVDVENVTFP..L..TV.KAPSSA........NT.FLLGGAGVRGLQIQDKFVKFTAI
gene-LOC110795717                                   ..QVNVEDYAFP..A..TV.KAPGSE........KS.FLLAGAGVRGLEIQGNFVKFTAI
gene-SI8410_06008823                                ..ELDIEGFVFP..P..VVAKPPGSS........KS.LFLGGAGVRSLEIQGRVIKFTSI
gene-STAS_11501                                     ..EVRVESIVFP..P..AV.KPPGSA........KT.LFLGGAGVRGMEIGGAFVKFTAI
EVM0031032                                          ..QVEVDTCVFP..P..AV.QPPGSD........NT.FFLGGAGVRGLEIEGKFIKFTAI
TkA04G183730                                        ..SLQVESIVFP..P..SV.KPPGAT........NT.LFLAGAGVRGMEIEGNFVKFTGI
gene-LOC104825165                                   ..EIQVDAVTFP..P..AV.KPPASS........KT.LFLGGAGVRGLDIEGKFVKFTAI
gene-HHK36_016491                                   ..ELKVESFCFP..P..TV.KPPGST........KT.LFLGGAGARGLEIQGKFIKFTAI
gene-FRX31_032955                                   ..ELKVEDYTFC..P..TLSTVPGSA........NS.FCLGGAGVRGMQIQDKFIKFTAI
TCM_042994                                          ..GIQVENVTFP..P..NV.KPPGST........KT.LFLGGAGERGLEIQGKFVKFTAI
Tel5E01G756500                                      ..ELEVDGVVFP..P..LA.RPPGSE........HA.HFLAGAGVRGMEIGGSFIKFTAI
gene-TAV2_LOCUS5378                                 ..PLHVDSFTFP..P..DI.TSPASS........KK.LFLGGAGVRGFEIEGKFEIVTVI
gene-TAV2_LOCUS16152                                ..KLHVDSVTFP..P..SV.NSPASS........NP.LFLGGAGVRGLDIQGKFVIFTVI
EVM20prediction20LG01.1567                          ..KVQVENCTFP..P..AV.KPPGST........NT.FFLGGAGVRGIEKPGIFVKVTAI
gene-TorRG33x02_127840                              ..GVQIETATFP..P..AV.TPPGSA........KA.LFLGGAGARGIEIQGNFVKFTAI
Tp57577_TGAC_v2_gene10384                           ..RIPKTTYSLP..PSNHR.DPPTTSXXXXXXXXXX.XXXXXXGERGLQIQDKFVKFTAI
gene-HS088_TW21G01722                               ............................................................
TraesCS5A02G475600                                  ..ELEVDGVVFP..P..LA.RPPGSE........HA.HFLAGAGVRGMEIGGNFIKFTAI
TraesCS5B02G488900                                  ..ELEVDGVVFP..P..LA.RPPGSE........HA.HFLAGAGVRGMEIGGKFIKFTAI
TraesCS7B02G038200                                  ..DMEVGRVVFG..A..SA.TPPHSS........KI.LFLGGAGERIGPRHGTLKLVTVI
Tubocapsicum_anomalum_10G026880                     ..KLQVENHVFP..PT.VV.KPPGSN........NI.LFLAGAGNRGLEIEGKFVKFTVI
Tubocapsicum_anomalum_17G002220                     .......................................MSVAGNRGLEIEGKFVKFSVI
Urofu.9G032800.v1.1                                 ..EVAVDGVVFP..PV.VA.RPPGSG........RP.HFLAGAGVRGMEIGGNFIKFTAI
VducChr11G242040                                    ..EIKIDSQVFP..P..SV.KPPGTT........NS.FFLGGAGERGLEIEGKFIKFTAI
vmacro07205_Vaccinium_macrocarpon_Stevens_v1        ..EIKIESQVFP..P..SV.KPPGTT........NS.FFLGGAGERGLEIEGKFIKFTAI
gene-HPP92_020856                                   ..ELLVEGVAFP..A..EA.IPPGSS........KR.LFLGGAGARGIEIGGSFKVVTAI
tung.gene.scaffold2716.00003                        ..QIEIDTVTFS..P..AA.KPPASN........KT.LFLGGAGVRGLEIQGKFVKFTAI
gene-LOC114190515                                   ..ALDVDNVTFP..P..TV.IPPSST........AA.FFLAGAGVRGLQIQDNFVKFTAI
gene-VitviT2T_019145                                ..AVQVENVLFP..P..SV.KPPGST........ND.LFLGGAGVRGLEIQGKFVKFTAI
W.mirabilis.00703                                   ..AMEIEGNFFP..S..TV.TSPGAT........QD.LILAGAGVRGLTIEGNFIPVTTI
gene-JRO89_XSUnG0164900                             ..GVQVENVTFS..P..TV.KPPGST........NL.HFLGGAGERGLNIQGKFILFTAI
Zm00001eb062510                                     ..EVVVDGVVFP..P..VA.RPPGSA........GS.HFLGGAGVRGLEIGGNFIKFTAI
gene-ZIOFF_031498                                   ..KLQVEGVDFP..S..LA.TPPGSS........KV.FFLAGAGERGLEIAGTFVTFTAI
gene-ZIOFF_035070                                   ..KLQVEGVDFP..S..LA.TPPGSS........KV.FFLAGAGERGLEIAGTFVTFTAI
Zla09G001950                                        ..KVEVDGVVFP..P..VV.RPPGSS........LT.HFLAGAGVRGVEIGGSFIKFTAI
gene-FEM48_Zijuj03G0079900                          ..GINVDNVEFP..P..SA.KPPGSK........NT.LFLGGAGVRGLNIEGKFVKFTAI
gene-FEM48_Zijuj03G0079800                          ..GINVDSVKFP..A..TA.KPPGST........NT.LFLGGAGVRGLNIEGNFVKFTAI
gene-ZOSMA_1G00350                                  ..DIQIEGISFP..F..SV.KPPGTS........NI.FFLAGAGVRGLEINGNFIKFTSI
AT3G55120                                           ..KLHVDSVTFV..P..SV.KSPASS........NP.LFLGGAGVRGLDIQGKFVIFTVI
Os03g60509                                          ..EVEVDGVVFP..P..VA.RPPGSG........HA.HFLAGAGVRGVEIAGNFIKFTAI
```

```
                                              60        70        80
                                               .         .         .
Medicago                              GVYLE.D.IA.VASLAAKWK....GKSSEELLETLDFYRDIIS.................
Chr16.g28500                          ...........IAIMV.AYK..................SSFVT.................
Chr1.g57423                           GVYLE.D.NA.VPQLAVKWK....GKTAEELTESVEFFRDIVT.................
Chr7.g32642                           GVYLE.D.NA.VPLLAVKWK....GKTAKELSESVEFFRDIVT.................
QL08p057674                           GVYLE.N.NA.VPSLAVKWR....GKSAEELTESVEFFRDIVT.................
gene-CFP56_42925                      GVYLE.D.NA.VPSLAVKWG....GKSAEELTESVEFFRDIVT.................
gene-LOC108830048                     GVYLA.P.VA.VPSLSVKWK....GKTTEELTESVPFFREIVT.................
gene-LOC108862091                     GVYLD.P.VS.VPSLSVKWE....GKTTEELTESVPFFREIVT.................
gene-LOC115736770                     GVYLE.D.AA.LPSLAAKWS....GKSAEELADSVEFFRDIVT.................
gene-C3L33_05584                      GVYLE.V.SA.VASLAVKWK....GKSAEELTESVEFFRDIVS.................
gene-LUZ62_010432                     GVYLE.S.EA.VNFLTKRWK....GKPVEQLSSSNQFYRDIVT.................
gene-LUZ62_034283                     AIYLQ.D.SA.LDLLACKWTPH..TNTPDQLASATDFFADIIN.................
gene-LUZ62_049060                     AIYLQ.D.SA.LNLLACKWTPH..TNTPDQLASATDFFADIIN.................
gene-LUZ62_054959                     GVYLE.S.EA.VNFLTKRWK....GKPVEQLSSSNQFYRDIVT.................
gene-LUZ62_079350                     AIYLQ.D.SA.LDLLACKWTPH..TNTPDQLASATDFFADIIN.................
gene-LUZ62_083640                     AIYLQ.D.SA.LDFLACKWSPHTNANTPDQLASATDFFADIIN.................
gene-LOC8264259                       GVYLE.D.DA.VPLLAVKWK....GKTAQELTDSVEFFRDIVT.................
RchiOBHm_Chr1g0365111                 GVYLE.D.KA.VPALAVKWK....GKTAEELTESVEFFREIVT.................
RchiOBHm_Chr7g0221431                 ............................................................
Rf070256510                           GVYLE.D.KA.VPELAIKWK....GKTAEELTESVQFFREIVT.................
Sspon.01G0031250-1A                   GVYLE.D.AA.VSALANKWA....GKTADELASDAAFFRDVVK.................
Sspon.01G0031250-1T                   SVYLE.D.AA.VSALAKKCA....GKTADELASDAAFFRDVVT.................
Sspon.01G0031250-3C                   GVYLE.D.AA.VSALAKKCA....GKTADELASDAAFFRDVVT.................
zt101450                              GVYLE.D.SS.LQSLAAKWK....GKSAKELTDSVEFYGDIVR.................
Sapur.010G169300.v5.1                 GVYLE.D.SS.LQSLAAKWK....GKSAKELTDSVEFYGDIVR.................
NDS_009150                            AVYME.G.TA.IPALAPKWS....AKTVDEMADSADFIREIIA.................
NDS_061126                            AVYME.G.TA.IPALAPKWS....AKTVDELADSADFIREIIA.................
NDS_061132                            AVYME.G.TA.IPALTPKWS....AKTVDELADSADFIREIIA.................
Samuk03G0051900                       GVYLE.N.DA.VSSLAVKWK....GKTAEELTESVEFFRDIVT.................
Samuk03G0054000                       GVYLE.N.DA.VSSLAVKWK....GKTAEELTESVEFFRDIVT.................
Sb000535g0022                         GVYVE.D.DA.VTSLAGKWK....GKSTEELAESVEFFRDIVT.................
EVM20prediction20contig126.452        GVYLE.D.SA.VPSLAVNWK....GKTAEELTESDDFFREIVS.................
SECCE5Rv1G0364630                     GVYLQ.ADAA.VSALAAKWA....GKPAADLAADAAFFRDVVT.................
gene-G2W53_032366                     GVYFE.D.NA.VPLLAAKWK....GKSAQELTDSVEFFREIVT.................
SETIT_024113mg                        GVYLE.D.AA.VAALAGKWA....GKSAGELASDPAFFRDVYT.................
SETIT_036958mg                        GVYLE.EDAA.VSALAKKWA....GKSADELASDVAFFRDVVT.................
SEVIR_9G033800v2                      GVYLE.EDAA.VSALAKKWA....GKSADELASDVTFFRDVVT.................
Sc06g0001890                          GVYVE.EDAA.VVVLAAKWK....GKTAAELTDSVEFFRDVIS.................
Sc06g0001900                          GVYIE.E.EVTVAVLAAKWK....GKTAAELVVPVEFFRDI...................
Solyc05g010310.3                      GVYLE.E.NV.VPFLAVKWK....GRSSEELTYSLEFFRDIVT.................
Solyc05g010320.3                      GVYLE.E.SA.IPFLADKWK....GKSSEELEHSVEFFRDIVT.................
gene-LOC107019217                     GVYLE.E.NV.VPFLAVKWK....GRSSEELTYSLEFFRDIVT.................
gene-LOC107020630                     GVYLE.E.SA.IPFLADKWK....GKSSEELEHSVEFFRDIVT.................
SORBI_3001G035600                     GVYLE.D.AA.VSALAKKWA....GKTADELASDAAFFRDVVT.................
gene-E2542_SST22123                   GVYLQ.D.DA.VKFLAAKWN....GKSAPELTESDEFFRDIVT.................
gene-LOC110795717                     GVYLE.E.SG.ISALAAKWK....GKTPAELVESVEFFRDIVT.................
gene-SI8410_06008823                  GVYVE.A.EA.VPSLAVKWN....EKTAEELADSVDFFQDIVT.................
gene-STAS_11501                       GVYLE.D.NA.VQSLAVKWK....GKSAKELTESVDFFTDIFT.................
EVM0031032                            GVYLE.D.AA.VPSLAVNWK....GKTAKELTDSDDFFTDIVT.................
TkA04G183730                          GVYLE.D.KA.IPSLAVNWK....GKTAAELTDSVEFFRDIVT.................
gene-LOC104825165                     GVYLE.D.NA.LPLLSVKWS....GRTAEELTDSVAFFRELVT.................
gene-HHK36_016491                     GVYLE.D.IA.VPSLAVKWK....GKTADELTDSVEFFRDIVS.................
gene-FRX31_032955                     GVYLE.EQKA.ISSLSMKWK....GKSAEELTDSIEFFRDIVT.................
TCM_042994                            GVYLE.D.IA.VESLAVKWK....GKSAEVLTESVEFFRDIVT.................
Tel5E01G756500                        GVYLQ.ADDA.VSALAAKWA....GKPAADLASDAAFFRDVVT.................
gene-TAV2_LOCUS5378                   GVYLE.A.IA.VPSLSVKWK....GKNAKELTDSISFFR.....................
gene-TAV2_LOCUS16152                  GVYLD.A.VA.VPSLSVKWE....GKTTEELTESVPFFREIVT.................
EVM20prediction20LG01.1567            GVYLE.E.KA.VNSLAAKWK....GKTAEELTESVEFFRDIVT.................
gene-TorRG33x02_127840                GVYLE.D.NA.VTWLAGKWS....NKTAEELTESVEFFRDIVT.................
Tp57577_TGAC_v2_gene10384             AIYLQ.A.TA.VPYLADKWK....GKSPQELTETVPFFRDIVT.................
gene-HS088_TW21G01722                 ............................................................
TraesCS5A02G475600                    GVYLQ.ADAA.VPALAAKWA....GKPAADLASDAAFFRDVVT.................
TraesCS5B02G488900                    GVYLQ.ADAA.VPALAAKWA....AKPAADLASDAAFFRDVVT.................
TraesCS7B02G038200                    GVYLE.Q.SA.VPFLTLRWQ....GKPAEELARSADFFRDIVA.................
Tubocapsicum_anomalum_10G026880       GVYLE.E.SA.IPFLAAKWK....GKSSEELTHSVEFFRDVVT.................
Tubocapsicum_anomalum_17G002220       GVYLE.E.SA.IPFLVDKWK....GESSEELTQSVEFFRDIVI.................
Urofu.9G032800.v1.1                   GVYLE.EGAA.VPALAKKWA....GKSADELASDVAFFRDVVT.................
VducChr11G242040                      GVYLE.V.SA.VESLAVKWK....GKSAEELTESVEFFRDIVSEWEDGDSDEGEGDVEDE
vmacro07205_Vaccinium_macrocarpon_Stevens_v1 GVFLE.V.SA.VESLAVKWK....GKSAEELTESVEFFRDIVSEWEDGDSDEGEGDEEDE
gene-HPP92_020856                     GVYLE.K.TA.VQALGGQWK....GKLPAELAGSADFFHQIVT.................
tung.gene.scaffold2716.00003          GVYLE.D.EA.VPLLAVKWK....GKSAKELTDSVEFFRDIVT.................
gene-LOC114190515                     GIYLQ.P.NA.VPLLSVKWN....AKSAHELTESVEFFRDIVT.................
gene-VitviT2T_019145                  GVYLE.S.SA.VPTLAVKWK....GKTVEELADSVDFFRDVVT.................
W.mirabilis.00703                     GVYMDRE.IV.SNQLMPKWK....GKSAEELNEDENFILEIIT.................
gene-JRO89_XSUnG0164900               AVYLE.D.DA.VASLAGKWK....GKSAEELAESVEFFRDIVT.................
Zm00001eb062510                       GVYLE.D.AA.VPALAKKWG....GKTADELASDAAFFRDVVT.................
gene-ZIOFF_031498                     GIYLE.E.EA.VKALAARWT....GRSADELAASLDFFRDIFA.................
gene-ZIOFF_035070                     GIYLE.E.EA.VKALAARWK....GRSADELAASLDFFRDIFA.................
Zla09G001950                          GVYLE.D.AA.VPALAKKWA....GKTADELAADATFFRDVVT.................
gene-FEM48_Zijuj03G0079900            GVYLE.D.NA.IPWLTAKWK....GKNSEELTESDHFFRDIVT.................
gene-FEM48_Zijuj03G0079800            GVYLE.E.SA.VPWLAAKWK....GKTLEELLESDEFSRDIVT.................
gene-ZOSMA_1G00350                    GVYLE.E.SASLQYLSPKWK....LLSASELSKSVEFFRDVVT.................
AT3G55120                             GVYLE.G.NA.VPSLSVKWK....GKTTEELTESIPFFREIVT.................
Os03g60509                            GVYLE.EGAA.VPALAKKWA....GKSADELAADAAFFRDVVT.................
```

```
                                                               90        100       110       120
Medicago                                         ..............GPFEKLIRGSKI..RELSGPEYSRKVMENCVAHLKSVGTYGDAE..
Chr16.g28500                                     ................................FPLD..........................F.
Chr1.g57423                                      ..............GPFEKFIQVTTI..LPLTGQQYAEKVSENCVAFWKSVGIYTDAE..
Chr7.g32642                                      ..............GPFEKFIQVTTI..LPLTGQQYSEKVSENCVAFWKSVGIYTDAE..
QL08p057674                                      ..............GPFEKFTQVTTI..LPLTGQQYSEKVTENCVAFWKSVGLYTEAE..
gene-CFP56_42925                                 ..............GPFEKFTQVTTI..LPLTGQQYSEKVTENCVAFWKSVGLYTEAE..
gene-LOC108830048                                ..............GSFEKFIKVTMK..LPLTGHQYSEKVTENCVAIWKSLGIYTDAE..
gene-LOC108862091                                ..............GSFEKFIKVTMK..LPLTGQQYSEKVTENCVAIWKSLGIYTDSE..
gene-LOC115736770                                ..............GPFEKFSRVTMI..LPLTGAQYAEKVTENCVKYWQSVGSYTDAE..
gene-C3L33_05584                                 ..............ASHTDLE...............EYT.......................
gene-LUZ62_010432                                ..............GPFDKLSQVTLV..AHLSGSQFVQKVVENCRTFMKKENLYSQKE..
gene-LUZ62_034283                                ..............GPFEKFIRVTMI..LPLTGEQYSEKVSENCVAHWKSIGIFTEAE..
gene-LUZ62_049060                                ..............GPFEKFIRVTMI..LPLTGEQYSEKVSENCVAHWKSIGIFTEAE..
gene-LUZ62_054959                                ..............APFDKLSQVTLV..AHLSGSQFVQKVAENCRTFMKKENLYSQKE..
gene-LUZ62_079350                                ..............GPFEKFIRVTMI..LPLTGEQYSEKVSENCVAHWKSIGIFTEAE..
gene-LUZ62_083640                                ..............GPFEKFIRVTMI..LPLTGEQYSEKVSENCVAHWKSIGIFTEAE..
gene-LOC8264259                                  ..............GPYEKFIRVTMI..LPLSGQQYSEKVAENCVAIWKSFGIYTDAE..
RchiOBHm_Chr1g0365111                            ..............GPFEKFTQVTMI..LPLTGQQYSEKVSENCVAIWKKFGIYTDAE..
RchiOBHm_Chr7g0221431                            ............................................................
Rf070256510                                      ..............GPFEKFTQVTMI..LPLTGQQYSEKVSENCVAIWKKFGIYTDAE..
Sspon.01G0031250-1A                              ..............GDFEKFTRVTMI..RPLTGQQYAEKVTENCVAFWKAVGLYTDAE..
Sspon.01G0031250-1T                              ..............GDFEKFTRVTMI..LPLTGEQYAEKVTENCVAFWKAVGLYTDAE..
Sspon.01G0031250-3C                              ..............GDFEKFTRVTMI..LPLTGEQYAEKLTENCVAFWKAVGLYTDAE..
zt101450                                         ..............GPFEKFMRVTMI..LPLTGLQYSEKVAENCVRIWKSLGIYTDAE..
Sapur.010G169300.v5.1                            ..............GPFEKFMRVTMI..LPLTGLQYSEKVAENCVRIWKSLGIFTDAE..
NDS_009150                                       ..............GPFEKLTKVTMI..LPLTGQQYSEKVAENCTAYWKAVGKYTDAE..
NDS_061126                                       ..............GPFEKLTKVTMI..LPLTGQQYSEKVAENCTAYWKAVGKYTDAE..
NDS_061132                                       ..............GPFEKLTKVTMI..LPLTGQQYSEKVAENCTAYWKAVGKYTDAE..
Samuk03G0051900                                  ..............GPFEKFMKVTMI..LPLTGQQYSEKVTENCIAFWKSIGIYTDAE..
Samuk03G0054000                                  ..............GPFEKFMKVTMI..VPLTGQQYSEKVTENCIAFWKSIGIYTDAE..
Sb000535g0022                                    ..............GPFEKFMQVTMI..LPLTGQQYSEKVSENCVAFWKSVGIYTDAE..
EVM20prediction20contig126.452                   ..............GPFEKFTKVTMI..LPLTGKQYSEKVAENCVAYWKAVGKYTDAE..
SECCE5Rv1G0364630                                ..............GEFEKFTRVTMI..LPLTGEQYSDKVTENCVAYWKATGVYTDAE..
gene-G2W53_032366                                ..............GGFEKFIQVTMI..LPLTGQQYAEKVSENCVHIWKSLGIYSDEE..
SETIT_024113mg                                   ..............GEFEKFTRVTFIWPKAVAAEEFAGKVMESRVAYLEAAGAYTDAE..
SETIT_036958mg                                   ..............GDFEKFTRVTMI..LPLTGEQYSDKVTENCVAYWKATGVYTDAE..
SEVIR_9G033800v2                                 ..............GDFEKFTRVTMI..LPLTGEQYSDKVTENCVAYWKATGVYTDAE..
Sc06g0001890                                     ..............GPFEKVTQVTMI..LPLTGQQYSEKVTENCVAYWKAVGIYTDEE..
Sc06g0001900                                     ............................................................
Solyc05g010310.3                                 ..............GPFEKFMRVTLL..LPLTGKQFSEKVAGNCVAIMKAMGNYSDAE..
Solyc05g010320.3                                 ..............GPFEKFTRVTMI..LPLTGKQYSEKVAENCVAYWKAIGTYSDAE..
gene-LOC107019217                                ..............GPFEKFMRVTLL..LPLTGKQFSEKVAGNCVAIMKATGNYSDAE..
gene-LOC107020630                                ..............GPFEKFTRVTMI..LPLTGKQYSEKVAENCVAYWKAIGTYSDAE..
SORBI_3001G035600                                ..............GDFEKFTRVTML..LPLTGEQYAGKVTENCVAFWKAVGLYTDAE..
gene-E2542_SST22123                              ..............GPFEKFMKVTMI..LPLTGQQYSEKVSENCVTIWKSLGIYTDAE..
gene-LOC110795717                                ..............GPFEKFTQVTMI..LPLTGQQYSEKVTENCVAFWKAIGIYTDDE..
gene-SI8410_06008823                             ..............GAFEKFIRVTMI..LPLTGQQYSEKVAENCVAYWKAVGIYTDAE..
gene-STAS_11501                                  ..............GPFEKFTRVTTI..LPLTGQQYSEKVAENCVAYLKSIGKYTDAE..
EVM0031032                                       ..............GSFGKFIKVTMI..LPLTGQQYSQKVAENCVAHWKSAGKYGDAE..
TkA04G183730                                     ..............GPFEKLTHITMI..LPLTGKQYSEKVSEMCVGVWKAHGTYTDAD..
gene-LOC104825165                                ..............GPFEKFVRVTMI..LPLTGVQYSEKVTENCVRIWKSLGIYTESE..
gene-HHK36_016491                                ..............GPFEKFTQVTTI..LPLTGHQYSEKVTENCVAYWKAVGIYTEAE..
gene-FRX31_032955                                ..............GPFEKFVMVTMI..LPLTGQQYSEKVAENCVAAWKAMGIYTEAE..
TCM_042994                                       ..............GAFEKFIRVTTI..LPLTGQQYSEKVSENCVAIWKSLGLYTDAE..
Tel5E01G756500                                   ..............GEFEKFTRVTMI..LPLTGAQYSDKVTENCVAYWKATGVYTDAE..
gene-TAV2_LOCUS5378                              ...............VFEKFTRVTMK..VKLSGTQYSEKVAEYCEEILKSSGKYTQSE..
gene-TAV2_LOCUS16152                             ..............GAFEKFIKVTMK..LPLTGQQYSEKVTENCVAIWKSLGIYTESE..
EVM20prediction20LG01.1567                       ..............GPFEKFTQVTMI..LPLTGQQYSEKVSENCVAIWKAVGIYTDAE..
gene-TorRG33x02_127840                           ..............GPFEKFTRITMI..LPLTGQQYSEKVSENCVAIWKSLGIYSDAE..
Tp57577_TGAC_v2_gene10384                        ..............GPFEKFMQVTMI..LPLTGQQYSEKVSENCVAIWKSLGIYTDEE..
gene-HS088_TW21G01722                            ........................ML..LPLTGQQYSEKVSENCVAFWKSIGIYSDAE..
TraesCS5A02G475600                               ..............GEFEKFTRVTMI..LPLTGAQYSDKVTENCVAYWKANGVYTDAE..
TraesCS5B02G488900                               ..............GEFEKFTRVTMI..LPLTGAQYSDKVTENCVAYWKATGVYTDAE..
TraesCS7B02G038200                               ..............GPFDKFTQVTLV..DTLTGQEYSDKVTENCVNIWKSSEHYSVDE.G
Tubocapsicum_anomalum_10G026880                  ..............GPFEKFTRVTMI..LSLTGKQYSEKVAENCVAYWKAIGTYSDAE..
Tubocapsicum_anomalum_17G002220                  ..............GPFEKFTRVTMI..LHLTGKQYSEKVAENCVAYWKARGTYSDAE..
Urofu.9G032800.v1.1                              ..............GDFEKFTRVTMI..LPLTGEQYSDKVSENCVAYWKATGVYTDAE..
VducChr11G242040                                 DGYKSIDDKEDEAIGPFEKFTQVTMI..LPLTGKQYSEKVAENCVAYWKAVGTYTDAE..
vmacro07205_Vaccinium_macrocarpon_Stevens_v1     DDYKSIDNKEDEAIGPFEKFTQVTTI..LPLTGKQYSEKVAENCVAYWKAVGTYTDAE..
gene-HPP92_020856                                ..............GRFEKLTRVSMI..LPLTGKQYSEKVASNCVAAWKAAEAYTNEE..
tung.gene.scaffold2716.00003                     ..............GPFEKFIRVTMI..LPLTGQQYSEKVTENCVAFWKSVGIYTEAE..
gene-LOC114190515                                ..............GPFEKFMQVTMI..LPLTGQQYSEKVAENCVAIWKSLGIYTDAE..
gene-VitviT2T_019145                             ..............GPFEKFTKVTTI..LPLTGRQYSDKVSENCVAFWKSVGIYTDAE..
W.mirabilis.00703                                ..............SSFPKMAQVSLL..RSLPGTAFCAKVADNCRSVLEKAGRLGESE..
gene-JRO89_XSUnG0164900                          ..............GPFEKFMQVTML..LPLTGPQYSEKVTENCIAFWKSAGIFTDAE..
Zm00001eb062510                                  ..............GDFEKFTRVTMI..LPLTGEQYAEKVTENCVAFWKAAGLYTDAE..
gene-ZIOFF_031498                                ..............GAFDKFTRITMV..KPLTGQQYSDKVAENCLAQWQAAGTLTDAE..
gene-ZIOFF_035070                                ..............GAFDKFTSITML..KPLTGQQYSDKVTENCLAQWQAAGTLTDAE..
Zla09G001950                                     ..............GDFEKFTRVTLI..RLLTGEQYSDKVTENCVAYWKATGVSTDAE..
gene-FEM48_Zijuj03G0079900                       ..............GPFEKLTRVAFI..STLTGQQYSEKVVENSTAILKSWGVYNDSE..
gene-FEM48_Zijuj03G0079800                       ..............GPFEKFTRVTMI..LPLTGVQYSEKLSENCVNIWKSFGIYTDSE..
gene-ZOSMA_1G00350                               ..............GPFSKFTRVTML..MPLTGEQYSDKVAENCEKIWKLMKIFTDAE..
AT3G55120                                        ..............GAFEKFIKVTMK..LPLTGQQYSEKVTENCVAIWKQLGLYTDCE..
Os03g60509                                       ..............GDFEKFTRVTMI..LPLTGEQYSDKVTENCVAAWKAAGVYTDAE..
```

```
                                                    130       140       150             160       170
Medicago                                            ..AEAMQKFAEAFKPVNFPPGASVFY..RQSPD..GILGLSFSP.DTSIPE...KEAA.L
Chr16.g28500                                        ............SKESGFPS...............G..QISFSK.DASVPE...AANV.V
Chr1.g57423                                         ..EKAIEKFLEVFKDQNFPPGASILF..TQSPK..GSLTIGFSR.DASVPE...AANA.V
Chr7.g32642                                         ..GKAIEKFIEVFKDQNFPPGASILF..TQSPK..GSLTICFSK.DASVPE...AANA.V
QL08p057674                                         ..TKAVEKFLEVFKDENFTPGSSILF..TQSPK..GSLTIGFSK.DGSIPE...VGKA.V
gene-CFP56_42925                                    ..TKAVEQFLEVFKDESFPPGSSILF..TQSPK..GSLTIAFSK.DGSIPE...VGKA.V
gene-LOC108830048                                   ..AKAVERFLEVFKDQTFPPGASILF..ALSPN..GSLTIAFSK.DDHIPK...TGKA.V
gene-LOC108862091                                   ..AKAVERFLDVFKDETFPPGASILF..ALSPE..GSLTVAFSK.DDSIPE...TGKA.V
gene-LOC115736770                                   ..AAAVEKFREAFKDKSFPPGASILF..TQLPN..GSLTIAFSE.DGSIPE...ASNT.V
gene-C3L33_05584                                    ................PFP....................IGFSK.DGLLPE...KGKV.V
gene-LUZ62_010432                                   ..EKAAIEFEKLFEPIDLPPGSTIFF..THCSS..GQLKVTFSK.DTSVPE...LENG.V
gene-LUZ62_034283                                   ..ANAVDKFKQVFKPETFPPGSSILF..THSPS..GTLSIAFSK.DDSVPE...TNMA.V
gene-LUZ62_049060                                   ..ANAVDKFKQVFKPETFPPGSSILF..THSPS..GTLSIAFSK.DDSVPE...TNMA.V
gene-LUZ62_054959                                   ..EKAAIEFEKLFEPIDLPPGSTIFF..THCSS..GQLKVTFSK.DTSVPE...LENG.V
gene-LUZ62_079350                                   ..ANAVDKFKQVFKPETFPPGSSILF..THSPS..GTLSIAFSK.DDSVPE...TNMA.V
gene-LUZ62_083640                                   ..ANAVDKFKQVFKPETFPPGSSILF..THSPS..GTLSIAFSK.DDSVPE...TNMA.V
gene-LOC8264259                                     ..AKAIDKFLEIFKEENFPPGSSILF..TQLPH..GSVAISFSK.DGSIPE...VENG.L
RchiOBHm_Chr1g0365111                               ..AKAIEKFVEVFKDQTFPPGASILF..TQSPN..GSLTIGFSK.DGSIPA...VGNA.V
RchiOBHm_Chr7g0221431                               ............WRSSKIRPSHLVLLFSTQSPN..GSLTIGFSK.DGSIPA...VGNA.V
Rf070256510                                         ..AKAIGKFTEVFKDQTFPPGASILF..TQSPN..GSLTIGFSK.DGSIPE...VGNA.V
Sspon.01G0031250-1A                                 ..GVAVEKFKEVFKPETFPPGASILF..THSPT..GILTVAFSK.DSSVPA...AGGV.A
Sspon.01G0031250-1T                                 ..GVAVEKFKEVFKPETFPPGASILF..THSPS..GVLTVAFSK.DSSVPA...AGGV.A
Sspon.01G0031250-3C                                 ..GVAVEKFKEVFKPETFPPGASILF..THSPS..GVLTVAFSK.DSSVPV...AGGV.A
ztl01450                                            ..AKAIEKFQEVFKEETFHPGSSILF..TLSSH..GSLAISFSK.DGSVPE...IENA.V
Sapur.010G169300.v5.1                               ..AKAIEKFQEVFKEETFGPGSSINF..TLSSH..GSLAISFSK.DGSVPE...VENA.V
NDS_009150                                          ..GKAVEEFLQVFKDESFSPGASILF..TQSPA..GSLTIAFSK.DGSIPE...QGKA.V
NDS_061126                                          ..GKAVEEFLQVFKDESFSPGASILF..TQSPA..GSLTIAFSK.DGSIPE...QGKA.V
NDS_061132                                          ..GKAVEEFLQVFKDESFSPGASILF..TQSPA..GSLTIAFSK.DGSIPE...QGKA.V
Samuk03G0051900                                     ..AKATEKFIEIFKDETFPPGTSILF..TQSPQ..GSLTISFSK.DDTIPE...VGNA.V
Samuk03G0054000                                     ..AKATEKFIEIFKDETFPPGTSILF..TQSPQ..GSLTISFSK.DDTIPE...VGNA.V
Sb000535g0022                                       ..AKAIEQFIEVFKDETFPPGTSILF..TQSPH..GSLTISFSK.DGTIPE...CGKV.V
EVM20prediction20contig126.452                      ..SEAIDKFLQVFKDETFAPGASILF..TQSPA..GSLTISFSK.DGSIPE...QGKA.V
SECCE5Rv1G0364630                                   ..AAAVDKFKEAFKPHSFAPGASILF..THSPA..GVLTVAFSK.DSSVPE...SGGV.A
gene-G2W53_032366                                   ..AKAIDKFISVFKDQTFPPGSSILF..TQSPN..GSLTIGFSK.DGSIPE...AGCE.V
SETIT_024113mg                                      ..GAAVEEFKAAFKNLSLAPGASVLF..THSPA..GVLTVAFSD.DSSVPE...ASIA.A
SETIT_036958mg                                      ..GAAVDKFKEAFKPETFPPGASILF..THSPA..GVLTVAFSK.DSSVPE...AGGV.A
SEVIR_9G033800v2                                    ..GAAVDKFKEAFKPETFPPGASILF..THSPA..GVLTVAFSK.DSSVPE...AGGV.A
Sc06g0001890                                        ..AKAVEKFKEVFKSETFPPGHSLIF..HHSPL..GSLTIGFSK.HDSVPE...AGSA.V
Sc06g0001900                                        .......................................IGFSK.HDAIPE...VGNA.V
Solyc05g010310.3                                    ..RQAIEKFLNVFQSETFSPGASILF..TQSVV..GSLTISFSD.DDSVPG...TGNV.V
Solyc05g010320.3                                    ..MQAIEKFLDVFQSETFPPGASILF..TQSPL..GSLTISFSE.DDSVPC...VGNA.V
gene-LOC107019217                                   ..RQAIEKFLNVFQSETFSPGASILF..TQSVV..GSLTISFSD.DDSVPG...TGNV.V
gene-LOC107020630                                   ..RQAIEKFLNVFQGETFPPGASILF..TQSPL..GSLTISFSK.DDSVPC...VGNA.V
SORBI_3001G035600                                   ..GVAVEKFKEAFKPETFPPGASILF..THSST..GVLTVAFSK.DSSVPA...SGGV.A
gene-E2542_SST22123                                 ..AKAIEKFVSVFKDETFPPGSSILF..TVSPQ..GSLAISFSK.DGTIPE...VGST.V
gene-LOC110795717                                   ..AKAVEKFLETFKDEKFPPGHSILF..TQSPL..GSLTIGFCE.HAWIPA...AGNA.V
gene-SI8410_06008823                                ..AEAVEKLKDTFKDETFPPGSSILF..TQSPS..GSLTIAFSK.DSTLPE...YGKA.V
gene-STAS_11501                                     ..SKAIEKFLEVFKHENFPPGASILF..TQLPV..GSLTISFSK.DGSIPK...HANA.V
EVM0031032                                          ..SEAIERFLQVLKDETFPPGASILF..TQSPS..GALTISFSK.DSSIPE...TSAA.V
TkA04G183730                                        ..ATTIEKFLEVFKDQNFPPTSSILF..TTSPA..GSLAISFSK.DGSIPE...APDV.V
gene-LOC104825165                                   ..AKAVEKFLKIFKDETFPPGSSILF..ALSPG..SSFTIAFSR.DGSIPE...TGEA.V
gene-HHK36_016491                                   ..AKAVEKFIEVFKDENFPPGNSILF..TQSPL..GSLTIGFSK.HGSLPE...VGNA.V
gene-FRX31_032955                                   ..SKAVEEFIQVFKDETFPPGSSILF..TQSS...PTLTIGFSK.DGSIPK...EVKA.T
TCM_042994                                          ..AKAIEKFLEVFKDENFPPGSSILF..TLSAH..GSLTISFSK.DSSVPE...VGTA.V
Tel5E01G756500                                      ..AAAVDKFKEAFGPHSFAPGASILF..THSPA..GVLTVAFSK.DSSVPE...SGGV.A
gene-TAV2_LOCUS5378                                 ..AKAIEKFLKVFKDHDFPPGSSILF..AICPK..GTLTIAFSK.DEKVPR...SGNA.V
gene-TAV2_LOCUS16152                                ..AKAVERFLEVFKDQTFAPGASILF..ALSPN..GSLTVAFSK.DDSIPE...TGKA.V
EVM20prediction20LG01.1567                          ..AKAVEKFLEVFKDESFAPGASVLF..TQSPL..GSLTITFSK.DGTVPE...VGKA.V
gene-TorRG33x02_127840                              ..AKAIEKFLEVFKDQDFAPGSSVLF..TQSPS..GSLTISFSK.DETIPE...AGNA.V
Tp57577_TGAC_v2_gene10384                           ..AKAIDKFVSVFKDETFPPGSSILF..TVSPEGLGSLTISFSK.DGSIPE...VASA.V
gene-HS088_TW21G01722                               ..AKAIEKFLEAFKDETFPPGSSILF..TQLPN..GSLTISFSK.DGSIPE...AGKV.V
TraesCS5A02G475600                                  ..AAAVDKFKEAFGPHSFAPGASILF..THSPA..GVLTVAFSK.DSSVPE...SGGV.A
TraesCS5B02G488900                                  ..AAAVDKFKEAFGPHSFAPGASILF..THSPA..GVLTVAFSK.DSSVPE...SGGV.A
TraesCS7B02G038200                                  HGSAATSELKGIFYPASFPTGSTIFF..THSAD..GVLQVAFSD.DTKVPE...QESG.R
Tubocapsicum_anomalum_10G026880                     ..SQAIEKFLNVFQSENFPPGASILF..TQSPN..GSLTISFSK.DDSVPG...TGNA.V
Tubocapsicum_anomalum_17G002220                     ..SQAIEKFLNVFQSEDFPSGASILF..TQSPI..GSLTISFSK.DDSVPG...TGNA.V
Urofu.9G032800.v1.1                                 ..GAAVEKFKEAFKPETFPPGASILF..THSPA..GVLTVAFSK.DSSVPE...SGGV.A
VducChr11G242040                                    ..AKAVEKFLEVFQDETFPPGASILF..TQSPH..GLLTIGFSK.DGLLPE...TGKV.V
vmacro07205_Vaccinium_macrocarpon_Stevens_v1        ..AKAVEKFLEVFQDETFPPGASILF..TQSPH..GLLTIGFSK.DGSLPE...TGKA.V
gene-HPP92_020856                                   ..DEAIKNFLEAFETMNFRPGSSIFF..THSPQ..GSFTIGFSE.DGAVPK...AAVV.V
tung.gene.scaffold2716.00003                        ..AKAIDKFLEVFKAEQFPPGSSILF..TQLPH..GSLAITFSK.DGSIPE...TENV.V
gene-LOC114190515                                   ..AEAIDKFVSVFKDETFPPGSSILF..TVSPK..GSLLISFSK.DGSIPE...EAST.V
gene-VitviT2T_019145                                ..AKAIEKFNEVLKDETFPPGNSILF..THSPL..GALTMSFSK.DGSLPE...VGNA.V
W.mirabilis.00703                                   ..TKAIQEFTEAFVGENLPAKGSVVF..VVSPS..G.LGIAISREPSSMAR...ELQA.K
gene-JRO89_XSUnG0164900                             ..AKATEKFNEVFKNENFPPGASILF..TQSPH..GSLTISFSK.DGTIPK...VGNA.V
Zm00001eb062510                                     ..GVAVEKFREVFKPETFAPGASILF..THSPA..GVLTVAFSK.DSSVPA...AGGV.A
gene-ZIOFF_031498                                   ..AAAVGKFKEVFQPETFPPGSSILF..THASS..GSLAIAFGE..........AERG.V
gene-ZIOFF_035070                                   ..AAAVGKFKEVFQPETFPPGSSILF..THASS..GSLAIALGE..........AERG.V
Zla09G001950                                        ..GVAVDKFKEAFKPRSFPPGASILF..THSPA..GVLTVAFSK.DSSVPK...DDVAGA
gene-FEM48_Zijuj03G0079900                          ..AKAIQKFLDIFEPQTFNPGSSIFF..SHSPQ..GSFKISFSK.DDSIPE...VWDA.A
gene-FEM48_Zijuj03G0079800                          ..AKAIEKFLEVFKPHTFPPGSSILF..TLSPK..GSFTVSFSK.DDSLPE...VGNV.V
gene-ZOSMA_1G00350                                  ..AEALDKFKEAFKDKTFPPGSSILF..TQLPT..GDLSVAFSD.DSSVHRD..AETV.V
AT3G55120                                           ..AKAVEKFLEIFKEETFPPGSSILF..ALSPT..GSLTVAFSK.DDSIPE...TGIA.V
Os03g60509                                          ..GAAADKFKEAFKPHSFPPGASILF..THSPP..GVLTVAFSK.DSSVPE.GAVAAA.A
```

```
                                                          180       190       200       210                     220
Medicago                                       IENKAVSSAVLETMIGEHAVSPDLKRCLAARLPAL.......L.NEGAF.KI....GN..
Chr16.g28500                                   IENKLLSEAFLESIVGKHGVSPTAKQSLDARLSEL.......L.NGCKE.SN....GAEA
Chr1.g57423                                    IENKLLSEAVLESIVGKHGVSPAAKQSLAVRLSEL.......L.IGCNE.SNDG.KAGNE
Chr7.g32642                                    IENKLLSEAVLESIVGKHGVSPAAKRSLAARLSEL.......L.NGCK...........
QL08p057674                                    IENKLLSEAVLESIIGKHGVSPEAKECLAKRLSEL.......L.KQDTD.KV....AENQ
gene-CFP56_42925                               IENKLLSEAVLESIIGKHGVSPEAKECLAKRLSEL.......L.KQGTD.KV....AENQ
gene-LOC108830048                              IENKLLAEAVLESIIGKKGVSPGTRLSLAERLSQL.......M.KENKV.EE....DATK
gene-LOC108862091                              IENKLLAEAVLESIIGKNGVSPGARLSVAERLAQL.......M.KENKV.EE....DATK
gene-LOC115736770                              IENKPLSEAVLESMIGKHGVSPQAKTSLASRIHEL.......L.RDCEK.KA....DEVD
gene-C3L33_05584                               IENKQLSEAVLESIVGKHGVSPVAKQSLASRVFEL.......F.KETKP.LK....EY..
gene-LUZ62_010432                              IDCPILKEVVLESIVGENGVSPAAKLSLALRIHDI.......I.NSV............
gene-LUZ62_034283                              IENKALCRAVIESIIGEHGVSPAAKSSIAVRFCEH.......L.KSQSA.AQ....NQDN
gene-LUZ62_049060                              IENKALCRAVIESIIGEHGVSPAAKSSIAVRFCEH.......L.KSQNA.AQ....NQDN
gene-LUZ62_054959                              IDCPILEEAVLESIVGENGVSPAAKLSLALRIHDI.......I.NSV............
gene-LUZ62_079350                              IENKALCRAVIKSIIGEHGVSPAAKSSIAVRFCEH.......L.KSQSA.AQ....NQDN
gene-LUZ62_083640                              IENKALCRAVIESIIGEHGVSPAAKSSIAVRFCEH.......L.KSQSA.AQ....NQDN
gene-LOC8264259                                IENKLLSEAILESIIGKHGVSPAARQSVAARLAYL.......F.EKSTQ.TD....KLDS
RchiOBHm_Chr1g0365111                          IENKLLSESVLESIIGKQGVSPEARKCVATRLSEL.......L.KESDD.CV....A...
RchiOBHm_Chr7g0221431                          IENKLLSESVLESIIGKQGVSPEARKCVATRLSEL.......L.KESDDC.....VAGNG
Rf070256510                                    IENKLLSEAVLESIIGKQGVSPAARQSVAARLSEL.......L.KESDD.SV....AGNG
Sspon.01G0031250-1A                            IENKPLCEAVLESIIGEHGVSPAAKLSLAARVSEL.......L.TKGTA.AA....A.DA
Sspon.01G0031250-1T                            IENKHLCEAVLESIIGEHGVSPAAKLSLAARVSEL.......L.TKGTA.AT....A.DE
Sspon.01G0031250-3C                            IENKHLCEAVLESIIGEHGVSPAAKLSLAARVSEL.......L.TKGTA.AT....A.DE
zt101450                                       IENKLLSAAILESMIGKHGVSPEAKQSLATTLSEL.......L.RETNE.NA....N...
Sapur.010G169300.v5.1                          IENKLLSAAILESMIGKHGVSPEAKQSLATTLSEL.......L.RETNE.NV....N...
NDS_009150                                     IENKLLAEAILESIIGKQGVSPAARQSLAARVSEL.......L.KQTSL.AA....EAQ.
NDS_061126                                     IENKLLAEAILESIIGKQGVSPTTRQSLAARVSEL.......L.KQTSL.AA....EAQ.
NDS_061132                                     IENKLLAEAILESIIGKQGVSPTTRQSLAARVSEL.......L.KQTSL.AA....EAQ.
Samuk03G0051900                                IENKLLSEAILESMIGKHGVSPAAKKSLAARLSIL.......L.NNKEP.E.......DG
Samuk03G0054000                                IENKLLSEAILESMIGKHGVSPAAKKSLAARLSIL.......L.NNKEP.E.......DG
Sb000535g0022                                  IENKLLSEAILESMIGKHGVSPVAKKSLASRFSEL.......L.KGE...........
EVM20prediction20contig126.452                 IENKQLSEAVLESIIGKHGVSPSAKQSLAARLSEL.......F................
SECCE5Rv1G0364630                              IENARLCEAVLESIIGEHGVSPAAKLSLATRVAEL.......L.KEAA...H....AGGE
gene-G2W53_032366                              IENRLLSESVLESIIGKNGVSPPARHSVASRLSDL.......F.KQEWC.FS.......
SETIT_024113mg                                 IDNKALCEAVLESIIGERSVSPATKQSIATRVPEI.......L.KGGA...........
SETIT_036958mg                                 IENRPLCEAVLESIIGKHGVSPAAKLSIAARVSEL.......L.NGAET.SP....AGDA
SEVIR_9G033800v2                               IENRPLCEAVLESIIGKHGVSPAAKLSIAARVSEL.......L.NGAET.SP....AGDA
Sc06g0001890                                   IDNKHLSEAVLESIIGKHGVSPVAKQSLASRMAAL.......L.NENVA.KE....ENVP
Sc06g0001900                                   IENKHMSEAVLESIIGKNGVSPAAKQSIAHRMAAI.......L.NDNVV.GE....GKVA
Solyc05g010310.3                               IENKPLSEAVLESMIGKNGVSPAAKRSLAKRVSEM.......L.EKSNA.EE....ST..
Solyc05g010320.3                               IENKQLSEAVLDSIIGEHGVSPAAKCSIAKRVSEL.......L.VCEKP.GS.......
gene-LOC107019217                              IENKPLSEAVLESMIGKNGVSPAAKRSLAKRVSEM.......L.EKSNA.EE....ST..
gene-LOC107020630                              IENKQLSEAVLDSIIGEHGVSPAAKCSIAKRVSEL.......L.VCEKP.GS.......
SORBI_3001G035600                              IENKHLCEAVLESIIGEHGVSPAAKLSLAARVSEL.......L.TKGTA.GA....A.DA
gene-E2542_SST22123                            IENKLLSEAVLESMIGKHGVSPAAKQSLASRLSEL.......F.KEGGV.VS....LIND
gene-LOC110795717                              IENKNLSEAVLESIIGKHGVSPQAKESIAVRVSEL.......L.NSEKT.VE....ANAT
gene-SI8410_06008823                           IENQAMSQAVLESVIGQHGVSPAAKKSLASRISEL.......L.KSVNG.GE....NGHV
gene-STAS_11501                                IQNKQLSEAVLESIIGKNGVSPAAKQSLALRLS..........................
EVM0031032                                     IKNKPLSEAVLESIIGKEGVSPEAKGSLATRLAEV.......L.KEEVE.KT....T...
TkA04G183730                                   LENEKLGQAVIESVIGKYGVSPAAKESLASRLSDF.......M.EEFEG.KA....NASG
gene-LOC104825165                              IENKLLGEAVLESIVGKNGVSPDTRRSLAKRLSQL.......L.KSENI.IG....AGQE
gene-HHK36_016491                              IENKQLSEAILESIIGKHGVSPEAKRNLAVRVSEL.......I.KEVDA.KK.......
gene-FRX31_032955                              IANKPLSEAVLESIIGKHGVSPEAKCSLAVRISEL.......M.KNQGD.TL....EEGA
TCM_042994                                     IENKLLANAVLESIVGKDGVSPVARKSMASRLSAL.......F.DDTVE.KA....AQNG
Tel5E01G756500                                 IENARLCEAVLESIIGEHGVSPAAKLSLATRVAEL.......L.KGAA...H....AGGE
gene-TAV2_LOCUS5378                            IKNKLFGEAIIESMIGKNGVSPETKKSLARRFAKL.......M.EQSNA.PNTFA.TQN.
gene-TAV2_LOCUS16152                           IENKLLAEAVLESIIGKKGVSPGTRLSIAERLAKL.......M.KKKKV.EE....DASS
EVM20prediction20LG01.1567                     IENKLLSEAVLESIIGKHGVSPAAKQNLAARISEL.......L.KEECE.KP....ETEK
gene-TorRG33x02_127840                         IENKLLSEAVLQSIIGKNGVSPEARRSVASRLSEL.......L.KENTT.IA....TATT
Tp57577_TGAC_v2_gene10384                      IENKLLSQAVLESMIGAHGVSPAAKQSLATRLSEL.......F.KEVGD.AN....N...
gene-HS088_TW21G01722                          IENKLLSEAILESIIGKQGVSPAAKKSLAARFPVL.......L.KESEK.VA....STEK
TraesCS5A02G475600                             IENARLCEAVLESIIGEHGVSPAAKLSLATRVAEL.......L.KGA.........AGGE
TraesCS5B02G488900                             IDNARLCEAVLESIIGEHGVSPAAKLSLATRVAEL.......L.KGAS...H....AGGE
TraesCS7B02G038200                             VDSRALSEAVLESIIGDNGVSPLARRSLAFRLCGL.......I.NHK............
Tubocapsicum_anomalum_10G026880                IENKQLSEAVLESIIGKHGVSPSAKFSLAKRVSELNWSLLRTI.KERNH.N.......CE
Tubocapsicum_anomalum_17G002220                IENKQLSEAVLESIIGKHGVSPAAKFSLAKRVSEL.......L.EKSND.EV....PVCE
Urofu.9G032800.v1.1                            IENKPLCEAVLESIIGEHGVSPAAKVSIAARVSEL.......L.KGGKA.NP....AGGA
VducChr11G242040                               IENKQLSEAVLESIIGKHGVSPAAKQSLAARMSAL.......F.KEA............
vmacro07205_Vaccinium_macrocarpon_Stevens_v1   IENKQLSEAVLESIIGKHGVSPAAKQSLAARISAL.......F.KEA............
gene-HPP92_020856                              IENEKLSNAILESIIGEKGVSPAAKQSLAHRMSGL.......F.LCMGV.KEII.SEAN.
tung.gene.scaffold2716.00003                   IENKLLSEAILESIIGKHGVSPEARQCMASRLAEL.......F.EKSEQ.VG....ELIE
gene-LOC114190515                              IENKLLSEAVLESMIGKHGVSPAAKQSLATRLSEL.......F.KEEGV.PE....SHN.
gene-VitviT2T_019145                           IENKLLTEAVLESIIGKHGVSPEAKKSLAARLSEL.......FCKEAGD.EK.......
W.mirabilis.00703                              IGNRSLAEAVLLSIIGKDGVSPHARASVASRLSSL.......L.KH.............
gene-JRO89_XSUnG0164900                        IENKLLSEAILESMIGKQGVSPPAKKSLAARLSKL.......L.NEDGK.TE....SKKE
Zm00001eb062510                                IENKRLCEAVLESIIGERGVSPAAKLSLAARVSEL.......L.AKETA.AP....A.DA
gene-ZIOFF_031498                              IENKALSEAILESIIGEHGVSPAAKRSLALRISEV.......L.KEPLG.PE....ETKT
gene-ZIOFF_035070                              IENKALSEAILESIIGEHGVSPAAKRSLALRISEV.......L.KEPLG.PE....ETKT
Zla09G001950                                   IENRALCETVLESIIGEHGVSPAAKLSLAARVAEL.......L.KVDAP.GH....A...
gene-FEM48_Zijuj03G0079900                     IESRLLFEAVLESIIGKSGVSPATRKSLAARLPLL.......L.KEISD.DD....RDLD
gene-FEM48_Zijuj03G0079800                     IENKLLSEAILESIIGKQGVSPQARKTLAERLLEL.......F.KLENS.GD....GNIA
gene-ZOSMA_1G00350                             ISNKLLSEAVLESIIGEHGVSPATRENMAERFVRL.......F.E.............
AT3G55120                                      IENKLLAEAVLESIIGKNGVSPGTRLSVAERLSQL.......M.MKNKD.EK....EVS.
Os03g60509                                     IENRALCEAVLDSIIGEHGVSPAAKRSIAARVSQL.......L.KAEST.GD....V...
```

**Medicago**	....
**Chr16.g28500**	R.NEK......................VEA..............................
**Chr1.g57423**	.........RDCQMAPPPSLARTSDRNDYISTV..............RQTSGILQHFVP.
**Chr7.g32642**	....
**QL08p057674**	.........Q...................CEC...............EGH..........
**gene-CFP56_42925**	.........H.....................C...............EGH..........
**gene-LOC108830048**	T..DN..QDEAND..............LSLGDKLA...........KEN...........
**gene-LOC108862091**	T...D..QEEAND..............LS....LA...........KEN...........
**gene-LOC115736770**	K.NV...APTAAE..............VSVA.............................
**gene-C3L33_05584**	....
**gene-LUZ62_010432**	....
**gene-LUZ62_034283**	P..........................VSINA............................
**gene-LUZ62_049060**	P..........................VSTNA............................
**gene-LUZ62_054959**	....
**gene-LUZ62_079350**	P..........................VSINA............................
**gene-LUZ62_083640**	P..........................ASINA............................
**gene-LOC8264259**	T.TSS...
**RchiOBHm_Chr1g0365111**	....
**RchiOBHm_Chr7g0221431**	E.VKE......................AEVKA............................
**Rf070256510**	.........K...................VEKC..............TKEAEV.......
**Sspon.01G0031250-1A**	P.QV.....EP................FSVTV............................
**Sspon.01G0031250-1T**	P.QA.....EP................VSVTA............................
**Sspon.01G0031250-3C**	P.QA.....EP................VSVTA............................
**ztl01450**	....
**Sapur.010G169300.v5.1**	....
**NDS_009150**	....
**NDS_061126**	....
**NDS_061132**	....
**Samuk03G0051900**	.........K...................PEA...............EKCAKE.......
**Samuk03G0054000**	.........K...................PEA...............EKCAKE.......
**Sb000535g0022**	....
**EVM20prediction20contig126.452**	....
**SECCE5Rv1G0364630**	P.AA.....EP................VSVSV............................
**gene-G2W53_032366**	....
**SETIT_024113mg**	....
**SETIT_036958mg**	Q.QA.....EP................VPVSA............................
**SEVIR_9G033800v2**	Q.QA.....EP................VPVSA............................
**Sc06g0001890**	P.QV...NEK.................ITISEKLNQ.KLDEEVKVGQENGNA........
**Sc06g0001900**	S.QEE...
**Solyc05g010310.3**	....
**Solyc05g010320.3**	....ELSSVQ.......
**gene-LOC107019217**	....
**gene-LOC107020630**	....ELSSVQ.......
**SORBI_3001G035600**	P.QA.....EP................VSVTA............................
**gene-E2542_SST22123**	Y.VTN...
**gene-LOC110795717**	L.GVE..NGKT................TKVVSEGIHG......QKDVEV...........
**gene-SI8410_06008823**	E......AAPPSLN.............GHVGPAAPL..........PENGHVKAAPPSED
**gene-STAS_11501**	....
**EVM0031032**	....
**TkA04G183730**	E...
**gene-LOC104825165**	EVQQ.......................ASVGDESA...........VEN...........
**gene-HHK36_016491**	.............................LDC............................
**gene-FRX31_032955**	L...QKVENGVPAKIYP...
**TCM_042994**	.........K...................PECQ...........................
**Tel5E01G756500**	P.AA.....EP................VSVSV............................
**gene-TAV2_LOCUS5378**	....
**gene-TAV2_LOCUS16152**	L..TD..QEEATD..............LSLGDKLA...........KEN...........
**EVM20prediction20LG01.1567**	ELLKEE.CEK...................SET...............EKVSGEKK.....
**gene-TorRG33x02_127840**	.............................TEA...............EKVVPEK......
**Tp57577_TGAC_v2_gene10384**	....
**gene-HS088_TW21G01722**	A..........................VELEAKNVNGS......................
**TraesCS5A02G475600**	P.AV.....EP................VSVSV............................
**TraesCS5B02G488900**	P.AA.....EP................VSVSV............................
**TraesCS7B02G038200**	....
**Tubocapsicum_anomalum_10G026880**	A.MTWAG....................VKVKSNCMDRN......................
**Tubocapsicum_anomalum_17G002220**	K...TGSEEVAIQ.......
**Urofu.9G032800.v1.1**	A.QA...EVEPV...............VPVSA............................
**VducChr11G242040**	....
**vmacro07205_Vaccinium_macrocarpon_Stevens_v1**	....
**gene-HPP92_020856**	....
**tung.gene.scaffold2716.00003**	K........K...................IQN...............EKLIQKNP.....
**gene-LOC114190515**	....
**gene-VitviT2T_019145**	.............................IEA...............EKVAPVAC.....
**W.mirabilis.00703**	....
**gene-JRO89_XSUnG0164900**	K.PGKL.DEKDTE...G..........TIVEREL..........................
**Zm00001eb062510**	P.QA.....EP................VSITA............................
**gene-ZIOFF_031498**	V.NS.......................VSV..............................
**gene-ZIOFF_035070**	V.NS.......................VSV..............................
**Zla09G001950**	P.AV.....EP................VSVSV............................
**gene-FEM48_Zijuj03G0079900**	R.NVI..NGKVDLN.............QKVSGHGHE.KVPER.KGQQETAKKI.......
**gene-FEM48_Zijuj03G0079800**	A.ATNGK....................VEVTKKVLEGKM....TKEVEQLGNKKV.....
**gene-ZOSMA_1G00350**	....
**AT3G55120**	............D..............HSVEEKLA...........KEN...........
**Os03g60509**	A.AA.....EP................APVSA............................

```
Medicago                                          ............................................................
Chr16.g28500                                      ............................................................
Chr1.g57423                                       .RRRRFVNFARVRGLEIQGNFVKFTAIGVYLEDSAVPQLAFKWKGKTAKELTESVEFFRD
Chr7.g32642                                       ............................................................
QL08p057674                                       ............................................................
gene-CFP56_42925                                  ............................................................
gene-LOC108830048                                 ............................................................
gene-LOC108862091                                 ............................................................
gene-LOC115736770                                 ............................................................
gene-C3L33_05584                                  ............................................................
gene-LUZ62_010432                                 ............................................................
gene-LUZ62_034283                                 ............................................................
gene-LUZ62_049060                                 ............................................................
gene-LUZ62_054959                                 ............................................................
gene-LUZ62_079350                                 ............................................................
gene-LUZ62_083640                                 ............................................................
gene-LOC8264259                                   ............................................................
RchiOBHm_Chr1g0365111                             ............................................................
RchiOBHm_Chr7g0221431                             ............................................................
Rf070256510                                       ............................................................
Sspon.01G0031250-1A                               ............................................................
Sspon.01G0031250-1T                               ............................................................
Sspon.01G0031250-3C                               ............................................................
ztl01450                                          ............................................................
Sapur.010G169300.v5.1                             ............................................................
NDS_009150                                        ............................................................
NDS_061126                                        ............................................................
NDS_061132                                        ............................................................
Samuk03G0051900                                   ............................................................
Samuk03G0054000                                   ............................................................
Sb000535g0022                                     ............................................................
EVM20prediction20contig126.452                    ............................................................
SECCE5Rv1G0364630                                 ............................................................
gene-G2W53_032366                                 ............................................................
SETIT_024113mg                                    ............................................................
SETIT_036958mg                                    ............................................................
SEVIR_9G033800v2                                  ............................................................
Sc06g0001890                                      ............................................................
Sc06g0001900                                      ............................................................
Solyc05g010310.3                                  ............................................................
Solyc05g010320.3                                  ............................................................
gene-LOC107019217                                 ............................................................
gene-LOC107020630                                 ............................................................
SORBI_3001G035600                                 ............................................................
gene-E2542_SST22123                               ............................................................
gene-LOC110795717                                 ............................................................
gene-SI8410_06008823                              G...........................................................
gene-STAS_11501                                   ............................................................
EVM0031032                                        ............................................................
TkA04G183730                                      ............................................................
gene-LOC104825165                                 ............................................................
gene-HHK36_016491                                 ............................................................
gene-FRX31_032955                                 ............................................................
TCM_042994                                        ............................................................
Tel5E01G756500                                    ............................................................
gene-TAV2_LOCUS5378                               ............................................................
gene-TAV2_LOCUS16152                              ............................................................
EVM20prediction20LG01.1567                        ............................................................
gene-TorRG33x02_127840                            ............................................................
Tp57577_TGAC_v2_gene10384                         ............................................................
gene-HS088_TW21G01722                             ............................................................
TraesCS5A02G475600                                ............................................................
TraesCS5B02G488900                                ............................................................
TraesCS7B02G038200                                ............................................................
Tubocapsicum_anomalum_10G026880                   ............................................................
Tubocapsicum_anomalum_17G002220                   ............................................................
Urofu.9G032800.v1.1                               ............................................................
VducChr11G242040                                  ............................................................
vmacro07205_Vaccinium_macrocarpon_Stevens_v1      ............................................................
gene-HPP92_020856                                 ............................................................
tung.gene.scaffold2716.00003                      ....................................................QNIE....
gene-LOC114190515                                 ............................................................
gene-VitviT2T_019145                              ............................................................
W.mirabilis.00703                                 ............................................................
gene-JRO89_XSUnG0164900                           ............................................................
Zm00001eb062510                                   ............................................................
gene-ZIOFF_031498                                 ............................................................
gene-ZIOFF_035070                                 ............................................................
Zla09G001950                                      ............................................................
gene-FEM48_Zijuj03G0079900                        ............................................................
gene-FEM48_Zijuj03G0079800                        ............................................................
gene-ZOSMA_1G00350                                ............................................................
AT3G55120                                         ............................................................
Os03g60509                                        ............................................................
```

```
Medicago                                          ............................................................
Chr16.g28500                                      ............................................................
Chr1.g57423                                       IVTGPFEKFIQVTTILPLTGQQYSEKVSENCVAFWKSVGIYTDAEGKAIEKFLEVFKDQN
Chr7.g32642                                       ............................................................
QL08p057674                                       ............................................................
gene-CFP56_42925                                  ............................................................
gene-LOC108830048                                 ............................................................
gene-LOC108862091                                 ............................................................
gene-LOC115736770                                 ............................................................
gene-C3L33_05584                                  ............................................................
gene-LUZ62_010432                                 ............................................................
gene-LUZ62_034283                                 ............................................................
gene-LUZ62_049060                                 ............................................................
gene-LUZ62_054959                                 ............................................................
gene-LUZ62_079350                                 ............................................................
gene-LUZ62_083640                                 ............................................................
gene-LOC8264259                                   ............................................................
RchiOBHm_Chr1g0365111                             ............................................................
RchiOBHm_Chr7g0221431                             ............................................................
Rf070256510                                       .................................................EA.........
Sspon.01G0031250-1A                               ............................................................
Sspon.01G0031250-1T                               ............................................................
Sspon.01G0031250-3C                               ............................................................
ztl01450                                          ............................................................
Sapur.010G169300.v5.1                             ............................................................
NDS_009150                                        ............................................................
NDS_061126                                        ............................................................
NDS_061132                                        ............................................................
Samuk03G0051900                                   ..................................................KPVEL.....
Samuk03G0054000                                   ..................................................KPVEL.....
Sb000535g0022                                     ............................................................
EVM20prediction20contig126.452                    ............................................................
SECCE5Rv1G0364630                                 ............................................................
gene-G2W53_032366                                 ............................................................
SETIT_024113mg                                    ............................................................
SETIT_036958mg                                    ............................................................
SEVIR_9G033800v2                                  ............................................................
Sc06g0001890                                      ............................................................
Sc06g0001900                                      ............................................................
Solyc05g010310.3                                  ............................................................
Solyc05g010320.3                                  ............................................................
gene-LOC107019217                                 ............................................................
gene-LOC107020630                                 ............................................................
SORBI_3001G035600                                 ............................................................
gene-E2542_SST22123                               ............................................................
gene-LOC110795717                                 ............................................................
gene-SI8410_06008823                              ............................................................
gene-STAS_11501                                   ............................................................
EVM0031032                                        ............................................................
TkA04G183730                                      ............................................................
gene-LOC104825165                                 ............................................................
gene-HHK36_016491                                 ............................................................
gene-FRX31_032955                                 ............................................................
TCM_042994                                        ............................................................
Tel5E01G756500                                    ............................................................
gene-TAV2_LOCUS5378                               ............................................................
gene-TAV2_LOCUS16152                              ............................................................
EVM20prediction20LG01.1567                        ....PVE.....................................................
gene-TorRG33x02_127840                            ............................................................
Tp57577_TGAC_v2_gene10384                         ............................................................
gene-HS088_TW21G01722                             ............................................................
TraesCS5A02G475600                                ............................................................
TraesCS5B02G488900                                ............................................................
TraesCS7B02G038200                                ............................................................
Tubocapsicum_anomalum_10G026880                   ............................................................
Tubocapsicum_anomalum_17G002220                   ............................................................
Urofu.9G032800.v1.1                               ............................................................
VducChr11G242040                                  ............................................................
vmacro07205_Vaccinium_macrocarpon_Stevens_v1      ............................................................
gene-HPP92_020856                                 ............................................................
tung.gene.scaffold2716.00003                      ...............................................SIEK.........
gene-LOC114190515                                 ............................................................
gene-VitviT2T_019145                              ............................................................
W.mirabilis.00703                                 ............................................................
gene-JRO89_XSUnG0164900                           ............................................................
Zm00001eb062510                                   ............................................................
gene-ZIOFF_031498                                 ............................................................
gene-ZIOFF_035070                                 ............................................................
Zla09G001950                                      ............................................................
gene-FEM48_Zijuj03G0079900                        ............................................................
gene-FEM48_Zijuj03G0079800                        ............................................................
gene-ZOSMA_1G00350                                ............................................................
AT3G55120                                         ............................................................
Os03g60509                                        ............................................................
```

```
Medicago                                      ............................................................
Chr16.g28500                                  ............................................................
Chr1.g57423                                   FPPGASILFTQSPKGSLTISFSRDASVPEAANAVIENKLLSEAVLESIVGKHGVSPAAKQ
Chr7.g32642                                   ............................................................
QL08p057674                                   ............................................................
gene-CFP56_42925                              ............................................................
gene-LOC108830048                             ............................................................
gene-LOC108862091                             ............................................................
gene-LOC115736770                             ............................................................
gene-C3L33_05584                              ..................................................HHGWCI....
gene-LUZ62_010432                             ............................................................
gene-LUZ62_034283                             ............................................................
gene-LUZ62_049060                             ............................................................
gene-LUZ62_054959                             ............................................................
gene-LUZ62_079350                             ............................................................
gene-LUZ62_083640                             ............................................................
gene-LOC8264259                               ............................................................
RchiOBHm_Chr1g0365111                         ............................................................
RchiOBHm_Chr7g0221431                         ............................................................
Rf070256510                                   ............................................................
Sspon.01G0031250-1A                           ............................................................
Sspon.01G0031250-1T                           ............................................................
Sspon.01G0031250-3C                           ............................................................
ztl01450                                      ............................................................
Sapur.010G169300.v5.1                         ............................................................
NDS_009150                                    ............................................................
NDS_061126                                    ............................................................
NDS_061132                                    ............................................................
Samuk03G0051900                               .......................DGKDIRVA.............................
Samuk03G0054000                               .......................DGKDIRVA.............................
Sb000535g0022                                 ............................................................
EVM20prediction20contig126.452                ............................................................
SECCE5Rv1G0364630                             ............................................................
gene-G2W53_032366                             ............................................................
SETIT_024113mg                                ............................................................
SETIT_036958mg                                ............................................................
SEVIR_9G033800v2                              ............................................................
Sc06g0001890                                  ............................................................
Sc06g0001900                                  ............................................................
Solyc05g010310.3                              ............................................................
Solyc05g010320.3                              ............................................................
gene-LOC107019217                             ............................................................
gene-LOC107020630                             ............................................................
SORBI_3001G035600                             ............................................................
gene-E2542_SST22123                           ............................................................
gene-LOC110795717                             ............................................................
gene-SI8410_06008823                          .........................TTPEKTSQ...........................
gene-STAS_11501                               ............................................................
EVM0031032                                    ............................................................
TkA04G183730                                  ............................................................
gene-LOC104825165                             ............................................................
gene-HHK36_016491                             ............................................................
gene-FRX31_032955                             ..........................LPN...............................
TCM_042994                                    ............................................................
Tel5E01G756500                                ............................................................
gene-TAV2_LOCUS5378                           ............................................................
gene-TAV2_LOCUS16152                          ............................................................
EVM20prediction20LG01.1567                    ............................................................
gene-TorRG33x02_127840                        ............................................................
Tp57577_TGAC_v2_gene10384                     ............................................................
gene-HS088_TW21G01722                         ............................................................
TraesCS5A02G475600                            ............................................................
TraesCS5B02G488900                            ............................................................
TraesCS7B02G038200                            ............................................................
Tubocapsicum_anomalum_10G026880               ............................................................
Tubocapsicum_anomalum_17G002220               ............................................................
Urofu.9G032800.v1.1                           ............................................................
VducChr11G242040                              ............................................................
vmacro07205_Vaccinium_macrocarpon_Stevens_v1  ............................................................
gene-HPP92_020856                             ............................................................
tung.gene.scaffold2716.00003                  .SP...................QNGN..................................
gene-LOC114190515                             ............................................................
gene-VitviT2T_019145                          ............................................................
W.mirabilis.00703                             ............................................................
gene-JRO89_XSUnG0164900                       ............................................................
Zm00001eb062510                               ............................................................
gene-ZIOFF_031498                             ............................................................
gene-ZIOFF_035070                             ............................................................
Zla09G001950                                  ............................................................
gene-FEM48_Zijuj03G0079900                    ............................................................
gene-FEM48_Zijuj03G0079800                    ............................................................
gene-ZOSMA_1G00350                            ............................................................
AT3G55120                                     ............................................................
Os03g60509                                    ............................................................
```

**Medicago**	....
**Chr16.g28500**	....
**Chr1.g57423**	SLAARLSELLNGCKESNCAEAGNEKVEA
**Chr7.g32642**	....
**QL08p057674**	....
**gene-CFP56_42925**	....
**gene-LOC108830048**	....
**gene-LOC108862091**	....
**gene-LOC115736770**	....
**gene-C3L33_05584**	....
**gene-LUZ62_010432**	....
**gene-LUZ62_034283**	....
**gene-LUZ62_049060**	....
**gene-LUZ62_054959**	....
**gene-LUZ62_079350**	....
**gene-LUZ62_083640**	....
**gene-LOC8264259**	....
**RchiOBHm_Chr1g0365111**	....
**RchiOBHm_Chr7g0221431**	....
**Rf070256510**	....
**Sspon.01G0031250-1A**	....
**Sspon.01G0031250-1T**	....
**Sspon.01G0031250-3C**	....
**ztl01450**	....
**Sapur.010G169300.v5.1**	....
**NDS_009150**	....
**NDS_061126**	....
**NDS_061132**	....
**Samuk03G0051900**	....
**Samuk03G0054000**	....
**Sb000535g0022**	....
**EVM20prediction20contig126.452**	....
**SECCE5Rv1G0364630**	....
**gene-G2W53_032366**	....
**SETIT_024113mg**	....
**SETIT_036958mg**	....
**SEVIR_9G033800v2**	....
**Sc06g0001890**	....
**Sc06g0001900**	....
**Solyc05g010310.3**	....
**Solyc05g010320.3**	....
**gene-LOC107019217**	....
**gene-LOC107020630**	....
**SORBI_3001G035600**	....
**gene-E2542_SST22123**	....
**gene-LOC110795717**	....
**gene-SI8410_06008823**	....
**gene-STAS_11501**	....
**EVM0031032**	....
**TkA04G183730**	....
**gene-LOC104825165**	....
**gene-HHK36_016491**	....
**gene-FRX31_032955**	....
**TCM_042994**	....
**Tel5E01G756500**	....
**gene-TAV2_LOCUS5378**	....
**gene-TAV2_LOCUS16152**	....
**EVM20prediction20LG01.1567**	....
**gene-TorRG33x02_127840**	....
**Tp57577_TGAC_v2_gene10384**	....
**gene-HS088_TW21G01722**	....
**TraesCS5A02G475600**	....
**TraesCS5B02G488900**	....
**TraesCS7B02G038200**	....
**Tubocapsicum_anomalum_10G026880**	....
**Tubocapsicum_anomalum_17G002220**	....
**Urofu.9G032800.v1.1**	....
**VducChr11G242040**	....
**vmacro07205_Vaccinium_macrocarpon_Stevens_v1**	....
**gene-HPP92_020856**	....
**tung.gene.scaffold2716.00003**	....
**gene-LOC114190515**	....
**gene-VitviT2T_019145**	....
**W.mirabilis.00703**	....
**gene-JRO89_XSUnG0164900**	....
**Zm00001eb062510**	....
**gene-ZIOFF_031498**	....
**gene-ZIOFF_035070**	....
**Zla09G001950**	....
**gene-FEM48_Zijuj03G0079900**	....
**gene-FEM48_Zijuj03G0079800**	....
**gene-ZOSMA_1G00350**	....
**AT3G55120**	....
**Os03g60509**	....
