# Supplementary material for: Evolutionary landscape of plant chalcone isomerase-fold gene families
Source: Front Plant Sci. 2025 Mar 28;16:1559547. doi: 10.3389/fpls.2025.1559547 (PMC11985768; doi:10.3389/fpls.2025.1559547)
Supplement: Supplementary Table 1 — The genomic data source of 259 species. [file DataSheet1.zip › Supplementary_information/Figure S4.pdf]

```
Medicago                                      ............................................................
gene-CEY00_Acc27669                           ............................................................
Aspi01Gene46726                               ............................................................
Azfi_s0030.g024403                            ............................................................
LXT01105                                      ............................................................
LXT05484                                      ............................................................
C.cajan_29159                                 ............................................................
C.cajan_29160                                 ............................................................
C.cajan_29161                                 ............................................................
C.cajan_48225                                 ............................................................
Cecan.1G005800.V3.1                           ............................................................
Cecan.2G031800.V3.1                           ............................................................
Cecan.5G005100.V3.1                           ............................................................
gene-LOC101500746                             ............................................................
gene-LOC101501059                             ............................................................
gene-IFM89_008902                             ............................................................
EUGRSUZ_J01152                                ............................................................
gene-GLYMA_10G292200v4                        ............................................................
gene-GLYMA_20G241500v4                        ............................................................
gene-GLYMA_20G241600v4                        ............................................................
gene-LOC110425500                             ............................................................
Lchi30763                                     ............................................................
Lchi33565                                     ............................................................
MARPO_0167s0012                               ............................................................
Mvestita10057                                 M.......LQHLCCVYYILHSSYFKDTMRREARCRTSNHTQTVAVYLYGHENDDIFSRLA
gene-LOC11440741                              ............................................................
gene-LOC11424676                              ............................................................
gene-LOC11440740                              ............................................................
gene-MLD38_038120                             ............................................................
gene-E3N88_23837                              ............................................................
gene-E3N88_23910                              ............................................................
gene-E3N88_23911                              ............................................................
gene-PHAVU_003G216600g                        ............................................................
gene-PHAVU_007G008600g                        ............................................................
PIPE20159                                     MVINLQLR..........................RS........................
Pis12873                                      ............................................................
Psat6g237920                                  MFHQ........................................................
Psat6g238360                                  ............................................................
gene-LOC116187009                             MFQVLT......................................................
gene-LOC116187298                             ............................................................
gene-LUZ62_030497                             ............................................................
gene-LUZ62_045336                             ............................................................
gene-LUZ62_075590                             ............................................................
gene-SELMODRAFT_151616                        ............................................................
gene-SELMODRAFT_444624                        ............................................................
gene-G2W53_032365                             ............................................................
gene-E2542_SST22125                           ............................................................
gene-E2542_SST22126                           ............................................................
gene-E2542_SST22127                           ............................................................
Spipo29G0020600.v2                            ............................................................
Tp57577_TGAC_v2_gene30361                     ............................................................
VducChr2G012310                               ............................................................
vmacro00636_Vaccinium_macrocarpon_Stevens_v1  ............................................................
gene-HPP92_020855                             ............................................................
gene-LOC114189846                             ............................................................
gene-LOC114192130                             ............................................................
gene-JRO89_XS14G0048500                       ............................................................
```

```
Medicago                                        ............................................................
gene-CEY00_Acc27669                             ............................................................
Aspi01Gene46726                                 ............................................................
Azfi_s0030.g024403                              ............................................................
LXT01105                                        ............................................................
LXT05484                                        ............................................................
C.cajan_29159                                   ............................................................
C.cajan_29160                                   ............................................................
C.cajan_29161                                   ............................................................
C.cajan_48225                                   ............................................................
Cecan.1G005800.V3.1                             ............................................................
Cecan.2G031800.V3.1                             ............................................................
Cecan.5G005100.V3.1                             ............................................................
gene-LOC101500746                               ............................................................
gene-LOC101501059                               ............................................................
gene-IFM89_008902                               ............................................................
EUGRSUZ_J01152                                  ............................................................
gene-GLYMA_10G292200v4                          ............................................................
gene-GLYMA_20G241500v4                          ............................................................
gene-GLYMA_20G241600v4                          ............................................................
gene-LOC110425500                               ............................................................
Lchi30763                                       ............................................................
Lchi33565                                       ............................................................
MARPO_0167s0012                                 ............................................................
Mvestita10057                                   FPCFKQ..........VSCHQQEHY...................................
gene-LOC11440741                                ............................................................
gene-LOC11424676                                ............................................................
gene-LOC11440740                                ............................................................
gene-MLD38_038120                               ............................................................
gene-E3N88_23837                                ............................................................
gene-E3N88_23910                                ............................................................
gene-E3N88_23911                                ............................................................
gene-PHAVU_003G216600g                          ............................................................
gene-PHAVU_007G008600g                          ............................................................
PIPE20159                                       .HSLLPACPSQQLPPTSRHLVFQLCHAAISTPGSSAYQPSSPTLILPPNYRLAIARSPSY
Pis12873                                        ............................................................
Psat6g237920                                    ............................................................
Psat6g238360                                    ............................................................
gene-LOC116187009                               ................IMRVLFILS...................................
gene-LOC116187298                               ............................................................
gene-LUZ62_030497                               ............................................................
gene-LUZ62_045336                               ............................................................
gene-LUZ62_075590                               ............................................................
gene-SELMODRAFT_151616                          ............................................................
gene-SELMODRAFT_444624                          ............................................................
gene-G2W53_032365                               ............................................................
gene-E2542_SST22125                             ............................................................
gene-E2542_SST22126                             ............................................................
gene-E2542_SST22127                             ............................................................
Spipo29G0020600.v2                              ............................................................
Tp57577_TGAC_v2_gene30361                       ............................................................
VducChr2G012310                                 ............................................................
vmacro00636_Vaccinium_macrocarpon_Stevens_v1    ............................................................
gene-HPP92_020855                               ............................................................
gene-LOC114189846                               ............................................................
gene-LOC114192130                               ............................................................
gene-JRO89_XS14G0048500                         ............................................................
```

	1
**Medicago**	....M...............
**gene-CEY00_Acc27669**	....MS.LS..............
**Aspi01Gene46726**	....
**Azfi_s0030.g024403**	....
**LXT01105**	....MA.P...............
**LXT05484**	....MA.P...............
**C.cajan_29159**	....MA.MT..............
**C.cajan_29160**	.....................................MV.AE.T...............
**C.cajan_29161**	....MA.................
**C.cajan_48225**	....MATA...............
**Cecan.1G005800.V3.1**	....MA.P...............
**Cecan.2G031800.V3.1**	....MA.S...............
**Cecan.5G005100.V3.1**	....MA.P...............
**gene-LOC101500746**	....MT.T...............
**gene-LOC101501059**	....MT.T...............
**gene-IFM89_008902**	....MA.................
**EUGRSUZ_J01152**	....MS.PL..............
**gene-GLYMA_10G292200v4**	....MA.T...............
**gene-GLYMA_20G241500v4**	....MA.................
**gene-GLYMA_20G241600v4**	....MA.T...............
**gene-LOC110425500**	....MS.S...............
**Lchi30763**	....MG.S...............
**Lchi33565**	....MG.S...............
**MARPO_0167s0012**	....ME.STHS............
**Mvestita10057**	............................PAASSSNSKQVNLA.......TLPVVLGLLY
**gene-LOC11440741**	....MATA...............
**gene-LOC11424676**	....MAKA...............
**gene-LOC11440740**	....M...............
**gene-MLD38_038120**	....MS.................
**gene-E3N88_23837**	....MN.SDNNDGD.........
**gene-E3N88_23910**	....MA.N...............
**gene-E3N88_23911**	....MA.N...............
**gene-PHAVU_003G216600g**	......................................M.MA.T...............
**gene-PHAVU_007G008600g**	....MA.T...............
**PIPE20159**	LGCSSSSPTPPT....PQPHCSPPSPRNPATAGNKEREG.MA.P...............
**Pis12873**	....MA.ST..............
**Psat6g237920**	....................................IKT.KK.M...............
**Psat6g238360**	....MS.A...............
**gene-LOC116187009**	............LWIH........................LATAQ..............
**gene-LOC116187298**	....MAMAQ..............
**gene-LUZ62_030497**	....MG.EVSS............
**gene-LUZ62_045336**	....MG.EVSS............
**gene-LUZ62_075590**	....MG.EVSS............
**gene-SELMODRAFT_151616**	....MA.................
**gene-SELMODRAFT_444624**	....MA.................
**gene-G2W53_032365**	....MA.T...............
**gene-E2542_SST22125**	....MAPT...............
**gene-E2542_SST22126**	....
**gene-E2542_SST22127**	....
**Spipo29G0020600.v2**	....MA.PV..............
**Tp57577_TGAC_v2_gene30361**	....
**VducChr2G012310**	....MS.E...............
**vmacro00636_Vaccinium_macrocarpon_Stevens_v1**	....MG.................
**gene-HPP92_020855**	....
**gene-LOC114189846**	....MA.T...............
**gene-LOC114192130**	....MA.A...............
**gene-JRO89_XS14G0048500**	....ML.................

```
Medicago                                          ........................................AAS.................
gene-CEY00_Acc27669                               .......................................QSPF.................
Aspi01Gene46726                                   ............................................................
Azfi_s0030.g024403                                ............................................................
LXT01105                                          ........................................APS.................
LXT05484                                          ........................................APS.................
C.cajan_29159                                     ........................................LGS.................
C.cajan_29160                                     ........................................LSN.................
C.cajan_29161                                     ........................................TIT.................
C.cajan_48225                                     ........................................ASS.................
Cecan.1G005800.V3.1                               ........................................APS.................
Cecan.2G031800.V3.1                               ........................................VSS.................
Cecan.5G005100.V3.1                               ........................................APS.................
gene-LOC101500746                                 ........................................AAS.................
gene-LOC101501059                                 ........................................AAS.................
gene-IFM89_008902                                 .......................................RQNT.................
EUGRSUZ_J01152                                    .......................................MVPT.................
gene-GLYMA_10G292200v4                            ........................................PAS.................
gene-GLYMA_20G241500v4                            ..........................................T.................
gene-GLYMA_20G241600v4                            ........................................PAS.................
gene-LOC110425500                                 ........................................LPS.................
Lchi30763                                         ........................................SPL.................
Lchi33565                                         ........................................SPL.................
MARPO_0167s0012                                   ....................................KV.TSPAGQSQAAEFPKVENVSVS
Mvestita10057                                     SISGVTLLEVQQGAPLEAYEESKPAF.....ETC..KE.TKQT.................
gene-LOC11440741                                  ........................................APT.................
gene-LOC11424676                                  ........................................AFM.................
gene-LOC11440740                                  ........................................AAS.................
gene-MLD38_038120                                 .........................................PQ.................
gene-E3N88_23837                                  ..........................NDRSIHSDNG......S.................
gene-E3N88_23910                                  ........................................LLS.................
gene-E3N88_23911                                  ........................................LLS.................
gene-PHAVU_003G216600g                            ........................................PPS.................
gene-PHAVU_007G008600g                            ........................................APT.................
PIPE20159                                         ........................................LPA.................
Pis12873                                          .......................................ELPA.................
Psat6g237920                                      ........................................AAS.................
Psat6g238360                                      ........................................ASS.................
gene-LOC116187009                                 .......................................TTTV.................
gene-LOC116187298                                 .......................................TTAV.................
gene-LUZ62_030497                                 .....................................F.VLP..................
gene-LUZ62_045336                                 .....................................F.VLP..................
gene-LUZ62_075590                                 .....................................F.VLP..................
gene-SELMODRAFT_151616                            .........................................PELLKQG............
gene-SELMODRAFT_444624                            .........................................PELLKQG............
gene-G2W53_032365                                 ........................................ASS.................
gene-E2542_SST22125                               ........................................APT.................
gene-E2542_SST22126                               ............................................................
gene-E2542_SST22127                               ............................................................
Spipo29G0020600.v2                                .......................................EIPA.................
Tp57577_TGAC_v2_gene30361                         ........................................MSA.................
VducChr2G012310                                   .......................................SFSA.................
vmacro00636_Vaccinium_macrocarpon_Stevens_v1      .......................................RYLV.................
gene-HPP92_020855                                 ............................................................
gene-LOC114189846                                 ............................................................
gene-LOC114192130                                 ........................................APT.................
gene-JRO89_XS14G0048500                           ............................................................
```

```
                                                                                       10
                                                                                        .
Medicago                                            ..........................IT........AITV.ENLEYP.............
gene-CEY00_Acc27669                                 ..........................VT........EVQV.ENYVFP.............
Aspi01Gene46726                                     ....................................................MAFA.............
Azfi_s0030.g024403                                  ............................................................
LXT01105                                            ..........................IV........GVKV.EFIEFP.............
LXT05484                                            ..........................IA........GVKV.EFIEFP.............
C.cajan_29159                                       ..........................VT........GLEV.EDVTFP.............
C.cajan_29160                                       ..........................VT........GVKV.EFLEFP.............
C.cajan_29161                                       ..........................PD........PVQV.EFLQFP.............
C.cajan_48225                                       ..........................IT........AVQV.DNLHFP.............
Cecan.1G005800.V3.1                                 ..........................IT........GVKV.EFIEFP.............
Cecan.2G031800.V3.1                                 ..........................IT........GLKV.ENFEFP.............
Cecan.5G005100.V3.1                                 ..........................IT........GVKV.EFIEFP.............
gene-LOC101500746                                   ..........................IT........GMKV.EFLEFP.............
gene-LOC101501059                                   ..........................IT........GVKV.EFLEFP.............
gene-IFM89_008902                                   ..........................VT........EVKV.EDITFS.............
EUGRSUZ_J01152                                      ..........................VS........EVQV.ESFVFP.............
gene-GLYMA_10G292200v4                              ..........................IT........NVTV.EFLQFP.............
gene-GLYMA_20G241500v4                              ..........................IS........AVQV.EFLEFP.............
gene-GLYMA_20G241600v4                              ..........................IT........NVTV.EFLQFP.............
gene-LOC110425500                                   ..........................VT........QVDV.DNFVCP.............
Lchi30763                                           ..........................LA........SVQV.ESFVFP.............
Lchi33565                                           ..........................LA........SVQV.ESFVFP.............
MARPO_0167s0012                                     AEAEAQGKTLANGLHPKVENTEVSE.VS........EVTVVEGVQVT.............
Mvestita10057                                       ..........................LD........GLNI.EGVPFT.............
gene-LOC11440741                                    ..........................IT........GVKV.ENIEFP.............
gene-LOC11424676                                    ..........................IT........GVKV.EFIEFP.............
gene-LOC11440740                                    ..........................IT........AITV.ENLEYP.............
gene-MLD38_038120                                   ..........................VA........EIQV.ETVCFP.............
gene-E3N88_23837                                    .........................LISH.ENDNGGDSGV.ETIMFP.............
gene-E3N88_23910                                    ..........................ST........GVQV.ETITFP.............
gene-E3N88_23911                                    ..........................ST........GVQV.ETITFP.............
gene-PHAVU_003G216600g                              ..........................IT........SVTV.EFLQFP.............
gene-PHAVU_007G008600g                              ..........................IT........DVQV.EFLHFP.............
PIPE20159                                           ..........................VS........EVQV.EAAVFP.............
Pis12873                                            ..........................VS........KLQV.EAAVFP.............
Psat6g237920                                        ..........................IT........GITI.ENLEFP.............
Psat6g238360                                        ..........................IT........AIHV.ENIEFP.............
gene-LOC116187009                                   ..........................VT........GVQV.ENFSFP.............
gene-LOC116187298                                   ..........................VT........GVHV.ENFPFP.............
gene-LUZ62_030497                                   ..........................PT........PLVI.DGITFP.............
gene-LUZ62_045336                                   ..........................PT........PLVI.DGITFP.............
gene-LUZ62_075590                                   ..........................PT........PLVI.DGITFP.............
gene-SELMODRAFT_151616                              .....................................LVI.EGTQFP.............
gene-SELMODRAFT_444624                              .....................................LVI.EGTQFP.............
gene-G2W53_032365                                   ..........................LP........GVQV.EFIEFP.............
gene-E2542_SST22125                                 ..........................IT........GVHV.EFLEFP.............
gene-E2542_SST22126                                 ............................................................
gene-E2542_SST22127                                 ............................................................
Spipo29G0020600.v2                                  ..........................VS........ELDI.EGFVFP.............
Tp57577_TGAC_v2_gene30361                           ..........................IT........AIHV.ENIEFP.............
VducChr2G012310                                     ..........................VT........QVQV.DSHVFP.............
vmacro00636_Vaccinium_macrocarpon_Stevens_v1        .........................VLTQT......RLTIIEASKSPIERASEAPIDRRS
gene-HPP92_020855                                   ............................................................
gene-LOC114189846                                   ....................................PVTV.EFLEFP.............
gene-LOC114192130                                   ..........................IT........DVQV.EFLHFP.............
gene-JRO89_XS14G0048500                             ............................................................
```

```
                                                      20                                      30
Medicago                                            .A.......VV.TSP.V..........T.......G.KS....YFL.....GGA......
gene-CEY00_Acc27669                                 .P.......TV.KPP.G..........T.......T.KT....LFL.....GGA......
Aspi01Gene46726                                     .P.......SI.VAP.G..........S.......S.KS....LVL.....GGA......
Azfi_s0030.g024403                                  ............................................................
LXT01105                                            .A.......VV.TLP.G..........S.......N.KS....YFL.....GGA......
LXT05484                                            .A.......VV.TLP.G..........S.......N.KS....YFL.....GGA......
C.cajan_29159                                       .A.......VV.KAP.S..........S.......S.AT....FFL.....GGA......
C.cajan_29160                                       .A.......TV.TPP.G..........S.......T.KS....YFL.....GGA......
C.cajan_29161                                       .A.......LV.TSP.A..........S.......A.KT....YFL.....GGA......
C.cajan_48225                                       .P.......LL.SSS.V..........S.......A.KT....YFL.....GGA......
Cecan.1G005800.V3.1                                 .G.......VM.TLP.G..........S.......D.KS....YFL.....GGA......
Cecan.2G031800.V3.1                                 .G.......VV.TPL.S..........S.......S.KS....YFL.....GGA......
Cecan.5G005100.V3.1                                 .G.......VV.TLP.G..........S.......N.KS....YFL.....GGA......
gene-LOC101500746                                   .P.......VV.TSP.A..........T.......A.KS....YFL.....GGA......
gene-LOC101501059                                   .P.......VV.TSP.A..........T.......A.KS....YFL.....GGA......
gene-IFM89_008902                                   .PT......II.KPP.G..........S.......T.NT....FFL.....GGA......
EUGRSUZ_J01152                                      .P.......TA.KPP.G..........S.......G.NT....LFL.....SGA......
gene-GLYMA_10G292200v4                              .A.......VV.TPP.A..........S.......T.KS....YFL.....GGA......
gene-GLYMA_20G241500v4                              .A.......VV.TSP.A..........S.......G.KT....YFL.....GGA......
gene-GLYMA_20G241600v4                              .A.......LV.TPP.G..........S.......T.KS....YFL.....GGA......
gene-LOC110425500                                   .P.......SD.KPP.A..........S.......S.TV....LFL.....GGA......
Lchi30763                                           .P.......RV.KPP.G..........S.......S.KS....LFL.....GGA......
Lchi33565                                           .P.......RV.KPP.G..........S.......S.KT....LFL.....GGA......
MARPO_0167s0012                                     .S.......TI.LVP.G..........S.......D.SE....LSL.....VGA......
Mvestita10057                                       .P.......SV.VAP.G..........C.......S.KP....LVL.....GGA......
gene-LOC11440741                                    .A.......VV.TPP.A..........S.......P.KS....YFL.....GGA......
gene-LOC11424676                                    .A.......VV.TPP.S..........S.......T.KS....YFL.....GGA......
gene-LOC11440740                                    .A.......VV.TSP.V..........T.......G.KS....YFL.....GGA......
gene-MLD38_038120                                   .P.......YV.NP.......................................W......
gene-E3N88_23837                                    .P.......HV.KPP.G..........A.......T.NT....LFL.....GGA......
gene-E3N88_23910                                    .P.......HV.KPP.G..........A.......T.NT....LFL.....GGA......
gene-E3N88_23911                                    .P.......HV.KPP.G..........A.......T.NT....LFL.....GGA......
gene-PHAVU_003G216600g                              .A.......VV.TPP.S..........S.......T.KS....YFL.....GGA......
gene-PHAVU_007G008600g                              .A.......VV.TSP.A..........T.......A.KT....YFL.....GGA......
PIPE20159                                           .P.......SV.KPT.G..........S.......S.NT....LFL.....GGA......
Pis12873                                            .P.......TV.KPP.G..........S.......S.NT....LFL.....GGA......
Psat6g237920                                        .A.......VV.TPP.G..........S.......T.NS....YFL.....GGA......
Psat6g238360                                        .A.......VV.TSP.V..........T.......G.KS....YFL.....GGA......
gene-LOC116187009                                   .P.......AA.KPP.G..........S.......D.KT....LFL.....GGA......
gene-LOC116187298                                   .P.......AA.KPP.G..........S.......D.KT....LFL.....GGA......
gene-LUZ62_030497                                   .A.......FV.TPP.D..........S.......S.KP....FFL.....GGA......
gene-LUZ62_045336                                   .A.......FV.TPP.D..........S.......S.KP....FFL.....GGA......
gene-LUZ62_075590                                   .A.......FV.TPP.D..........S.......S.KP....FFL.....GGA......
gene-SELMODRAFT_151616                              .P.......GL.TSP.V..........S.......Q.KS....LFL.....AGA......
gene-SELMODRAFT_444624                              .P.......GL.TSP.V..........S.......Q.KS....LFL.....AGA......
gene-G2W53_032365                                   .A.......TI.TPPAG..........S.......V.KS....YFL.....GGA......
gene-E2542_SST22125                                 .A.......VI.TST.A..........S.......G.RT....YFL.....GGA......
gene-E2542_SST22126                                 ............................................................
gene-E2542_SST22127                                 ............................................................
Spipo29G0020600.v2                                  .P.......VVAKPP.G..........S.......S.KS....LFL.....GGA......
Tp57577_TGAC_v2_gene30361                           .A.......VI.TSP.V..........T.......G.KS....YFL.....GGA......
VducChr2G012310                                     .P.......TV.KPP.G..........T.......S.KT....LFL.....GGAVLGDIG
vmacro00636_Vaccinium_macrocarpon_Stevens_v1        SPMIGQPSDWI.KPK.GILLSIGQRRISADNMTHGSERSPHLETFLITRDSAGG......
gene-HPP92_020855                                   ............................................................
gene-LOC114189846                                   .A.......FV.TPP.S..........S.......T.KP....YFL.....GGA......
gene-LOC114192130                                   .A.......VV.TSP.A..........T.......A.KT....YFL.....GGA......
gene-JRO89_XS14G0048500                             .....................................................A......
```

```
                                                                                         40        50        60
                                                                                         .         .         .
Medicago                                    ........................GER.GLTI.EGNFI.KFTAIGVYL.EDIAVASLAAK
gene-CEY00_Acc27669                         ........................GVR.GLDT.EGEFV.KYTAIGVYL.EDKAIESLAVK
Aspi01Gene46726                             ........................GDR.GLEI.NGNFI.KFTAIGIYV.EEGVIPHLSPK
Azfi_s0030.g024403                          .............................MEI.NGNFI.KFTAIGIYV.DAAVVPHLAPK
LXT01105                                    ........................GER.GLVI.DGKFI.KFTAIGVYL.EDKAIPSLAVK
LXT05484                                    ........................GER.GLVI.DGKFI.KFTAIGVYL.EDKAIPSLAVK
C.cajan_29159                               ........................GIR.GLQS.EGNFI.KFTVIGFYL.QDNAIPFLSHK
C.cajan_29160                               ........................GVR.GRNF.NGEFV.KVTAVAVYL.EDKAVTSLAAT
C.cajan_29161                               ........................GER.GLMI.EGKFI.KFTAIGVYL.EDQAIQSLAPK
C.cajan_48225                               ........................GVR.GLMI.EGSFI.KFTGLGVYL.EDKAVASLATK
Cecan.1G005800.V3.1                         ........................GER.GLVI.DGNFI.KFTAIGVYL.EDKAIPSLALK
Cecan.2G031800.V3.1                         ........................GVR.GQNL.DGNFI.KFTTIGVYL.EDKAVPSLAAK
Cecan.5G005100.V3.1                         ........................GER.GLVI.DGNFI.KFTAIGVYL.EDKAIPSLALK
gene-LOC101500746                           ........................GER.GLTI.EGNFI.KFTAIGVYL.EDIAVASLATK
gene-LOC101501059                           ........................GVR.GLHI.DGEFV.KFTGLGVYL.EDKAVLSLAAN
gene-IFM89_008902                           ........................GVR.GLQI.QDKFI.KFTAIAVYLEEEKAIPFLALK
EUGRSUZ_J01152                              ........................GVR.GLEV.DGKYV.KYTAIGVYL.EATAVPILATK
gene-GLYMA_10G292200v4                      ........................GVR.GLNI.EEEFV.KFTGIGVYL.EDKAVSSLGAK
gene-GLYMA_20G241500v4                      ........................GER.GLTI.EGKFI.KFTGIGVYL.EDKAVPSLAAK
gene-GLYMA_20G241600v4                      ........................GVR.GLNI.QEEFV.KFTGIGVYL.EDKAVSSLAAK
gene-LOC110425500                           ........................GVR.DLYI.DGNFK.KFTAIGVYW.ELNTVPLLAAK
Lchi30763                                   ........................GVR.GLEI.EGKFV.KFTAIGVYL.EDDAVPLLAVK
Lchi33565                                   ........................GVR.GLEI.EGKFV.KFTAIGVYL.EDDAVPLLAVK
MARPO_0167s0012                             ........................GVR.GVRLPHGVEL.KVTVLGIYT.NLEVLNHLS.K
Mvestita10057                               ........................GFR.GLEI.NGTLI.KFTSVGIYL.DEEVISYLSPK
gene-LOC11440741                            ........................GVR.GLDI.DGEFV.KFTGIGIYL.EEKAVASLTPK
gene-LOC11424676                            ........................GVR.GLDV.DGEFV.KFTGLGVYL.EEKAVASLTLK
gene-LOC11440740                            ........................GER.GLTI.EGNFI.KFTAIGVYL.EDIAVASLAAK
gene-MLD38_038120                           ........................RFR.GIEI.PGKFL.KVTAIGFYL.EDGAVPFLAVK
gene-E3N88_23837                            ........................GVR.CVEI.ESKLV.KISGIGVYL.EDNAIQSLAAK
gene-E3N88_23910                            ........................GVR.CMEI.ESKLV.KISGIGVYL.EDKAIQSLAAT
gene-E3N88_23911                            ........................GVR.CVEI.ESKLV.KISGIGVYL.EDKAIQSLAAT
gene-PHAVU_003G216600g                      ........................GVR.GLNI.EGEFV.KFTGIGVYL.EEKAVAWLCAK
gene-PHAVU_007G008600g                      ........................GER.GLTI.EGKFI.KFTAIGVYL.EDKAVASLATK
PIPE20159                                   ........................GVR.GVVI.DGNFI.KVTAIGVYL.EVAALPLLAEK
Pis12873                                    ........................GAR.GVTI.QGNFI.KVTAIGVYL.EAAAVPLLAEK
Psat6g237920                                ........................GVR.GVFI.DGGFL.KATGISVYL.EESAISSLALK
Psat6g238360                                ........................GER.GLTI.NGTFI.KFTCIGVYL.EDKAVESLATK
gene-LOC116187009                           ........................GVR.GLDV.DGKFV.KYTAIGVYL.EEKALPLLATE
gene-LOC116187298                           ........................GVR.GLDV.DGKFV.KFTAIGVYL.EEKALPLLATK
gene-LUZ62_030497                           ........................GVR.GLDI.GGQFI.KFTAIAVYL.EESALQILAQK
gene-LUZ62_045336                           ........................GVR.GLDI.GGQFI.KFTAIAVYL.EESALQILAQK
gene-LUZ62_075590                           ........................GVR.GLDI.GGQFI.KFTVIAVYL.EESALQILAQK
gene-SELMODRAFT_151616                      ........................GVR.NIQI.NPQVTITVTVLGFYF.EDGILKHLSGK
gene-SELMODRAFT_444624                      ........................GVR.SIQI.NPQVTITVTVLGFYF.EDGILKHLSGK
gene-G2W53_032365                           ........................GER.GLVI.DGNFI.KFTAIGVYL.EDKALPLLASN
gene-E2542_SST22125                         ........................GER.GLTI.EGKFI.KFTAIGVYV.EDKAVASLATK
gene-E2542_SST22126                         ............................................................
gene-E2542_SST22127                         ..............................MV.EGNFI.KFTDLGVYL.EDKAVALLATK
Spipo29G0020600.v2                          ........................GVR.SLEI.QGKEI.KFSSIGVYV.EAEAVPSLAAK
Tp57577_TGAC_v2_gene30361                   ........................GER.GLTI.EGNFI.KFTAIGVYL.EDVAVASLATK
VducChr2G012310                             RDEAMGATGGNGSTYGDATGSAAVGVRSGLYL.EREFA.NITAIGVYL.EVNAIECLAVK
vmacro00636_Vaccinium_macrocarpon_Stevens_v1........................GDR......SGAFA.DPYKSGRYI...YV......T
gene-HPP92_020855                           ............................................................
gene-LOC114189846                           ........................GVR.GLNI.EGEFV.KFTGIGVYL.EEKAVAWLGSK
gene-LOC114192130                           ........................GER.GLTI.EGKFI.KFTAIGVYL.EDKAVASLAAK
gene-JRO89_XS14G0048500                     ........................GVR.DLEI.EGKLK.KFTAIGVYL.EDNAVPFLASK
```

```
                                                       70        80        90        100       110       120
                                                       .         .         .         .         .         .
Medicago                                               WKGKSSEELLETLDFY.RDIISGPFE.KLIRGSKIRELSGPEYSRKVMENCVAHLKSVGT
gene-CEY00_Acc27669                                    WKGKTEEELMDSIDFF.SDIVTGPFE.KFTHVKMIRHLTGKEFVRKVGENCLAYWKSSGI
Aspi01Gene46726                                        LNGKTVEELCDKELLF.EELISAPFE.KFVRVVFLVPLSGPQYSEKVLERIGVQ....AL
Azfi_s0030.g024403                                     LAGKSVDELCEKELIF.EELLIAPFE.KFVRVVFLVPLTGIQYSEKVIERIGVQ....AY
LXT01105                                               WNGKSADDLFNSLDFF.RDIISGPFE.KLIRGSKILPLSGPEYVKKVSENCVDHLKSVGT
LXT05484                                               WNGKSADDLFNSLDFF.RDIISGPFE.KLIRGSKILPLSGPEYVKKVSENCVDHLKSVGT
C.cajan_29159                                          WNALSASQLTQSLQFF.RDIIVGPFE.KFIKVTVMIPMTGKQYSEKVSENCMEIWKSLGI
C.cajan_29160                                          WKGKSAAELLESLDFY.RDIIKGPFE.KLIRVSNIRKLEGSEYVRKVSKNCMVHMKSVGT
C.cajan_29161                                          WKGNPSQQLLDNLHFF.RDIISGPFE.KLIRGSKILPLSGREYSRKVMENCVAHMKSVGT
C.cajan_48225                                          WKSKDSEGLVETLDFF.RDIISGGFE.KMIRCSYIRELSGREYSRKVMENCVAHMKSVGT
Cecan.1G005800.V3.1                                    WKGKSADELFKSLDFF.RDIISGPFE.KLIRGSKILPLSGPEYVKKVSENCVAHLKSVGT
Cecan.2G031800.V3.1                                    WKGKTENELLESLDFF.RDIIAGPYE.KLIRGTKILPLLGKEYSRKVSENCVAHLKSVGT
Cecan.5G005100.V3.1                                    WKEKSADELFKSLDFF.RDIISGPFE.KLIRGSKILPLSGPEYVKKVSENCVAHLKSVGT
gene-LOC101500746                                      WKGKTSQQLLQTLDFY.RDIISGPFE.KLIRGSKIRELSGPEYSRKVMENCVAHLKSIGT
gene-LOC101501059                                      WKDKTPAQLLESLDFY.RDIVKGPFE.KFIRGTKVRKLEGPEYVRKVSENCIAHMKAQGT
gene-IFM89_008902                                      WKGKTVDELENSDDFY.LDIITGPFE.KIVLVKMISQLMGQQYAMGLTENCIKFWKTLGN
EUGRSUZ_J01152                                         WKGKTADELRDSIEFF.RDVITGPFE.KLTQVSFITLLTGPQYTNKVTENCIAFWNCNGG
gene-GLYMA_10G292200v4                                 WKGKSAAELLDSLDFY.RDIIKGPFE.KLIRGSKLRTLDGREYVRKVSENCVAHMESVGT
gene-GLYMA_20G241500v4                                 WKGKTSEELVHTLHFY.RDIISGPFE.KLIRGSKILPLAGAEYSKKVMENCVAHMKSVGT
gene-GLYMA_20G241600v4                                 WKGKSAAELLDSLDFY.RDIIKGPFE.KLIRGSKLRTLDGREYVRKVSENCVAHMQSVGT
gene-LOC110425500                                      WRGKTAQELMDSVKFF.RDIVTGAFE.KFIRVTKVLTLTSQQYSEKVAENCVAIWKSLEL
Lchi30763                                              WKGKTAQELMDSVEFF.RDIVTGPFE.KFTRVTLISTLTGQQYSEKVAENCVAFWKAVGS
Lchi33565                                              WKGKTAQELMDSVEFF.RDIVTGPFE.KFTRVTLISTLTGQQYSEKVAENCVAFWKAVGS
MARPO_0167s0012                                        YDGLSAEELLKDDQFL.LAFLEAPVD.KLARVTMLLPLTGEDYAGKSGDTTEMSLKEVNK
Mvestita10057                                          LKGKSVDELCDEELLF.EELLAAPFE.KFVRVVFLVPLTGIQYSEKVLERISVQ....AL
gene-LOC11440741                                       WKGKTPSQLFESLEFY.RDIIKGPFE.KFIRSTKVRTLEGSEYVRKVSENCIAHMKSEGT
gene-LOC11424676                                       WKGKTPSQLFESLDFY.RDIIKGPFE.KFIRCTKVRTLEGSEYVRKVSENCIAHMKFEGT
gene-LOC11440740                                       WKGKTSQELLDTLDFY.RDIISGPFE.KLIRGSKIRELSGPEYSRKVMENCVAHLKSVGT
gene-MLD38_038120                                      WKGKAAEELTESEEFI.TDVIAAPYE.RFLRVTMLLPLTGVQYSEKVTENCVAYWKSIGK
gene-E3N88_23837                                       WKGKGIEELQDSEDFF.KDIINGPFE.KFIQVTAIIPLTGTQFSQKVSAYCDRIWKAQGT
gene-E3N88_23910                                       WKGKGVEELQDSEDFF.KDIINGPFE.KFIQVTAIIPLAGKQFSKNVSAYCDRIWKAQGT
gene-E3N88_23911                                       WKGKGVEELQDLEDFF.KDIINGPFE.KFIQVTAIIPLAGKQFSKNVSAYCDRIWKAQGT
gene-PHAVU_003G216600g                                 WKGKNAAELLESLEFY.RDIIKGPFE.KYIRGSKLRTLDGPEYVRKVSENCVIFMKSVGS
gene-PHAVU_007G008600g                                 WKGKPSEELINTLDFY.RDIISGPFE.KLIRGSKILQLSGTEYSRKVMENCVAHLKSVGT
PIPE20159                                              WKGKTADELEGSLEFF.RDIVTGPYE.KFTRVTLLVPLPAHLYSDKVSEACVAIWKAAGI
Pis12873                                               WKGKTAEELDGSLEFF.REIVTGPYE.KFTRVTMLLPLTGVQYSEKVTENCVGIWKAAGI
Psat6g237920                                           WKGKTEAELLESLDFY.RDIIKGPFE.KLIRGGKIITLDGREYVRKVSENCIAFMKSDGT
Psat6g238360                                           WKGKTSEELLETLDFY.RDIISGPFE.KLIRGSKIKELSGPEYSRKVMENCVAHLKSVGT
gene-LOC116187009                                      WKGKSAEELTDTDEFF.RDIITGPFE.KLTQVSFITQLTGKQYSDKVAENCIAFWKSIGA
gene-LOC116187298                                      WKGKSAEELTDTDEFF.RDIITGPFE.KLTQVSFITQLTGKQYSDKVSENCIAFWKSIDA
gene-LUZ62_030497                                      WSSNTADELTGSLEFY.RDIINGPFN.......YLR........................
gene-LUZ62_045336                                      WSSNTADELTGSLEFY.RDIINGPFK.KFYQIAFLRHLTGEQYTDKVIENSVNHWKVVGI
gene-LUZ62_075590                                      WSSNTADELTGSLEFY.RDIINGPFK.KFYQIAFLRHLTGEQYTDKVIENSVNHWKVVGI
gene-SELMODRAFT_151616                                 WSGKSGSELEKEDDFF.TDIINGMYDTKIFSVTMLKPLPGTDFSKKVMENTKQVLAESNS
gene-SELMODRAFT_444624                                 WSGKSGSELEKEDDFF.TDIINAPSE.KIFSVTMLKPLPGTEFSKKVMENTKQVLAESNS
gene-G2W53_032365                                      WKGKSEDELSKSLQFF.RDIITGPYE.KLIRGSKILPLSGEEYVKKVSENCVGHLKSVGS
gene-E2542_SST22125                                    WKGKASEELAETLDFF.RDIISGPFE.KLVRGSKILPLSGPEYSRKVMENCVAHLKSVGT
gene-E2542_SST22126                                    ......................................................MKSVGT
gene-E2542_SST22127                                    WKGKASEELPETLDFF.KDIISGPFK.KLIRCSKIRTLSDPEYPRKVMENCVAPMKSVGT
Spipo29G0020600.v2                                     WKEKTAEELANSVEFF.RDIVTGPFE.KFIRVTMIQPLTGQQYSEKVAENCVAYWKSIGI
Tp57577_TGAC_v2_gene30361                              WKGKSSEELLETLDFY.RDIISGPFE.KLIRGSKIRELSGPEYSRKVTENCVAHLKSVGT
VducChr2G012310                                        WKGKTAEELTDSIDFF.RDIVTGPFE.KFIHVKMILHLPGMIYVEKVKENCLAYWKATGT
vmacro00636_Vaccinium_macrocarpon_Stevens_v1           WI......LFDKSHIYIDEFLIRPFE.KFILVKMIRHLTGMIYVEKMKENCLAYWNATGT
gene-HPP92_020855                                      ............................................................
gene-LOC114189846                                      WKGKSAAELVESLEFY.RDIIKGPFE.KFIRGSKVRTLEGPEYVRKVSENCVNFMKSDGN
gene-LOC114192130                                      WKGKPSEELIETLDFY.RDIISGPFE.KLIRGSKILPLSGPEYSKKVMENCVAHLKTVGT
gene-JRO89_XS14G0048500                                WKGKTVEELTDSVEFF.RDIVTGPFE.KFTQVSFISPLTGIQYTGKVVENCIAFWKSVGV
```

```
                                                          130       140       150       160
                                                            .         .         .         .
Medicago                                            YGDAEAEA.MQKFAEAFKPVNFPPGASVFYRQSPDGIL...........G..........
gene-CEY00_Acc27669                                 HNAAAGEA.LERFTKVFEDEYCPPGAAVLFTQSPYGSI...........T..........
Aspi01Gene46726                                     YTKLQDEH.KQQFLEIFKAESFPPRSSVLLSFSKEG.L...........K..........
Azfi_s0030.g024403                                  TTKVQEEH.KQKFLEIFKAENFPPRASVLFSFSKEG.L...........K..........
LXT01105                                            YTDAEAKA.IEKFIEAFKDQTFPPGSSVFYRQSPAGTL...........T..........
LXT05484                                            YTDAEAKA.IEKFIEAFKDQTFPPGSSVFYRQSPAGTL...........T..........
C.cajan_29159                                       YTDAEGRA.IEKFVSVFKDQTFPPGSSMLFTFSHKGSL...........A..........
C.cajan_29160                                       YSDAEEKA.IEELRNAFSVHNFPPSASVFYKQSPTGTL...........G..........
C.cajan_29161                                       YGEAEAAA.IEKFAQAFQNVNFAAGASVFYRQSPHGIL...........G..........
C.cajan_48225                                       YGEAEAAA.IERFAEAFKSVNFPPGASVFFRQSPDGIL...........G..........
Cecan.1G005800.V3.1                                 YTDADAKA.IKKFVEAFKDQTFPPGSSVFYRQSPAGTL...........T..........
Cecan.2G031800.V3.1                                 YSDAEAIA.IEKFIEAFKDHAFPPGSSVFYKQSPNGSL...........T..........
Cecan.5G005100.V3.1                                 YTDAEAKA.IEKFVEAFKDQTFPPGSSVFYRQSPAGTL...........T..........
gene-LOC101500746                                   YGDEEVEA.MQKFAEAFKNVNFPPGASVFYRQSPDGVL...........G..........
gene-LOC101501059                                   YGDAEEKA.IQEFREAYKDQTFPPGATVFYRQSPNGTL...........G..........
gene-IFM89_008902                                   YTESKDKGLLEELIQVFQDEIFPPNSTILFTQSS.PTL...........T..........
EUGRSUZ_J01152                                      YKQEEAEA.IDELIEVFKDQTFPPGSSIFFTHLTNGSY...........V..........
gene-GLYMA_10G292200v4                              YSEAEEKA.IEEFRNAFKDQNFPPGSTVFYKQSPTGTL...........G..........
gene-GLYMA_20G241500v4                              YGDAEAAA.IEKFAEAFKNVNFAPGASVFYRQSPDGIL...........G..........
gene-GLYMA_20G241600v4                              YSDEEEKA.IEEFRNAFKDQNFPPGSTVFYKQSPTGTL...........G..........
gene-LOC110425500                                   YTDAEAKA.IEKFLEFFKDEN..SDSSTLFTLSAHGSL...........T..........
Lchi30763                                           FTDAEATA.VEKLKEVFQDRTFPPGASILFTHSPLGSF...........T..........
Lchi33565                                           FTDAEATA.VEKLKEVFQDRTFPPGASILFTHSPLGSF...........T..........
MARPO_0167s0012                                     WGEAEELA.LKEYREFFKQKTCPPGSTIFFAVTKSG.L...........E..........
Mvestita10057                                       YTNIQDEH.KQKFLEVFKPESFPPRSSVLLSFSKEG.L...........K..........
gene-LOC11440741                                    YGDAEEKA.IQEFREAFKDQFFPPGTAAFYRQSPNGAL...........G..........
gene-LOC11424676                                    YGDAEEKA.IQELREAFKVQFFPPGAGVFFRQSPNGAL...........G..........
gene-LOC11440740                                    YGDAEAEA.MQKFAEAFKPINFPPGASVFYRQSPDGIL...........G..........
gene-MLD38_038120                                   YTDTEAAA.VMEFLETFKDKTFAPGC....V.......................
gene-E3N88_23837                                    YNHKDAKT.IDKFNEVFKDQNFSPGSSILLTISNAGSL...........T..........
gene-E3N88_23910                                    YNHEDAKT.IEKFNEVFKDQNFSPGSSILLTISTAGSL...........T..........
gene-E3N88_23911                                    YNHEDAKT.IEKFNEVFKDQNFSPGSSILLTISTAGSL...........T..........
gene-PHAVU_003G216600g                              YGEAEEKA.IEEFRYAFKDQNFPPGSTVFYRQSPTGTL...........G..........
gene-PHAVU_007G008600g                              YGDAEAKG.IEEFAEAFKKVNFPPGASVFYRQSPDGIL...........G..........
PIPE20159                                           YTDAEADA.VDKFKAAFKEEGFAPGNSILFTQSPSGTL...........T..........
Pis12873                                            YTDAEADA.VEKFKAAFKDESFGPGDSILFTQSPLGTL...........T..........
Psat6g237920                                        YNDEEEKA.IEKFRQAFQEQHFPPGSSVFYKQSPNGTL...........G..........
Psat6g238360                                        YGDAEVEA.IQKFAEAFKNVNFPPGASVFYRQSPDGIL...........G..........
gene-LOC116187009                                   YGASEARA.IDWFLDVFKDQSFPPGSSIFFTHLSNGSYVERDGISLHRVQ..........
gene-LOC116187298                                   YGESEAGA.IDRFLHVFKDQCFPPGSCIFFTHLSNGSY...........V..........
gene-LUZ62_030497                                   .........................SSI...KLPS..............Y..........
gene-LUZ62_045336                                   SVEKEAEA.VDKFKDVFKPETFPPGTSIFFTQCPSGSI...........S..........
gene-LUZ62_075590                                   SVEKEAEA.VDKFKDVFKPETFPPGTSIFFTQCPSGSI...........S..........
gene-SELMODRAFT_151616                              LGEDEEKA.IEEFSKLFEDQALKPGMGPFYVSSSSG.L...........G..........
gene-SELMODRAFT_444624                              LGEDEEKA.IEEFSKLFEDQALKPGMGPFYVSSSSG.L...........G..........
gene-G2W53_032365                                   YDEEEAKA.IEEMIQAFKGHHFKPGSSVFYKQSPHGTL...........T..........
gene-E2542_SST22125                                 YGDAEAAA.IEKFAQAFKNVNFPPGGSVFYRQSPDGIL...........G..........
gene-E2542_SST22126                                 YGDAEATA.TEKFAQAFKNMNFPPGAFVFFRQSPDGIL...........GVSNTDFVIIL
gene-E2542_SST22127                                 YGDAEAAA.IEKFTQAFKNMNFPPGASVFFRQSPDGIL...........G..........
Spipo29G0020600.v2                                  YTDAEAEA.VEKLLDTFKEETFPPGSSILFTQSPSGSL...........T..........
Tp57577_TGAC_v2_gene30361                           YGDAEVEA.MEKFVEAFKPINFPPGASVFYRQSPDGIL...........G..........
VducChr2G012310                                     YNDAAGEA.LENFKKVFNGEDCPPGAAILFTQFPRGSI...........T..........
vmacro00636_Vaccinium_macrocarpon_Stevens_v1        YNDATVEA.LENGFQR..........................................
gene-HPP92_020855                                   ..................MPSDSSIFFTHSPQGSL...........T..........
gene-LOC114189846                                   YGEAEEKA.IEEFRYAFKDQHFPPGSTVFYRQSPTGTL...........G..........
gene-LOC114192130                                   YGDAEAAA.IEQFAEAFKPVNFPPGASVFYRQSPDGIL...........G..........
gene-JRO89_XS14G0048500                             YTEAQENA.IEELLAVFKNENFPTGTSLFFTHLPQDSF...........T..........
```

```
                                                                     170       180
Medicago                                      ...........LSFSP.....DTSIPEKEAALIENKAVSSAV................LE
gene-CEY00_Acc27669                           ...........ISFSK.....DGLLPETGKAVIENKQVSETV................LQ
Aspi01Gene46726                               ...........VAFTK.....GNDIPEKPVAVIEDEAFGEAV................LA
Azfi_s0030.g024403                            ...........IAFTK.....DNSIPEKPIAVIEDESFATAV................LA
LXT01105                                      ...........LSFSK.....DGAIPADEGVVIENKPVSEAV................LE
LXT05484                                      ...........LSFSK.....DGAIPADEGVVIENKPVSEAV................LE
C.cajan_29159                                 ...........ITFSQ.....DHSIPRVAAIVIQNKQLSEAV................LE
C.cajan_29160                                 ...........LSFSK.....DETMPEDEYAVIDNLALSEAI................LE
C.cajan_29161                                 ...........LSFSE.....DGTLPGKEGAVIENKALSEAV................LE
C.cajan_48225                                 ...........LSFSQ.....DETLPANEDAVIENKALSEAV................LE
Cecan.1G005800.V3.1                           ...........LSFSK.....DGTITEDDGVVIENKPVSEAV................LE
Cecan.2G031800.V3.1                           ...........ISFSQ.....DGKIPKEEVVIIHNKPLSEAV................LE
Cecan.5G005100.V3.1                           ...........LSFSK.....DGTIAEDDGVVIENKPVSEAV................LE
gene-LOC101500746                             ...........LSFSK.....DASIPEKEDAVIENKAVSSAV................LE
gene-LOC101501059                             ...........LSFSK.....DETIPEHEYAVFNNKPLSEAV................LE
gene-IFM89_008902                             ...........IGFSE.....DGSIPKEGKANIENKRLSEAI................LG
EUGRSUZ_J01152                                ...........ISFSD.....HNRIPEIGNAVIDNNELAGTV................LE
gene-GLYMA_10G292200v4                        ...........LSFSK.....DETIPEHEHAVIDNKPLSEAV................LE
gene-GLYMA_20G241500v4                        ...........LSFSE.....DATIPEKEAAVIENKAVSAAV................LE
gene-GLYMA_20G241600v4                        ..........QLSFSK.....DETIPEHEHAVIDNKPLSEAV................LE
gene-LOC110425500                             ...........ISFSK.....DSSVPQVGIAVIENKLLANAV................LE
Lchi30763                                     ...........IGFSE.....HSSIPEQGNAVIESKQLMETV................LE
Lchi33565                                     ...........IGFSE.....HSSIPEQGNAVIESKQLMETV................LE
MARPO_0167s0012                               ...........ISQSF.....DSSIPKKAEYVVKNPVFGAGL................VG
Mvestita10057                                 ...........IAFTR.....DDSIPETPIATIEDETFAASV................LA
gene-LOC11440741                              ...........LRFSK.....DETIPEHEYAVINNKPLSEAV................LE
gene-LOC11424676                              ...........LRFSK.....DETIPEHEYVVINNKPLSEAV................LE
gene-LOC11440740                              ...........LSFSP.....DTSIPEKEAALIENKAVSSAV................LE
gene-MLD38_038120                             .............YP...........................................LH
gene-E3N88_23837                              ...........ISFSK.....DGGIQDTMISVIENEKIGPAI................IE
gene-E3N88_23910                              ...........ISFSK.....DGSIQDTMVSVLENEKIGPAM................IA
gene-E3N88_23911                              ...........ISFSK.....DGSIQDTMVSVLENEKIGPAM................IA
gene-PHAVU_003G216600g                        ...........LSFSK.....DDTIPENEYVVIENKALSEAV................LE
gene-PHAVU_007G008600g                        ...........LSFSE.....DATIPGEEAVVIENKAVSAAV................LE
PIPE20159                                     ...........IAFSD.....DGSVPEAGKAVIANKPMSETV................LE
Pis12873                                      ...........IGFSK.....DSSVPEAGKTVIANKPISETV................LD
Psat6g237920                                  ...........LSFSK.....DGTIPEDEHSVIDNKPLSESV................LE
Psat6g238360                                  ...........LSFSK.....DISIPEKEDAIIENKAASSAV................LE
gene-LOC116187009                             ...........ISFSK.....HESIPEVGNAVIENNELAEAV................LE
gene-LOC116187298                             ...........ISFSK.....HESIPEVGNAVIENNELAEAV................LE
gene-LUZ62_030497                             ...........ITFSK.....DDSVPETNNAVIENTALSYTV................LE
gene-LUZ62_045336                             ...........IAFSK.....DDSVPETNNAVIENTALSYTV................LE
gene-LUZ62_075590                             ...........IAFSK.....DDSVPETNNAVIENTALSYTV................LE
gene-SELMODRAFT_151616                        ...........VGFTDP....DEPSNLKISSSIGNVKFANAL................LS
gene-SELMODRAFT_444624                        ...........VGFTDP....DEPSNLKISSSIGNVKFANAL................LS
gene-G2W53_032365                             ...........ISFSE.....DATLPEDEGVVIKNKAASMAV................LE
gene-E2542_SST22125                           ...........LSFSE.....DATLPGKEAAVIENKAVSEAV................LE
gene-E2542_SST22126                           ISVSVLFLKPRISFCQ.....DGTIPGNEGAVGENKAVSEAV................LE
gene-E2542_SST22127                           ...........VSVSNYVSYFNGTIPGNECAVIENKVVSEPV................LE
Spipo29G0020600.v2                            ...........IAFSK.....DSTLPESGKAVIKNLAMSQAF................LT
Tp57577_TGAC_v2_gene30361                     ...........LSFSQ.....DASIPEKEAAVIENKAASSAV................LE
VducChr2G012310                               ...........ISFSK.....DDSIPETEKSVIENREVSETI................LE
vmacro00636_Vaccinium_macrocarpon_Stevens_v1  .............FSK.....DDSIPETGKSVIENKEVSETV................LE
gene-HPP92_020855                             ...........IGFSE.....DGTVPETAVGVIENEKLSNEVPHDCYNNNLILHIHKDKN
gene-LOC114189846                             ...........LSFSK.....DETIPENEYAVIENKALSEAV................LE
gene-LOC114192130                             ...........LSFSE.....DASIPGEEAAVIENKAVSAAV................LE
gene-JRO89_XS14G0048500                       ...........ISFSK.....DTSIPEIGNAEIKSRDLSETV................LE
```

	190 200 210 220
Medicago	TMIGEHAV...SPDLK.....RCLAARLPALLNEGAFKI..GN.................
gene-CEY00_Acc27669	TIIGKNGV...SPAAK.....QSLAARISQFLNKYSA.......................
Aspi01Gene46726	TIIWKEGV...SPGAK.....VSLAERLSKYF............................
Azfi_s0030.g024403	TIIWKDGV...SPAAK.....VSLAERLSKHFT...........................
LXT01105	TMIGKNAV...SPALK.....QNLALRYLELLNAQTNFN..IKN................
LXT05484	TMIGKNAV...SPALK.....QNLALRYLELLNAQTNFN..IKN................
C.cajan_29159	TVLGNDSI...SPQLK.....HSLASRLSHFFKQN.........................
C.cajan_29160	TMIGEKPV...SPALK.....ESLASRFCEFLKEANPNT..ETHN...............
C.cajan_29161	TMIGEHAV...SPDLK.....RSLASRLPALLDQQGIKI.....................
C.cajan_48225	TMIGEHPV...SPALK.....HSLASRLPALFNHGFIVI.....................
Cecan.1G005800.V3.1	TMIGKDAV...SPALK.....QNLAVRYLELLKVPTNFN..IQN................
Cecan.2G031800.V3.1	TMIGKHPV...SPALK.....HSLASRLSKLLGEGQSQ......................
Cecan.5G005100.V3.1	TMIGKDAV...SPALK.....QNLAVRYLELLKVLTNFN..IQN................
gene-LOC101500746	TMIGEHAV...SPDLK.....RCLAARLPALFDQGTFSI..GN.................
gene-LOC101501059	TMIGEIPV...SPALK.....ESLATRLYEFMKNDNFTI..GN.................
gene-IFM89_008902	TVLCKGGV...SPAAR.....RSLAVRLCDLFKVEEQKA..AKC................
EUGRSUZ_J01152	TIVGKNGV...SPAAR.....HSLGCRLAQLMKESIQRN..DKDGTNGK....SEHFSS.
gene-GLYMA_10G292200v4	TMIGEIPV...SPALK.....ESLATRFHQFFKELEANP..NNEN...............
gene-GLYMA_20G241500v4	TMIGEHAV...SPDLK.....RSLASRLPAVLSHGIIV......................
gene-GLYMA_20G241600v4	TMIGEIPV...SPALK.....ESLATRFHQFFKELEANP..NIEN...............
gene-LOC110425500	TIIGKDGV...SPVSR.....KSLASRLSALFDDSGEKA..AQDGKPESQW.........
Lchi30763	TIIGQNGV...SPAAK.....QSLAMRVSEFLKEFDPER..AHKEG..............
Lchi33565	TIIGQNGV...SPAAK.....QSLAMRVSEFLKEFDPER..AHKEG..............
MARPO_0167s0012	TMLSVKGV...SPHTR.....AKFGENMSTLLKNKIKDS..SEVVANGAS..........
Mvestita10057	TIIHKEGV...SPAAK.....VSLAERLCKHF............................
gene-LOC11440741	TMIGEIPV...SPALK.....ESLATRFYEFLKIDNFNI..RN.................
gene-LOC11424676	TMIGEIPV...SPALK.....ESLATRFYEFMKIDNFNI.....................
gene-LOC11440740	TMIGEHAV...SPDLK.....RCLAARLPALLNEGAFKI..GN.................
gene-MLD38_038120	TV...................................A......................
gene-E3N88_23837	TAIGKHGF...SPEAK.....KSVASRLSTIIN...........................
gene-E3N88_23910	TAIGKHGV...SPEAK.....KSVASRLSSIIN...........................
gene-E3N88_23911	TAIGKHGV...SPEAK.....KSVASRLSSIIN...........................
gene-PHAVU_003G216600g	TMIGEIPV...SPALK.....ESLATRFYEFLKEDNSNT..E..................
gene-PHAVU_007G008600g	TMIGEHAV...SPDLK.....RSLASRLPAVLNGGIIV......................
PIPE20159	TIIGENGV...SPAAK.....KSLAARVSQLLGELDAVQ..PEKTQEAEKK..QEQPEEA
Pis12873	TIIGENGV...SPEAR.....KSLAARISQLLGDLDKTA..EV.................
Psat6g237920	TMIGEIPV...SPALK.....ESLATRIHEFLKIENFNI..GN.................
Psat6g238360	TMIGEHAV...SPDLK.....RCLAARLPALLNEGTFKI..GN.................
gene-LOC116187009	TIIGNGGV...SPAAR.....KSLATRLAEFMKLEKID......................
gene-LOC116187298	TIIGNGGV...SPAAR.....KSLATRLAEFMKLEKIS......................
gene-LUZ62_030497	TIIGQIPV...SPAAK.....RSLAVRFSEHFKLLSTTI..SQAKEQMEVPSPVTQPLE.
gene-LUZ62_045336	TIIGEIPV...SPAAK.....RSLAVRFSEHFKLLSTTI..SQAKEQMEVPSPVTQPLE.
gene-LUZ62_075590	TIIGQIPV...SPAAK.....RSLAVRFSEHFKLLSTTI..SQAKEQMEVPR..VSPNH.
gene-SELMODRAFT_151616	TMIGKNPV...SPASK.....ACIAQRLSALL............................
gene-SELMODRAFT_444624	TMIGKNPV...SPASK.....ACIAQRLSALL............................
gene-G2W53_032365	TMIGH..........................L............................
gene-E2542_SST22125	TMIGEHAV...SPDLK.....RSLASRLPKVLNEGIVV......................
gene-E2542_SST22126	TMIGEHIV...SSALK.....RSLASRLPALLNDGIIII.....................
gene-E2542_SST22127	TMIGEPAV...SPALK.....RNLASRLPALLNDGIVIT.....................
Spipo29G0020600.v2	TIVGQHGV...SPAAK.....KSLASRISELLKSVNEGE..NGHVE.......ATPPPEN
Tp57577_TGAC_v2_gene30361	TMIGEHAV...SPDLK.....RCLAARLPALLNEGTFKI.....................
VducChr2G012310	TIIGKNGV...SPAAK.....QSLATRLCELLNNYEA.......................
vmacro00636_Vaccinium_macrocarpon_Stevens_v1	TIIGKNGV...SPAAK.....QSLATRLSELRNNNEA.......................
gene-HPP92_020855	TIINANCFAPGSPTAQPPLDIESISWSLGRPLDDANGTSLPASSA...............
gene-LOC114189846	TMIGEIPV...SPALK.....ESLATRFYEFLKEDNSKT..E..................
gene-LOC114192130	TMIGEHAV...SPDLK.....RSLASRLPAVLNDGIIV......................
gene-JRO89_XS14G0048500	TIIGKNGV...SPAAR.....QSLAARLSELMKD..........................

**Medicago**	....
**gene-CEY00_Acc27669**	....
**Aspi01Gene46726**	....
**Azfi_s0030.g024403**	V
**LXT01105**	....
**LXT05484**	....
**C.cajan_29159**	....
**C.cajan_29160**	....
**C.cajan_29161**	....
**C.cajan_48225**	....
**Cecan.1G005800.V3.1**	....
**Cecan.2G031800.V3.1**	....
**Cecan.5G005100.V3.1**	....
**gene-LOC101500746**	....
**gene-LOC101501059**	....
**gene-IFM89_008902**	....
**EUGRSUZ_J01152**	....
**gene-GLYMA_10G292200v4**	....
**gene-GLYMA_20G241500v4**	....
**gene-GLYMA_20G241600v4**	....
**gene-LOC110425500**	...FRVSCLSDQ..........LIWLPP....RGIPR.
**Lchi30763**	.............E........................
**Lchi33565**	.............E........................
**MARPO_0167s0012**	....
**Mvestita10057**	....
**gene-LOC11440741**	....
**gene-LOC11424676**	....
**gene-LOC11440740**	....
**gene-MLD38_038120**	....
**gene-E3N88_23837**	....
**gene-E3N88_23910**	....
**gene-E3N88_23911**	....
**gene-PHAVU_003G216600g**	....
**gene-PHAVU_007G008600g**	....
**PIPE20159**	Q..KEIK...............................
**Pis12873**	..........................PK..........
**Psat6g237920**	....
**Psat6g238360**	....
**gene-LOC116187009**	.....................................F
**gene-LOC116187298**	V
**gene-LUZ62_030497**	.AALKLN...............................
**gene-LUZ62_045336**	.AALKLN...............................
**gene-LUZ62_075590**	.LKLLLN...............................
**gene-SELMODRAFT_151616**	....
**gene-SELMODRAFT_444624**	....
**gene-G2W53_032365**	....
**gene-E2542_SST22125**	....
**gene-E2542_SST22126**	....
**gene-E2542_SST22127**	....
**Spipo29G0020600.v2**	...GHVEAATPPPEDGTTPEAP....PEKTSQ......
**Tp57577_TGAC_v2_gene30361**	....
**VducChr2G012310**	....
**vmacro00636_Vaccinium_macrocarpon_Stevens_v1**	....
**gene-HPP92_020855**	....
**gene-LOC114189846**	....
**gene-LOC114192130**	....
**gene-JRO89_XS14G0048500**	....
