# Supplementary material for: Evolutionary landscape of plant chalcone isomerase-fold gene families
Source: Front Plant Sci. 2025 Mar 28;16:1559547. doi: 10.3389/fpls.2025.1559547 (PMC11985768; doi:10.3389/fpls.2025.1559547)
Supplement: Supplementary Table 1 — The genomic data source of 259 species. [file DataSheet1.zip › Supplementary_information/Figure S5.pdf]

```
                                                             1
Medicago                           .............................M..................AAS..IT.....
AH009939.v2.1                      ..........................MT.A..................STL..FT.....
BjuB05g27840S                      ...................MSSSNC.PS.L..................LPT..VT.....
BnaAnng08210D                      ...................MFSSGS.QL.P..................LPS..VT.....
BnaC07g45760D                      ...................MFSSGS.QL.Q..................LPS..VT.....
BnaCnng45660D                      ...................MSSSHC.PT.P..................LPS..VT.....
Bo7g117570                         ...................MFSSGS.QL.Q..................LPS..VT.....
Csa06g029660                       M................SSSSVVVS.PS.P..................LPT..VA.....
Csa09g057890                       M................SSSSVVVS.PS.P..................LPT..VA.....
Csa09g065990                       M................SSSSVVVS.PS.P..................LPT..VA.....
gene-LOC110809010                  .......................MS.SS.S..................LPS..VA.....
Cspi12547                          ..........................MA...................NSAI..VS.....
CRGY0216793                        MQLG......................MA.SKS.............QSSSPTNHLAAKGEN
gene-GSCOC_T00026625001            ..........................MA.P..................AFC..VS.....
gene-BT93_J0013                    ..........................MS.PL................MVPP..VS.....
gene-BAE44_0002895                 ..........................MA.........................VS.....
gene-EJB05_33153                   ..........................MA.........................LP.....
Et_6A_046239                       .................................................MA..LS.....
EUGRSUZ_J01153                     ..........................MS.PS................VVPP..VG.....
EVM_prediction_Fe2.12311           ..........................MA.S..................SII..VS.....
FCD_00011565                       ..............................................MSSMT..IT.....
FCD_00011570                       ............................................................
maker-Fvb7-4-snap-gene-70.27       MDLKKPTQPA....NRKRKTKTKQQSMR.....GC..DATRDSKRRYSLST..IT.....
gene-LOC25497742                   ..........................MA....................TPS..VT.....
Mde000077.17                       .......................MS.PS.P..................LPS..VT.....
gene-MERR_LOCUS44217               MSLT......NSLL.SPNMTWSTMPPMP....................LPS..VS.....
gene-E3N88_23834                   ..........................MA.N..................LLS..ST.....
gene-E3N88_23835                   ............................................................
gene-L484_017265                   ..........................MA.SATQ..............VSPL..VT.....
gene-PHJA_002477600                ............................................................
RchiOBHm_Chr7g0221421              ..........................M.....................AQS..VT.....
gene-E2542_SST22124                ..........................ME.......GD.......................
EVM0012662                         M.....................SEK.MP.K..................MPS..VT.....
Tg14g13850                         ..........................MS.A..................SPS..VT.....
Tp57577_TGAC_v2_gene30359          ..........................MA...................MAAS..IT.....
Urofu.3G140000.v1.1                ..........................MG................................
tung.gene.scaffold2716.00002       ..........................MSLA..................TTF..VT.....
Zla05G003490                       ..........................MA.........................VT.....

                                          10          20                30
Medicago                           ....AITV...ENLEYPAVVTSP.VT.....GKS.YFLGGA...................
AH009939.v2.1                      ....DIKV...DDYVFPSAVNAP.GS.....DKS.FFLAGA...................
BjuB05g27840S                      ....KLQV...DSVTFPPSVISP.AS.....SNP.LFLGGA...................
BnaAnng08210D                      ....NLQV...DSVNFPPSVISP.AS.....SNP.LFLGGA...................
BnaC07g45760D                      ....NLQV...DSVNFPPSVISP.AS.....SNP.LFLGGA...................
BnaCnng45660D                      ....KLQV...DSVTFPPSVISP.AS.....SNP.LFLGGA...................
Bo7g117570                         ....NLQV...DSVNFPPSVISP.AS.....SNP.LFLGGA...................
Csa06g029660                       ....ELHV...DSVTFAPSVRSP.AS.....SNP.LFLGGA...................
Csa09g057890                       ....ELHV...DSVTFAPSVRSP.AS.....SNP.LFLGGA...................
Csa09g065990                       ....ELHV...DSVTFAPSVRSL.A.......NP.LFLGGA...................
gene-LOC110809010                  ....ELQV...EAFTFPRFVKPP.GS.....AKT.LFLGGA...................
Cspi12547                          ....ELQV...DGFVFQSTVKPP.GS.....SKT.LILGGA...................
CRGY0216793                        KNGRERGV........PPSVKPP.GS.....SNE.LFLGGA...................
gene-GSCOC_T00026625001            ....EVEV...EGHVFPSSMKPS.GS.....NGT.FFLGGA...................
gene-BT93_J0013                    ....ELQV...ESFVFPLTAKPP.GS.....DNT.LFLGGADSSSLTTTELESSFTLTKP
gene-BAE44_0002895                 ....KLAV...DGVVFPPVARPS.LEQGMVITFI.PSSVRA...................
gene-EJB05_33153                   ....EVVV...EGVVFSPVARPP.GS.....GNT.HFLAGA...................
Et_6A_046239                       ....EVAV...DGVVFPPVSRPP.GS.....SHS.HFLAGA...................
EUGRSUZ_J01153                     ....EIQV...ECFVFPPTVKPP.GS.....GNT.LFLSGA...................
EVM_prediction_Fe2.12311           ....SVAV...EDFVFPPSVRPP.AT.....EKS.FFLGGA...................
FCD_00011565                       ....GVQV...EKAAFPPTVTPP.GS.....AKT.LFLGGA...................
FCD_00011570                       ............................................................
maker-Fvb7-4-snap-gene-70.27       ....GIQI...GGTSFPPAVKPP.GS.....GNT.FFLGGA...................
gene-LOC25497742                   ....SLAI...ESIVFPPTMKAP.GS.....TNN.FFLGGT...................
Mde000077.17                       ....KLVV...DSVTFPPSVNSP.AS.....SNP.LFLGGA...................
gene-MERR_LOCUS44217               ....PLHV...DSFSFPPAITSP.AS.....SKQ.LFLGGA...................
gene-E3N88_23834                   ....GVQV...ETIMFPPHVKPP.GA.....TNT.LFLGGA...................
gene-E3N88_23835                   ............................................................
gene-L484_017265                   ....GLEV...ETQWFPPEVKPP.GS.....AKT.FFLAGA...................
gene-PHJA_002477600                .....MRI...LCLCWKSRCNSP.AA......NV......P...................
RchiOBHm_Chr7g0221421              ....GIQV...EETTFPPAVKPP.GS.....ANT.LFLGGA...................
gene-E2542_SST22124                ...........GTLSF....KEEDGA.....RNN....GGR...................
EVM0012662                         ....QVEV...DACVFLPAVKPP.GS.....DKN.TFLGGA...................
Tg14g13850                         ....KLVV...ESVVFPPAVKPS.GS.....GKT.LFLGGA...................
Tp57577_TGAC_v2_gene30359          ....GVKV...ESFEFPAVVTPP.AY.....PKS.YFLGGA...................
Urofu.3G140000.v1.1                ...........R............QG......RRR...A.....................
tung.gene.scaffold2716.00002       ....QIEVDRLDTVTLSSAVKPP.AS.....NKT.LFLSGT...................
Zla05G003490                       ....EVEV...DGVVFPPVVRPP.GS.....GFT.HFLAGA...................
```

```
                                                                      40        50        60
Medicago                          ..................G..ER.GL.TIEG.NFIKFTAIGVYL.EDIAVASLAAKWKGK
AH009939.v2.1                     ..................G..AR.GL.EIQG.KFVKFTAIGVYL.EKTGISALANKWKGK
BjuB05g27840S                     ..................G..VR.GL.EIQG.KFMIFTVIGVYL.DPVVVLSLSVKWKGK
BnaAnng08210D                     ..........................GI.DVVG.DKFVITFFGVYL.DPVAVPLLSVKWKGK
BnaC07g45760D                     ..................G..VR.GI.DVLGDKFVIITFFGVYL.DPVAVPLLSVKWKGK
BnaCnng45660D                     ..................G..VR.GL.DIHG.KFVIFTVIGVYL.DAVAVPSLFVKWKGK
Bo7g117570                        ..................G..VR.GI.DVLGDKFVIITFFGVYL.DPVAVPLLSVKWKGK
Csa06g029660                      ..................G....SV.VVSS.VFVFFFLLTMRI.H.NAVPSLSLKWKGK
Csa09g057890                      ..................G..S.....VVEG.EFIIFTVVGVYL.DANAVPSLSLKWKGK
Csa09g065990                      ..................G..VR.GL.EIEG.EFIIFTVVGVYL.DANAVPSLSLKWKGK
gene-LOC110809010                 ..................G..VR.GL.DIQG.KFVKFTAIGVYL.EDIAVPSLAVKWKGK
Cspi12547                         ..................G..AR.GL.EIQG.KFVKFTAIGVYV.EGEAIPLLAVKWKGK
CRGY0216793                       ..................G..WR.GL.DIQG.KFVKFTAIGVSG.EQRR....HDPFRQV
gene-GSCOC_T00026625001           ..................G..YR.GL.EIQG.KFIKFSLPGVYL.EETAIPSLATKWKGK
gene-BT93_J0013                   IAEREERRKRFRISMTYGG..VR.GL.EIDG.KHVKYTAIGVYL.EVTAVPILAVKWKGK
gene-BAE44_0002895                ..................G..VH.GL.EIGG.NFIKFTAIGVYLEEDAAVSALAKKWTGK
gene-EJB05_33153                  ..................G..VR.GL.EIEG.KFIKFAAIAVYL.EDAAAAALSGKWSGK
Et_6A_046239                      ..................G..VR.GL.EIGG.NFIKFTAIGVYL.EDMAVTALAKRWAGK
EUGRSUZ_J01153                    ..................G..VR.GL.EVDG.KYVKYTAIGVYL.EAKAVPILAAKWKGK
EVM_prediction_Fe2.12311          ..................G..VR.GM.TIEG.KFITFSAIGIYF.EETAVASLADKWNGK
FCD_00011565                      ..................G..VR.GM.EIRG.KFVKFTAIGVYL.ESNAVTWLAGKWEGK
FCD_00011570                      .....................MR.GL.EIQG.KFVKFMAIGVYL.ELNAVTWLAGKWKGK
maker-Fvb7-4-snap-gene-70.27      ..................G..AR.GM.EIQG.NFVKFTAIGVYL.EGKAVPALAVKWKGK
gene-LOC25497742                  ..................G..AR.GI.QIQD.KFVKFTAIGVYL.QDIAVPYLAEKWKAK
Mde000077.17                      ..................G..VR.GL.DIQG.KFVIFTVIGIHL.DCNAVSSLSVKWKGK
gene-MERR_LOCUS44217              ..................G..VR.GL.EIEG.KFVTVTVIGVYL.DAMAVPSLSVKWKGK
gene-E3N88_23834                  ..................G..VR.CV.EIES.KLVKISGIGVYL.EDKAIQSLAATWKGK
gene-E3N88_23835                  ...............................................................
gene-L484_017265                  ..................G..WR.GM.EVDG.KFVKFTTTGVYL.KDEAVSWLAAKWKGK
gene-PHJA_002477600               ..................G..VR.GL.EIGG.KFVKFTAIGVYL.EGNAVPSLAVKWKGK
RchiOBHm_Chr7g0221421             ..................G..AR.GL.EIQG.NFVRFTAIGVYL.EDKAGPALAVKWSGR
gene-E2542_SST22124               ..................G..ER.GEVTIEG.ESVKVTAIGVYW.EDKAVSLIAAKWKGK
EVM0012662                        ..................GQGERTGN...RG.KIHQIHSHGSVP.GGCRRSVARLKWNGK
Tg14g13850                        ..................G..VR.GM.EIEG.KFVKFTAIGVYL.EDNAVPSLADKWKGK
Tp57577_TGAC_v2_gene30359         ..................G..VR.GL.DIDG.EFVKFTAIGVYL.EENAVASLTAKWKAK
Urofu.3G140000.v1.1               ...............................................................
tung.gene.scaffold2716.00002      ..................G..VR.GL.DIQG.KFLKFAAIGVYL.EDKAVPLLTVKWKGA
Zla05G003490                      ..................G..VR.GL.EIGG.NFIKFTAIGVYL.EDAAVPSLAKKWAGK
```

```
                                  70        80                                     90
Medicago                          SSEELLETLDFYRDIIS.......GP...................FEKLI.....RG.S
AH009939.v2.1                     TTTELLDSDEFFRDIVT.......GP...................FEKFT.....QV.T
BjuB05g27840S                     TTEELTESVPFFREIVT..............................................V.A
BnaAnng08210D                     TT.ELMESVPFFREVVT.......GT...................FEKLI.....KV.M
BnaC07g45760D                     TTEELMESVPFFREVVT.......GT...................FEKFI.....KV.T
BnaCnng45660D                     TTEELTESVPFFREIVT.......GS...................FEKFI.....KV.T
Bo7g117570                        TTEELMESVPFFREVVT.......GT...................FEKFI.....KV.T
Csa06g029660                      TAEELTESVPFFREIVT.......GA...................FEKFI.....KV.T
Csa09g057890                      TAEELMESVPFFREIVT.......GA...................FEKFI.....KL.T
Csa09g065990                      PAEELTESVPFFREIVT.......GA...................FEKFV.....KV.T
gene-LOC110809010                 SADELTESIEFFRDVVT.......GP...................FKKFI.....RV.T
Cspi12547                         KAEELADSVDFYRDIVT..DWI.......................FQRWN..........
CRGY0216793                       EGQE.....................CGGS.................H..SKKFT.....KV.T
gene-GSCOC_T00026625001           TAEELMESVEFFRDIIT.......GN...................TTLFS.....FF.S
gene-BT93_J0013                   SADELRDSIEFFRDVVT.......GP...................FEKFT.....QV.S
gene-BAE44_0002895                SADELASDVAFLCDVVT.......GD...................FEKFT.....QV.T
gene-EJB05_33153                  TADELAADPAFFRGGVQ.......GG...................LQATDVLAAGRV.R
Et_6A_046239                      TAEELAADVAFFRDVVT.......GE...................FEKFT.....RV.T
EUGRSUZ_J01153                    TADELRDSIDFFRDVVT.......GP...................FEKLT.....QV.S
EVM_prediction_Fe2.12311          SATELTESVEFFRDVVT.......GP...................FEIFM.....QI.T
FCD_00011565                      SADELTNSVQFFRDIVS.......GP...................FEKFT.....RI.T
FCD_00011570                      SAEELNDSVEFYRDIVR...............................................
maker-Fvb7-4-snap-gene-70.27      TAEELTESVEFFREIRMTY.....RP....MDMVEVRLDGGQGH.RYSTWT.....PA.Q
gene-LOC25497742                  SAHELTDTVPFFRDIVT.......GP...................FEKFM.....RV.T
Mde000077.17                      TKEELTESVPFFREIVT.......GD...................LLTFH.....HL..
gene-MERR_LOCUS44217              NAKELTESISFFRQLIT.......GA...................FEKFT.....RV.T
gene-E3N88_23834                  GVEELQDSEDFFKDIIN.......GN...................PQDLI.....NS.R
gene-E3N88_23835                  ...............................................................
gene-L484_017265                  TAEELLESDEFFQDIVT.......GP...................FEKFY.....RL.T
gene-PHJA_002477600               SAKELTDSVGFFADIIT.......GP...................FEKFT.....KI.I
RchiOBHm_Chr7g0221421             VRRPRS...................P...................LEKFT.....QV.T
gene-E2542_SST22124               SAAELLESLDFYRDIIK.......GTMKYMMPLCIERLK..MKHDFLEKLI.....RG.S
EVM0012662                        STKELMDSNDFL...................................................
Tg14g13850                        SADELTDSVDFFRDIVT.......GP...................FEKFT.....KV.T
Tp57577_TGAC_v2_gene30359         TPAQLFESLDFYRDIIK.......AS...................VS................
Urofu.3G140000.v1.1               ...RVGPRLLPRR............L...................HRRVR.....EVHE
tung.gene.scaffold2716.00002      TAQELTNSVEFFRDIVK.......GP...................FEKFI.....RV.T
Zla05G003490                      TADELASDAAFFCDVVT.......GD...................FEKFT.....RV.T
```

```
                                 100               110       120
Medicago                      KI.RELS.GP......EYS.RKVMENC.VAHLKSV.....GTYGDAE.............
AH009939.v2.1                 MI.LPLT.GQ......QYS.EKGNREL.CCLLESH.........................
BjuB05g27840S                 MK.LPLT.GQ......QYS.EKVTENC.VAIWKSL.....GIYTDSE.............
BnaAnng08210D                 MR.VPLP.GQ......LYS.QIITGTS.VKIWKSL.....GIYTYSE.............
BnaC07g45760D                 MR.VPLP.GQ......LYS.QTVTGTF.VEIWKSL.....GIYTDSE.............
BnaCnng45660D                 MK.LPLT.GQ......QYS.EKVTENC.VAIWKSL.....GIYTDSE.............
Bo7g117570                    MR.VPLP.GQ......LYS.QTVTGTF.VEIWKSL.....GIYTDSE.............
Csa06g029660                  MK.LPLF.GQ......QYW.ERVMENC.VAMWKSL.....GIYTDCE.............
Csa09g057890                  MK.QPLF.GH......KIA.EEVMKKC.VATWKFL.....EIYTECE.............
Csa09g065990                  IK.QPFF.GQ......KYW.ERVMANC.VAIWKSL.....GIYTDCE.............
gene-LOC110809010             MI.LPLT.GQ......QYS.EKVTENC.VAFWKSV.....GIYTDAE.............
Cspi12547                     ........GIRTR.ECSYR.........................................
CRGY0216793                   YA.VAVG.RA......RCTRRRWWRRI.VATLESL.....GPLHRSE.............
gene-GSCOC_T00026625001       FI..........................................................
gene-BT93_J0013               FI.TQLT.GP......EYT.DKL..HC.FLEFHGD........KQEE.............
gene-BAE44_0002895            MI.LPLI.GE......QYS.KKVMENC.IAYWKAT.....DEYTDAK.............
gene-EJB05_33153              PL.HPLA.GR......SHH.RELLTVH.................................
Et_6A_046239                  MI.RLLT.GE......QYS.EKVAENC.MAHWKAT.....GGYTDAE.............
EUGRSUZ_J01153                FI.TQLT.GQ......QYT.NKVTENC.IAFWKSN.....GGYKQEE.............
EVM_prediction_Fe2.12311      LL.KHLT.GA......QYS.AKVAENC.VAICKAL.....GTYSEAE.............
FCD_00011565                  MI.LPLT.GK......VYS.EKVAENC.EAIWKSL.....GIYTETE.............
FCD_00011570                  .....LR.SY......SYN.DYFAVDR.ATILGE......GVEELPE.............
maker-Fvb7-4-snap-gene-70.27  FA.LNLK.GRIDQFDIRHG.ASTMNNCLVQYWRKQNDGWSGVYVADNQIRPCPPQHEEIE
gene-LOC25497742              MI.RPLT.GQ......EYS.NKVSENC.VAIWKSL.....GIYTNEE.............
Mde000077.17                  ............................................................
gene-MERR_LOCUS44217          MK.VKLS.GT......QYS.EKVAEYC.EEILKSS.....GRYTKSE.............
gene-E3N88_23834              LE..........................................................
gene-E3N88_23835              MI.RSLT.GK......QFS.EKVSEHC.VGIWKAQ.....GTYTDED.............
gene-L484_017265              HI.RRLE.GE......EFS.GKVGGHL.AGMIKSA.....GAYGEAE.............
gene-PHJA_002477600           TI.MPLT.GQ......QYS.EKVAEKL.RRLLESNR..........E.............
RchiOBHm_Chr7g0221421         MI.LPLT.GQ......QYS.EKVSENC.VAIW............................
gene-E2542_SST22124           KI.ISLD.GP......EYV.RKLSENC.VAHMKSV.....GTYSNAE.............
EVM0012662                    .........Q......PSS.LKVVENC.IANLKFS.....GKYGDVE.............
Tg14g13850                    ML.LPLT.GQ......QYS.GKVAENC.VASWKAV.....GKYTDAE.............
Tp57577_TGAC_v2_gene30359     ..SSPLL.........VFSFETIKIGC.................................
Urofu.3G140000.v1.1           GD.FPLAEGR......R.........R.VAYLKAA.....GAYTDAE.............
tung.gene.scaffold2716.00002  MV.LPLT.GQ......QYS.EKVSENC.VAIWKSE.........................
Zla05G003490                  MI.LPLT.GE......QYS.DKVAENC.VAYWKAT.....GVYTDAE.............

                                    130               140
Medicago                      ......AEAMQKFAEA.........FKPVNFPP........................GAS
AH009939.v2.1                 ............................................................
BjuB05g27840S                 ......AKDVERFVQV.........FKDEMFPP........................SAS
BnaAnng08210D                 ......AKAVERFLEV.........FKDEKFPR........................GAS
BnaC07g45760D                 ......AKAVERFLEV.........FKDEKFPR........................GAS
BnaCnng45660D                 ......AKAVKKFLEV.........FKDQTFPP........................GAS
Bo7g117570                    ......AKAVEKFLEV.........FKDEKFPR........................GAS
Csa06g029660                  ......AKAVETFLEV.........LKDENFLP........................GAS
Csa09g057890                  ......AKAVKTFLEV.........FKDENFLP........................GAS
Csa09g065990                  ......AKAVETFLEV.........FKDENFLP........................GAS
gene-LOC110809010             ......AKAIEKFLRV.........FKDENFPP........................GSS
Cspi12547                     ......EQA...................................................
CRGY0216793                   ......DEAIDTFTEV.........CQDETFPP........................ASS
gene-GSCOC_T00026625001       ............................................................
gene-BT93_J0013               ......AKAIDELIEV.........FKDQTFPP........................GSS
gene-BAE44_0002895            ......GVAVEKFKEA.........FKPEKFPP...........................
gene-EJB05_33153              ......ARA...................................................
Et_6A_046239                  ......GEAAVKFKEA.........FKPETFPP........................GAS
EUGRSUZ_J01153                ......AEGIDKFIEV.........FKDQTFPP........................GSS
EVM_prediction_Fe2.12311      ......EKAVEKFMEI.........FEEHNFPP........................GTS
FCD_00011565                  ......EKALEKFLQI.........FKDQNLLP........................GSS
FCD_00011570                  ......EKAIEMFLQV.........FKDKNFPP........................GSF
maker-Fvb7-4-snap-gene-70.27  FKWLGM...VDEFVDNSWWVGTITDVRADNYMVTIKLTNEQKECNHSQLRRHCEWIDGKW
gene-LOC25497742              ......IKAINKFVSV.........FKDETFPP........................GSS
Mde000077.17                  ............................................................
gene-MERR_LOCUS44217          ......AKAMEEFLVV.........FRDQDFPP........................GSS
gene-E3N88_23834              ............................................................
gene-E3N88_23835              ......AKAIDKFIEA.........YKDQNFPP........................GSS
gene-L484_017265              ......AKAVDKFIVL.........YKDKEFLS.......................VGFS
gene-PHJA_002477600           ............................................................
RchiOBHm_Chr7g0221421         ............................................................
gene-E2542_SST22124           ......EKAIGDFRYAF.......YFKDQNFPP........................CAS
EVM0012662                    ......SEALEKFLQV.........FKDET..............................
Tg14g13850                    ......AEAIKKFLEV.........FKDETFPP........................GAS
Tp57577_TGAC_v2_gene30359     ......NRA...................................................
Urofu.3G140000.v1.1           ......AAAVEECKAA.........FKELSLAP........................GA.
tung.gene.scaffold2716.00002  ............................................................
Zla05G003490                  ......GVAVDKFKEA.........FKPHSFPP........................GAS
```

```
                                     150         160
Medicago                             VFY.RQSPD..GIL...G............LS..........FS...P......DT....
AH009939.v2.1                        ..........................................WN................
BjuB05g27840S                        ILF.ALSSK..GSL...TLIGAIDLHLRLHVA..........FS...K......DD....
BnaAnng08210D                        ILF.ALSPE..GSL...T............IA..........FS...K......DD....
BnaC07g45760D                        ILF.ALSPE..GSL...T............IA..........FS...K......DD....
BnaCnng45660D                        ILF.ALSPN..GSL...T............IA..........FS...K......DD....
Bo7g117570                           ILF.ALSPE..GSL...T............IA..........FS...K......DD....
Csa06g029660                         ILF.ALSPN..GSL...T............VA..........FS...K......DD....
Csa09g057890                         ILF.AFSPN..GSL...T............VA..........FS...K......DD....
Csa09g065990                         ILF.AFSPN..GSL...T............VA..........FS...K......DD....
gene-LOC110809010                    IFF.TQSPN..GSL...T............IT..........FS...K......DE....
Cspi12547                            .................A............IG............................
CRGY0216793                          IFH.TVQPW..SFD...D............HA..........SP...K......EG....
gene-GSCOC_T00026625001              ...............................................R............
gene-BT93_J0013                      IFF.THLTN..GSY................VG..........HA...I......EP....
gene-BAE44_0002895                   ............................................................
gene-EJB05_33153                     ..........................................FT................
Et_6A_046239                         VLF.TCSPR..GIF...T............IA..........FS...E......DS....
EUGRSUZ_J01153                       IFF.THLTN..GSY...V............VS..........IF................
EVM_prediction_Fe2.12311             ILF.KQIPC..GIL...L............IA..........FG...K......HN....
FCD_00011565                         VLF.TQSPS..RFL...D............VDHRKAW....SFS...G......NE....
FCD_00011570                         VLF.TQSLS..DSL...M............AN............................
maker-Fvb7-4-snap-gene-70.27         EFHMHYCSS..GFVLL..............MG...MWDDAHAFYWRYKGPKVEADSSKVE
gene-LOC25497742                     ILF.TVSPKGSGSL...T............IS..........FS...K......DG....
Mde000077.17                         ..............................LN..........FD................
gene-MERR_LOCUS44217                 IFF.AICPR..GSL...T............VS............................
gene-E3N88_23834                     ..............................VS..........FV................
gene-E3N88_23835                     IHH.TISPA..GSL...M............IS..........FS...K......DG....
gene-L484_017265                     NLY.HQSPT..GSL...T............VR...............K............
gene-PHJA_002477600                  ..............................IH..............CR............
RchiOBHm_Chr7g0221421                ............................................................
gene-E2542_SST22124                  VFY.KQLPT..GTL...G............LS..........FS...K......DE....
EVM0012662                           ............................................................
Tg14g13850                           ILF.TQSPV..GSL...T............VC..........IL...S......FR....
Tp57577_TGAC_v2_gene30359            ................RN.........................FY...H............
Urofu.3G140000.v1.1                  ..L.THSLA..GVL...T............VA..........CS...D......DS....
tung.gene.scaffold2716.00002         ............................................................
Zla05G003490                         ILF.THSPA..GVL...T............VS...........S...C......TR....
```

```
                                                    170        180            190
Medicago                             ..............SIPEKEAALIE.NKAVSSAV.....LETM.IGEHAV..........
AH009939.v2.1                        .......................IQ...................................
BjuB05g27840S                        ..............SIPETGKA.........RQF.....LNRS.LGRT.V..........
BnaAnng08210D                        ..............SIPETGKAVIE.NKLLTEAS..........................
BnaC07g45760D                        ..............NIPETGKAVIE.NKLLAEAV.....LEFI.IGTEAA..........
BnaCnng45660D                        ..............SIPKTGKAVIE.NKLLAEA...........................
Bo7g117570                           ..............SIPVTGKAVIE.NKLLAEAV.....LEFI.IGTEAA..........
Csa06g029660                         ..............SIPETGKAVIQ.NKLLAEDV.....LELF.IEKKVA..........
Csa09g057890                         ..............SIPETGKAVIQ.NKMLAEDV.....LELF.IEKNGA..........
Csa09g065990                         ..............SIPETGKAVIQ.NKLLAEDV.....LELF.IAKKCA..........
gene-LOC110809010                    ..............CI............................................
Cspi12547                            .....................G.IS.......................GV..........
CRGY0216793                          ..............ACQE.GECSDE.NKVLPERG.....AGVH.HRQDGV..........
gene-GSCOC_T00026625001              ............................................................
gene-BT93_J0013                      ..............GLPRTRTRPLE.....................KHDR..........
gene-BAE44_0002895                   ............................................................
gene-EJB05_33153                     .....................T......................................
Et_6A_046239                         ..............SVPEVDGVAVE.NKQLCEAV.....L..I.IGEHGV..........
EUGRSUZ_J01153                       ............................................................
EVM_prediction_Fe2.12311             ..............FIPEADVVAKG.NVPF..............................
FCD_00011565                         ..............EKPGFKFSGIA.HRSEQDSS.....PPAL.RREENV..........
FCD_00011570                         ..............................SGAI.....LELI.IGKHGV..........
maker-Fvb7-4-snap-gene-70.27         DKGVKSEADIADLKVVPP...SMVKEEMNLTMKVVPPCKLSQMHISSHQVRQIGMKSERD
gene-LOC25497742                     ..............SIPEVETTVIS...................................
Mde000077.17                         ............................................................
gene-MERR_LOCUS44217                 ............................................................
gene-E3N88_23834                     ............................................................
gene-E3N88_23835                     ..............SIPKTMNSVIE.NEKIGPAI.....IEMV.IGKHGV..........
gene-L484_017265                     .....................T......................................
gene-PHJA_002477600                  ....................R..VRSHQ................................
RchiOBHm_Chr7g0221421                ............................................................
gene-E2542_SST22124                  .....NE.......TIPEHEYAVID.NKPLSEAV.....LAGDYDPRDSC..........
EVM0012662                           ..............SIPETGAAVIE.NKLLSEAV.....LELI.IGKEGV..........
Tg14g13850                           ..............SYLSQGSCSCS.N.................................
Tp57577_TGAC_v2_gene30359            ................PEIGAHTIK.RRDESDGV..........VVTGGE..........
Urofu.3G140000.v1.1                  ..............SVPEAGIAAIE.NKALCEAV.....LEAIVIGERSV..........
tung.gene.scaffold2716.00002         ............................................................
Zla05G003490                         ..............PIPRSLRRV................LQRL.VGAGGRRRGG....DR
```

```
                                      200
Medicago                              .SPDLK....................RCLAA.............................
AH009939.v2.1                         ............................................................
BjuB05g27840S                         .SPGAR....................LSVAK.............................
BnaAnng08210D                         ............................................................
BnaC07g45760D                         .SPRTR....................LSVAE.............................
BnaCnng45660D                         ............................................................
Bo7g117570                            .SPRTR....................LSVAE.............................
Csa06g029660                          .CRGAR....................LSVTE.............................
Csa09g057890                          .CPGAR....................LTVAE.............................
Csa09g065990                          .CRGAR....................LSVAE.............................
gene-LOC110809010                     ............................................................
Cspi12547                             ..............................................DY............
CRGY0216793                           .SPAAK....................KSLAAFSKIQENRVKSLEEKRYQLTKNQRLEVDP
gene-GSCOC_T00026625001               ............................................................
gene-BT93_J0013                       .EPVPS......................................................
gene-BAE44_0002895                    ............................................................
gene-EJB05_33153                      ............................................................
Et_6A_046239                          .PPAAK....................LSIAA.............................
EUGRSUZ_J01153                        ............................................................
EVM_prediction_Fe2.12311              ............................................................
FCD_00011565                          .KGLEI....................QGKFV.............................
FCD_00011570                          .SPDAK....................RSMAS.............................
maker-Fvb7-4-snap-gene-70.27          EDEAIKSEADNAAGLKVVPPWMVKKEMNLTM..........................EQP
gene-LOC25497742                      ...CVK....................DHLA..............................
Mde000077.17                          ..........................LSVAS.............................
gene-MERR_LOCUS44217                  ..........................KLLYS.............................
gene-E3N88_23834                      ............................................................
gene-E3N88_23835                      .SPETK....................KNVAS.............................
gene-L484_017265                      ............................................................
gene-PHJA_002477600                   ............................................................
RchiOBHm_Chr7g0221421                 ............................................................
gene-E2542_SST22124                   .FPCFK....................REFGY.............................
EVM0012662                            .SPEAK......................................................
Tg14g13850                            ............................................................
Tp57577_TGAC_v2_gene30359             ............................................................
Urofu.3G140000.v1.1                   .SPATK....................QSIAT.............................
tung.gene.scaffold2716.00002          ............................................................
Zla05G003490                          EQGALR....GGAGVNH......RRARGLAG............GEAEF.........GGP

                                                                               210
Medicago                              ..................................RL.....PAL................
AH009939.v2.1                         ............................................................
BjuB05g27840S                         ..................................RL.....AQL................
BnaAnng08210D                         ............................................................
BnaC07g45760D                         ..................................RL.....SQL................
BnaCnng45660D                         ...........................................L................
Bo7g117570                            ..................................RL.....SQL................
Csa06g029660                          ..................................RL.....AQL................
Csa09g057890                          ..................................RL.....AQL................
Csa09g065990                          ..................................RL.....AQL................
gene-LOC110809010                     ............................................................
Cspi12547                             ..................................RR.....TWH................
CRGY0216793                           SLAFFDSILANPSNPPTHRCNCRRPIKPPHPRMARI.....TEIMLLSVTLLSLMAAAVA
gene-GSCOC_T00026625001               ............................................................
gene-BT93_J0013                       ............................................................
gene-BAE44_0002895                    ...............EKKK..........NAKGYVV........................
gene-EJB05_33153                      ............................................................
Et_6A_046239                          ..................................RM.....SEL................
EUGRSUZ_J01153                        ............................................................
EVM_prediction_Fe2.12311              ............................................................
FCD_00011565                          ..................................KF.....TAI.............GVY
FCD_00011570                          ..................................IL.....AEV................
maker-Fvb7-4-snap-gene-70.27          G.................................KL.....SQM........HVSSHQVR
gene-LOC25497742                      ............................................................
Mde000077.17                          ............................................................
gene-MERR_LOCUS44217                  ..................................SK.....SKC................
gene-E3N88_23834                      ............................................................
gene-E3N88_23835                      ..................................RL.....STI................
gene-L484_017265                      ............................................................
gene-PHJA_002477600                   ............................................................
RchiOBHm_Chr7g0221421                 ............................................................
gene-E2542_SST22124                   ..................................KV.....LCV................
EVM0012662                            ............................................................
Tg14g13850                            ............................................................
Tp57577_TGAC_v2_gene30359             .........................................NEA................
Urofu.3G140000.v1.1                   ..................................RM.....PEI................
tung.gene.scaffold2716.00002          ............................................................
Zla05G003490                          GRG......APEGGRPRRR..........AGGGARVGVSLKTEL................
```

```
                                                         220
                                                           .
Medicago                      ....LN.EGAF...............K.I........GN.....................
AH009939.v2.1                 ............................................................
BjuB05g27840S                 ....MK.NNKV...............E.E........DA.TKT.D...QEEAND......
BnaAnng08210D                 .....S......................................................
BnaC07g45760D                 ....MN.SDKV...............E.E........DA.TKT.................
BnaCnng45660D                 ....MK.ENKV...............E.K........DA.TKT..DN.QDEAND......
Bo7g117570                    ....MN.SDKV...............E.E........DA.TKT.................
Csa06g029660                  ....MK.ENKV...............E.E........EE.H...................
Csa09g057890                  ....MK.STEN...............D.I........DF.S...................
Csa09g065990                  ....MN.ENKV...............E.E........EE.N...................
gene-LOC110809010             ............................................................
Cspi12547                     ....FT......................................................
CRGY0216793                   EIKNLKIQSDARPMIEFEKFGFTHTGKVSISVSDVSIET.TSA.AAGSPDKSLFGFFLLS
gene-GSCOC_T00026625001       ............................................................
gene-BT93_J0013               ............................................................
gene-BAE44_0002895            ............................................................
gene-EJB05_33153              ............................................................
Et_6A_046239                  ....LN.GTST...............T.A........G..DAL.QA....D.........
EUGRSUZ_J01153                ............................................................
EVM_prediction_Fe2.12311      ............................................................
FCD_00011565                  ....L..EFNAVTWL.........AGK.........WKA.NRP.RS..............
FCD_00011570                  ....LM.DQNK...............T.H........AV.PSG.EKK.............
maker-Fvb7-4-snap-gene-70.27  QID.MKSEPDE..........................DEGDDDIDQE.............
gene-LOC25497742              ....FK.IIAA...............A.................................
Mde000077.17                  .....N......................................................
gene-MERR_LOCUS44217          ....VE......................................................
gene-E3N88_23834              ............................................................
gene-E3N88_23835              ....IN......................................................
gene-L484_017265              ............................................................
gene-PHJA_002477600           ............................................................
RchiOBHm_Chr7g0221421         ............................................................
gene-E2542_SST22124           .....S.ETGQ..........................SQ.H...................
EVM0012662                    ............................................................
Tg14g13850                    ............................................................
Tp57577_TGAC_v2_gene30359     .....S......................................................
Urofu.3G140000.v1.1           ....LK.GGA..................................................
tung.gene.scaffold2716.00002  ............................................................
Zla05G003490                  ....LN.ARDA..............GR.........PARCSPA.................
```

```
Medicago                      ............................................................
AH009939.v2.1                 ............................................................
BjuB05g27840S                 ....LSPGDKLA..............................KEN...............
BnaAnng08210D                 ............................................................
BnaC07g45760D                 ............................................................
BnaCnng45660D                 ....VSLGDKLA..............................KEN...............
Bo7g117570                    ............................................................
Csa06g029660                  ............................................................
Csa09g057890                  ............................................................
Csa09g065990                  ............................................................
gene-LOC110809010             ............................................................
Cspi12547                     ............................................................
CRGY0216793                   DESLLQVIQEASQNPSFCVLHSLFTLHLFTFQDLNPDLSFSHNDSVKIPNEYSLYFANCN
gene-GSCOC_T00026625001       ............................................................
gene-BT93_J0013               ............................................................
gene-BAE44_0002895            ............................................................
gene-EJB05_33153              ............................................................
Et_6A_046239                  ....VAISS...................................................
EUGRSUZ_J01153                ............................................................
EVM_prediction_Fe2.12311      ............................................................
FCD_00011565                  ............................................................
FCD_00011570                  ...LLAV.....................................................
maker-Fvb7-4-snap-gene-70.27  ............................................................
gene-LOC25497742              ............................................................
Mde000077.17                  ............................................................
gene-MERR_LOCUS44217          ............................................................
gene-E3N88_23834              ............................................................
gene-E3N88_23835              ............................................................
gene-L484_017265              ............................................................
gene-PHJA_002477600           ............................................................
RchiOBHm_Chr7g0221421         ............................................................
gene-E2542_SST22124           ............................................................
EVM0012662                    ............................................................
Tg14g13850                    ............................................................
Tp57577_TGAC_v2_gene30359     ............................................................
Urofu.3G140000.v1.1           ............................................................
tung.gene.scaffold2716.00002  ............................................................
Zla05G003490                  ....VRVNCQRYH...............................................
```

**Medicago**	....
**AH009939.v2.1**	....
**BjuB05g27840S**	....
**BnaAnng08210D**	....
**BnaC07g45760D**	....
**BnaCnng45660D**	....
**Bo7g117570**	....
**Csa06g029660**	....
**Csa09g057890**	....
**Csa09g065990**	....
**gene-LOC110809010**	....
**Cspi12547**	....
**CRGY0216793**	PGTLVTMSVRTELFNLDSGVKDYLSAGQTQLPALYFLFPSLISSF
**gene-GSCOC_T00026625001**	....
**gene-BT93_J0013**	....
**gene-BAE44_0002895**	....
**gene-EJB05_33153**	....
**Et_6A_046239**	....
**EUGRSUZ_J01153**	....
**EVM_prediction_Fe2.12311**	....
**FCD_00011565**	....
**FCD_00011570**	....
**maker-Fvb7-4-snap-gene-70.27**	....
**gene-LOC25497742**	....
**Mde000077.17**	....
**gene-MERR_LOCUS44217**	....
**gene-E3N88_23834**	....
**gene-E3N88_23835**	....
**gene-L484_017265**	....
**gene-PHJA_002477600**	....
**RchiOBHm_Chr7g0221421**	....
**gene-E2542_SST22124**	....
**EVM0012662**	....
**Tg14g13850**	....
**Tp57577_TGAC_v2_gene30359**	....
**Urofu.3G140000.v1.1**	....
**tung.gene.scaffold2716.00002**	....
**Zla05G003490**	......YPTLLHSY...............................
