# Supplementary figures and images for: Evolutionary landscape of plant chalcone isomerase-fold gene families
Source: Front Plant Sci. 2025 Mar 28;16:1559547. doi: 10.3389/fpls.2025.1559547 (PMC11985768; doi:10.3389/fpls.2025.1559547)

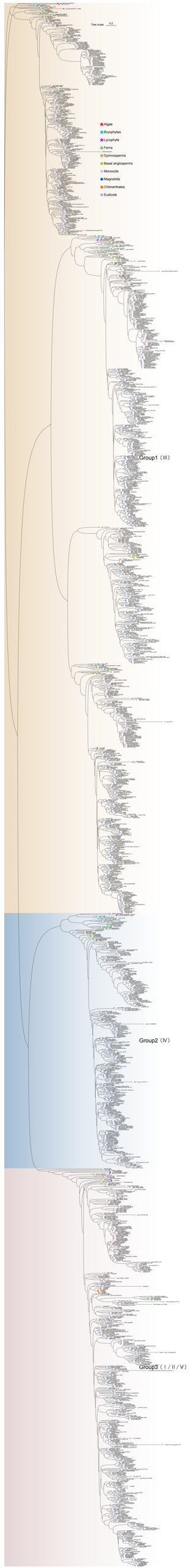

Supplement: Supplementary Table 1 — The genomic data source of 259 species. [file DataSheet1.zip › Supplementary_information/Figure S2.pdf]

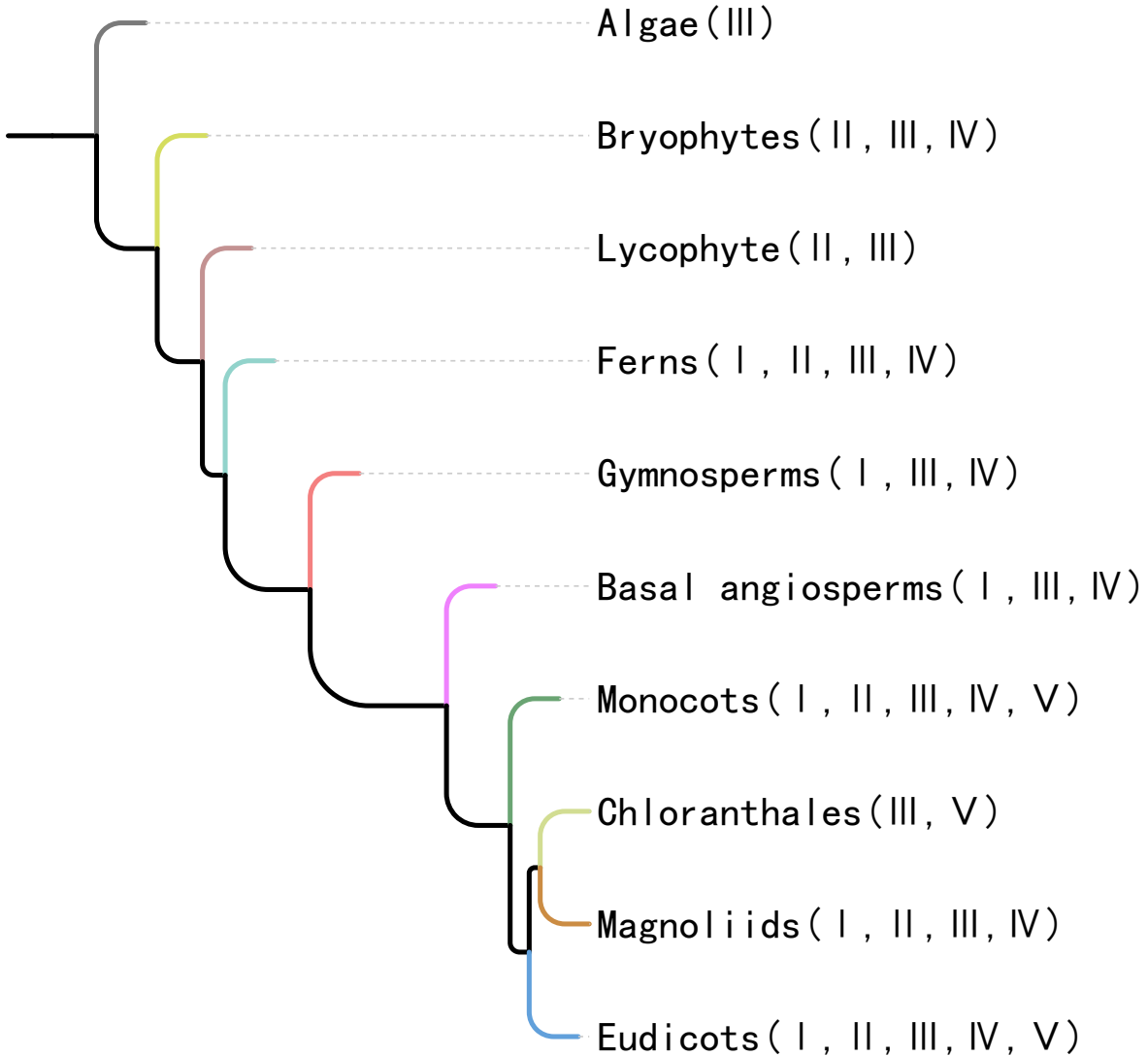

Supplement: Supplementary Table 1 — The genomic data source of 259 species. [file DataSheet1.zip › Supplementary_information/Figure S6.pdf]

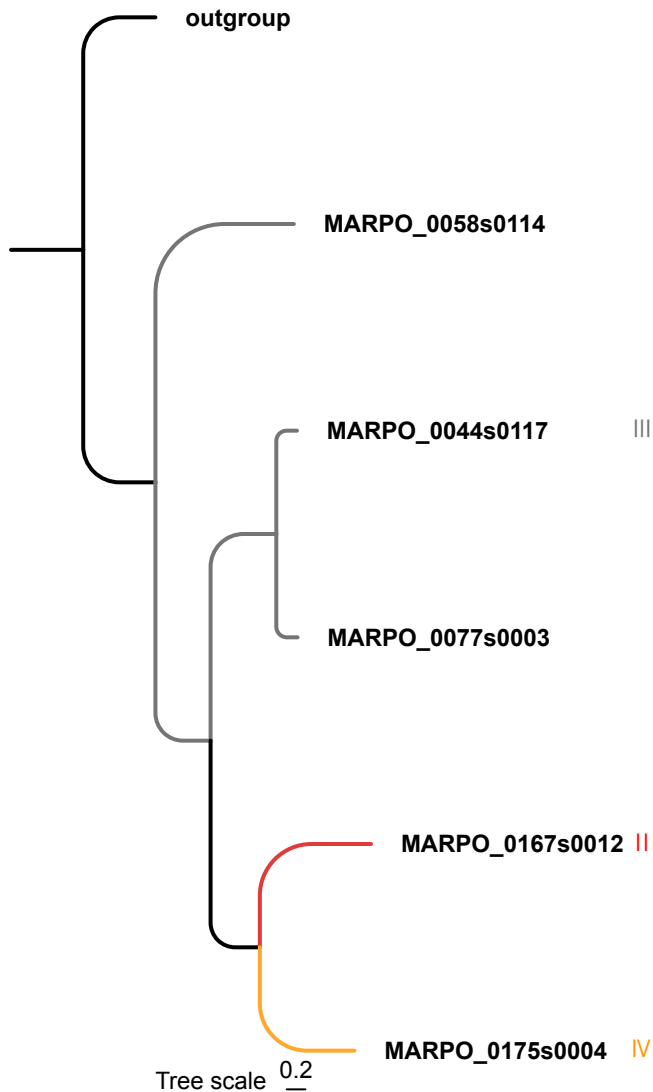

Supplement: Supplementary Table 1 — The genomic data source of 259 species. [file DataSheet1.zip › Supplementary_information/Figure S7.pdf]

Motif1

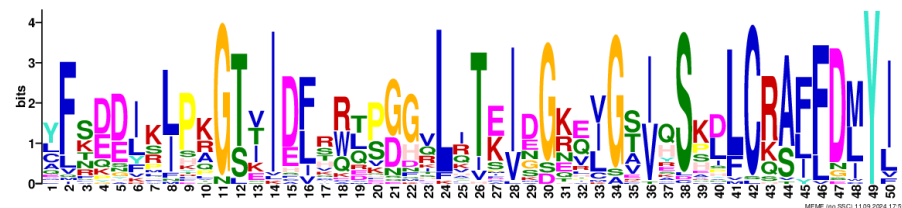

Motif2

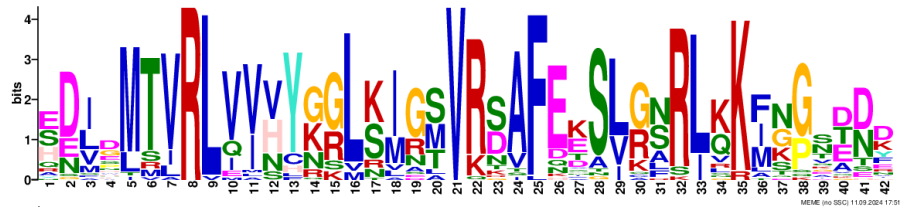

Motif3

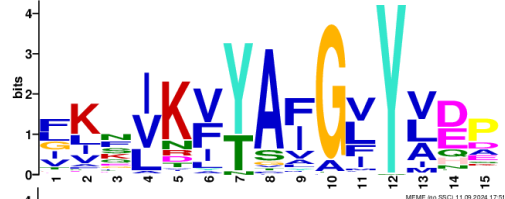

Motif4

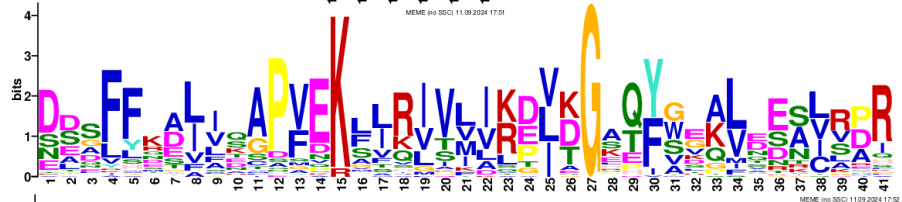

Motif5

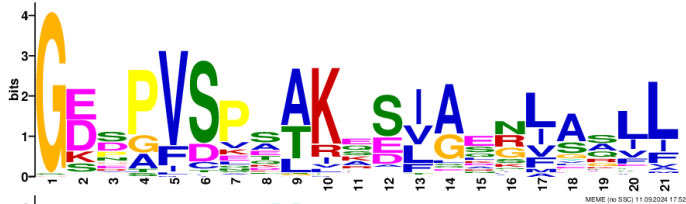

Motif6

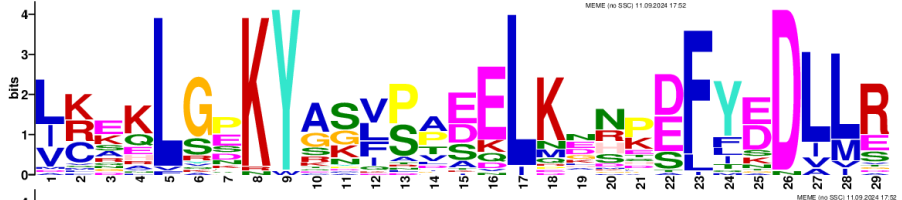

Motif7

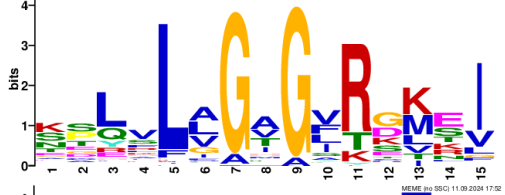

Motif8

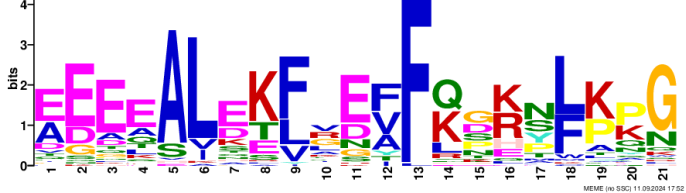

Motif9

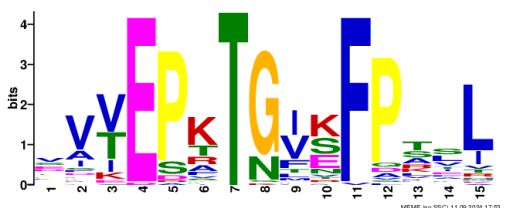

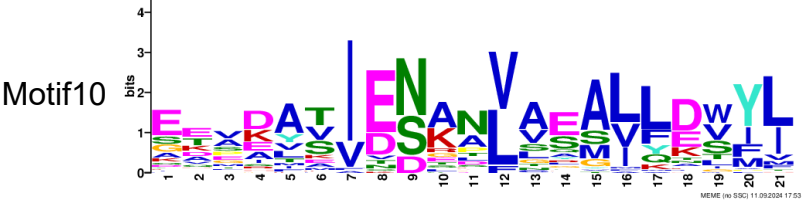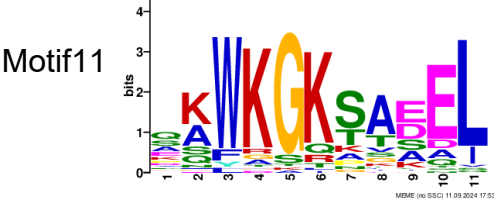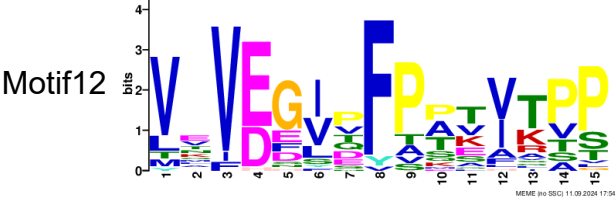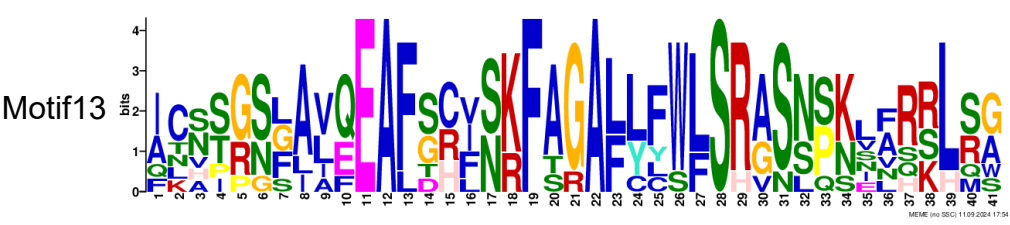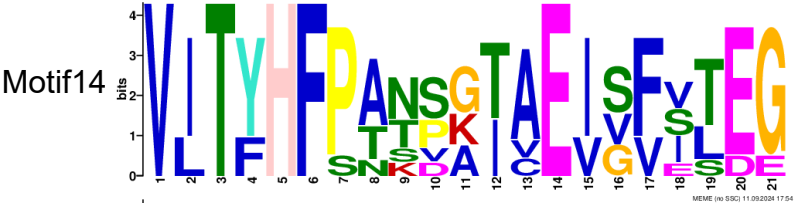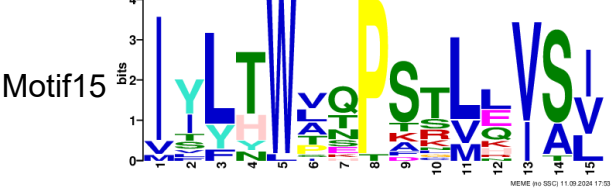

Supplement: Supplementary Table 1 — The genomic data source of 259 species. [file DataSheet1.zip › Supplementary_information/Figure S8.pdf]

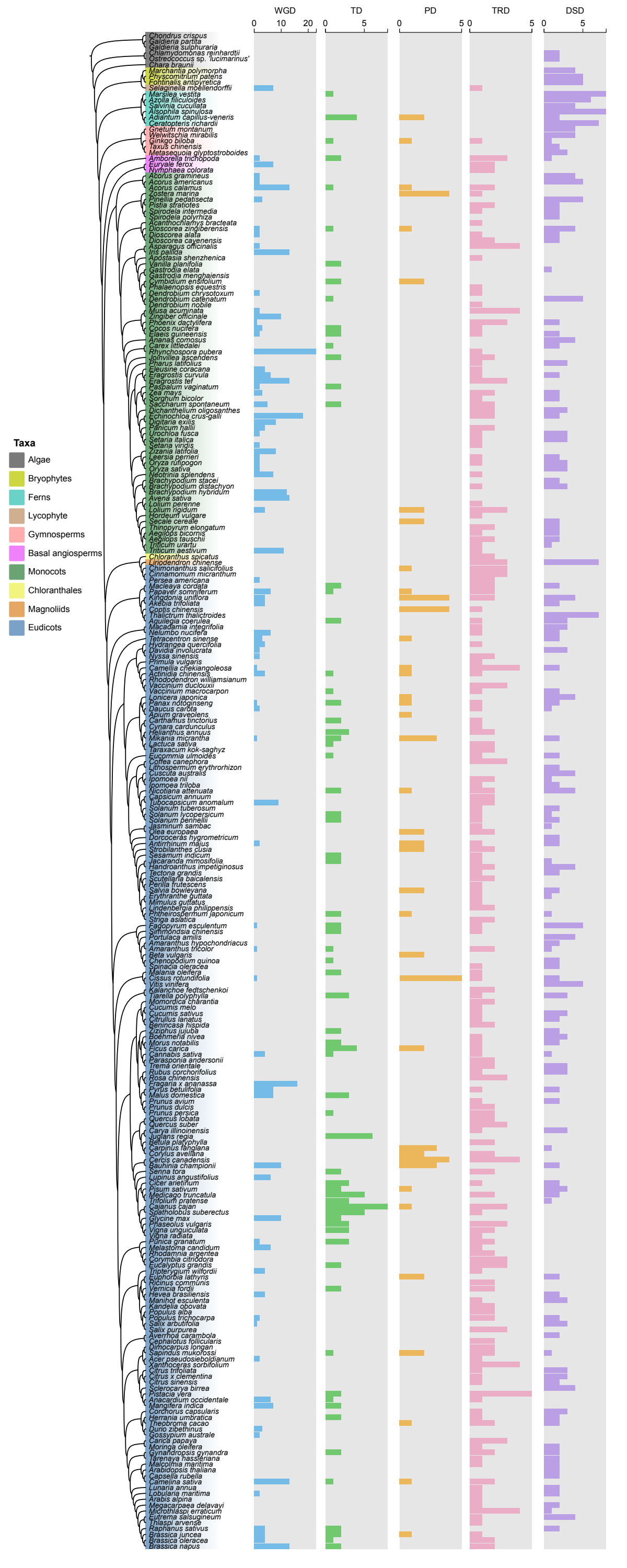

Supplement: Supplementary Table 1 — The genomic data source of 259 species. [file DataSheet1.zip › Supplementary_information/Figure S9.pdf]
