# Supplementary material for: Evolutionary landscape of plant chalcone isomerase-fold gene families
Source: Front Plant Sci. 2025 Mar 28;16:1559547. doi: 10.3389/fpls.2025.1559547 (PMC11985768; doi:10.3389/fpls.2025.1559547)
Supplement: Supplementary Table 1 — The genomic data source of 259 species. [file Figure S1.pdf › Supplementary_information/Figure S1.pdf]

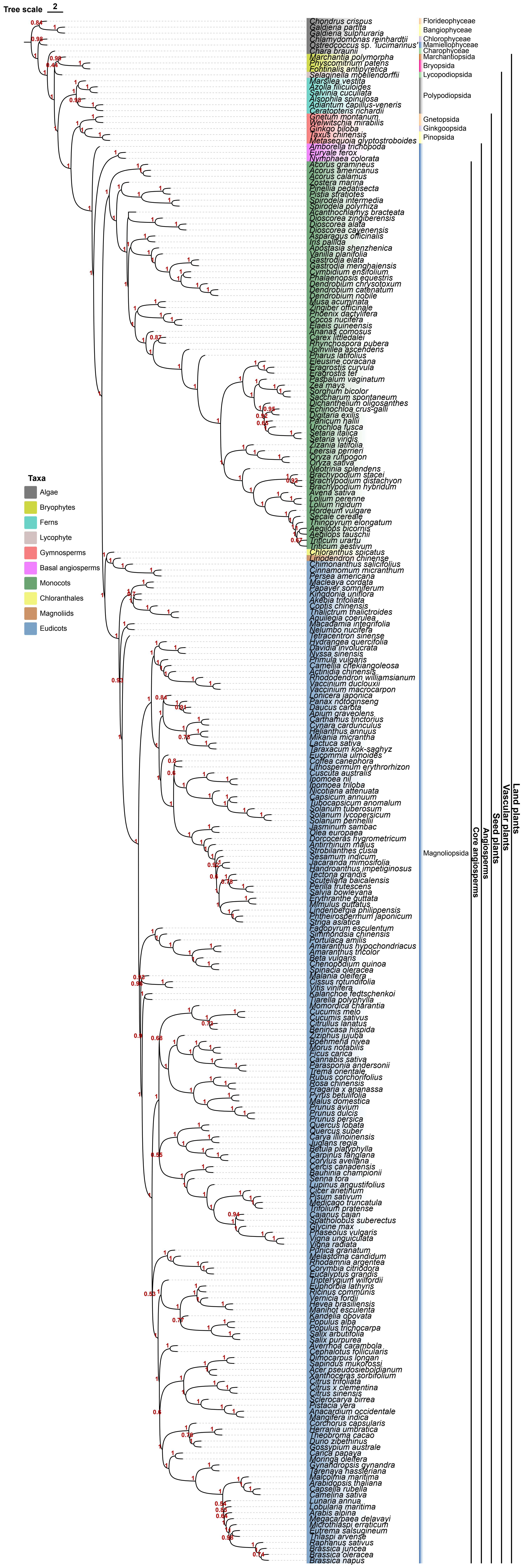

Tree scale 2
Taxa
Algae
Bryophytes
Ferns
Lycophyte
Gymnosperms
Basal angiosperms
Monocots
Chloranthales
Magnoliids
Eudicots
Florideophyceae
Bangiophyceae
Chlorophyceae
Mamiellophyceae
Charophyceae
Marchantiopsida
Bryopsida
Lycopodiopsida
Polypodiopsida
Gnetopsida
Ginkgoopsida
Pinopsida
Magnoliopsida
Land plants
Vascular plants
Seed plants
Angiosperms
Core angiosperms
Chondrus crispus
Galdieria partita
Galdieria sulphuraria
Chlamydomonas reinhardtii
Ostreococcus sp. 'lucimarinus'
Chara braunii
Marchantia polymorpha
Physcomitrium patens
Fontinalis antipyretica
Selaginella moellendorffii
Marsilea vestita
Azolla filiculoides
Salvinia cucullata
Alsophila spinulosa
Adiantum capillus-veneris
Ceratopteris richardii
Gnetum montanum
Welwitschia mirabilis
Ginkgo biloba
Taxus chinensis
Metasequoia glyptostroboides
Amborella trichopoda
Euryale ferox
Nymphaea colorata
Acorus gramineus
Acorus americanus
Acorus calamus
Zostera marina
Pinellia pedatisecta
Pistia stratiotes
Spirodela intermedia
Spirodela polyrhiza
Acanthochlamys bracteata
Dioscorea zingiberensis
Dioscorea alata
Dioscorea cayenensis
Asparagus officinalis
Iris pallida
Apostasia shenzhenica
Vanilla planifolia
Gastrodia elata
Gastrodia menghaiensis
Cymbidium ensifolium
Phalaenopsis equestris
Dendrobium chrysotoxum
Dendrobium catenatum
Dendrobium nobile
Musa acuminata
Zingiber officinale
Phoenix dactylifera
Cocos nucifera
Elaeis guineensis
Ananas comosus
Carex littledalei
Rhynchospora pubera
Joinvillea ascendens
Pharus latifolius
Eleusine coracana
Eragrostis curvula
Eragrostis tef
Paspalum vaginatum
Zea mays
Sorghum bicolor
Saccharum spontaneum
Dichanthelium oligosanthes
Echinochloa crus-galli
Digitaria exilis
Panicum hallii
Urochloa fusca
Setaria italica
Setaria viridis
Zizania latifolia
Leersia perrieri
Oryza rufipogon
Oryza sativa
Neotrinia splendens
Brachypodium stacei
Brachypodium distachyon
Brachypodium hybridum
Avena sativa
Lolium perenne
Lolium rigidum
Hordeum vulgare
Secale cereale
Thinopyrum elongatum
Aegilops bicornis
Aegilops tauschii
Triticum urartu
Triticum aestivum
Chloranthus spicatus
Liriodendron chinense
Chimonanthus salicifolius
Cinnamomum micranthum
Persea americana
Macleaya cordata
Papaver somniferum
Kingdonia uniflora
Akebia trifoliata
Coptis chinensis
Thalictrum thalictroides
Aquilegia coerulea
Macadamia integrifolia
Nelumbo nucifera
Tetracentron sinense
Hydrangea quercifolia
Davidia involucrata
Nyssa sinensis
Primula vulgaris
Camellia chekiangoleosa
Actinidia chinensis
Rhododendron williamsianum
Vaccinium duclouxii
Vaccinium macrocarpon
Lonicera japonica
Panax notoginseng
Daucus carota
Apium graveolens
Carthamus tinctorius
Cynara cardunculus
Helianthus annuus
Mikania micrantha
Lactuca sativa
Taraxacum kok-saghyz
Eucommia ulmoides
Coffea canephora
Lithospermum erythrorhizon
Cuscuta australis
Ipomoea nil
Ipomoea triloba
Nicotiana attenuata
Capsicum annuum
Tubocapsicum anomalum
Solanum tuberosum
Solanum lycopersicum
Solanum pennellii
Jasminum sambac
Olea europaea
Dorcoceras hygrometricum
Antirrhinum majus
Strobilanthes cusia
Sesamum indicum
Jacaranda mimosifolia
Handroanthus impetiginosus
Tectona grandis
Scutellaria baicalensis
Perilla frutescens
Salvia bowleyana
Erythranthe guttata
Mimulus guttatus
Lindenbergia philippensis
Phtheirospermum japonicum
Striga asiatica
Fagopyrum esculentum
Simmondsia chinensis
Portulaca amilis
Amaranthus hypochondriacus
Amaranthus tricolor
Beta vulgaris
Chenopodium quinoa
Spinacia oleracea
Malania oleifera
Cissus rotundifolia
Vitis vinifera
Kalanchoe fedtschenkoi
Tiarella polyphylla
Momordica charantia
Cucumis melo
Cucumis sativus
Citrullus lanatus
Benincasa hispida
Ziziphus jujuba
Boehmeria nivea
Morus notabilis
Ficus carica
Cannabis sativa
Parasponia andersonii
Trema orientale
Rubus corchorifolius
Rosa chinensis
Fragaria x ananassa
Pyrus betulifolia
Malus domestica
Prunus avium
Prunus dulcis
Prunus persica
Quercus lobata
Quercus suber
Carya illinoinensis
Juglans regia
Betula platyphylla
Carpinus fangiana
Corylus avellana
Cercis canadensis
Bauhinia championii
Senna tora
Lupinus angustifolius
Cicer arietinum
Pisum sativum
Medicago truncatula
Trifolium pratense
Cajanus cajan
Spatholobus suberectus
Glycine max
Phaseolus vulgaris
Vigna unguiculata
Vigna radiata
Punica granatum
Melastoma candidum
Rhodamnia argentea
Corymbia citriodora
Eucalyptus grandis
Tripterygium wilfordii
Euphorbia lathyris
Ricinus communis
Vernicia fordii
Hevea brasiliensis
Manihot esculenta
Kandelia obovata
Populus alba
Populus trichocarpa
Salix arbutifolia
Salix purpurea
Averrhoa carambola
Cephalotus follicularis
Dimocarpus longan
Sapindus mukorossi
Acer pseudosieboldianum
Xanthoceras sorbifolium
Citrus trifoliata
Citrus x clementina
Citrus sinensis
Sclerocarya birrea
Pistacia vera
Anacardium occidentale
Mangifera indica
Corchorus capsularis
Herrania umbratica
Theobroma cacao
Durio zibethinus
Gossypium australe
Carica papaya
Moringa oleifera
Gynandropsis gynandra
Tarenaya hassleriana
Malcolmia maritima
Arabidopsis thaliana
Capsella rubella
Camelina sativa
Lunaria annua
Lobularia maritima
Arabis alpina
Megacarpaea delavayi
Microthlaspi erraticum
Eutrema salsugineum
Thlaspi arvense
Raphanus sativus
Brassica juncea
Brassica oleracea
Brassica napus
